# Palladium‐Catalyzed Decarbonylative Trifluoromethylation of Acid Fluorides

**DOI:** 10.1002/anie.201800644

**Published:** 2018-03-13

**Authors:** Sinead T. Keaveney, Franziska Schoenebeck

**Affiliations:** ^1^ Institute of Organic Chemistry RWTH Aachen University Landoltweg 1 52074 Aachen Germany

**Keywords:** carboxylic acids, catalysis, density functional calculations, palladium, trifluoromethylation

## Abstract

While acid fluorides can readily be made from widely available or biomass‐feedstock‐derived carboxylic acids, their use as functional groups in metal‐catalyzed cross‐coupling reactions is rare. This report presents the first demonstration of Pd‐catalyzed decarbonylative functionalization of acid fluorides to yield trifluoromethyl arenes (ArCF_3_). The strategy relies on a Pd/Xantphos catalytic system and the supply of fluoride for transmetalation through intramolecular redistribution to the the Pd center. This strategy eliminated the need for exogenous and detrimental fluoride additives and allows Xantphos to be used in catalytic trifluoromethylations for the first time. Our experimental and computational mechanistic data support a sequence in which transmetalation by R_3_SiCF_3_ occurs prior to decarbonylation.

Owing to their wide abundance, stability and relatively low cost, carboxylic acids and their derivatives belong to some of the most attractive functionalities for synthetic manipulations.^1^ They are featured in numerous natural products and are also key fragments resulting from biomass valorization.^2^ As such, strategies to selectively convert carboxylic acids into value‐added functional groups are in high demand. In particular, the introduction of fluorine into organic molecules has been recognized as a strategy to manipulate properties, impacting pharmaceutical, agrochemical and materials chemistry research.^3^ In this context, the introduction of a trifluoromethyl group via Pd^0^/Pd^II^ catalysis belongs to one of the greatest challenges in the cross‐coupling arena (see Figure 1). This is due to several reasons, including the very difficult reductive elimination of ArCF_3_ from the [L_*n*_Pd^II^(Ar)(CF_3_)] intermediate.^4^ This challenging step has only been accomplished by a handful of ligands, of which several, for example, Xantphos^5^ and dfmpe,^6^ are (so far) only effective stoichiometrically, but not in catalysis. This is due to the propensity of the transmetalating “CF_3_ anions” to displace these weaker coordinating ligands.4e, 5c, 7 Moreover, several equivalents of fluoride salt additive are generally required to activate the transmetalating agent, such as R_3_SiCF_3_, and even the smallest parts per million quantities of moisture introduced by these salts can cause catalyst decomposition.4e, 8 As such, Buchwald's catalytic Pd^0^/Pd^II^‐catalyzed trifluoromethylation of aryl chlorides is a major accomplishment.^9^ We report herein our studies to widen the precursor pool for trifluoromethylation from aryl chlorides or bromides^9^, ^10^ to underexplored and easily accessible aryl carboxylic acid derivatives, that is, acid fluorides.

**Figure 1:**
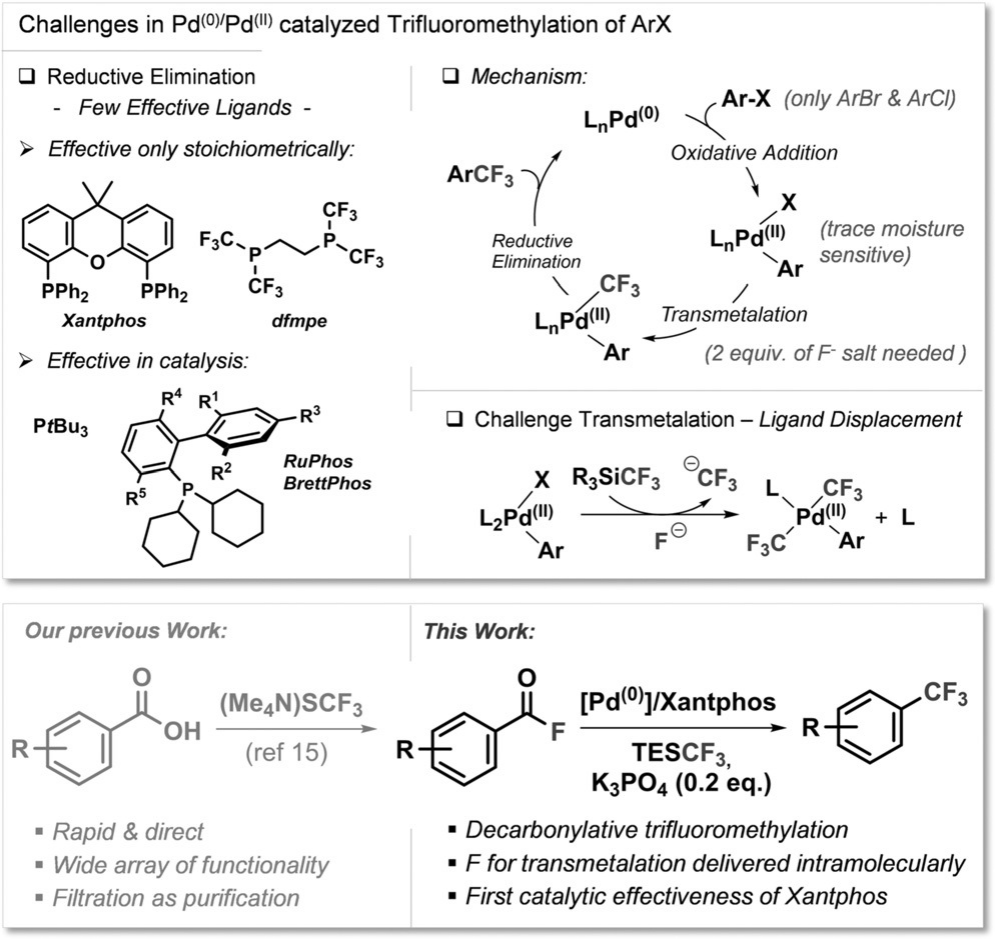
Overview of challenges in Pd^0/^Pd^II^‐catalyzed trifluoromethylation (top) and our work (bottom).

We envisioned that a decarbonylation/trifluoromethylation strategy utilizing carboxylic acid derivatives could circumvent some of the most severe challenges in Pd^0^/Pd^II^ catalyzed trifluoromethylations. If we were able to react acid fluorides with a Pd^0^ source a Pd^II^‐F intermediate would be directly generated (Figure 1), which is the only Pd^II^ intermediate that can be transmetalated directly without the need for an external fluoride additive.5c Thus, the liberation of reactive “CF_3_” anions, which are detrimental to many metal/ligand combinations, would be avoided.4e, 5c, 7a Encouragingly, acid fluorides have previously been coupled to give the corresponding ketones under metal catalysis, as pioneered by Rovis.^11^ However, while the decarbonylation of carboxylic acids and their derivatives1m,1o, 12 (including acid chlorides^13^) has been widely studied, to date, there has been no report of a successful decarbonylation of acid fluorides.

Our group recently reported a facile and direct synthesis of acid fluorides from carboxylic acids,^14^ allowing us to investigate whether a decarbonylation/functionalization protocol could be developed for a wide array of acid fluorides.

We started our investigations with the biaryl acid fluoride **1 a**, utilizing Xantphos as the ligand for Pd. Although Xantphos^15^ has never been effective in catalytic trifluoromethylations, it is one of the few ligands capable of facilitating reductive elimination from [L_*n*_Pd^II^(Ar)(CF_3_)].5b,5c To our delight 15 % of the desired product ArCF_3_ **2 a** (see Table 1) was formed in our initial investigations employing TMSCF_3_ as transmetalating agent. These data indicated that Xantphos is capable of promoting both the decarbonylation and ArCF_3_ reductive elimination steps without the need for exogenous fluoride. Further examination of the reaction conditions (see the Supporting Information) revealed that the highest yield of **2 a** was obtained with TESCF_3_ and a catalytic amount of K_3_PO_4_.1b, 16 When we applied the same reaction conditions to the analogous ArCl and ArBr substrates, the trifluoromethylated products were not generated, indicating that the acid fluoride moiety is key.

**Table 1:** Scope of the decarbonylative trifluoromethylation.

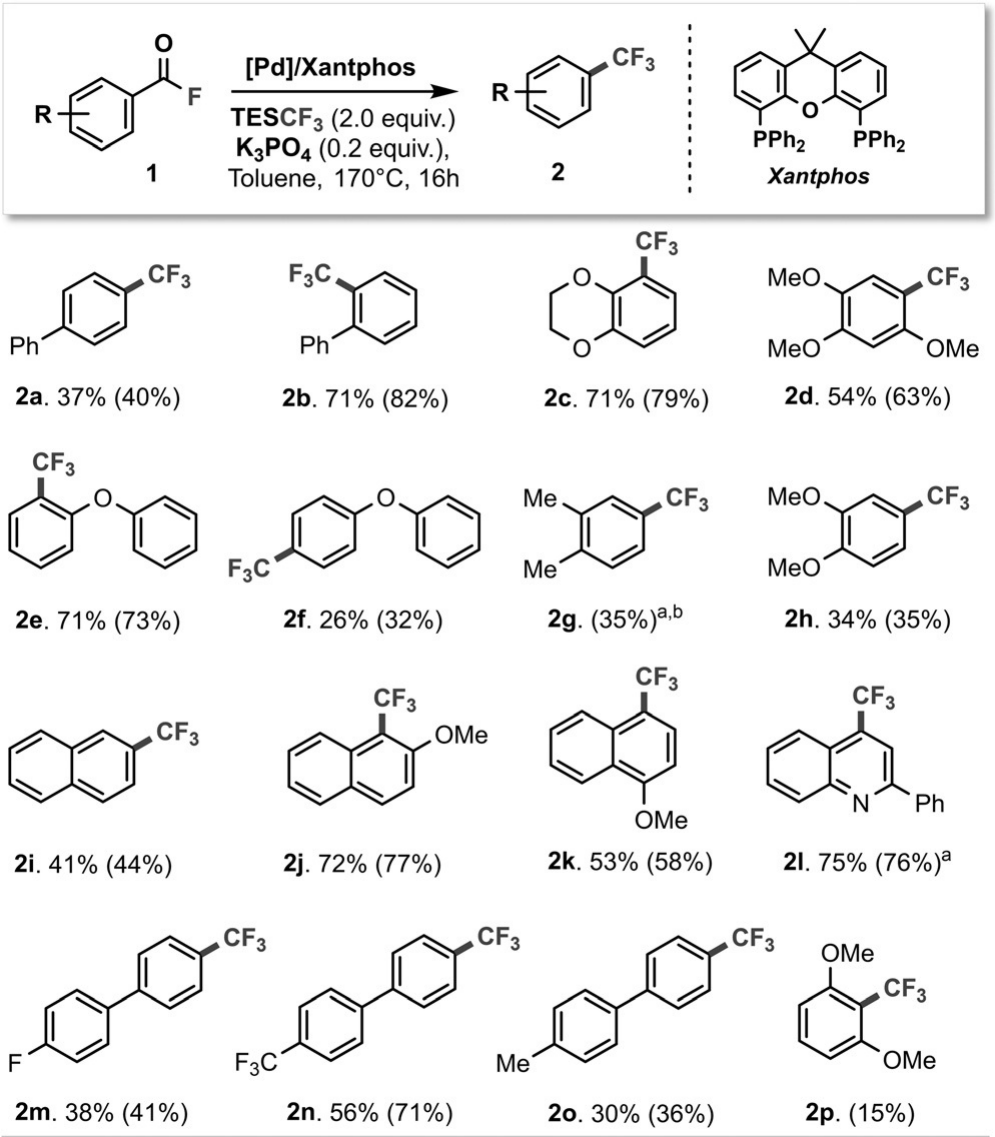

Conditions: **1** (0.4 mmol), [(cinnamyl)PdCl]_2_ (8 mg, 0.016 mmol), Xantphos (28 mg, 0.048 mmol), K_3_PO_4_ (17 mg, 0.08 mmol), TESCF_3_ (160 μL, 0.8 mmol) in toluene (1.2 mL). Isolated yields are shown (conversion in parentheses as determined by ^19^F NMR spectroscopic analysis against internal standard). [a] Reaction performed at 180 °C. [b] Species **2 g** was not isolated due its volatility.

With the successful conditions in hand, we subsequently examined the generality of the method (Table 1). We successfully converted a range of acid fluorides into the corresponding ArCF_3_ compounds, with ether, alkyl and fluorine containing substituents being tolerated. Notably, the acid fluoride derivative of 3,4‐dimethoxybenzoic acid (**1 h**), which is the end‐product from Lignin valorization,^17^ was also successful. The highest yields were obtained for those substrates that possessed *otho*‐substitution to the COF functionality,^18^ in line with reactivity trends previously seen for metal‐catalyzed decarbonylations of other carbonyl derivatives,1j, 19 or trifluoromethylations.^20^ For example, the slight modification from *para*‐ to *ortho*‐linkage of the biphenyl acid fluoride **1 a** (to **1 b**) resulted in a two‐fold increase in yield.

These reactivity features make this a complimentary method to Buchwald's procedure with ArCl.^9^ While 2‐phenylbenzoyl fluoride **1 b** gave 82 % conversion to the desired ArCF_3_ product **2 b** with our protocol, the corresponding 2‐phenyl aryl chloride generated much less of **2 b** with Buchwald's method in our tests (BrettPhos: <5 % conversion, RuPhos: 34 % conversion).

To investigate whether sequential functionalization of carboxylic acids would be possible, we subjected a handful of exemplary substrates (Table 2) to the bench‐stable salt (Me_4_N)SCF_3_ in DCM,^14^ followed by Pd‐catalyzed decarbonylation/trifluoromethylation. This allowed for efficient conversion of carboxylic acids to ArCF_3_ over two steps in good overall yields.

**Table 2:** The two‐step conversion of carboxylic acids **3** to ArCF_3_ **2**.

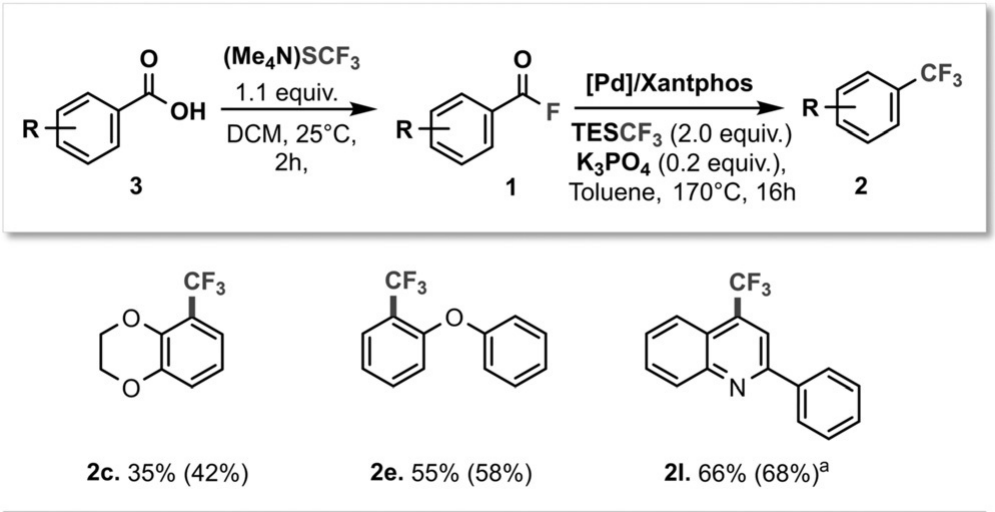

Conditions: **3** (0.4 mmol), (Me_4_N)SCF_3_ (77 mg, 0.44 mmol) in DCM (2 mL), then: the resulting ArCOF **1**, [(cinnamyl)PdCl]_2_ (8 mg, 0.016 mmol), Xantphos (28 mg, 0.048 mmol), K_3_PO_4_ (17 mg, 0.08 mmol), TESCF_3_ (160 μL, 0.8 mmol) in toluene (1.2 mL). Isolated yields are shown (conversion in parentheses as determined by ^19^F NMR spectroscopic analysis against internal standard). [a] Reaction performed at 180 °C.

We next investigated the mechanism of the transformation.^21^ Following the oxidative addition of the acid fluoride to Pd^0^, the corresponding Pd^II^ intermediate could in principle undergo decarbonylation, followed by transmetalation with R_3_SiCF_3_ (Mechanism A, Figure 2). Alternatively, the Pd^II^ intermediate could be transmetalated prior to CO loss to give the CF_3_‐bound Pd^II^CO analog, which could then decarbonylate (Mechanism B). In both cases, a Pd^II^‐F intermediate would be present which would be very prone to transmetalation. We computationally^22^ assessed these possibilities with CPCM (toluene) M06L/def2TZVP//ωB97XD/6‐31G(d)+SDD level of theory at 160 °C and using phenyl acid fluoride as model substrate.^23^ Our data suggest that oxidative addition of the phenyl acid fluoride is relatively facile, proceeding with a barrier of Δ*G* ^≠^=10.1 kcal mol^−1^. As such, the acid fluoride appears to be a promising alternative to acid chlorides, which have been widely used in catalysis but are also much less robust than their fluorinated counterparts.^13^

**Figure 2:**
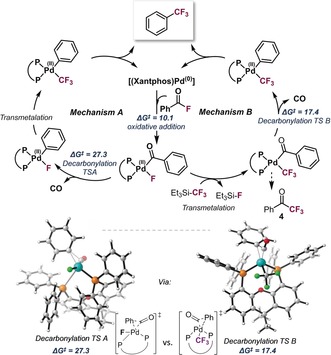
Mechanistic possibilities for ArCOF to ArCF_3_ conversion (top) and computed activation free energy barriers at the CPCM (toluene) M06L/ def2TZVP// ωB97XD/6‐31G(d)+SDD level of theory at 160 °C [Δ*G* ^≠^ given in kcal mol^−1^]. Bottom: illustration of computed decarbonylation transition states.

Decarbonylation from the PhCO‐[Pd^II^]‐F intermediate is predicted to have a free energy barrier of Δ*G* ^≠^=27.3 kcal mol^−1^. Interestingly, the alternative of decarbonylating the already transmetalated complex, that is, PhCO‐[Pd^II^]‐CF_3_, is computed to have a much lower activation free energy barrier of only Δ*G* ^≠^=17.4 kcal mol^−1^. These data indicate that decarbonylation becomes more facile when moving from the F to CF_3_ ligated complex. A distortion/interaction analysis^24^ of the barriers for decarbonylation from both PhCO‐[Pd^II^]‐F and PhCO‐[Pd^II^]‐CF_3_ reveal that the lower barrier for decarbonylation from PhCO‐[Pd^II^]‐CF_3_ arises from the transition state having a more favorable Pd—Xantphos interaction as well as a slightly lower distortion energy, relative to the transition state for decarbonylation from PhCO‐[Pd^II^]‐F (see the Supporting Information for details).^25^

Considering that transmetalation between R_3_SiCF_3_ and Pd^II^‐F has experimentally been shown to occur “within the time of mixing”,5c and that decarbonylation from PhCO‐[Pd^II^]‐CF_3_ is more facile than that from PhCO‐[Pd^II^]‐F, Mechanism B appears to be favored for the conversion of ArCOF to ArCF_3_.

In Mechanism B there is the choice to either decarbonylate and proceed productively towards ArCF_3_ from the intermediate complex ArCO‐[Pd^II^]‐CF_3_, or instead to reductively eliminate directly to the corresponding ketone ArCOCF_3_ **4** (see Scheme 1). Experimentally we observed a strong temperature dependence of the overall product selectivity (see Scheme 1): reacting biphenyl acid fluoride **1 a** at 145 °C under otherwise identical catalysis conditions resulted in a significant portion of biphenyl‐4‐trifluoromethyl ketone **4 a** being formed (32 %) along with the product resulting from CO‐loss, that is, ArCF_3_ **2 a** (in 28 %). A systematic increase of the reaction temperature led to much less of the ketone **4 a** being formed (4 % at 160 °C and 0 % at 180 °C), yielding ArCF_3_ **2 a** as exclusive product at 180 °C (43 % at 160 °C and 46 % at 180 °C).^26^ Given that higher temperature will impact the entropic contributions in the activation free energy barrier, it appears reasonable that more decarbonylation (to ultimately form ArCF_3_) takes place. Computationally, these trends are qualitatively reflected. We calculate an activation free energy difference (ΔΔ*G* ^≠^) of 1.0 kcal mol^−1^ at 25 °C and 1.5 kcal mol^−1^ at 160 °C for the competing pathways of ketone formation versus decarbonylation for substrate **1 a**, with preference for decarbonylation in each case.

**Scheme 1:**
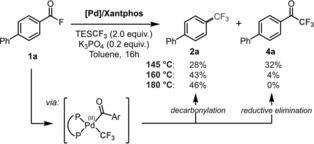
Temperature‐dependent ArCF_3_ **2 a** versus ArCOCF_3_ **4 a** formation.

In conclusion, the first decarbonylative functionalization of acid fluorides to ArCF_3_ compounds was showcased. The strategy relies on the intramolecular supply of the crucial fluoride for transmetalation, allowing Xantphos to be effective in catalytic trifluoromethylations for the first time, as exogenous fluoride and detrimental over‐transmetalation could be avoided. Our computational and experimental reactivity data support a mechanism that involves first transmetalation, followed by decarbonylation. Given that Pd^II^‐F is a key intermediate for selective and additive‐free transmetalations to introduce a range of functionalities (with CF_3_ being the most challenging), this work sets the stage to convert carboxylic acids to a wide array of compounds via the vital acid fluoride intermediate.

## Conflict of interest

The authors declare no conflict of interest.

## Supporting information

As a service to our authors and readers, this journal provides supporting information supplied by the authors. Such materials are peer reviewed and may be re‐organized for online delivery, but are not copy‐edited or typeset. Technical support issues arising from supporting information (other than missing files) should be addressed to the authors.

Supplementary
