# Supplementary material for: Palladium‐Catalyzed Decarbonylative Trifluoromethylation of Acid Fluorides
Source: Angew Chem Int Ed Engl. 2018 Mar 13;57(15):4073–7. doi: 10.1002/anie.201800644 (PMC5900963; doi:10.1002/anie.201800644)
Supplement: Supplementary file 1 — Supplementary [file ANIE-57-4073-s001.pdf]

Angewandte Chemie
Eine Zeitschrift der Gesellschaft Deutscher Chemiker

Supporting Information

Palladium-Catalyzed Decarbonylative Trifluoromethylation of Acid Fluorides

*Sinead T. Keaveney and Franziska Schoenebeck**

anie_201800644_sm_miscellaneous_information.pdf

Supporting Information

Contents:

General information **2**

Experimental procedures and compound characterization data **3**

Synthesis of acyl fluorides *3*

Conversion of acyl fluorides to trifluoromethyl arenes *8*

Two-step conversion of carboxylic acids to trifluoromethyl arenes *13*

Computational details **15**

Summary of calculated barriers at 25 °C and 160 °C *15*

Distortion/Interaction Analysis *17*

Reaction development and control reactions **20**

Examination of different additives and CF_3 sources *20*

Test reactions using $Ar(CO)CF_3$ *20*

Examination of different temperatures *21*

Test reactions using bromo and chloro containing substrates *21*

Test reactions using the conditions reported by Buchwald et al *22*

Control reactions with no catalyst present *22*

Reaction monitoring: formation of $ArCF_3$ over time *23*

NMR Spectra **24**

Acyl fluorides *24*

Trifluoromethyl arenes *48*

References **69**

Energetic data, thermal data and Cartesian coordinates of computed structures **70**

General Structures *70*

ArCOF *72*

$ArCOCF_3$ *72*

$ArCF_3$ *73*

Oxidative addition transition states *74*

$ArCO-[Pd^{(II)}]-F$ intermediates *76*

Decarbonylation from $ArCO-[Pd^{(II)}]-F$ transition states *78*

$Ar-[Pd^{(II)}]-F$ intermediates *81*

$ArCO-[Pd^{(II)}]-CF_3$ intermediates *84*

Decarbonylation from $ArCO-[Pd^{(II)}]-CF_3$ transition states *86*

Reductive elimination from $ArCO-[Pd^{(II)}]-CF_3$ transition states *89*

$Ar-[Pd^{(II)}]-CF_3$ intermediates *91*

Reductive elimination from $Ar-[Pd^{(II)}]-CF_3$ transition states *94*

General information

Reagents and starting materials

Unless otherwise stated, all reagents and starting materials were commercially available and used as received. Triethyl(trifluoromethyl)silane (96 %) was purchased from ChemPur. $(Me_4N)SCF_3$ was prepared according to the reported literature procedure.[1]

Solvents.

Dichloromethane and toluene were purified by the Pure Solvent PS-MD-5 solvent drying system from Innovative Technology. *n*-Hexane and ethyl acetate were technical grade.

Experimental Techniques.

The work-up of all reactions and the isolation of products were carried out in a fume hood using standard techniques. Whether a reaction was performed under an argon or air atmosphere is specified in the experimental procedure.

Characterization.

All ^{1}H NMR, ^{13}C NMR and ^{19}F NMR spectra were recorded at ambient temperature either on Varian V-NMRS 600 or Varian V-NMRS 400 spectrometer. Chemical shifts (δ) are quoted in parts per million (ppm) and were referenced to the residual solvent peak for the ^{1}H and ^{13}C NMR spectra. In some of the ^{13}C NMR spectra there is a peak at 83.3 ppm; this is an artifact from the spectrometer. Coupling constants (*J*) are given in Hz. The resonance multiplicity is described as s (singlet), d (doublet), t (triplet), q (quartet), m (multiplet), dd (doublet of doublets) and br (broad). ^{19}F NMR spectra were recorded using the F-H decoupled pulse sequence from the Varian program library.

High Resolution Mass Spectrometric (HRMS) analyses were performed on a Thermo Scientific LTQ Orbitrap XL (ESI), and on a Finnigan SSQ 7000, EI: 70 eV (EI).

Gas Chromatography - Mass Spectrometry (GC-MS) analyses were performed using an Agilent Technologies 5975 series MSD mass spectrometer coupled with an Agilent Technologies 7820A gas chromatograph (with an Agilent 19091s-433 HP-SMS column (30 m x 0.250 μm x 0.25 μm)).

Experimental procedures and compound characterization data

Synthesis of acyl fluorides

General method A, based on the procedure reported by our group:[2]

Carboxylic acid (0.52 mmol, 1 equiv.) and $(Me_4N)SCF_3$ (100 mg, 0.57 mmol, 1.1 equiv.) were dissolved in dichloromethane (2 mL) under an argon atmosphere, and the reaction mixture was stirred at room temperature for 2 hours. The mixture was then filtered through a pad of celite (3-4 cm in a Pasteur pipette) and the solvent removed under reduced pressure.

General method B, based on a reported literature procedure:[3]

Carboxylic acid (2.8 mmol, 1 equiv.) was added to dichloromethane (20 mL), then XtalFluor-E® (0.78 g, 3.4 mmol, 1.2 equiv.) and triethylamine trihydrofluoride (0.60 mL, 3.4 mmol, 1.2 equiv.) were added sequentially under an air atmosphere. The mixture was stirred at room temperature for 2 hours, after which $NaHCO_3$ (1 M, 20 mL) was added and the mixture stirred vigorously for 1 hour. The reaction mixture was then transferred to a separating funnel and the organic phase collected, and the aqueous phase was extracted with dichloromethane (2 x 30 mL). The organic extracts were combined and washed with water (2 x 60 mL) and brine (1 x 60 mL). The organic phase was then dried using Na_2SO_4, filtered and the solvent removed under reduced pressure.

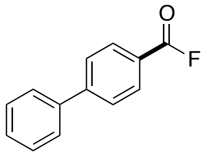

1a

[1,1'-Biphenyl]-4-carbonyl fluoride 1a:

Method B. The reaction was performed on a larger scale, using 7.6 mmol of carboxylic acid. The title compound was obtained after purification using flash column chromatography on silica gel using 5% ethyl acetate in hexane (R_f = 0.43) in 65% yield (988 mg) as a white crystalline solid. M.p. = 95-97 °C. ^{1}H NMR (600 MHz, $CDCl_3$) δ 7.44 (m, 1H), 7.50 (m, 2H), 7.64 (m, 2H), 7.75 (m, 2H), 8.12 (m, 2H). ^{13}C NMR (151 MHz, $CDCl_3$) δ 123.5 (d, J = 61.3 Hz), 127.4, 127.7, 128.8, 129.1, 132.0 (d, J = 3.8 Hz), 139.2, 148.1, 157.4 (d, J = 343.3 Hz). ^{19}F NMR (564 MHz, $CDCl_3$) 18.1 ppm. HRMS (EI): calculated for $C_{13}H_9OF$: 200.06319 [M^+]. Found: 200.06384. The ^{19}F NMR data is in agreement with that reported in the literature,[4] no other data has been previously reported for this compound.

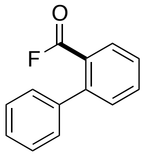

1b

[1,1'-Biphenyl]-2-carbonyl fluoride 1b:

Method B. The title compound was obtained after purification using flash column chromatography on silica gel using 5% ethyl acetate in hexane (R_f = 0.51) in 79% yield

(473 mg) as a colourless oil. ^{1}H NMR (600 MHz, $CDCl_3$) δ 7.35 (m, 2H), 7.45 (m, 4H), 7.51 (m, 2H), 7.68 (m, 1H), 8.04 (m, 1H). ^{13}C NMR (151 MHz, $CDCl_3$) δ 124.2 (d, J = 56.8 Hz), 127.6, 127.9, 128.3, 128.4, 131.7 (d, J = 2.1 Hz), 132.2 (d, J = 2.4 Hz), 133.9, 140.1, 145.5 (m), 157.5 (d, J = 348.1 Hz). ^{19}F NMR (376 MHz, $CDCl_3$) δ 35.0. MS (70 eV, EI): *m/z* (%) 200 (M^+, 100), 183 (4), 170 (22), 152 (28), 126 (5), 104 (10), 76 (13), 63 (4), 51 (4). The data are in agreement with those previously reported in the literature.[2]

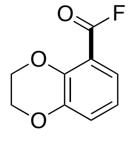

1c

2,3-Dihydrobenzo[*b*][1,4]dioxine-5-carbonyl fluoride 1c:

Method B: The title compound was obtained after purification using flash column chromatography on silica gel using 20% ethyl acetate in hexane (R_f = 0.38) in 36% yield (182 mg) as a white solid. M.p. = 52-54 °C. ^{1}H NMR (400 MHz, $CDCl_3$) δ 4.29 (m, 2H), 4.40 (m, 2H), 6.90 (m, 1H), 7.15 (m, 1H), 7.48 (m, 1H). ^{13}C NMR (151 MHz, $CDCl_3$) δ 63.7, 64.8, 113.5 (d, J = 60.1 Hz), 120.6, 124.1, 125.5 (d, J = 2.4 Hz), 144.3 (d, J = 3.5 Hz), 146.5 (d, J = 4.9 Hz), 154.6 (d, J = 342.8 Hz). ^{19}F NMR (282 MHz, $CDCl_3$) δ 32.2. HRMS (ESI) calculated for $C_9H_7O_3FNa$: 205.02714 $[M+Na]^+$, Found: 205.02673.

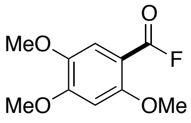

1d

2,4,5-Trimethoxylbenzoyl fluoride 1d:

Method B. The title compound was obtained after purification using flash column chromatography on silica gel using 30% ethyl acetate in hexane (R_f = 0.21) in 59% yield (374 mg) as a white solid. M.p. = 118-120 °C. ^{1}H NMR (400 MHz, $CDCl_3$) δ 3.86 (s, 3H), 3.93 (s, 3H), 3.96 (s, 3H), 6.51 (s, 1H), 7.35 (s, 1H). ^{13}C NMR (151 MHz, $CDCl_3$) δ 56.2, 56.4, 56.6, 96.7 (d, J = 2.6 Hz), 103.7 (d, J = 59.6 Hz), 114.9 (m), 142.7, 153.9, 156.2, 157.4 (d, J = 357.9 Hz). ^{19}F NMR (376 MHz, $CDCl_3$) δ 28.4. HRMS (ESI) calculated for $C_{10}H_{11}O_4FNa$: 237.05336 $[M+Na]^+$. Found: 237.05338.

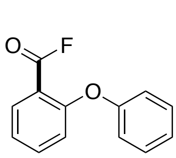

1e

2-Phenoxybenzoyl fluoride 1e:

Method B. The title compound was obtained after purification using flash column chromatography on silica gel using 5% ethyl acetate in hexane (R_f = 0.42) in 63% yield (380 mg) as a colourless oil. ^{1}H NMR (400 MHz, $CDCl_3$) δ 6.92 (m, 1H), 7.04 (m, 2H), 7.18 (m, 2H), 7.37 (m, 2H), 7.55 (m, 1H), 8.00 (dd, J = 7.9, 1.6 Hz 1H). ^{13}C NMR (101 MHz, $CDCl_3$) δ 115.8 (d, J = 59.5 Hz), 119.1 (d, J = 2.8 Hz), 119.6, 122.9, 124.5, 130.0, 133.6 (d, J = 3.0 Hz), 136.4, 154.8 (d, J = 344.0 Hz), 155.8, 159.4 (d, J = 2.9 Hz). ^{19}F NMR (376 MHz, $CDCl_3$) δ 32.2. HRMS (ESI) calculated for $C_{13}H_9O_2FNa$: 239.04788 $[M+Na]^+$. Found: 239.04793.

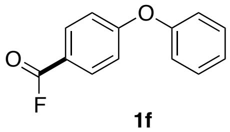

1f

4-Phenoxybenzoyl fluoride 1f:

Method A, the reaction was performed on a 1.04 mmol scale (rather than 0.52 mmol). The title compound was obtained after filtration through a pad of silica (3-4 cm in a Pasteur pipette) using 10% ethyl acetate in hexane in 99% yield (223 mg) as a pale yellow oil. ^{1}H NMR (400 MHz, $CDCl_3$) δ 7.01 (m, 2H), 7.09 (m, 2H), 7.24 (m, 1H), 7.42 (m, 2H), 7.98 (m, 2H). ^{13}C NMR (101 MHz, $CDCl_3$) δ 117.4 (m), 118.5 (d, J = 62.0 Hz), 120.6, 125.3, 130.2, 133.8 (d, J = 4.1 Hz), 154.7, 156.9 (d, J = 340.9 Hz), 164.0. ^{19}F NMR (376 MHz, $CDCl_3$) δ 16.8. HRMS (ESI) calculated $C_{13}H_9O_2FNa$: 239.04788 $[M+Na]^+$. Found: 239.04739.

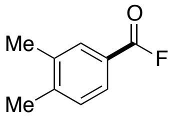

1g

3,4-Dimethylbenzoyl fluoride 1g:

Method B: The title compound was obtained after purification using flash column chromatography on silica gel using 5% ethyl acetate in hexane (R_f = 0.65) in 76% yield (324 mg) as a colourless oil. ^{1}H NMR (400 MHz, $CDCl_3$) δ 2.31 (s, 3H), 2.33 (s, 3H), 7.25 (m, 1H), 7.74 (m, 1H), 7.77 (s, 1H). ^{13}C NMR (101 MHz, $CDCl_3$) δ 19.6, 20.2, 122.3 (d, J = 60.3 Hz), 129.0 (d, J = 3.9 Hz), 130.3 (m), 132.3 (d, J = 4.2 Hz), 137.6 (d, J = 1.3 Hz), 145.3, 157.6 (d, J = 342.8 Hz). ^{19}F NMR (376 MHz, $CDCl_3$) δ 17.3. HRMS (EI) calculated for C_9H_9OF: 152.06319 $[M^+]$, Found: 152.06382.

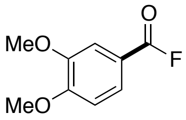

1h

3,4-Dimethoxylbenzoyl fluoride 1h:

Method B. The title compound was obtained after purification using flash column chromatography on silica gel using 40% ethyl acetate in hexane (R_f = 0.57) in 78% yield (402 mg) as a white solid. M.p. = 61-63 °C. ^{1}H NMR (600 MHz, $CDCl_3$) δ 3.94 (s, 3H), 3.97 (s, 3H), 6.95 (d, J = 8.5 Hz, 1H), 7.48 (m, 1H), 7.71 (dd, J = 8.4,1.5 Hz, 1H). ^{13}C NMR (151 MHz, $CDCl_3$) δ 56.1, 56.2, 110.6, 113.0 (d, J = 4.5 Hz), 116.9 (d, J = 62.0 Hz), 126.3 (d, J = 3.1 Hz), 149.1, 155.0, 157.4 (d, J = 340.0 Hz). ^{19}F NMR (376 MHz, $CDCl_3$) δ 15.6. HRMS (ESI) calculated for $C_9H_9O_3FNa$: 207.04288 $[M+Na]^+$. Found: 207.04279.

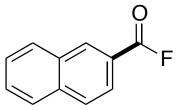

1i

2-Naphthoyl fluoride 1i:

Method B. The title compound was obtained after purification using flash column chromatography on silica gel using 5% ethyl acetate in hexane (R_f = 0.54) in 54% yield (265 mg) as a white solid. M.p. = 51-52 °C. ^{1}H NMR (600 MHz, $CDCl_3$) δ 7.62 (m, 1H), 7.69 (m, 1H), 7.94 (m, 2H), 8.01 (m, 2H), 8.64 (s, 1H). ^{13}C NMR (151 MHz, $CDCl_3$) δ 122.0 (d, J = 60.4 Hz), 125.6 (d, J = 4.1 Hz), 127.4, 127.9, 129.0, 129.6, 129.7, 132.3, 134.0 (d, J = 3.1 Hz), 136.4, 157.6 (d, J = 343.6 Hz). ^{19}F NMR (376 MHz, $CDCl_3$) δ 18.0. MS (70 eV, EI): *m/z* (%) 174 (M^+, 100), 155

(8), 146 (74), 126 (23), 99 (4), 87 (4), 73 (8), 63 (7), 51 (3). The data are in agreement with those previously reported in the literature.[5]

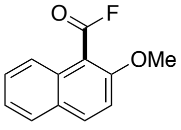

1j

2-Methoxy-1-naphthoyl fluoride 1j:

Method B: The title compound was obtained after purification using flash column chromatography on silica gel using 20% ethyl acetate in hexane (R_f = 0.45) in 76% yield (435 mg) as a white solid. M.p. = 77-79 °C. ^{1}H NMR (600 MHz, $CDCl_3$) δ 4.04 (s, 3H), 7.31 (m, 1H), 7.43 (m, 1H), 7.60 (m, 1H), 7.82 (m, 1H), 8.03 (m, 1H), 8.12 (m, 1H). ^{13}C NMR (151 MHz, $CDCl_3$) δ 56.8, 109.9 (d, *J* = 56.7 Hz), 112.7, 123.7, 124.6, 128.4 (d, *J* = 1.4 Hz), 128.5, 128.9, 131.6 (d, *J* = 1.5 Hz), 135.2, 157.1 (d, *J* = 351.2 Hz), 158.2 (d, *J* = 2.0 Hz). ^{19}F NMR (564 MHz, $CDCl_3$) δ 53.6. HRMS (ESI) calculated for $C_{12}H_9O_2FNa$: 227.04788 $[M+Na]^+$, Found: 227.04784.

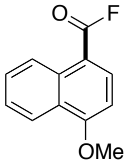

1k

4-Methoxy-1-naphthoyl fluoride 1k:

Method B: The title compound was obtained without further purification in 92% yield (523 mg) as a white solid. M.p. = 60-62 °C. ^{1}H NMR (600 MHz, $CDCl_3$) δ 4.10 (s, 3H), 6.85 (d, *J* = 8.4 Hz, 1H), 7.57 (m, 1H), 7.71 (m, 1H), 8.36 (m, 2H), 9.04 (d, *J* = 8.7 Hz, 1H). ^{13}C NMR (151 MHz, $CDCl_3$) δ 56.1, 102.8, 112.0 (d, *J* = 56.2 Hz), 122.7, 125.1, 125.7 (d, *J* = 4.1 Hz), 126.2, 129.6, 133.5 (d, *J* = 7.7 Hz), 136.1 (d, *J* = 1.9 Hz), 156.3 (d, *J* = 339.4 Hz), 161.8. ^{19}F NMR (564 MHz, $CDCl_3$) δ 25.8. HRMS (ESI) calculated for $C_{12}H_9O_2FNa$: 227.04788 $[M+Na]^+$, Found: 227.04762.

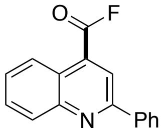

1l

2-Phenylquinoline-4-carbonyl fluoride 1l:

Method A. The title compound was obtained after filtration through a pad of silica (3-4 cm in a Pasteur pipette) using 30% ethyl acetate in hexane in 96% yield (125 mg) as an off-white solid. M.p. = 91-93 °C. ^{1}H NMR (600 MHz, CD_3CN) δ 7.58 (m, 3H), 7.79 (m, 1H), 7.91 (m, 1H), 8.24 (d, *J* = 8.4 Hz, 1H), 8.30 (m, 2H), 8.61 (s, 1H), 8.76 (d, *J* = 8.6 Hz, 1H). ^{13}C NMR (151 MHz, CD_3CN) δ 122.5, 123.6 (d, *J* = 6.1 Hz), 124.5, 127.3, 129.0, 129.1, 129.5 (d, *J* = 59.4 Hz), 130.3, 130.4, 130.7, 137.8, 149.0 (m), 155.7 (d, *J* = 348.8 Hz), 156.5. ^{19}F NMR (376 MHz, $CDCl_3$) δ 32.4. MS (70 eV, EI): *m/z* (%) 251 (M^+, 100), 222 (22), 204 (68), 176 (9), 151 (3), 111 (11), 75 (13), 51 (7). The data are in agreement with those previously reported in the literature.[2]

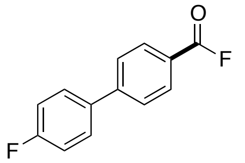

1m

4'-Fluoro-[1,1'-biphenyl]-4-carbonyl fluoride 1m:

Method B. The title compound was obtained after purification using flash column chromatography on silica gel using 5% ethyl acetate in hexane (R_f = 0.48) in 62% yield (377 mg) as a white solid. M.p. = 103-105 °C. ^{1}H NMR (600 MHz, $CDCl_3$) δ 7.18 (m, 2H), 7.61 (m, 2H), 7.70 (m, 2H), 8.11 (m, 2H). ^{13}C NMR (151 MHz, $CDCl_3$) δ 116.1 (d, *J* = 21.3 Hz), 123.5 (d, *J* = 61.6 Hz), 127.5, 129.1 (d, *J* = 8.4 Hz), 132.0 (d, *J* = 3.6 Hz), 135.4 (d, *J* = 2.9 Hz), 147.0, 157.3 (d, *J* = 343.7 Hz), 163.3 (d, *J* = 249.0 Hz). ^{19}F NMR (564 MHz, $CDCl_3$) δ -113.0 (s, 1F), 18.2 (s, 1F). HRMS (EI) calculated for $C_{13}H_8OF_2$: 218.05377 [M^+], Found: 218.05368.

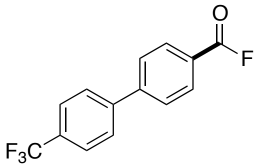

1n

4'-Trifluoromethyl-[1,1'-biphenyl]-4-carbonyl fluoride 1n:

Method B. The title compound was obtained after purification using flash column chromatography on silica gel using 5% ethyl acetate in hexane (R_f = 0.41) in 34% yield (257 mg) as a white solid. M.p. = 102-104 °C. ^{1}H NMR (600 MHz, $CDCl_3$) δ 7.75 (m, 6H), 8.16 (m, 2H). ^{13}C NMR (151 MHz, $CDCl_3$) δ 124.0 (q, *J* = 272.1 Hz), 124.4 (d, *J* = 61.7 Hz), 126.0 (q, *J* = 3.7 Hz), 127.7, 127.9, 130.8 (q, *J* = 33.0 Hz), 132.1 (d, *J* = 3.6 Hz), 142.8, 146.5, 157.1 (d, *J* = 343.7 Hz). ^{19}F NMR (564 MHz, $CDCl_3$) δ -62.7 (s, 3F), 18.6 (s, 1F). HRMS (EI) calculated for $C_{14}H_8OF_4$: 268.05058 [M^+], Found: 268.05088.

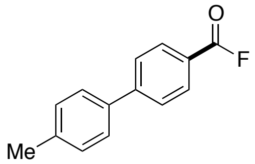

1o

4'-Methyl-[1,1'-biphenyl]-4-carbonyl fluoride 1o:

Method B. The title compound was obtained without further purification in 81% yield (486 mg) as a white solid. M.p. = 123-124 °C. ^{1}H NMR (600 MHz, $CDCl_3$) δ 2.42 (s, 3H), 7.30 (m, 2H), 7.54 (m, 2H), 7.73 (m, 2H), 8.10 (m, 2H). ^{13}C NMR (151 MHz, $CDCl_3$) δ 21.2, 123.1 (d, *J* = 61.2 Hz), 127.2, 127.4, 129.8, 132.0 (d, *J* = 3.7 Hz), 136.3, 138.9, 148.0, 157.4 (d, *J* = 342.8 Hz). ^{19}F NMR (376 MHz, $CDCl_3$) δ 17.9. HRMS (EI) calculated for $C_{14}H_{11}OF$: 214.07884 [M^+], Found: 214.07926.

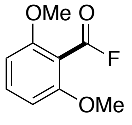

1p

2,6-Dimethoxybenzoyl fluoride 1p:

Method B: The title compound was obtained without further purification in 97% yield (499 mg) as a white solid. M.p. = 66-68 °C. ^{1}H NMR (600 MHz, $CDCl_3$) δ 3.85 (s, 6H), 6.58 (d, *J* = 8.5 Hz, 2H), 7.40 (m, 1H). ^{13}C NMR (151 MHz, $CDCl_3$) δ 56.2, 103.9, 106.7 (d, *J* = 57.6 Hz), 133.9, 155.5 (d, *J* = 350.1 Hz), 159.1. ^{19}F NMR (282 MHz, $CDCl_3$) δ 53.6. HRMS (ESI) calculated for $C_9H_9O_3FNa$: 207.04279 [$M+Na]^+$, Found: 207.04282.

Conversion of acyl fluorides to trifluoromethyl arenes

General method:

Acyl fluoride* (0.4 mmol, 1 equiv.), [(cinnamyl)PdCl]$_2$ (8 mg, 0.016 mmol, 0.04 equiv.), Xantphos (28 mg, 0.048 mmol, 0.12 equiv.) and potassium phosphate (17 mg, 0.08 mmol, 0.2 equiv.) were added to a 20 mL pressure tube under an argon atmosphere. Toluene (1.2 mL) was then added, followed by triethyl(trifluoromethyl)silane (160 µL, 0.8 mmol, 2 equiv.). The tube was then sealed and the reaction mixture heated with vigorous stirring at 170 °C for 16 hours. The reaction mixture was then allowed to cool to room temperature, 4-(trifluoromethoxy)anisole (60 µL, 0.4 mmol, 1 equiv.) was added as an internal standard and the mixture filtered through a pad of celite (3-4 cm in a Pasteur pipette). The crude reaction mixture was then analyzed using GC-MS and ^{19}F NMR spectroscopic analysis.

* *If the acyl fluoride was a liquid it was added to the reaction mixture after the addition of toluene.*

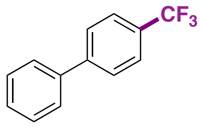
2a

4-(Trifluoromethyl)-1,1'-biphenyl 2a:

The title compound was obtained after purification using flash column chromatography on silica gel using hexane (R_f = 0.47) in 40% yield (37 mg) as a white solid. M.p. = 63-65 °C. ^{1}H NMR (400 MHz, $CDCl_3$) δ 7.39 (m, 1H), 7.46 (m, 2H), 7.58 (m, 2H), 7.68 (m, 4H). ^{13}C NMR (151 MHz, $CDCl_3$) δ 124.3 (q, J = 271.9 Hz), 125.7 (q, J = 3.6 Hz), 127.3, 127.4, 128.2, 129.0, 130.3 (m), 139.8, 144.7. ^{19}F NMR (376 MHz, $CDCl_3$) δ -62.4. MS (70 eV, EI): *m/z* (%) 222 (M^+, 100), 201 (10), 172 (4), 152 (24), 126 (2), 111 (2), 86 (4), 63 (2), 51 (2). The data are in agreement with those previously reported in the literature.[6,7]

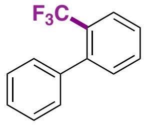
2b

2-(Trifluoromethyl)-1,1'-biphenyl 2b:

The title compound was obtained after purification using flash column chromatography on silica gel using hexane (R_f = 0.48) in 71% yield (63 mg) as a colourless oil. ^{1}H NMR (400 MHz, $CDCl_3$) δ 7.32 (m, 3H), 7.39 (m, 3H), 7.45 (m, 1H), 7.55 (m, 1H), 7.74 (m, 1H). ^{13}C NMR (151 MHz, $CDCl_3$) δ 124.1 (q, J = 274.2 Hz), 126.0 (q, J = 5.3 Hz), 127.3, 127.6, 127.7, 128.4 (q, J = 29.9 Hz), 128.9, 131.3, 132.0, 139.8, 141.4. ^{19}F NMR (564 MHz, $CDCl_3$) δ -56.8. MS (70 eV, EI): *m/z* (%) 222 (M^+, 100), 201 (46), 183 (14), 152 (17), 126 (5), 101 (4), 75 (4), 51 (3). The data are in agreement with those previously reported in the literature.[6]

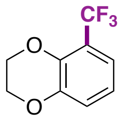

2c

5-(Trifluoromethyl)-2,3-dihydrobenzo[*b*][1,4]dioxine 2c:
The title compound was obtained after purification using flash column chromatography on silica gel using 5% ethyl acetate in hexane (R_f = 0.33) in 71% yield (58 mg) as a colourless oil. ^{1}H NMR (400 MHz, $CDCl_3$) δ 4.29 (m, 2H), 4.34 (m, 2H), 6.86 (m, 1H), 7.02 (m, 1H), 7.12 (m, 1H). ^{13}C NMR (151 MHz, $CDCl_3$) δ 64.0, 64.4, 118.8 (q, *J* = 31.2 Hz), 118.9 (q, *J* = 5.2 Hz), 120.4, 121.0, 123.4 (q, *J* = 272.7 Hz), 141.9 (m), 144.1. ^{19}F NMR (564 MHz, $CDCl_3$) δ -61.9. HRMS (EI) calculated for $C_9H_7O_2F_3$: 204.03927 [M^+], Found: 204.03900.

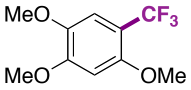

2d

1,2,4-Trimethoxy-5-(trifluoromethyl)benzene 2d:
The title compound was obtained after purification using flash column chromatography on silica gel using 20% ethyl acetate in hexane (R_f = 0.29) in 54% yield (51 mg) as a white solid. M.p. = 73-74 °C. ^{1}H NMR (400 MHz, $CDCl_3$) δ 3.84 (s, 3H), 3.86 (s, 3H), 3.91 (s, 3H), 6.56 (s, 1H), 7.03 (s, 1H). ^{13}C NMR (151 MHz, $CDCl_3$) δ 56.1, 56.6, 56.8, 97.9, 110.0 (q, *J* = 31.3 Hz), 110.3 (q, *J* = 5.2 Hz), 123.8 (q, *J* = 271.3 Hz), 142.4, 152.5, 152.6. ^{19}F NMR (564 MHz, $CDCl_3$) δ -60.7. MS (70 eV, EI): *m/z* (%) 236 (M^+, 100), 221 (77), 217 (13), 193 (71), 178 (10), 163 (9), 147 (14), 120 (16), 81 (5), 69 (20), 53 (6). The data are in agreement with those previously reported in the literature.[8]

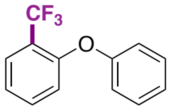

2e

1-Phenoxy-2-(trifluoromethyl)benzene 2e:
The title compound was obtained after purification using flash column chromatography on silica gel using hexane (R_f = 0.29) in 71% yield (67 mg) as a colourless oil. ^{1}H NMR (400 MHz, $CDCl_3$) δ 6.91 (d, *J* = 8.3 Hz, 1H), 7.03 (m, 2H), 7.14 (m, 2H), 7.35 (m, 2H), 7.43 (m, 1H), 7.66 (m, 1H). ^{13}C NMR (101 MHz, $CDCl_3$) δ 119.1, 119.4, 121.3 (q, *J* = 31.2 Hz), 122.6, 123.4 (q, *J* = 272.6 Hz), 124.1, 127.2 (q, *J* = 5.0 Hz), 129.9, 133.2, 155.5 (m), 156.4. ^{19}F NMR (376 MHz, $CDCl_3$) δ -62.0. MS (70 eV, EI): *m/z* (%) 238 (M^+, 100), 217 (8), 199 (9), 169 (21), 141 (16), 114 (18), 95 (5), 77 (44), 51 (21). The data are in agreement with those previously reported in the literature.[9]

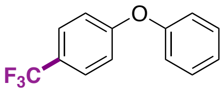

2f

1-Phenoxy-4-(trifluoromethyl)benzene 2f:
The title compound was obtained after purification using flash column chromatography on silica gel using hexane (R_f = 0.39) in 26% yield (24 mg) as a colourless oil. ^{1}H NMR (400 MHz, $CDCl_3$) δ 7.04 (m, 4H), 7.18 (m, 1H), 7.38 (m, 2H), 7.56 (m, 2H). ^{13}C NMR (151 MHz, $CDCl_3$) δ 117.8, 119.9, 124.2 (q, *J* = 271.4 Hz), 124.5, 124.8 (q, *J* = 32.7 Hz), 127.1 (q, *J* = 3.7 Hz), 130.1, 155.7, 160.5 (m). ^{19}F NMR (564 MHz, $CDCl_3$) δ -61.8. MS (70 eV, EI):

m/z (%) 238 (M^+, 100), 210 (10), 189 (3), 169 (9), 141 (26), 115 (4), 95 (4), 77 (30), 51 (13). The data are in agreement with those previously reported in the literature.[9]

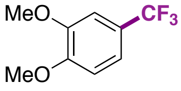

2h

1,2-Dimethoxy-4-(trifluoromethyl)benzene 2h:

The title compound was obtained after purification using flash column chromatography on silica gel using 20% ethyl acetate in hexane (R_f = 0.44) in 34% yield (28 mg) as a colourless oil. ^{1}H NMR (600 MHz, $CDCl_3$) δ 3.92 (s, 6H), 6.91 (d, J = 8.4 Hz, 1H), 7.07 (m, 1H), 7.21 (d, J = 8.4 Hz, 1H). ^{13}C NMR (151 MHz, $CDCl_3$) δ 56.0 (overlap of two peaks), 108.0 (q, J = 3.5 Hz), 110.5, 118.3 (q, J = 4.1 Hz), 122.9 (q, J = 32.8 Hz), 124.3 (q, J = 271.2 Hz), 149.0, 151.5. ^{19}F NMR (564 MHz, $CDCl_3$) δ -61.6. MS (70 eV, EI): m/z (%) 206 (M^+, 100), 191 (23), 163 (25), 143 (49), 120 (13), 95 (8). 75 (5), 63 (5), 51 (4). The data are in agreement with those previously reported in the literature.[8,10]

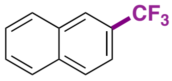

2i

2-(Trifluoromethyl)naphthalene 2i:

The title compound was obtained after purification using flash column chromatography on silica gel using hexane (R_f = 0.62) in 41% yield (32 mg) as a white solid. M.p. = 55-57 °C. ^{1}H NMR (600 MHz, $CDCl_3$) δ 7.63 (m, 3H), 7.93 (m, 3H), 8.16 (s, 1H). ^{13}C NMR (151 MHz, $CDCl_3$) δ 121.4 (q, J = 3.0 Hz), 124.4 (q, J = 272.0 Hz), 125.7 (q, J = 4.5 Hz), 127.1, 127.6 (q, J = 32.2 Hz), 127.8, 128.0, 128.8, 129.0, 132.2, 134.5. ^{19}F NMR (564 MHz, $CDCl_3$) δ -62.3. MS (70 eV, EI): m/z (%) 196 (M^+, 100), 177 (21), 146 (30), 127 (8), 98 (4), 75 (4), 51 (2). The data are in agreement with those previously reported in the literature.[11]

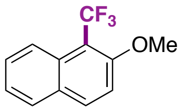

2j

2-Methoxy-1-(trifluoromethyl)naphthalene 2j:

The title compound was obtained after purification using flash column chromatography on silica gel using 5% ethyl acetate in hexane (R_f = 0.38) in 72% yield (65 mg) as a pale yellow solid. M.p. = 52-54 °C. ^{1}H NMR (600 MHz, $CDCl_3$) δ 4.01 (s, 3H), 7.32 (d, J = 9.1 Hz, 1H), 7.42 (m, 1H), 7.56 (m, 1H), 7.81 (d, J = 8.2 Hz, 1H), 7.98 (d, J = 9.1 Hz, 1H), 8.23 (m, 1H). ^{13}C NMR (151 MHz, $CDCl_3$) δ 57.2, 111.0 (q, J = 28.9 Hz), 114.1, 123.9 (q, J = 5.2 Hz), 124.2, 125.4 (q, J = 275.8 Hz), 128.1, 128.6, 129.0, 131.0, 134.3, 156.9 (m). ^{19}F NMR (564 MHz, $CDCl_3$) δ -52.6. HRMS (ESI) calculated for $C_{12}H_{10}OF_3$: 227.06783 $[M+H]^+$, Found: 227.06712.

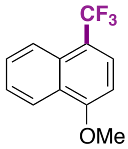

2k

1-Methoxy-4-(trifluoromethyl)naphthalene 2k:

The title compound was obtained after purification using flash column chromatography on silica gel using 5% ethyl acetate in hexane (R_f = 0.48) in 53% yield (48 mg) as a yellow

oil. ^{1}H NMR (600 MHz, $CDCl_3$) δ 4.04 (s, 3H), 6.77 (d, *J* = 8.2 Hz, 1H), 7.56 (m, 1H), 7.64 (m, 1H), 7.80 (d, *J* = 8.2 Hz, 1H), 8.15 (m, 1H), 8.36 (d, *J* = 8.5 Hz, 1H). ^{13}C NMR (151 MHz, $CDCl_3$) δ 55.7, 101.7, 118.3 (q, *J* = 30.2 Hz), 122.7, 124.0, 125.1 (q, *J* = 272.1 Hz), 125.7 (q, *J* = 6.1 Hz), 125.9, 125.9, 128.0, 130.1, 158.4. ^{19}F NMR (564 MHz, CDCl3) δ -59.0. HRMS (ESI) calculated for $C_{12}H_{10}OF_3$: 227.06783 $[M+H]^+$, Found: 227.06721

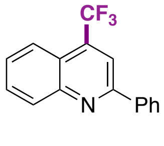

2l

2-Phenyl-4-(trifluoromethyl)quinoline 2l:

The reaction was performed at 180 °C for 16 hours, not 170 °C. The title compound was obtained after purification using flash column chromatography on silica gel using 5% ethyl acetate in hexane (R_f = 0.17) in 75% yield (82 mg) as a pale yellow solid. M.p. = 54-56 °C. ^{1}H NMR (400 MHz, $CDCl_3$) δ 7.52 (m, 3H), 7.65 (m, 1H), 7.81 (m, 1H), 8.15 (m, 4H), 8.26 (m, 1H). ^{13}C NMR (151 MHz, $CDCl_3$) δ 116.0 (q, *J* = 5.4 Hz), 121.8, 123.6 (q, *J* = 274.8 Hz), 123.8, 127.5, 127.9, 129.0, 130.0, 130.4, 130.6, 135.0 (q, *J* = 31.5 Hz), 138.4, 149.1, 156.6. ^{19}F NMR (376 MHz, $CDCl_3$) δ -61.5. MS (70 eV, EI): *m/z* (%) 273 (M^+, 100), 254 (4), 222 (3), 204 (53), 176 (8), 151 (3), 127 (6), 102 (7), 75 (6), 51 (4). The data are in agreement with those previously reported in the literature.[12,13]

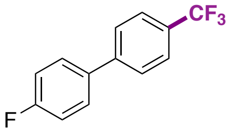

2m

4-Fluoro-4’-trifluoromethyl-[1,1’-biphenyl] 2m:

The title compound was obtained after purification using flash column chromatography on silica gel using hexane (R_f = 0.48) in 38% yield (36 mg) as a colourless oil. ^{1}H NMR (400 MHz, $CDCl_3$) δ 7.14 (m, 2H), 7.54 (m, 2H), 7.66 (m, 4H). ^{13}C NMR (151 MHz, $CDCl_3$) δ 115.9 (d, *J* = 21.3 Hz), 124.2 (q, *J* = 271.9 Hz), 125.8 (q, *J* = 3.7 Hz), 127.3, 128.9 (d, *J* = 8.2 Hz), 129.4 (q, *J* = 32.6 Hz), 135.9 (d, *J* = 3.3 Hz), 143.7, 162.9 (d, *J* = 247.8 Hz). ^{19}F NMR (564 MHz, $CDCl_3$) δ -114.2, -62.5. MS (70 eV, EI): *m/z* (%) 240 (M^+, 100), 221 (15), 190 (9), 170 (28), 151 (4), 120 (4), 95 (5), 75 (3). The data are in agreement with those previously reported in the literature.[14]

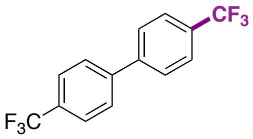

2n

4,4'-Bis(trifluoromethyl)-1,1'-biphenyl 2n:

The title compound was obtained after purification using flash column chromatography on silica gel using hexane (R_f = 0.55) in 56% yield (65 mg) as a white solid. M.p. = 81-82 °C. ^{1}H NMR (400 MHz, $CDCl_3$) δ 7.70 (m, 8H). ^{13}C NMR (151 MHz, $CDCl_3$) δ 124.1 (q, *J* = 272.2 Hz), 125.9 (q, *J* = 3.7 Hz), 127.6, 130.3 (q, *J* = 32.6 Hz), 143.2. ^{19}F NMR (564 MHz, $CDCl_3$) δ -62.6. MS (70 eV, EI): *m/z* (%) 290 (M^+, 100), 271 (23),

240 (9), 219 (6), 201 (15), 170 (4), 152 (11), 120 (3), 95 (4), 75 (2). The data are in agreement with those previously reported in the literature. [14,15]

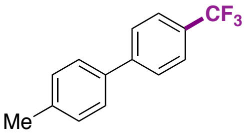

2o

4-Methyl-4'-(trifluoromethyl)-1,1'-biphenyl 2o:

The title compound was obtained after purification using flash column chromatography on silica gel using hexane (R_f = 0.56) in 30% yield (22 mg) as a white solid. M.p. = 122-124 °C. ^{1}H NMR (600 MHz, $CDCl_3$) δ 2.42 (s, 3H), 7.29 (m, 2H), 7.51 (m, 2H), 7.68 (s, 4H). ^{13}C NMR (151 MHz, $CDCl_3$) δ 21.1, 124.3 (q, J = 271.8 Hz), 125.6 (q, J = 3.8 Hz), 127.1, 127.2, 129.0 (q, J = 32.6 Hz), 129.7, 136.8, 138.1, 144.6. ^{19}F NMR (564 MHz, $CDCl_3$) δ -62.4. MS (70 eV, EI): *m/z* (%) 236 (M^+, 100), 217 (9), 183 (3), 167 (39), 152 (10), 139 (3), 108 (3), 91 (8), 82 (4), 63 (4), 51 (2). The data are in agreement with those previously reported in the literature. [14,16]

Two-step conversion of carboxylic acids to trifluoromethyl arenes

General method:

Step One: Carboxylic acid (0.4 mmol, 1 equiv.), $(Me_4N)SCF_3$ (77 mg, 0.44 mmol, 1.1 equiv.) and dichloromethane (2 mL) were added to a 10 mL sample tube under an argon atmosphere. The tube was sealed and the reaction mixture was stirred at room temperature for 2 hours. Hexane (1 mL) was then added to the reaction mixture, and the mixture filtered through a pad of celite (3-4 cm in a Pasteur pipette) and the solvent removed under reduced pressure. The obtained material was further purified as outlined below, before being taken on to the second step.

Step Two: $[(cinnamyl)PdCl]_2$ (8 mg, 0.016 mmol, 0.04 equiv.), Xantphos (28 mg, 0.048 mmol, 0.12 equiv.) and potassium phosphate (17 mg, 0.08 mmol, 0.2 equiv.) were added to a 20 mL pressure tube under an argon atmosphere. The acyl fluoride was then transferred to the pressure tube by rinsing the vial containing the compound first with 1 mL of toluene and then 0.2 mL toluene (to ensure that all of the acyl fluoride was transferred to the pressure tube). Triethyl(trifluoromethyl)silane (160 µL, 0.8 mmol, 2 equiv.) was then added, the tube was sealed and the reaction mixture was heated with vigorous stirring at 170 °C for 16 hours. The reaction mixture was then allowed to cool to room temperature, 4-(trifluoromethoxy)anisole (60 µL, 0.4 mmol, 1 equiv.) was added as an internal standard and the mixture filtered through a pad of celite (3-4 cm in a Pasteur pipette). The crude reaction mixture was then analyzed using GC-MS and ^{19}F NMR spectroscopic analysis.

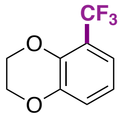
2c

5-(Trifluoromethyl)-2,3-dihydrobenzo[*b*][1,4]dioxine 2c:

Step One: The crude material was filtered through a pad of silica (3-4 cm in a Pasteur pipette) using 20% ethyl acetate in hexane (10-15 mL) to remove the coloured impurities, and the solvent removed under reduced pressure. Step Two: The title compound was obtained after purification using flash column chromatography on silica gel using 5% ethyl acetate in hexane (R_f = 0.33) in 35% yield (28.5 mg) as a colourless oil. Characterization data is the same as that reported on page 9.

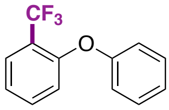
2e

1-Phenoxy-2-(trifluoromethyl)benzene 2e:

Step One: The crude material was purified using flash column chromatography on silica gel using 5% ethyl acetate in hexane (R_f = 0.42) to give a colourless oil. Step Two: The title compound was obtained after purification using flash column chromatography on silica gel using hexane (R_f = 0.29) in 55% yield (52.3 mg) as a colourless oil. Characterization data is the same as that reported on page 9.

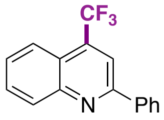

2l

2-Phenyl-4-(trifluoromethyl)quinoline 2l:

Step One: The crude material was filtered through a pad of silica (3-4 cm in a Pasteur pipette) using 10% ethyl acetate in hexane (10-15 mL) to remove the coloured impurities, and the solvent removed under reduced pressure. Step Two: The reaction was performed at 180 °C for 16 hours, not 170 °C. The title compound was obtained after purification using flash column chromatography on silica gel using 5% ethyl acetate in hexane (R_f = 0.17) in 66% yield (72.1 mg) as a pale yellow solid. Characterization data is the same as that reported on page 11.

Computational details

All calculations were performed using the Gaussian 09 software.[17] The frequency calculations and structure optimizations were performed using ωB97XD with the 6-31G(d) basis set for C, O, H, F and P, and the SDD ECP for Pd and Si. These calculations were performed at both 25 °C and 160 °C. Single point energy calculations were performed using both the M06 and M06L methods, and the def2TZVP basis set. The CPCM solvation model was used to incorporate the solvent effects of toluene. Frequency calculations were performed to confirm whether calculated structure is a minimum or a transition state. For transition state structures, IRC calculations were performed to confirm whether the located transition state connects the correct minima. The barriers calculated using the M06 and M06L methods showed similar trends, as such only the results for the calculations using the M06L method are shown below. The (cinnamyl)Pd(Xantphos) complex was used as the pre-catalyst (the $Pd^{(0)}$ species prior to oxidative addition of acyl fluoride) for the calculations.[18]

Summary of calculated barriers at 25 °C and 160 °C

Table S1: Summary of the barriers for each key step in the catalytic cycle at 25 °C.

**Barrier**	**Substrate**	
	Phenyl acyl fluoride	[1,1'-Biphenyl]-4-carbonyl fluoride **1a**
Oxidative addition	10.8	11.6
Decarbonylation from *ArCO-[Pd$^{(II)}$]-F*	25.1	24.8
Decarbonylation from *ArCO-[Pd$^{(II)}$]-CF$_3$*	18.1	13.8
Reductive elimination from *ArCO-[Pd$^{(II)}$]-CF$_3$*	16.4	14.8
Reductive elimination from *Ar-[Pd$^{(II)}$]-CF$_3$*	24.1	23.9

Table S2: Summary of the barriers for each key step in the catalytic cycle at 160 °C.

**Barrier**	**Substrate**	
	Phenyl acyl fluoride	[1,1'-Biphenyl]-4-carbonyl fluoride **1a**
Oxidative addition	10.1	11.4
Decarbonylation from *ArCO-[Pd$^{(II)}$]-F*	27.3	23.5
Decarbonylation from *ArCO-[Pd$^{(II)}$]-CF$_3$*	17.4	13.0
Reductive elimination from *ArCO-[Pd$^{(II)}$]-CF$_3$*	16.5	14.5
Reductive elimination from *Ar-[Pd$^{(II)}$]-CF$_3$*	24.6	24.6

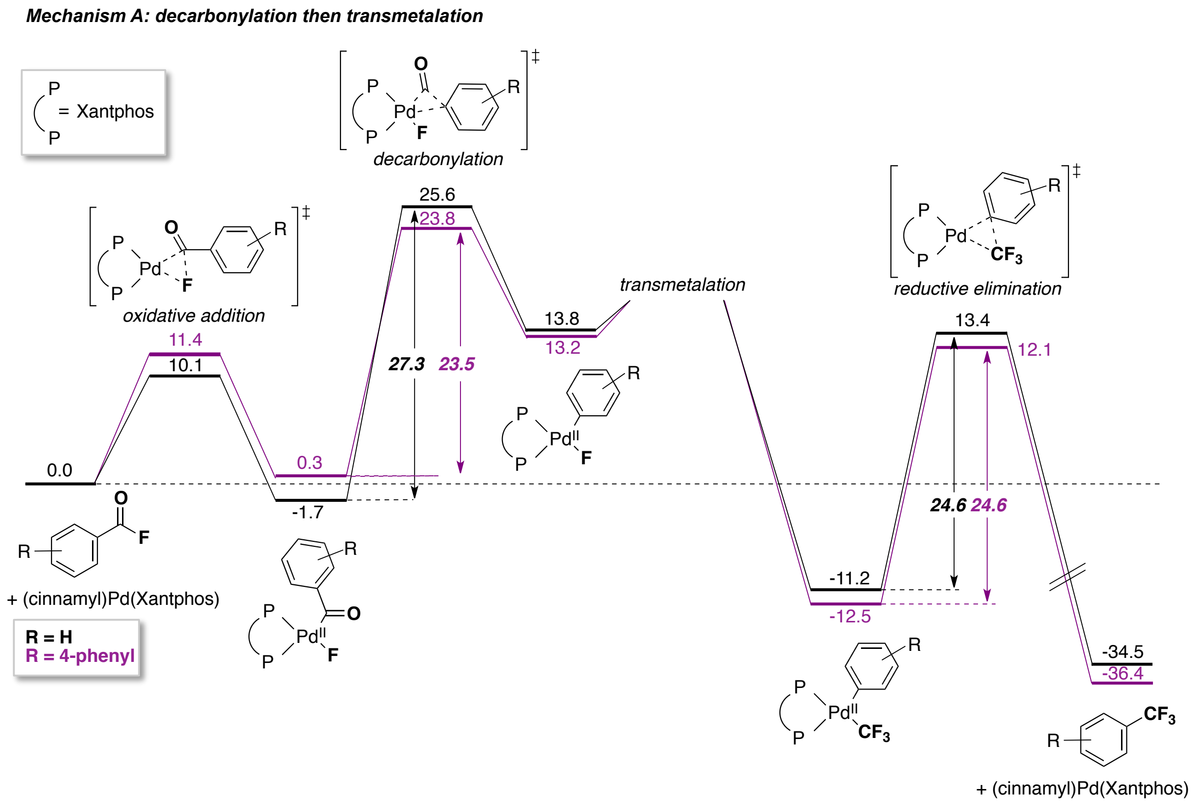

Figure S1: The calculated barriers for Mechanism A for phenyl acyl fluoride (black) and [1,1'-biphenyl]-4-carbonyl fluoride **1a** (pink) at 160°C.

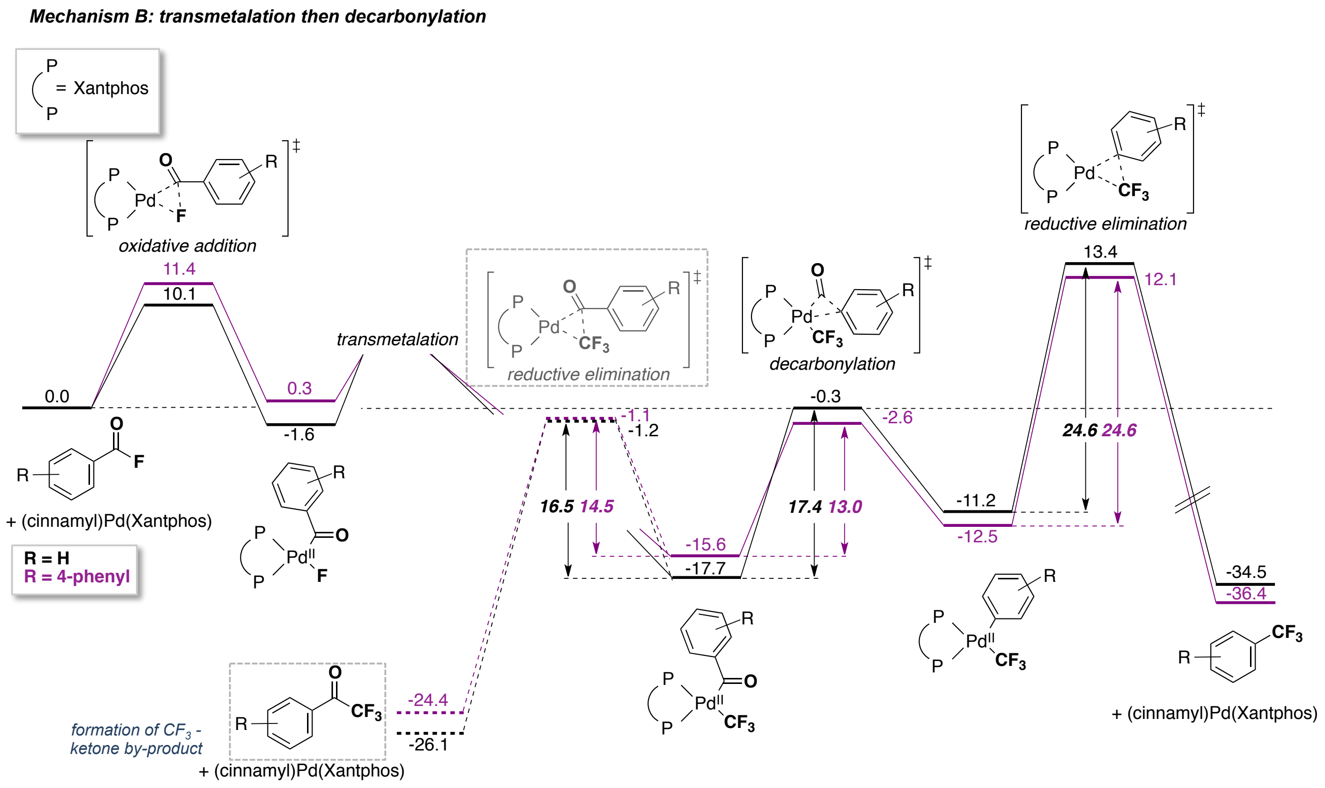

Figure S2: The calculated barriers for Mechanism B for phenyl acyl fluoride (black) and [1,1'-biphenyl]-4-carbonyl fluoride **1a** (pink) at 160°C.

Distortion/Interaction Analysis

For each transition state an IRC calculation was run at 160 °C using the ωB97XD method, with the 6-31G(d) basis set for C, O, H, F and P and the SDD ECP for Pd. For each point along the calculated IRC single point energy calculations were performed using the M06L method and the def2TZVP basis set, and the CPCM solvation model was used to incorporate the solvent effects of toluene. For each point along the IRC, single points energy calculations were performed for: 1) the whole structure; 2) just the Xantphos fragment and 3) just the PhCO-Pd-X (X = F or CF_3) fragment (see Figure S3 for an example of the structures). These energies were compared with the energy of the corresponding intermediate (either PhCO-[$Pd^{(II)}$]-F or PhCO-[$Pd^{(II)}$]-CF_3) to determine the interaction and distortion energies using the equations shown below. See the cited paper for a more detailed discussion on the use of distortion/interaction analysis.[19]

$$\textit{Potential energy surface } (\Delta E) = E\,(\text{IRC point}) - E\,(\text{Intermediate})$$
$$\Delta E(\text{distortion}) = [E(\text{fragment 1}) + E(\text{fragment 2})] - E\,(\text{Intermediate})$$
$$\Delta E = \Delta E(\text{distortion}) + \Delta E(\text{interaction})$$

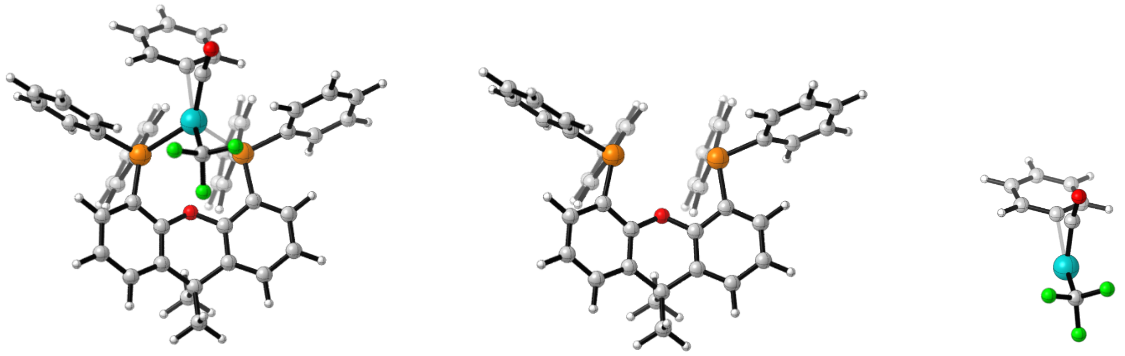

Figure S3: Examples of the structures used in the single point energy calculations: the 'whole structure' (left), the Xantphos fragment (middle) and the PhCO-Pd-X (in this example, X = CF_3) fragment (right).

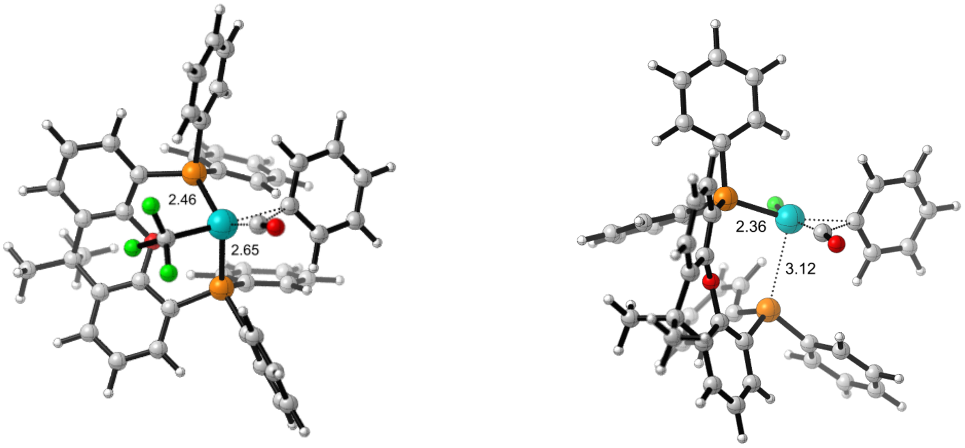

Figure S4: The transition states for decarbonylation from: PhCO-[$Pd^{(II)}$]-CF_3 (left) and PhCO-[$Pd^{(II)}$]-F (right).

Table S3: Single point energies used for the distortion/interaction analysis for decarbonylation from PhCO-[Pd$^{(II)}$]-CF_3, and the ΔE values derived from this data.

IRC Point	E (whole complex)	E (Xantphos fragment)	E (PhCO-Pd-CF_3 fragment)	ΔE	ΔE (distortion)	ΔE(interaction)
0	-3074.55977449	-2263.71795441	-810.76680420	21.48860	68.56182	-47.07322
-0.60152	-3074.56073735	-2263.71797045	-810.77052999	20.88440	66.21378	-45.32938
-1.20298	-3074.56253747	-2263.71798867	-810.77509182	19.75481	63.33976	-43.58495
-1.80430	-3074.56486166	-2263.71800475	-810.77977117	18.29635	60.39333	-42.09698
-2.40540	-3074.56719434	-2263.71801962	-810.78370309	16.83257	57.91668	-41.08411
0.60151	-3074.55971556	-2263.71793493	-810.76417997	21.52558	70.22078	-48.69519
1.20302	-3074.56053793	-2263.71790735	-810.76269310	21.00954	71.17110	-50.16157
1.80449	-3074.56198141	-2263.71787427	-810.76224374	20.10374	71.47384	-51.37010
2.40594	-3074.56364806	-2263.71782526	-810.76257648	19.05790	71.29580	-52.23790
PhCO-[Pd$^{(II)}$]-CF_3 Intermediate	-3074.59401873					

Table S4: Single point energies used for the distortion/interaction analysis for decarbonylation from PhCO-[Pd$^{(II)}$]-F via the 'mono-ligated' transition state, and the ΔE values derived from this data.

IRC Point	E (whole complex)	E (Xantphos fragment)	E (PhCO-Pd-F fragment)	ΔE	ΔE (distortion)	ΔE(interaction)
0	-2836.71481640	-2263.71446147	-572.93009999	29.21401	73.29971	-44.08570
-0.57827	-2836.71563182	-2263.71442457	-572.92795938	28.70235	74.66611	-45.96377
-1.16575	-2836.71730480	-2263.71443557	-572.92654704	27.65253	75.54547	-47.89294
-1.75313	-2836.71944992	-2263.71443826	-572.92607283	26.30645	75.84135	-49.53490
-2.34021	-2836.72159009	-2263.71443108	-572.92616995	24.96347	75.78492	-50.82145
0.58448	-2836.71488479	-2263.71439943	-572.93350677	29.17111	71.20085	-42.02973
1.17198	-2836.71586156	-2263.71438490	-572.93768174	28.55818	68.59013	-40.03195
1.75946	-2836.71745801	-2263.71437481	-572.94194197	27.55639	65.92313	-38.36674
2.34698	-2836.71935138	-2263.71437551	-572.94586164	26.36828	63.46306	-37.09477
PhCO-[Pd$^{(II)}$]-F Intermediate	-2836.76137188					

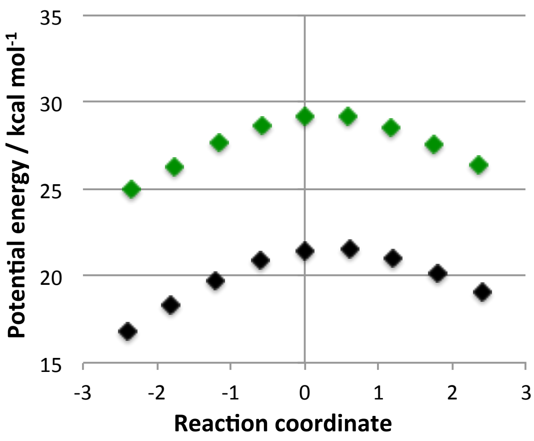

Figure S5: Plot of the potential energy (ΔE) along the reaction coordinate (derived from the IRC calculations) for decarbonylation from PhCO-[Pd$^{(II)}$]-CF_3 (black) and PhCO-[Pd$^{(II)}$]-F (green).

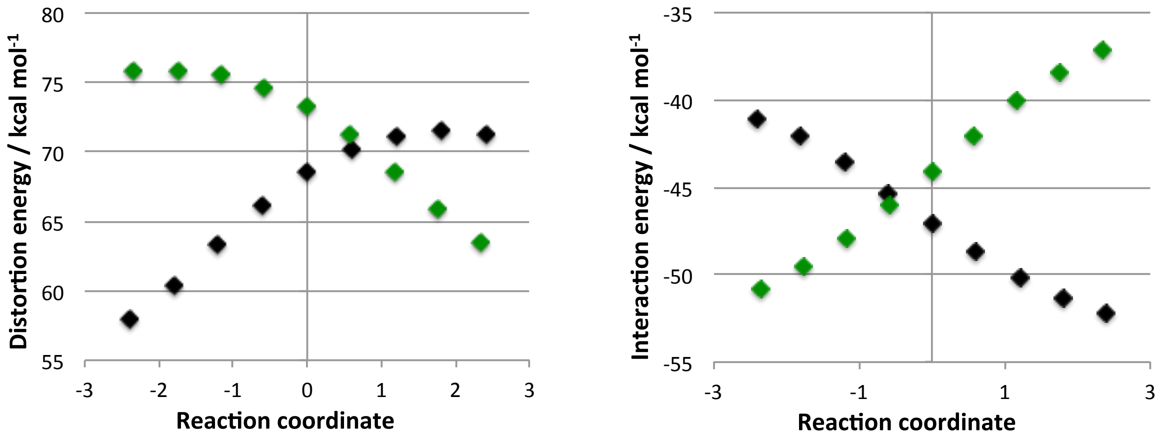

Figure S6: Plots of the distortion energy (left) and interaction energy (right) along the reaction coordinate (derived from the IRC calculations) for decarbonylation from PhCO-[Pd$^{(II)}$]-CF_3 (black) and PhCO-[Pd$^{(II)}$]-F (green).

Reaction development and control reactions

Examination of different additives and CF_3 sources

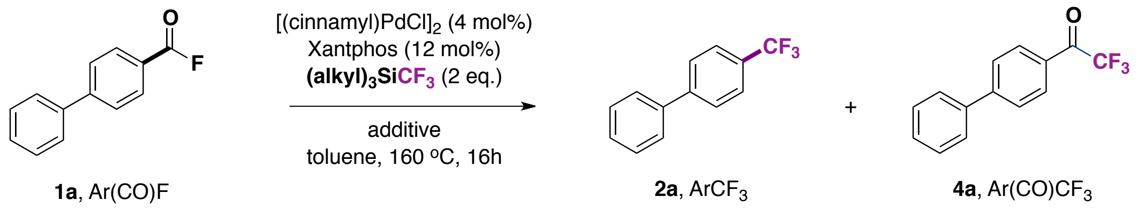

Reaction conditions: [1,1'-biphenyl]-4-carbonyl fluoride **1a** (20 mg, 0.1 mmol), $[(cinnamyl)PdCl]_2$ (2 mg, 0.004 mmol), Xantphos (7 mg, 0.012 mmol), additive (as indicated below), $(alkyl)_3SiCF_3$ (0.2 mmol) in toluene (0.3 mL) was heated at 160 °C in a 5 mL pressure tube for 16 hours. The crude reaction mixture was analyzed using both ^{19}F NMR spectroscopy and GC-MS. The extent of conversion was determined by ^{19}F NMR spectroscopic analysis of the crude reaction mixture, using 4-(trifluoromethoxy)anisole (15 µL, 0.1 mmol) as an internal standard.

**CF_3 source**	**Additive**	**Result (extents of conversion)**
$(Me)_3SiCF_3$	-	15% $ArCF_3$ **2a**, 30% $Ar(CO)CF_3$ **4a**, 6% Ar(CO)F **1a**
$(Me)_3SiCF_3$	K_3PO_4 (1 eq.)	13% $ArCF_3$ **2a**, 4% $Ar(CO)CF_3$ **4a**, no Ar(CO)F **1a**
$(Me)_3SiCF_3$	K_3PO_4 (0.2 eq.)	31% $ArCF_3$ **2a**, 5% $Ar(CO)CF_3$ **4a**, no Ar(CO)F **1a**
$(Et)_3SiCF_3$	K_3PO_4 (0.2 eq.)	43% $ArCF_3$ **2a**, 4% $Ar(CO)CF_3$ **4a**, no Ar(CO)F **1a**
$(Et)_3SiCF_3$	K_3PO_4 (0.05 eq.)	35% $ArCF_3$ **2a**, 15% $Ar(CO)CF_3$ **4a**, 15% Ar(CO)F **1a**

Test reactions using $Ar(CO)CF_3$

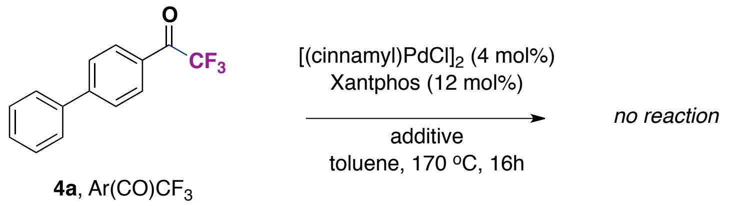

Reaction conditions: 1-([1,1'-biphenyl]-4-yl)-2,2,2-trifluoroethanone **4a** (25 mg, 0.1 mmol), $[(cinnamyl)PdCl]_2$ (2 mg, 0.004 mmol), Xantphos (7 mg, 0.012 mmol), additive (see below) in toluene (0.3 mL) was heated at 170 °C in a 5 mL pressure tube for 16 hours. The crude reaction mixture was analyzed using both ^{19}F NMR spectroscopy and GC-MS.

**Additive**	**Result**
-	Unreacted $Ar(CO)CF_3$ **4a**
K_3PO_4 (0.2 eq.)	Unreacted $Ar(CO)CF_3$ **4a**
K_3PO_4 (0.2 eq.) and $(Et)_3SiCF_3$ (2 eq.)	Unreacted $Ar(CO)CF_3$ **4a**

Examination of different temperatures

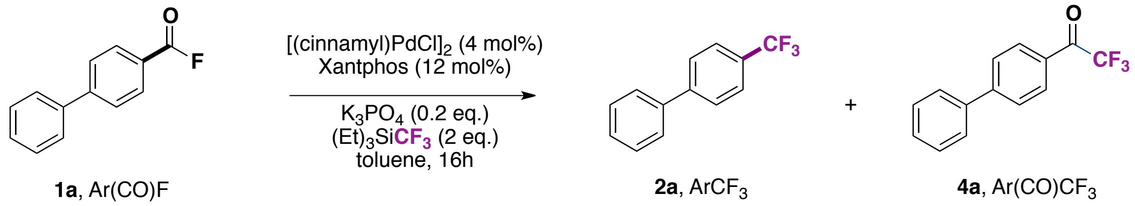

Reaction conditions: [1,1'-biphenyl]-4-carbonyl fluoride **1a** (20 mg, 0.1 mmol), $[(cinnamyl)PdCl]_2$ (2 mg, 0.004 mmol), Xantphos (7 mg, 0.012 mmol), K_3PO_4 (4 mg, 0.02 mmol), $(Et)_3SiCF_3$ (40 µL, 0.2 mmol) in toluene (0.3 mL) were heated at the specified temperature in a 5 mL pressure tube for 16 hours. The crude reaction mixture was analyzed using both ^{19}F NMR spectroscopy and GC-MS. The extent of conversion was determined by ^{19}F NMR spectroscopic analysis of the crude reaction mixture, using 4-(trifluoromethoxy)anisole (15 µL, 0.1 mmol) as an internal standard.

**Temperature / °C**	**Result (extents of conversion)**
145	28% $ArCF_3$ **2a**, 32% $Ar(CO)CF_3$ **4a**
160	43% $ArCF_3$ **2a**, 4% $Ar(CO)CF_3$ **4a**
180	46% $ArCF_3$ **2a**, 0% $Ar(CO)CF_3$ **4a**

Test reactions using *bromo* and *chloro* containing substrates

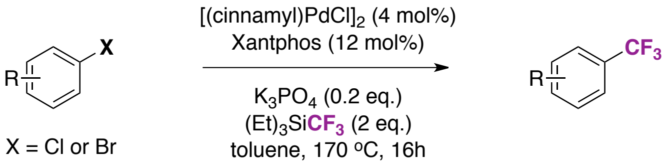

Reaction conditions: ArX (0.1 mmol), $[(cinnamyl)PdCl]_2$ (2 mg, 0.004 mmol), Xantphos (7 mg, 0.012 mmol), K_3PO_4 (4 mg, 0.02 mmol), $(Et)_3SiCF_3$ (40 µL, 0.2 mmol) in toluene (0.3 mL) were heated at 170 °C in a 5 mL pressure tube for 16 hours. The crude reaction mixture was analyzed using both ^{19}F NMR spectroscopy and GC-MS. The extent of conversion was determined by ^{19}F NMR spectroscopic analysis of the crude reaction mixture, using 4-(trifluoromethoxy)anisole (15 µL, 0.1 mmol) as an internal standard.

**Substrate**	**Result**
4-Chloro-1,1'-biphenyl	No formation of $ArCF_3$, mainly unreacted ArX
4-Bromo-1,1'-biphenyl	No formation of $ArCF_3$, mainly unreacted ArX
2-Chloronaphthalene	No formation of $ArCF_3$, mainly unreacted ArX
2-Bromonaphthalene	No formation of $ArCF_3$, mainly unreacted ArX

Test reactions using the conditions reported by Buchwald *et al*

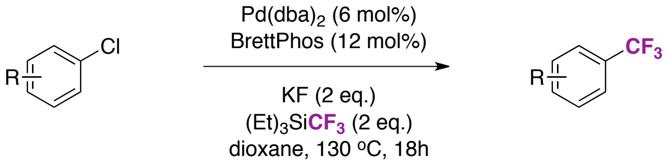

Reaction conditions: ArCl (0.1 mmol), $Pd(dba)_2$ (4 mg, 0.006 mmol), BrettPhos (7 mg, 0.012 mmol), KF (12 mg, 0.2 mmol), $(Et)_3SiCF_3$ (40 µL, 0.2 mmol) in dioxane (0.3 mL) were heated at 130 °C in a 5 mL pressure tube for 18 hours. The crude reaction mixture was analyzed using both ^{19}F NMR spectroscopy and GC-MS. The extent of conversion was determined by ^{19}F NMR spectroscopic analysis of the crude reaction mixture, using 4-(trifluoromethoxy)anisole (15 µL, 0.1 mmol) as an internal standard.

Substrate	Result (extents of conversion)
1-Chloronaphthalene*	75% $ArCF_3$
2-Chloro-1,1'-biphenyl	< 5% $ArCF_3$

* Which is comparable to the reported yield of 84% for this procedure[20]

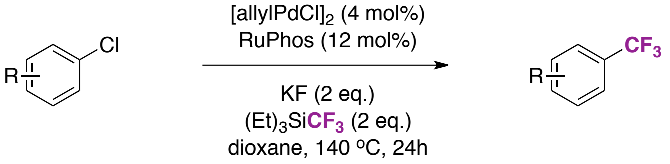

Reaction conditions: ArCl (0.1 mmol), $[allylPdCl]_2$ (2 mg, 0.004 mmol), RuPhos (6 mg, 0.012 mmol), KF (12 mg, 0.2 mmol), $(Et)_3SiCF_3$ (40 µL, 0.2 mmol) in dioxane (0.3 mL) were heated at 140 °C in a 5 mL pressure tube for 24 hours. The crude reaction mixture was analyzed using both ^{19}F NMR spectroscopy and GC-MS. The extent of conversion was determined by ^{19}F NMR spectroscopic analysis of the crude reaction mixture, using 4-(trifluoromethoxy)anisole (15 µL, 0.1 mmol) as an internal standard.

Substrate	Result (extents of conversion)
9-Chloroanthracene*	87% $ArCF_3$
2-Chloro-1,1'-biphenyl	34% $ArCF_3$

* Which is comparable to the reported yield of 90% for this procedure[20]

Control reactions with no catalyst present

Reaction conditions: [1,1'-biphenyl]-4-carbonyl fluoride **1a** (20 mg, 0.1 mmol) and the reagents shown below were heated in toluene (0.3 mL) at 170 °C in a 5 mL pressure tube for 16 hours. The crude reaction mixtures were analyzed using both ^{19}F NMR spectroscopy and GC-MS, using 4-(trifluoromethoxy)anisole (15 µL, 0.1 mmol) as an internal standard.

Reagents/additives present	Result (GC-MS)
K_3PO_4 (0.2 eq.)	Unreacted Ar(CO)F
$(Et)_3SiCF_3$ (2 eq.)	Unreacted Ar(CO)F
K_3PO_4 (0.2 eq.) and $(Et)_3SiCF_3$ (2 eq.)	Complete consumption of Ar(CO)F, no formation of $ArCF_3$ product.*

* Unidentified by-products formed.

Reaction monitoring: formation of $ArCF_3$ over time

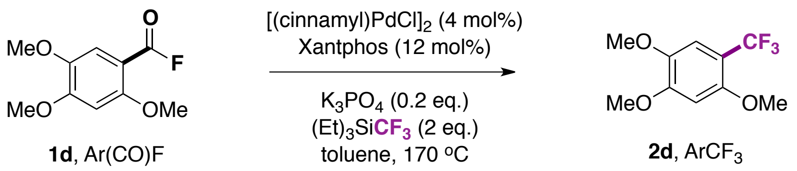

Reaction conditions: A series of 5 mL pressure tubes were prepared containing Ar(CO)F **1d** (21 mg, 0.1 mmol), $[(cinnamyl)PdCl]_2$ (2 mg, 0.004 mmol), Xantphos (7 mg, 0.012 mmol), K_3PO_4 (4 mg, 0.02 mmol), $(Et)_3SiCF_3$ (40 µL, 0.2 mmol) in toluene (0.3 mL). All the tubes were transferred to a heating block pre-warmed to 170 °C and the timer started. The tubes were removed at the time points specified below, cooled in an ice bath to halt the reaction, and acetone (3mL) was added. The extent of conversion to $ArCF_3$ **2d** at different time points was determined by ^{19}F NMR spectroscopic analysis of the crude reaction mixture, using 4-(trifluoromethoxy)anisole (15 µL, 0.1 mmol) as an internal standard.

Time / minutes[a]	Conversion to $ArCF_3$ 2d / %		Average conversion[b] / %
	Repeat 1	Repeat 2	
5	0.25	0.51	0.38 (0.13)
7.5	0.31	0.57	0.44 (0.13)
10	0.41	1.23	0.82 (0.41)
15	1.27	0.76	1.01 (0.26)
20	2.15	2.12	2.14 (0.01)
30	3.29	2.52	2.90 (0.38)
40	5.14	5.44	5.29 (0.15)
80	14.56	10.90	12.73 (1.83)
120	17.56	21.49	19.52 (1.96)
960 (16 h)	61.79	58.43	60.11 (1.68)

[a] As the timer was started as soon as the tubes were added to the heating block there will be a period of time in which the reaction mixture warms to 170 °C; based on when the reaction mixture started vigorously bubbling this is estimated to be between 1-2 minutes. As such, the actual reaction times at 170 °C will be slightly less than that shown here, thus the $ArCF_3$ product **2d** forms in *less than* 5 minutes.
[b] The error shown in parentheses is half the range of the duplicate experiments

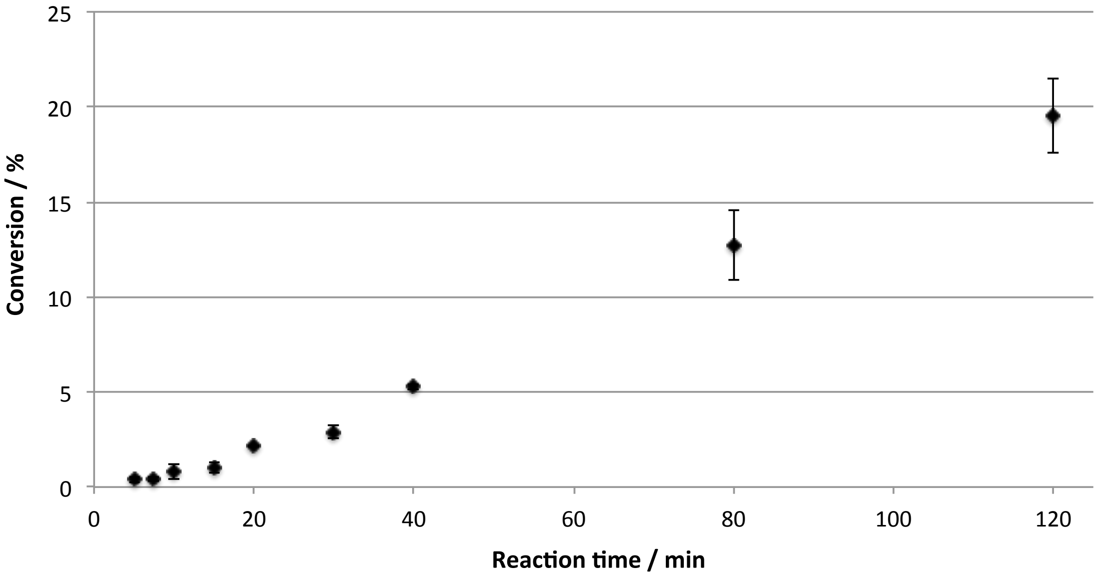

Figure S7: Plot of $ArCF_3$ **2d** formation over time. Error bars represent half the range of duplicate experiments.

NMR Spectra

Acyl fluorides

^{1}**H** (600 MHz, $CDCl_3$)

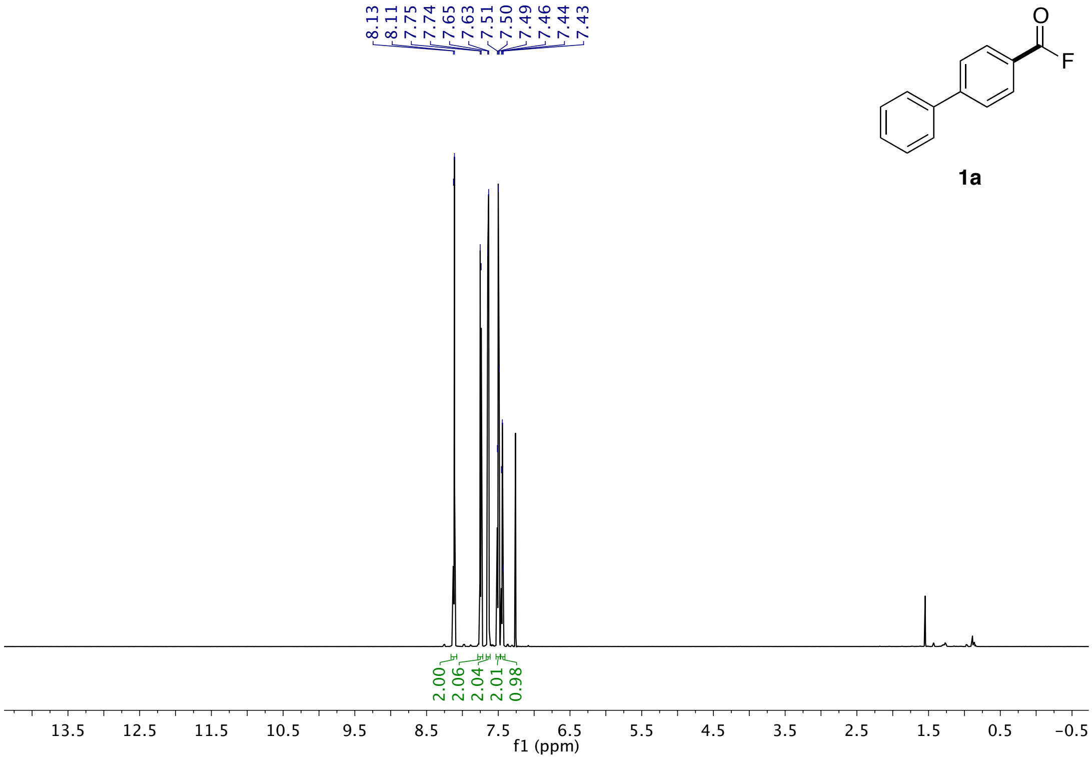

^{19}**F** (564 MHz, $CDCl_3$)

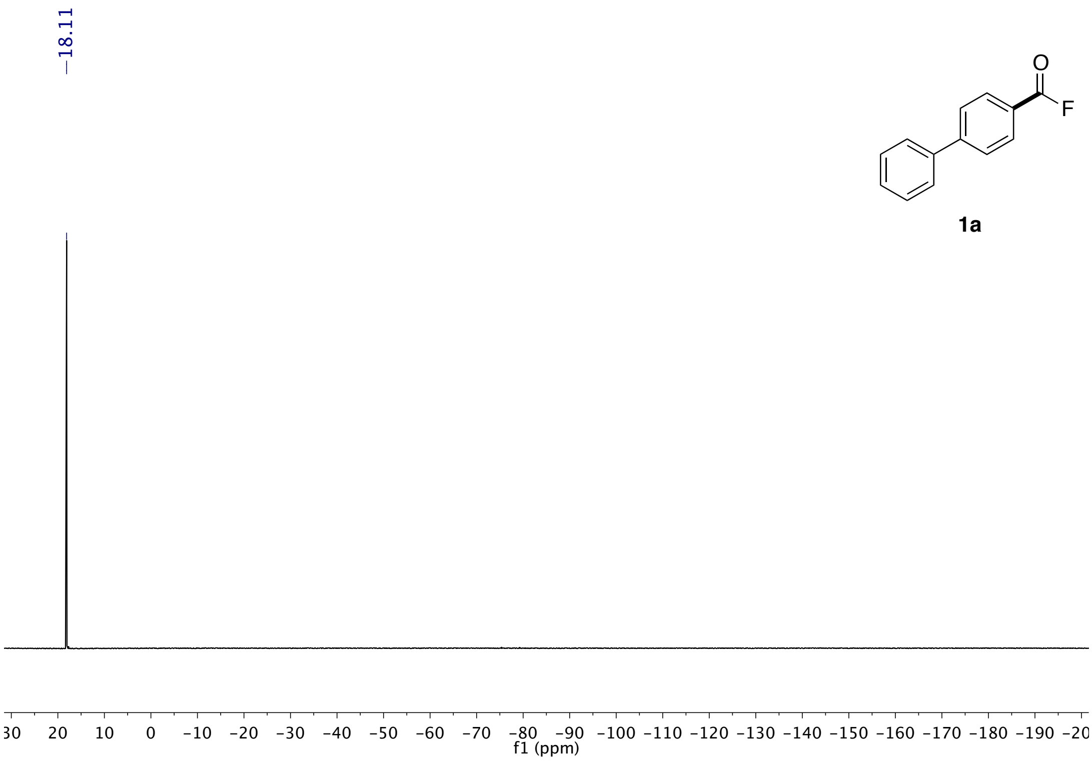

^{13}**C** (151 MHz, $CDCl_3$)

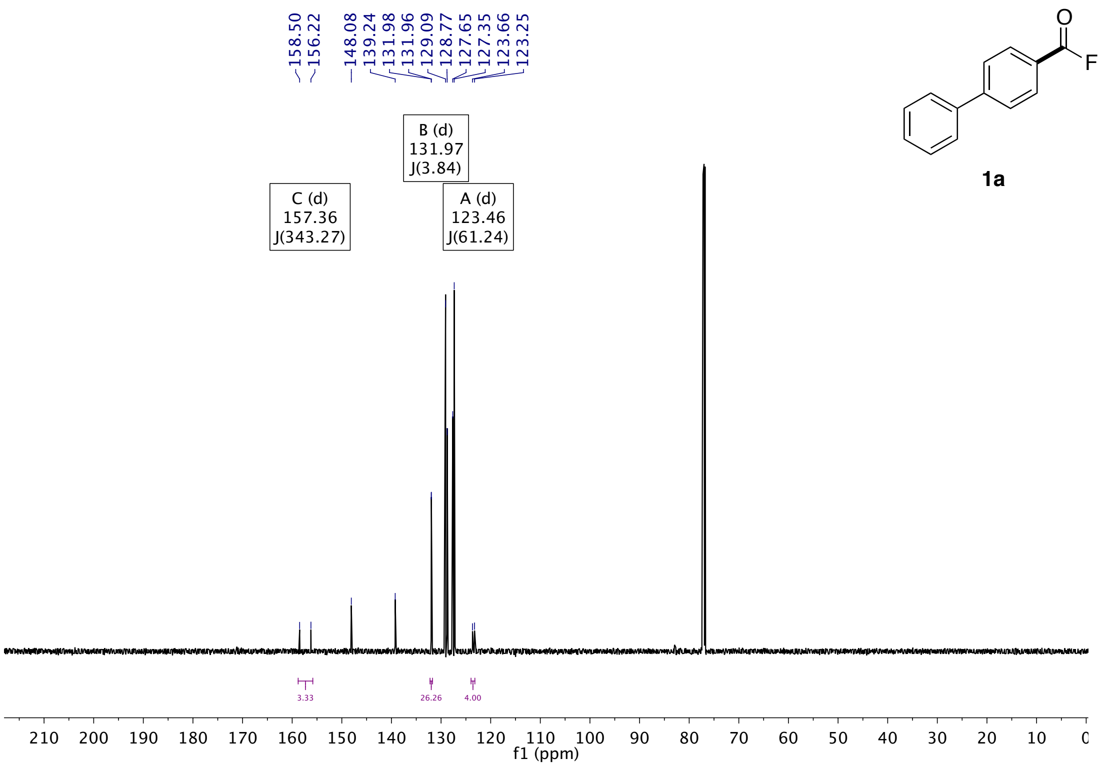

1a

* the peak at 83.3 ppm is an artifact from the spectrometer

^{1}**H** (600 MHz, $CDCl_3$)

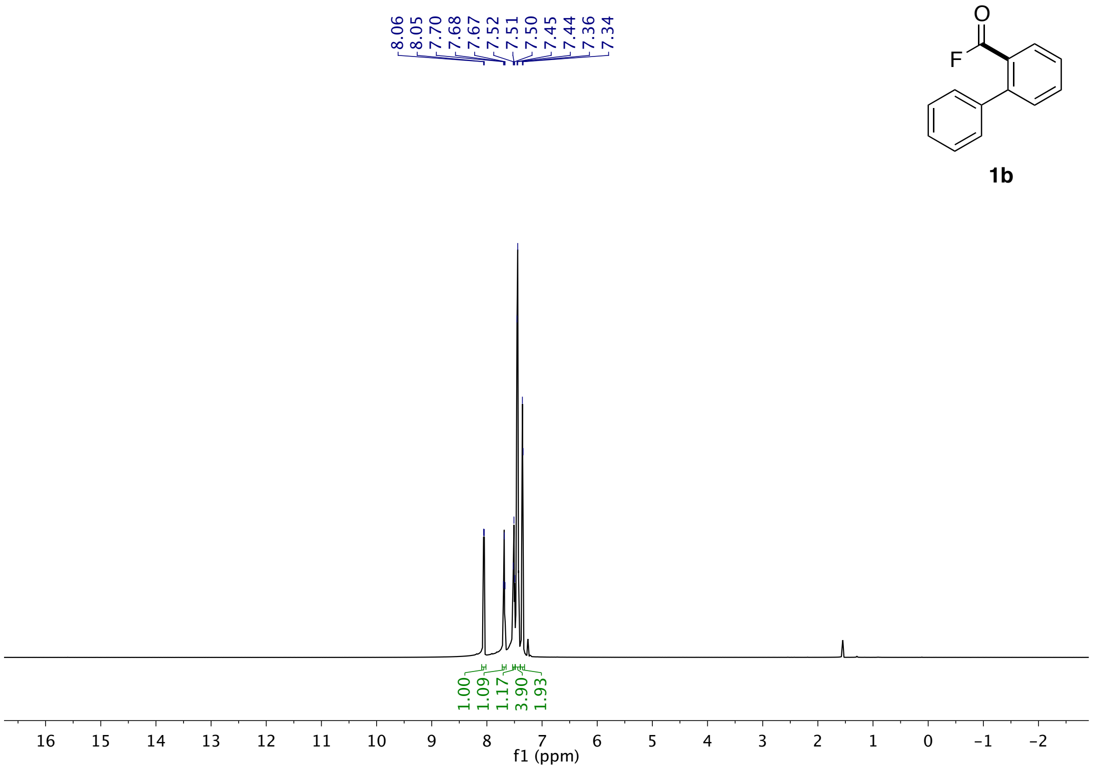

1b

^{19}F (376 MHz, CDCl$_3$)

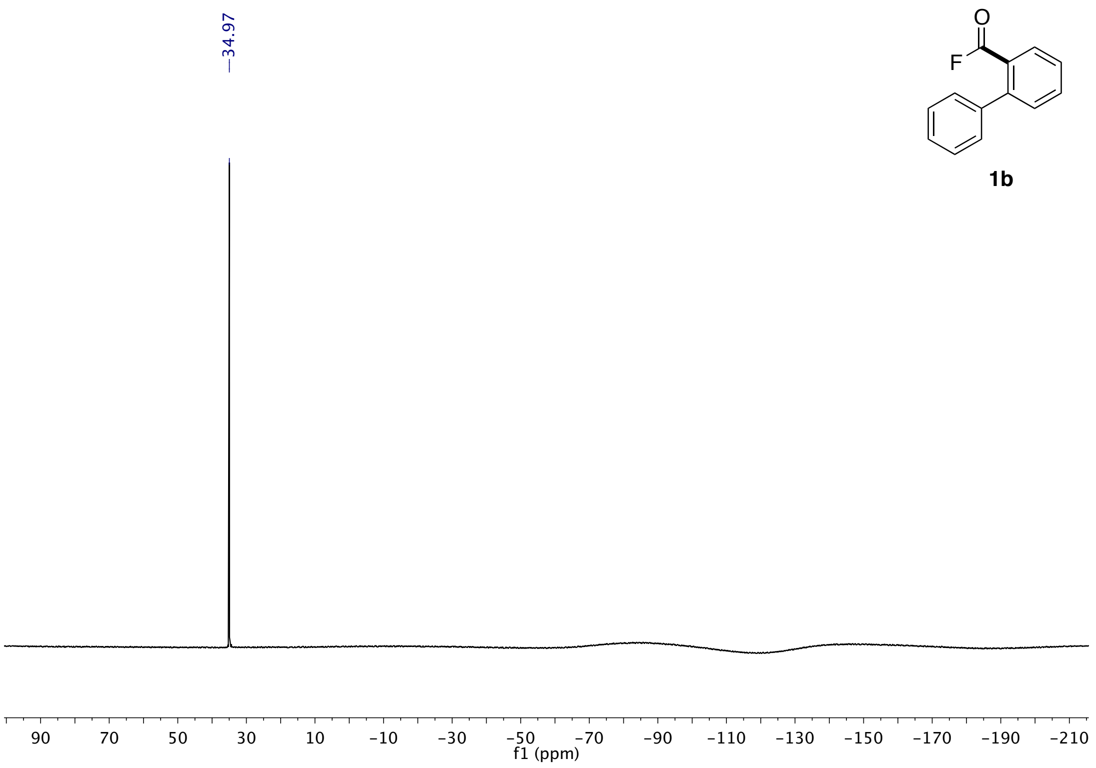

^{13}C (151 MHz, CDCl$_3$)

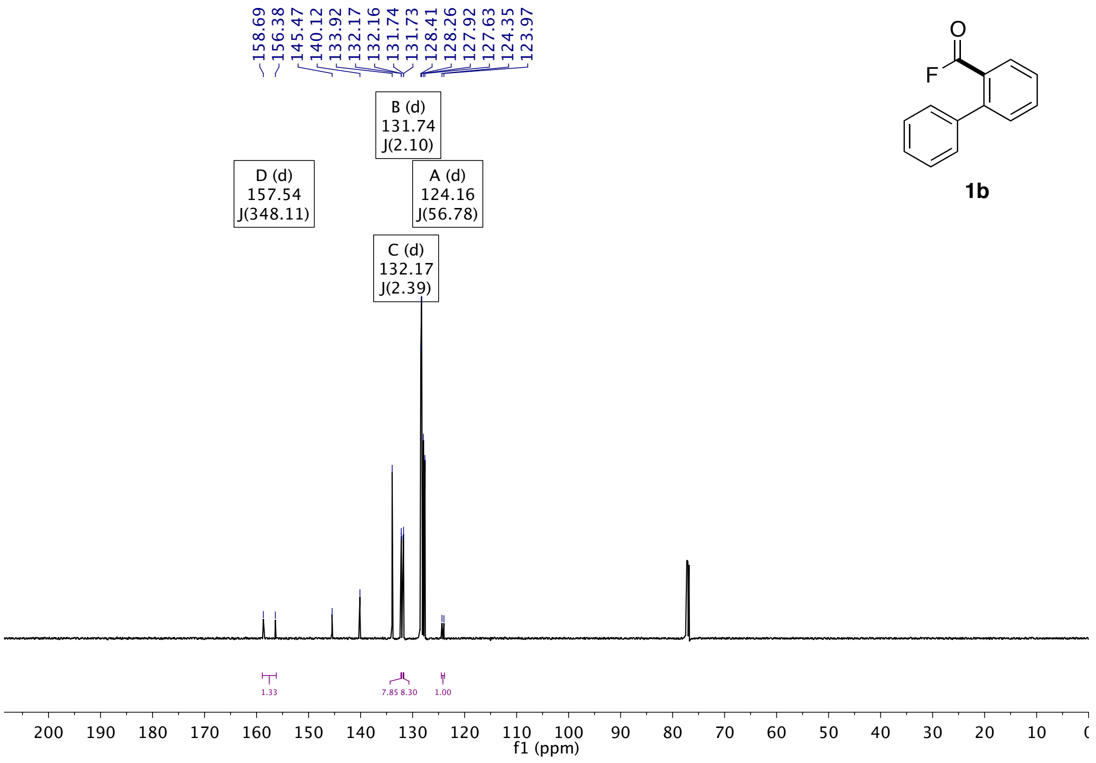

^{1}H (400 MHz, $CDCl_3$)

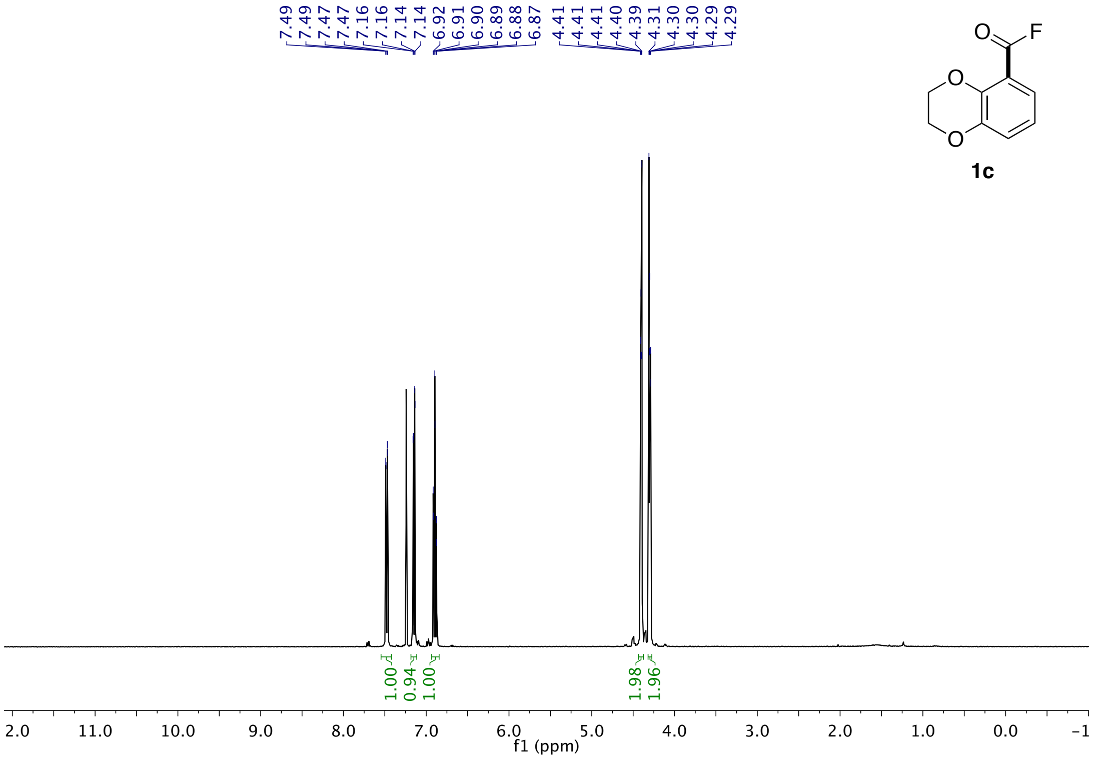

1c

^{19}F (282 MHz, $CDCl_3$)

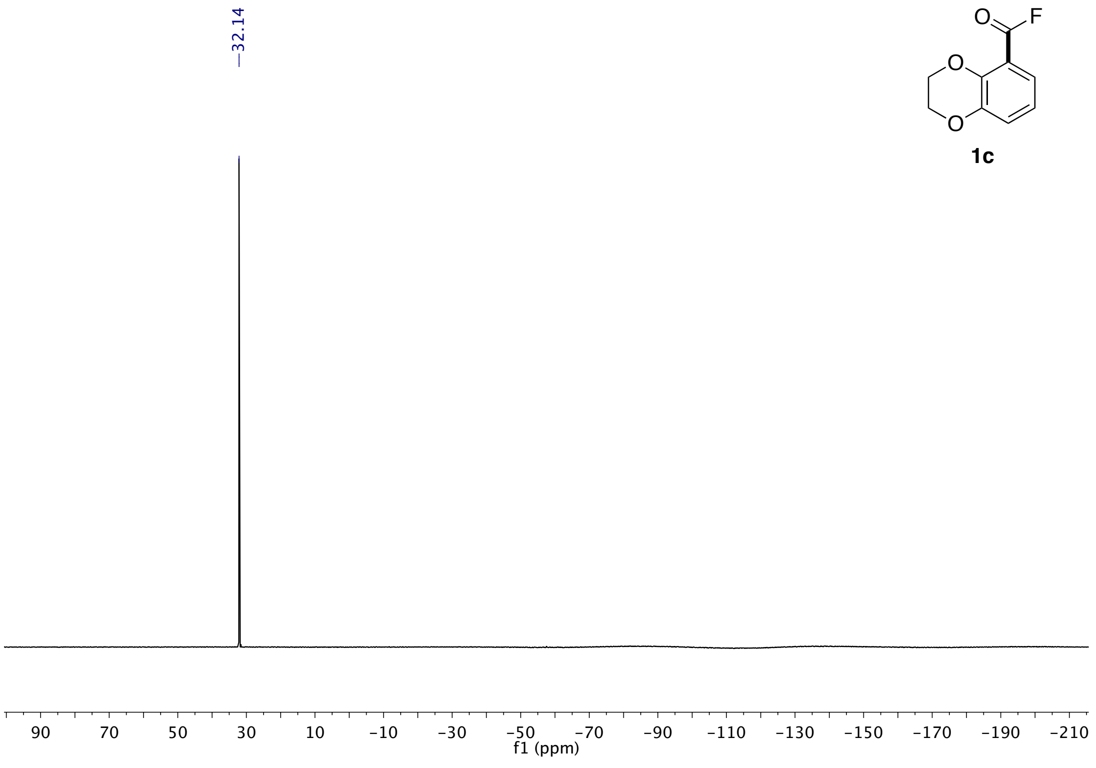

1c

^{13}C (151 MHz, $CDCl_3$)

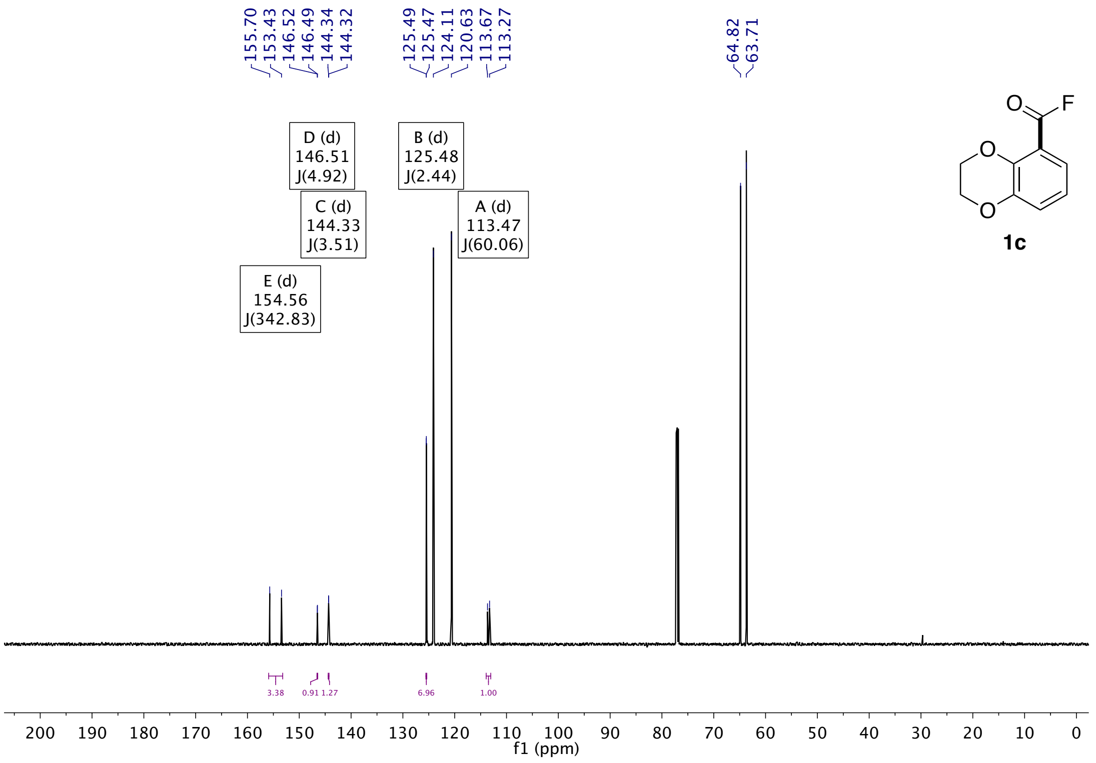

^{1}H (400 MHz, $CDCl_3$)

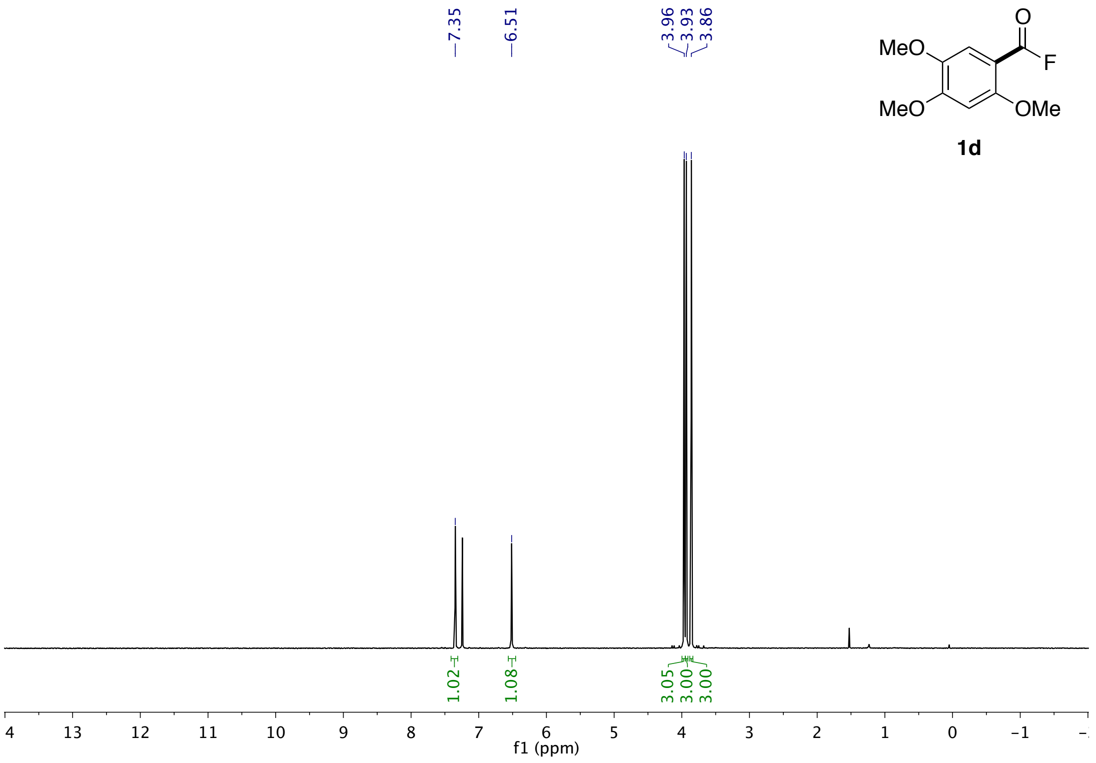

^{19}F (376 MHz, $CDCl_3$)

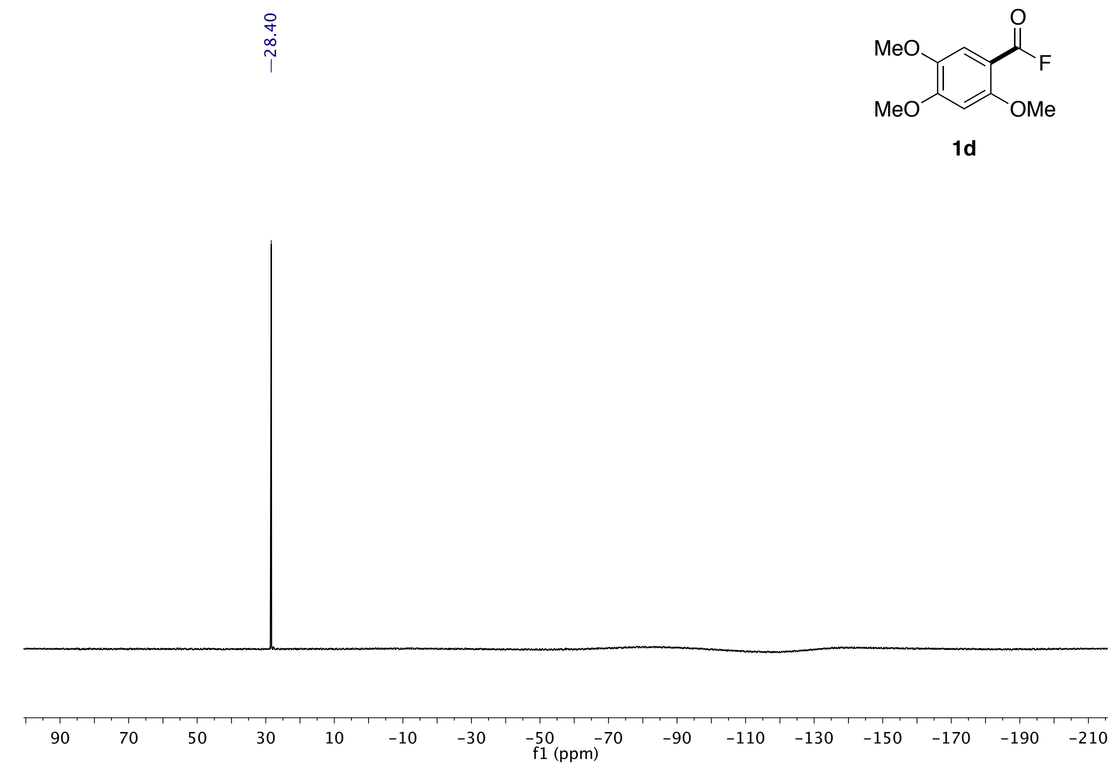

^{13}C (151 MHz, $CDCl_3$)

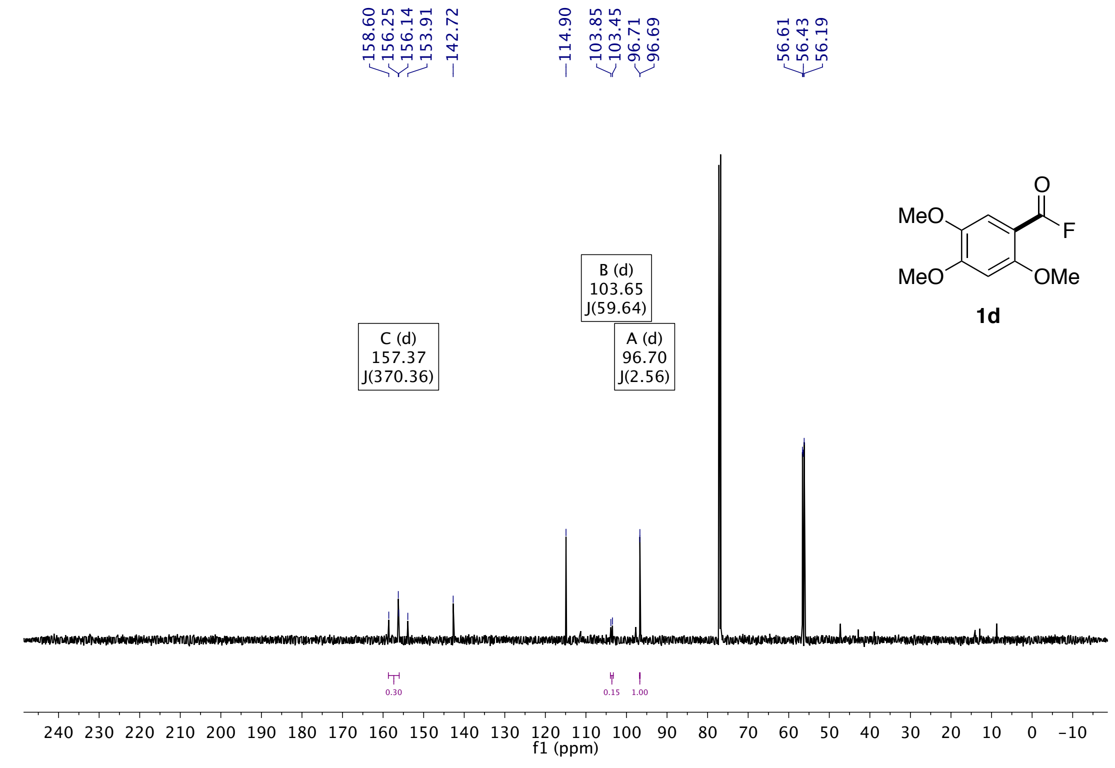

^{1}H (400 MHz, $CDCl_3$)

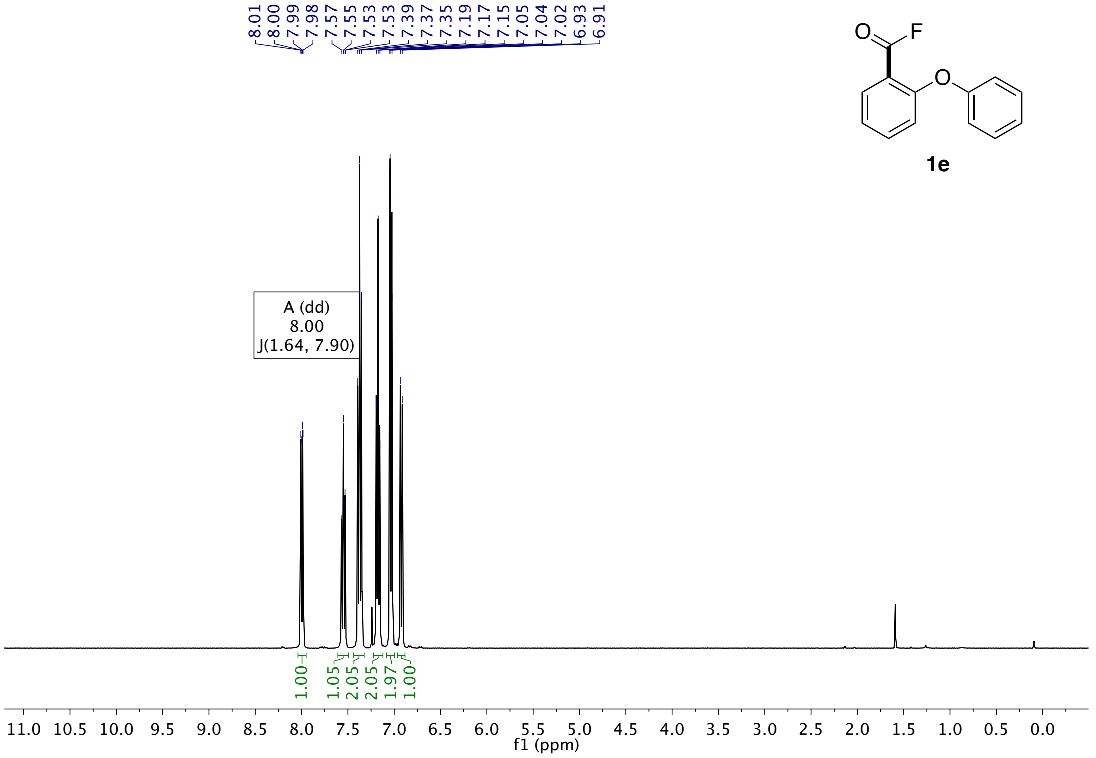

1e

^{19}F (376 MHz, $CDCl_3$)

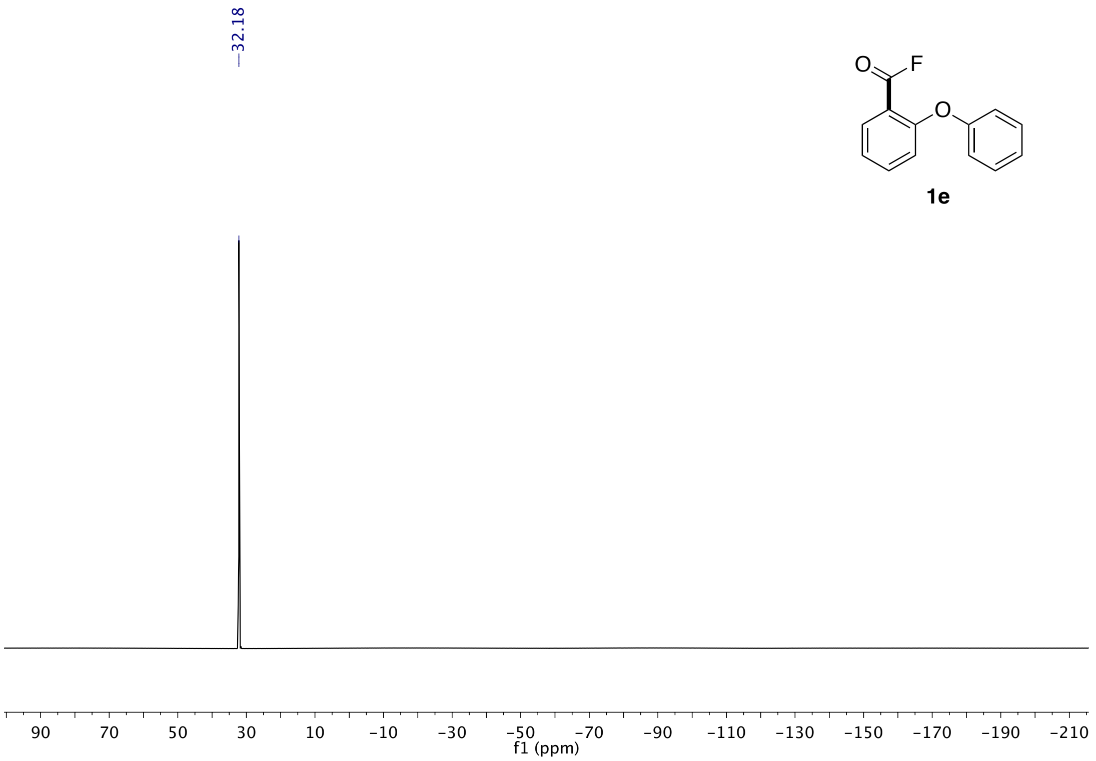

1e

^{13}**C** (101 MHz, $CDCl_3$)

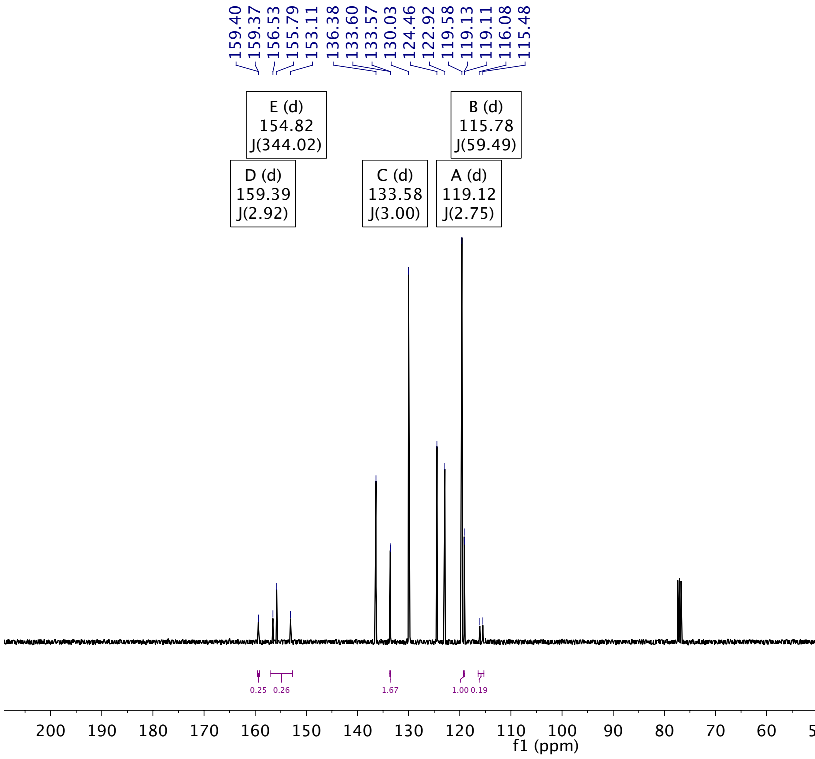

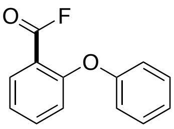

1e

^{1}**H** (400 MHz, $CDCl_3$)

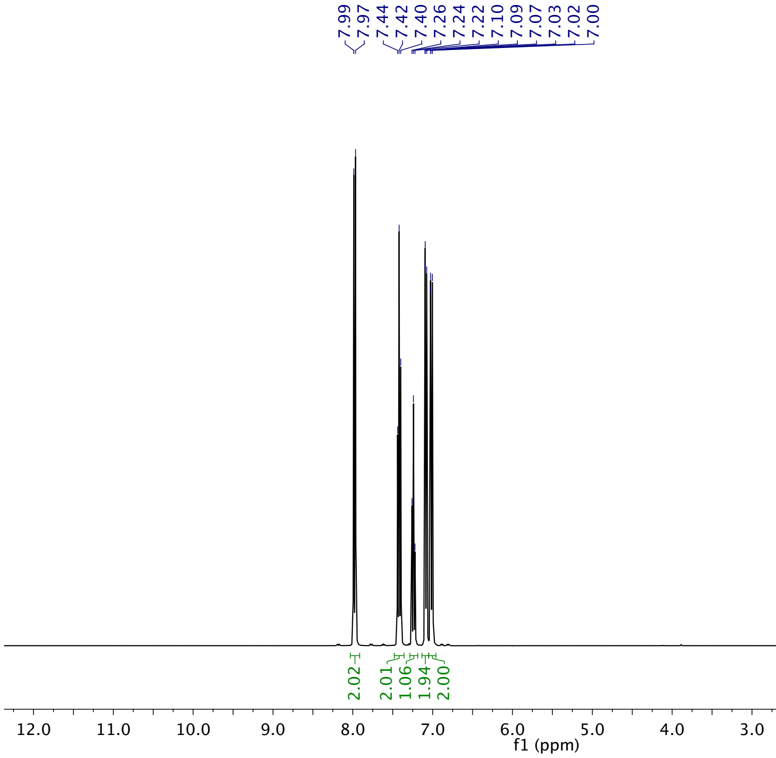

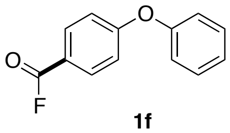

1f

^{19}F (376 MHz, $CDCl_3$)

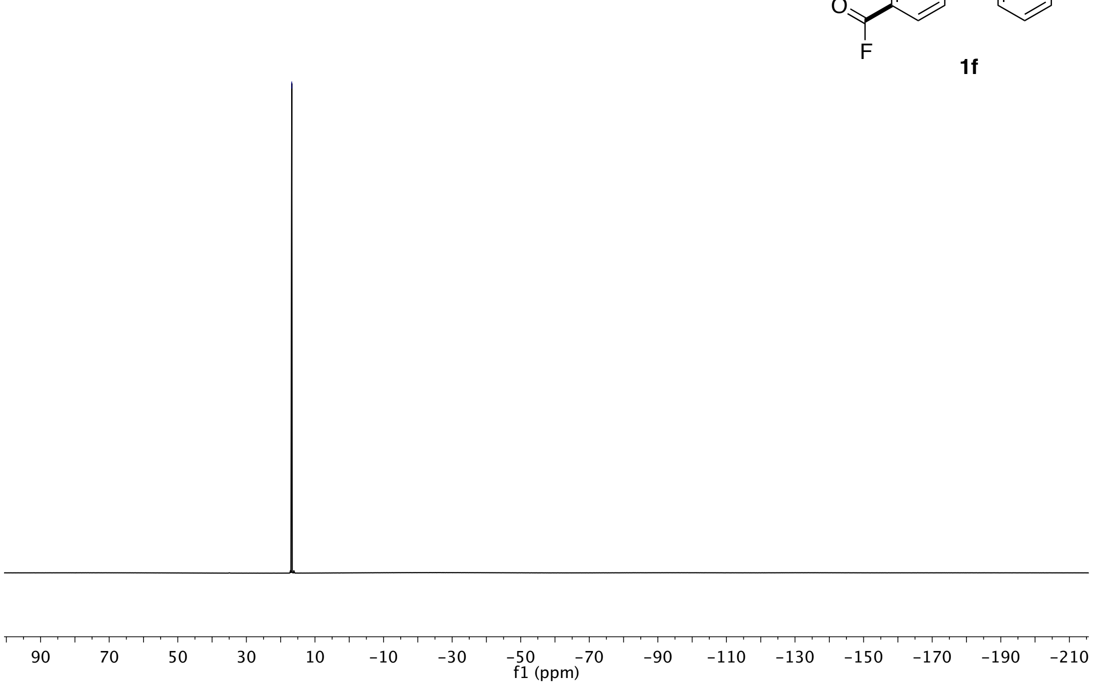

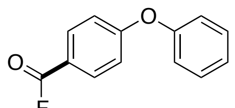

1f

^{13}C (101 MHz, $CDCl_3$)

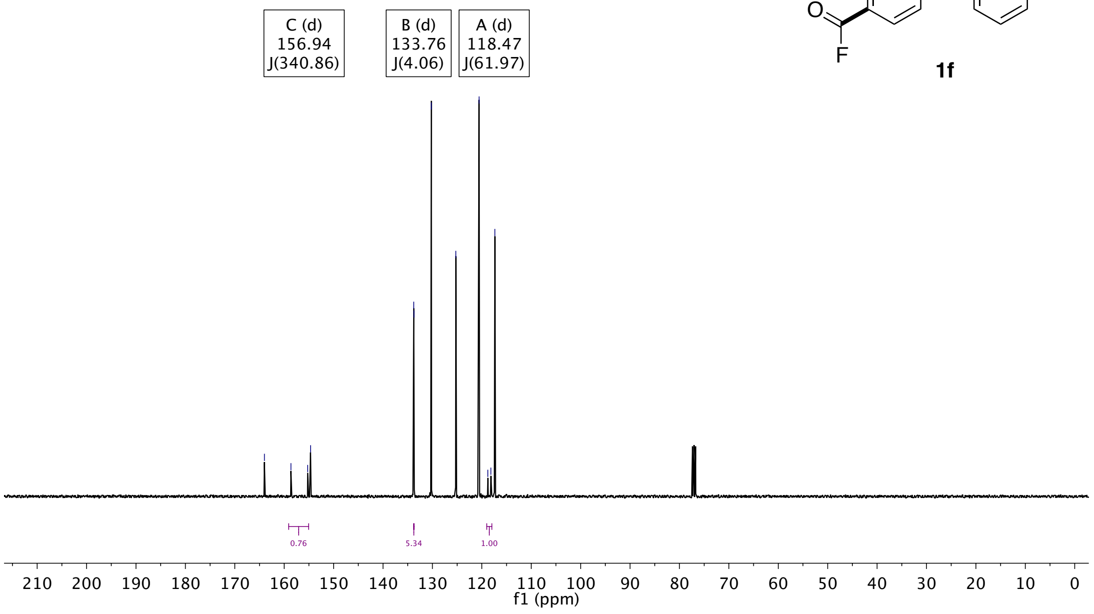

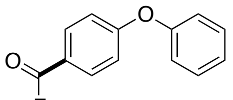

1f

^{1}**H** (400 MHz, $CDCl_3$)

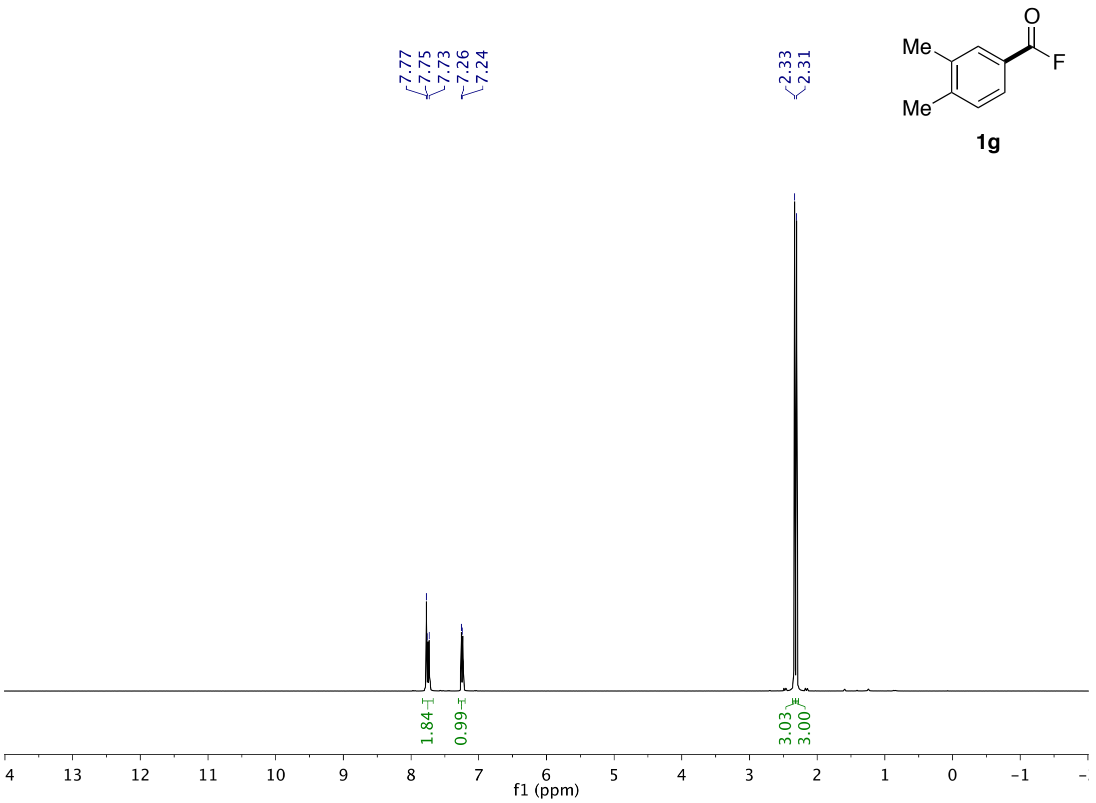

^{19}**F** (376 MHz, $CDCl_3$)

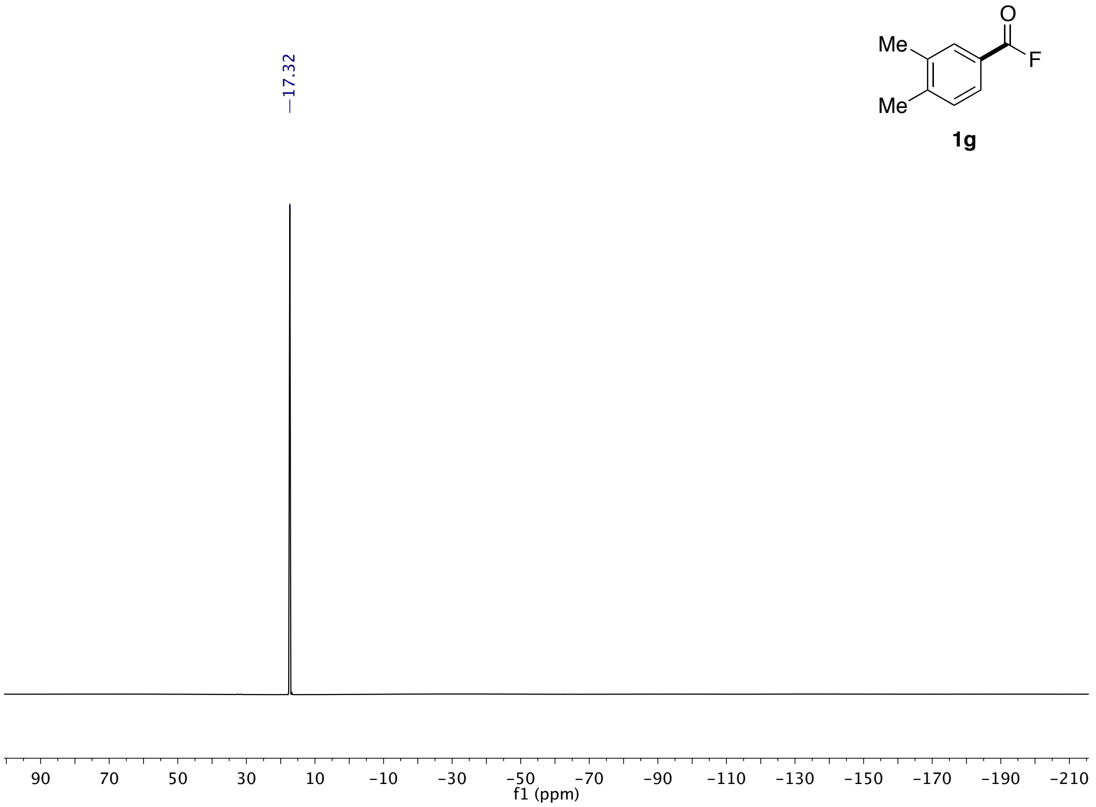

^{13}C (101 MHz, $CDCl_3$)

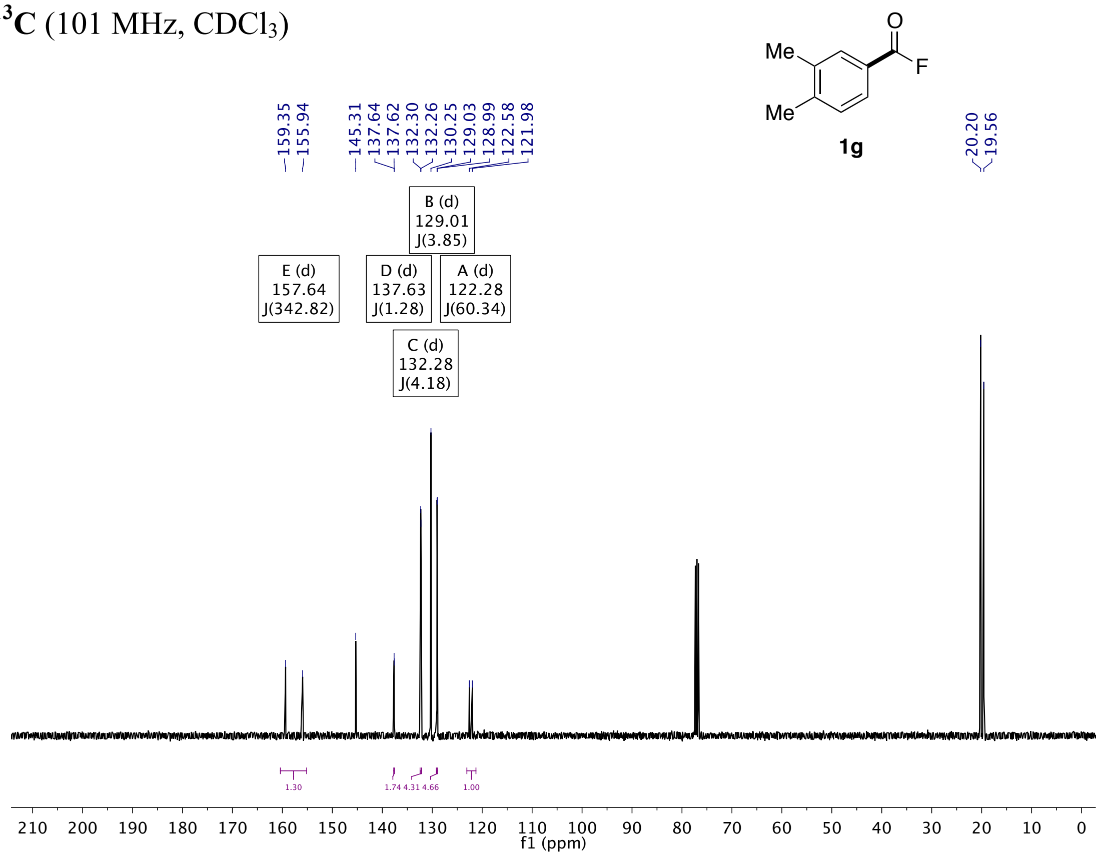

1g

^{1}H (600 MHz, $CDCl_3$)

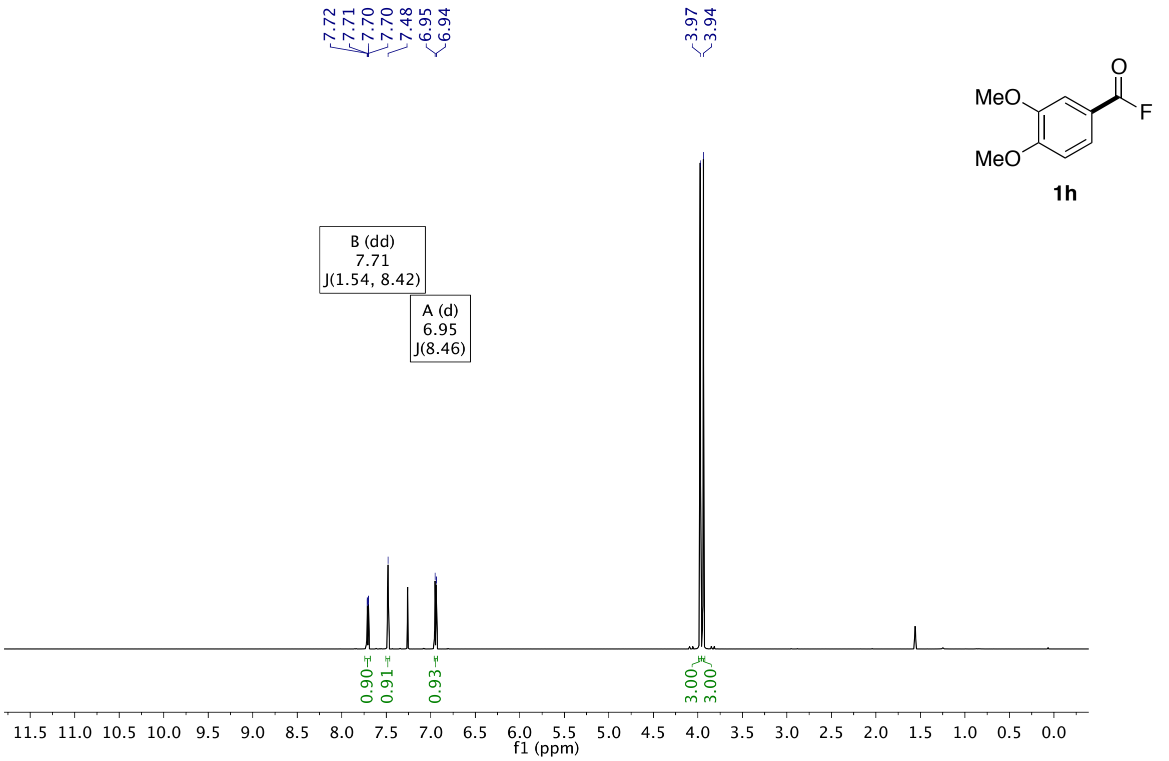

1h

^{19}**F** (376 MHz, $CDCl_3$)

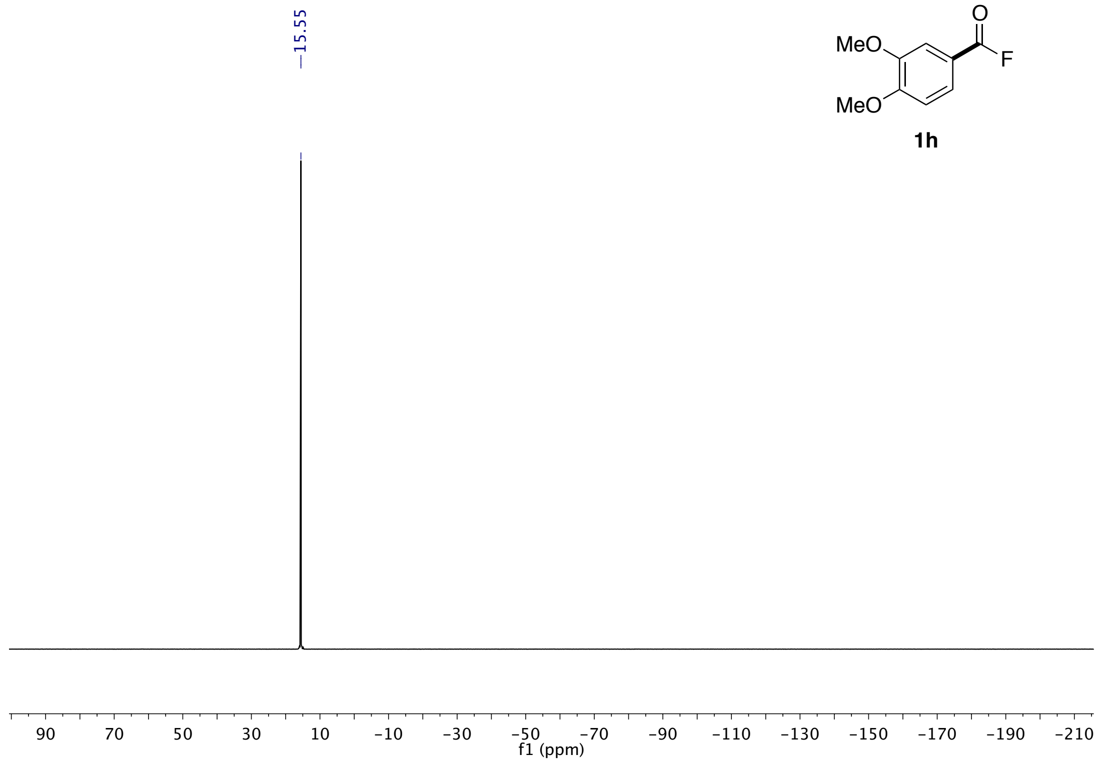

^{13}**C** (151 MHz, $CDCl_3$)

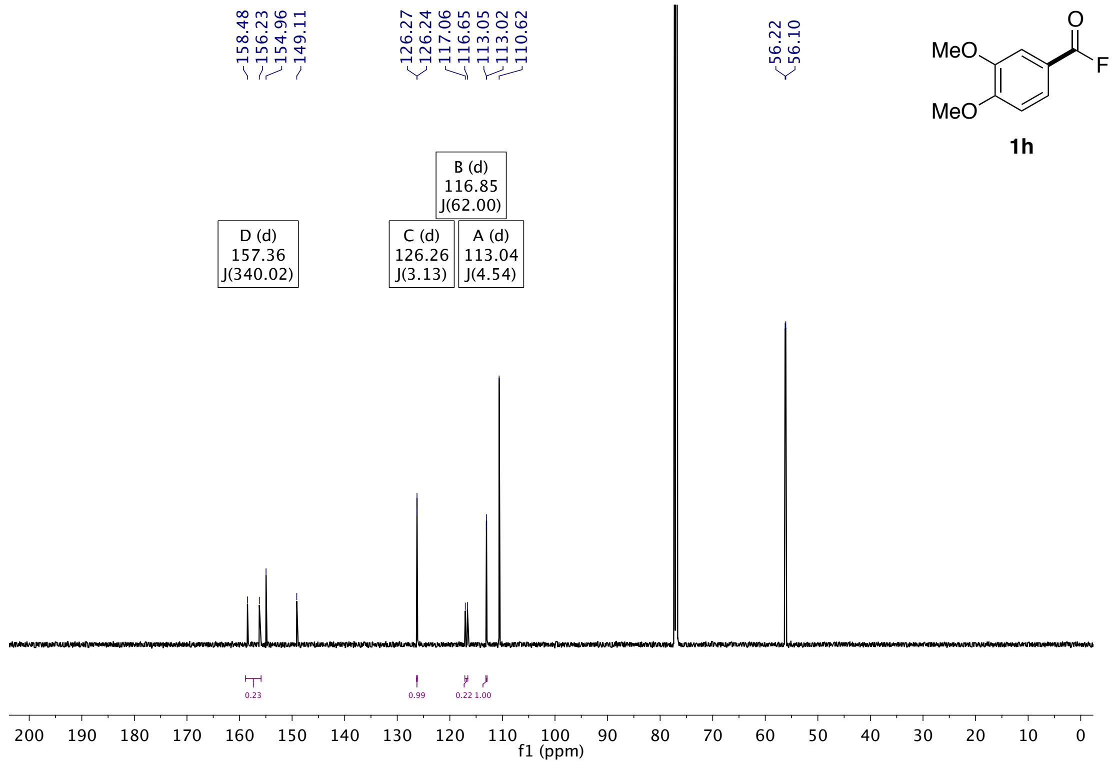

^{1}**H** (600 MHz, CDCl$_3$)

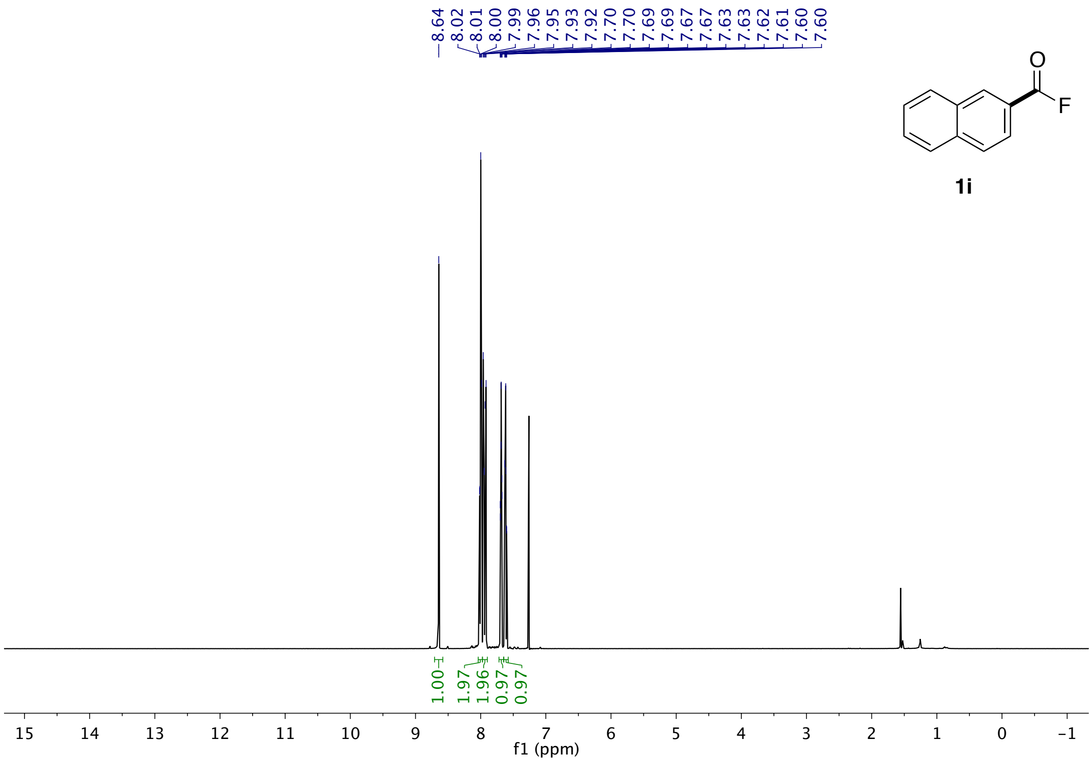

^{19}**F** (376 MHz, CDCl$_3$)

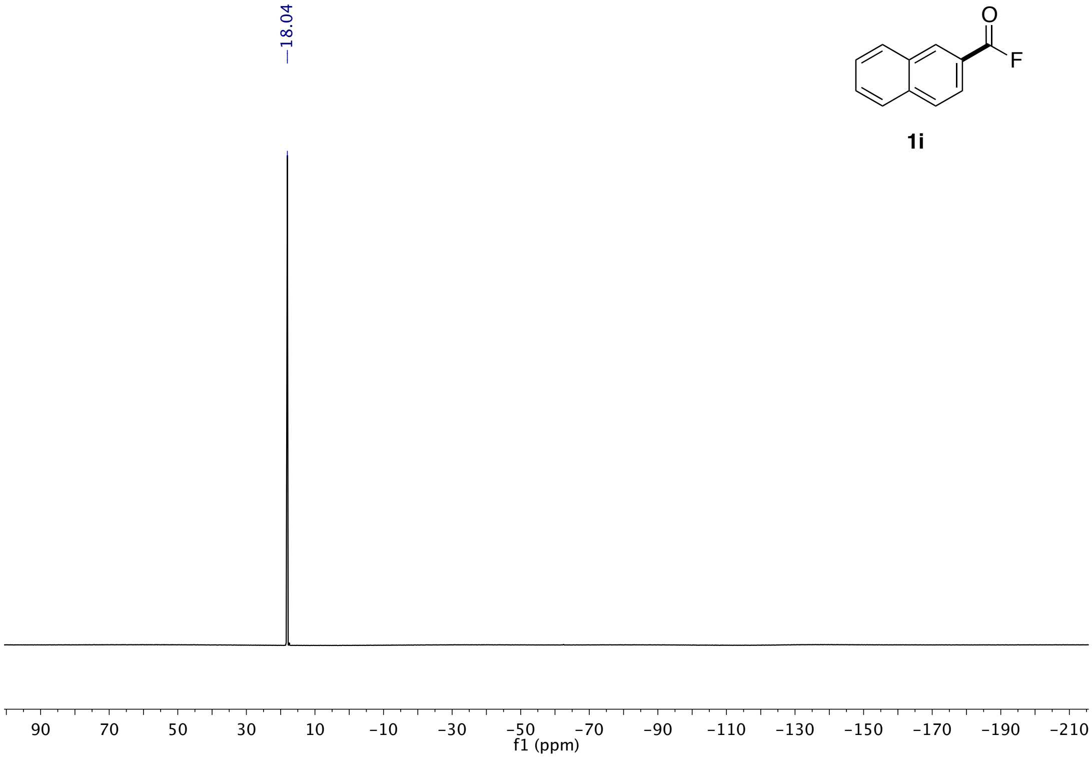

^{13}**C** (151 MHz, $CDCl_3$)

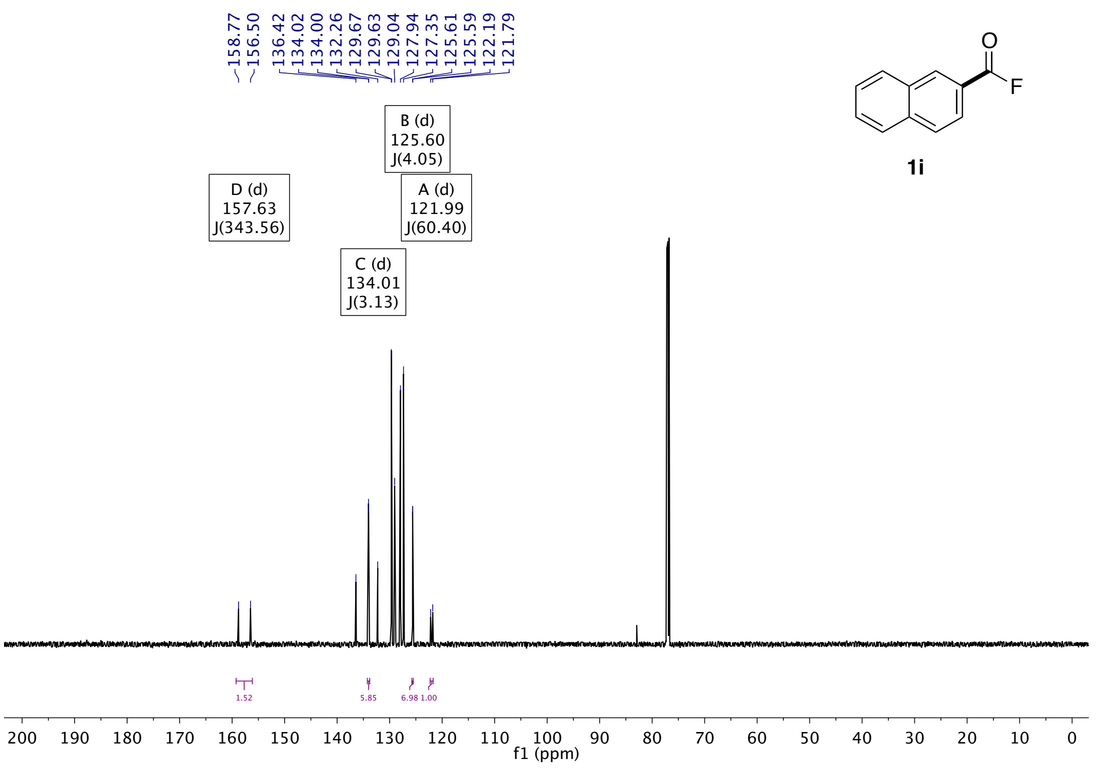

* the peak at 83.3 ppm is an artifact from the spectrometer

^{1}**H** (600 MHz, $CDCl_3$)

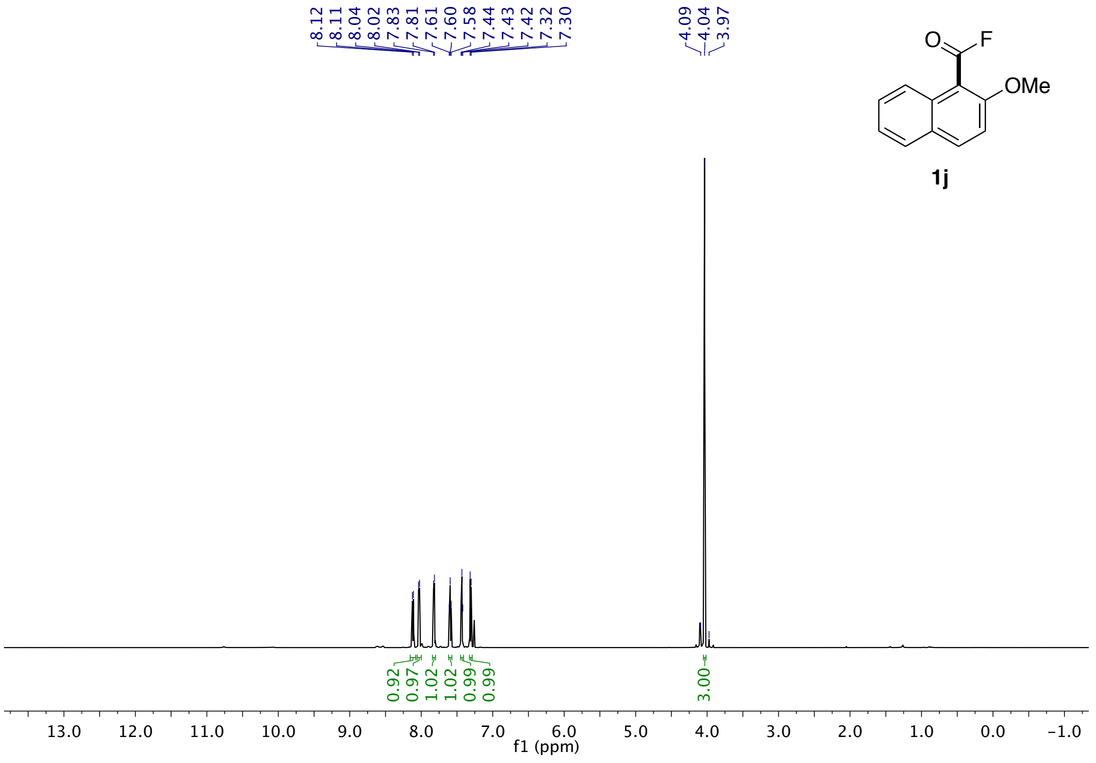

^{19}F (564 MHz, $CDCl_3$)

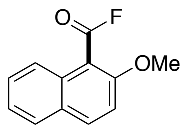

1j

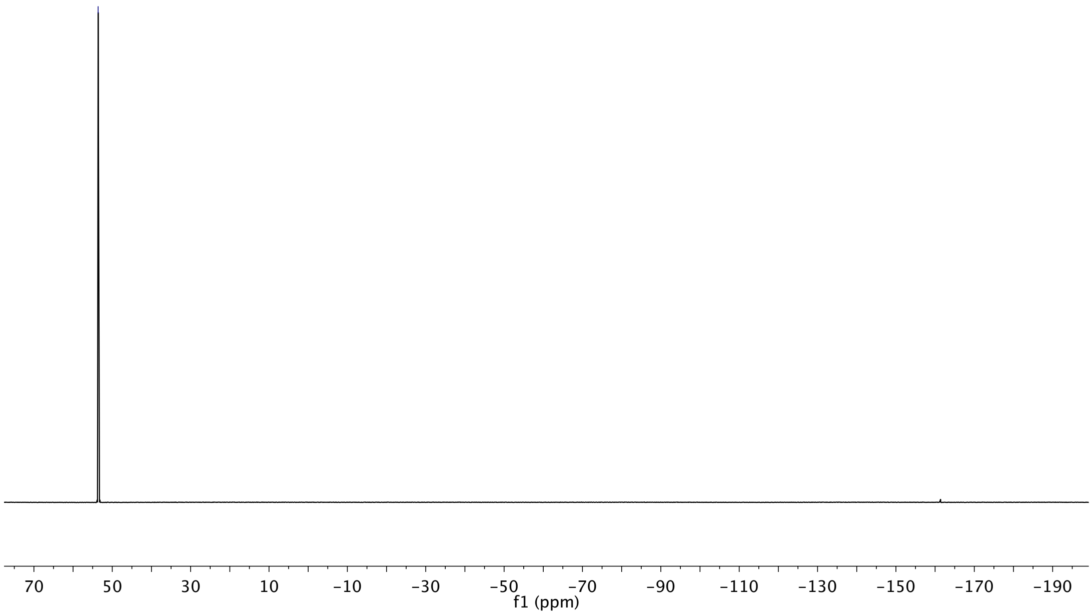

^{13}C (151 MHz, $CDCl_3$)

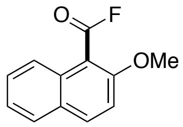

1j

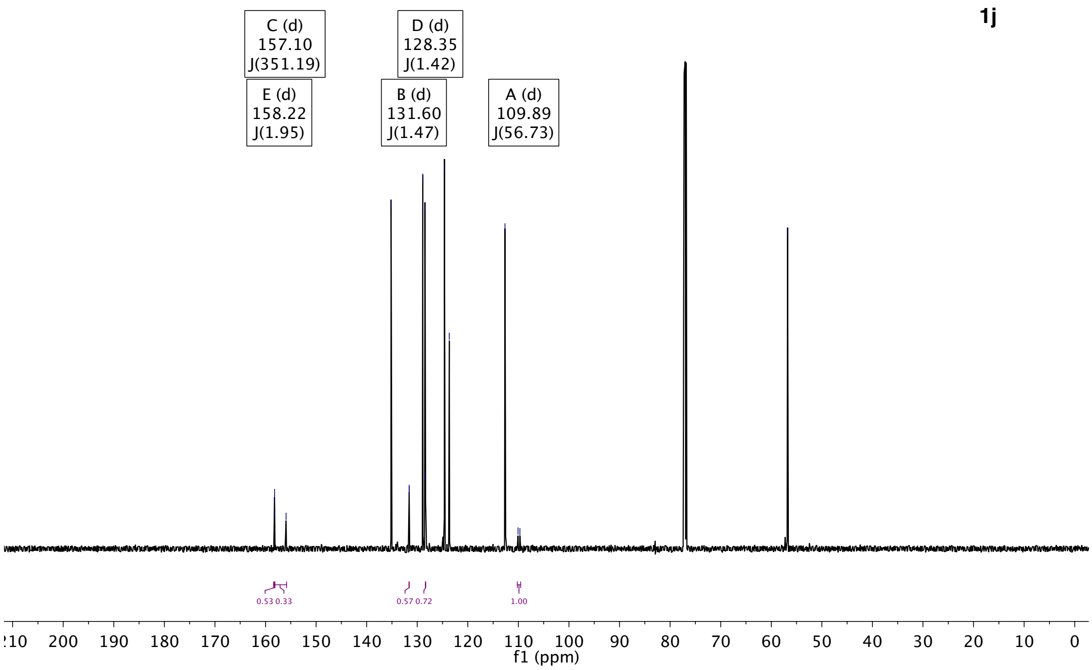

* the peak at 83.3 ppm is an artifact from the spectrometer

^{1}**H** (600 MHz, CDCl$_3$)

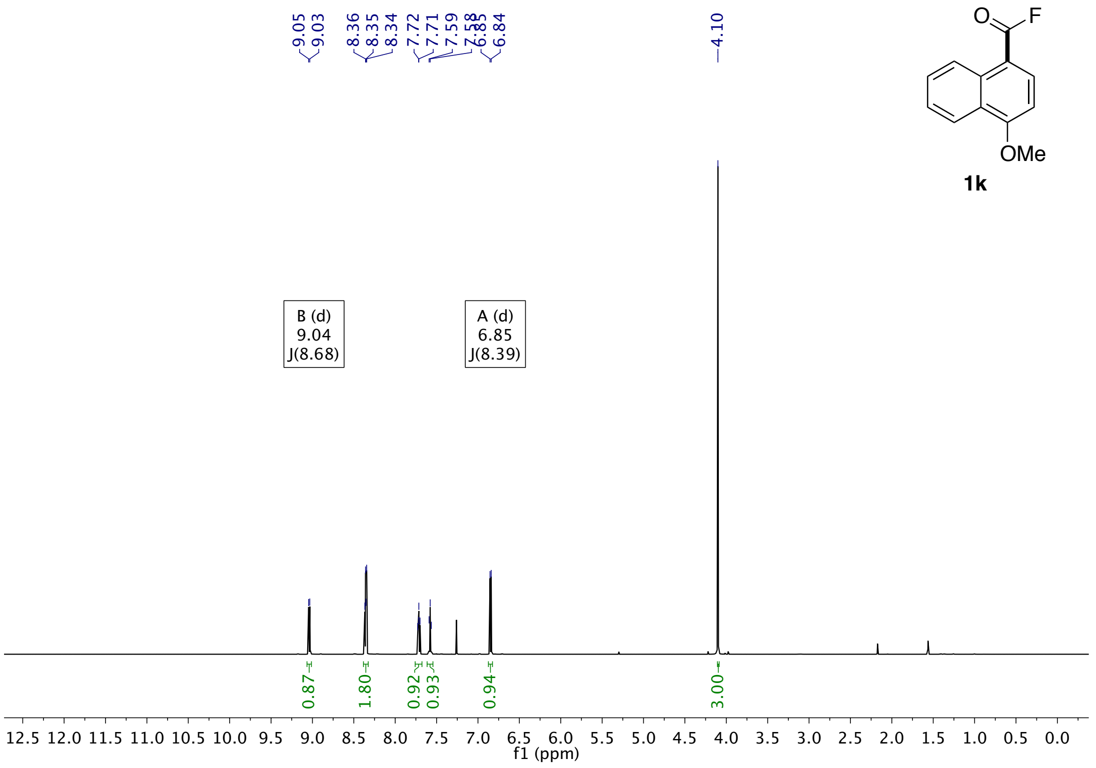

^{19}**F** (564 MHz, CDCl$_3$)

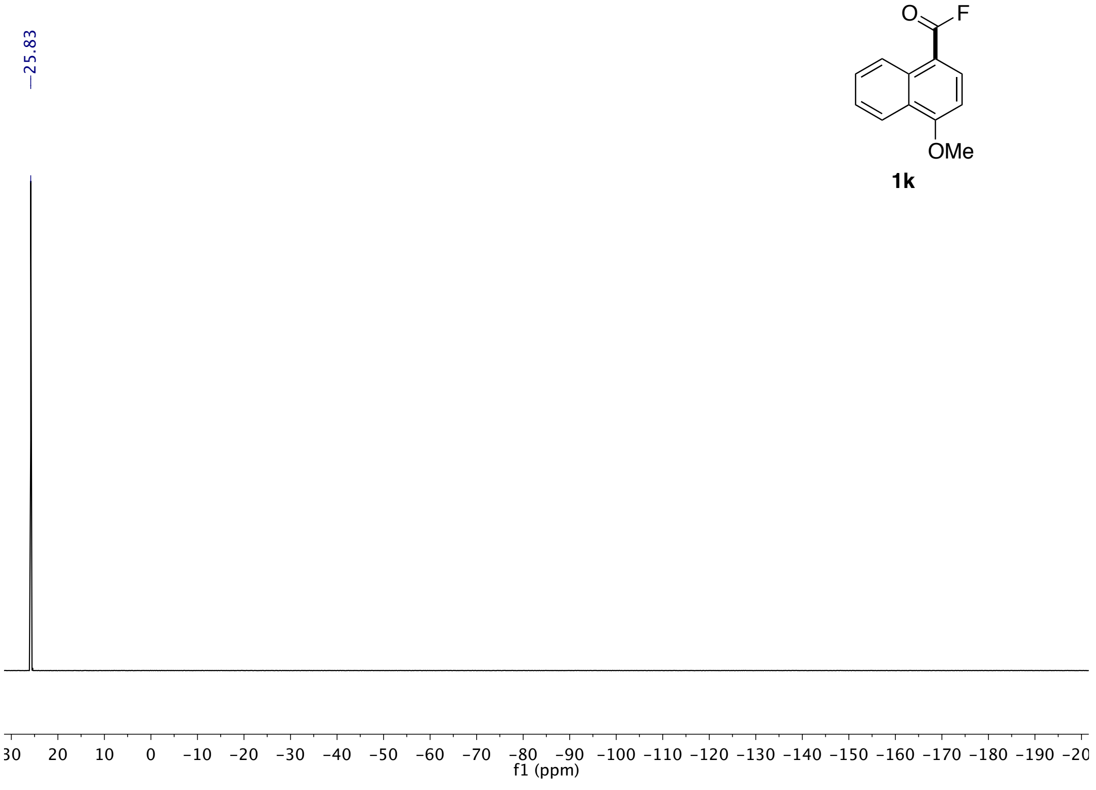

^{13}**C** (151 MHz, $CDCl_3$)

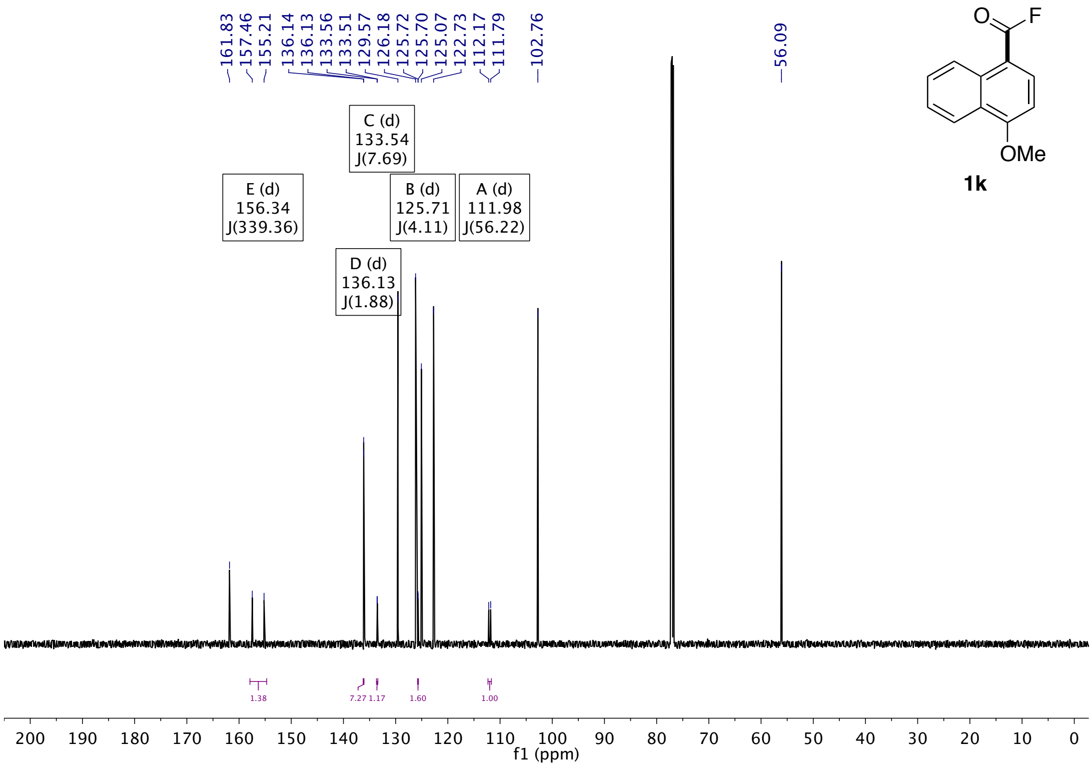

1k

^{1}**H** (600 MHz, CD_3CN)

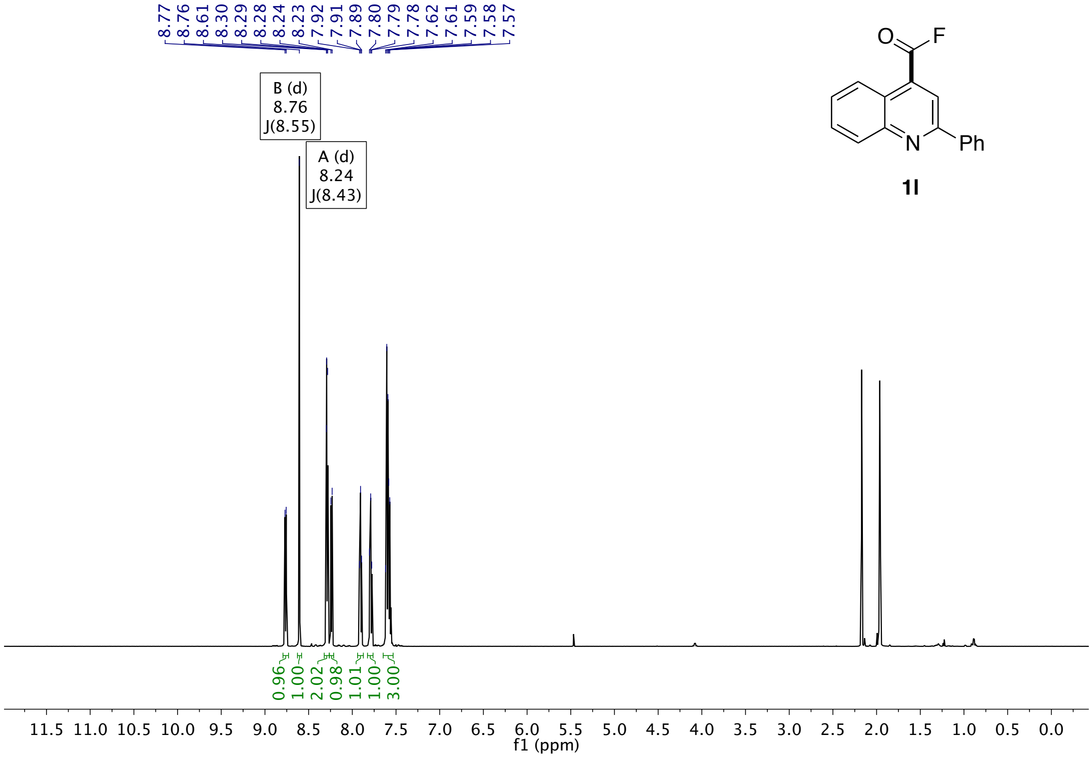

1l

^{19}**F** (376 MHz, CD_3CN)

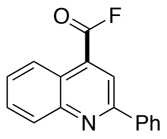

1l

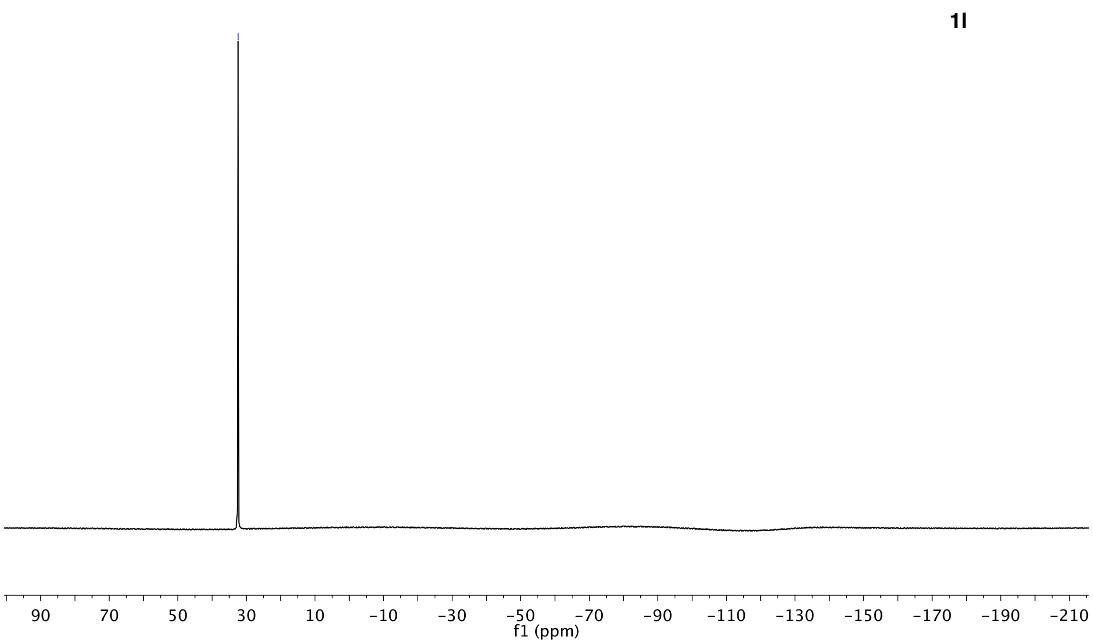

^{13}**C** (151 MHz, CD_3CN)

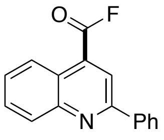

1l

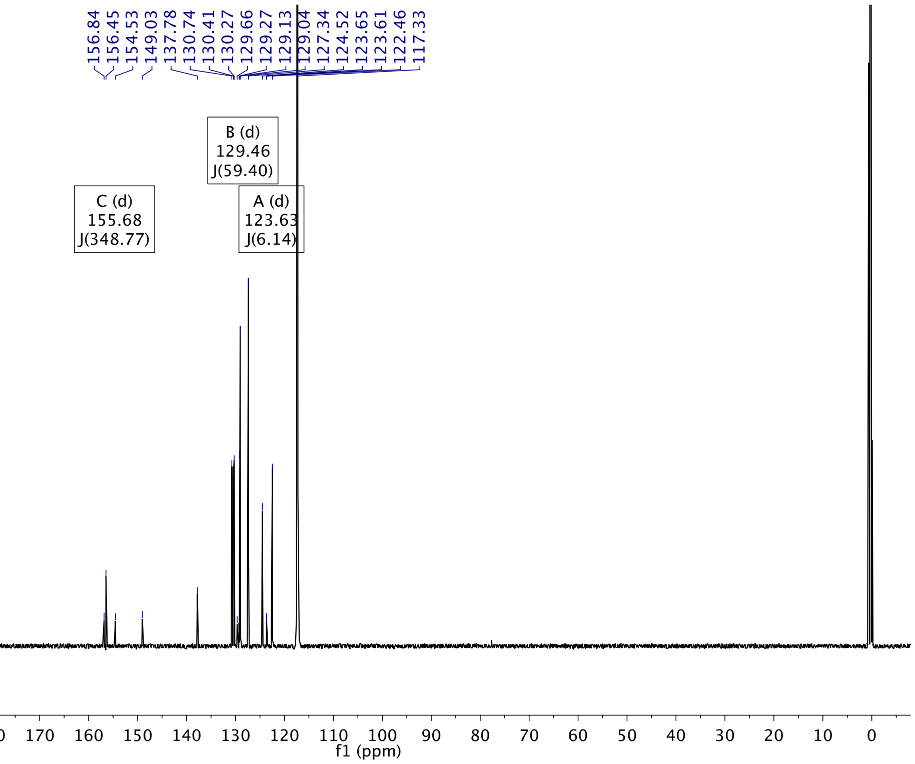

^{1}**H** (600 MHz, $CDCl_3$)

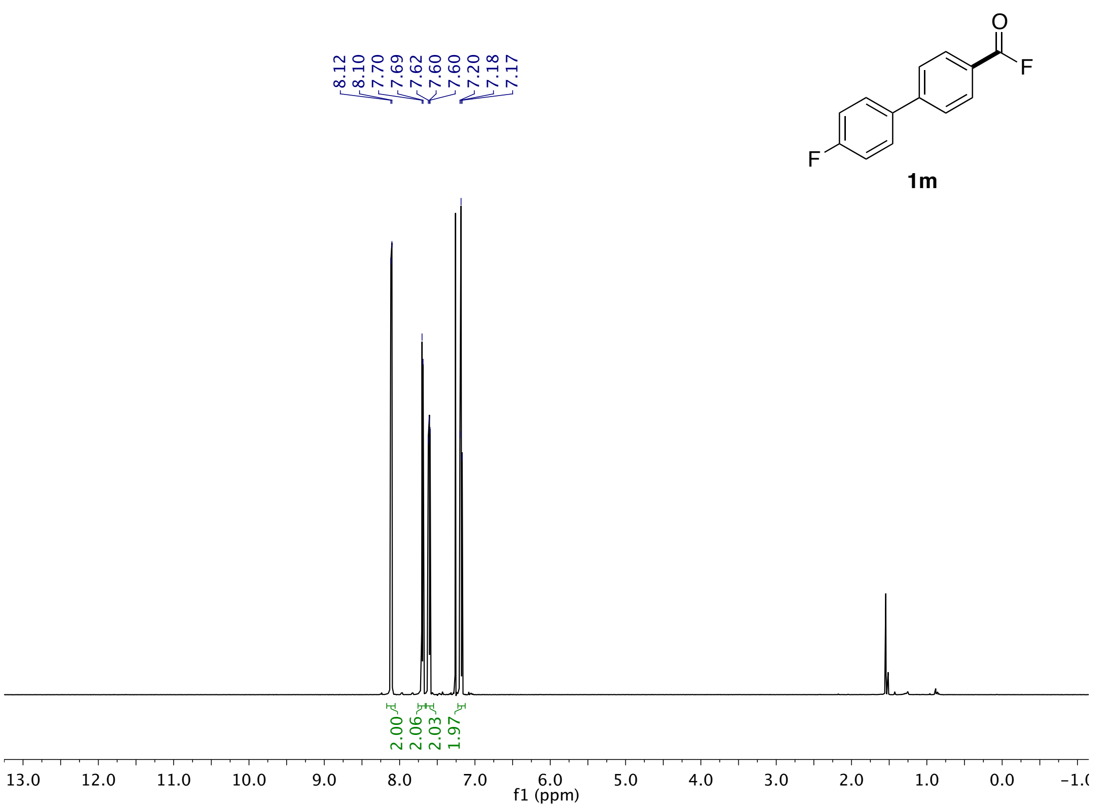

^{19}**F** (564 MHz, $CDCl_3$)

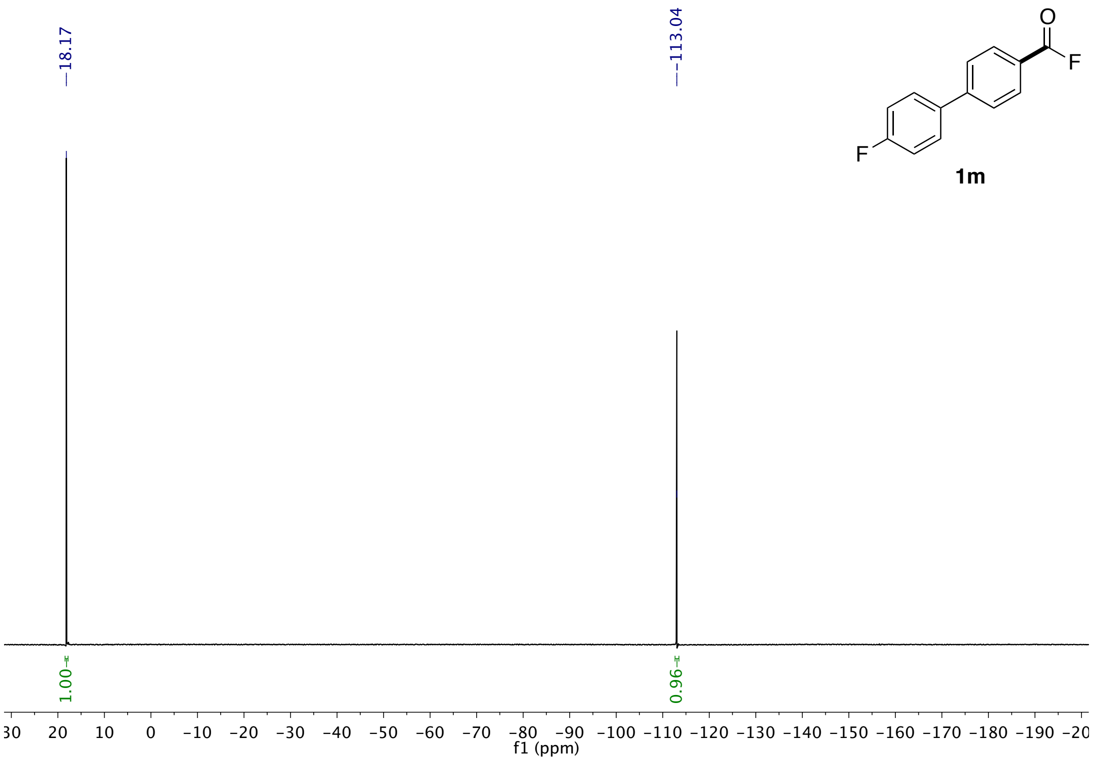

13C (151 MHz, $CDCl_3$)

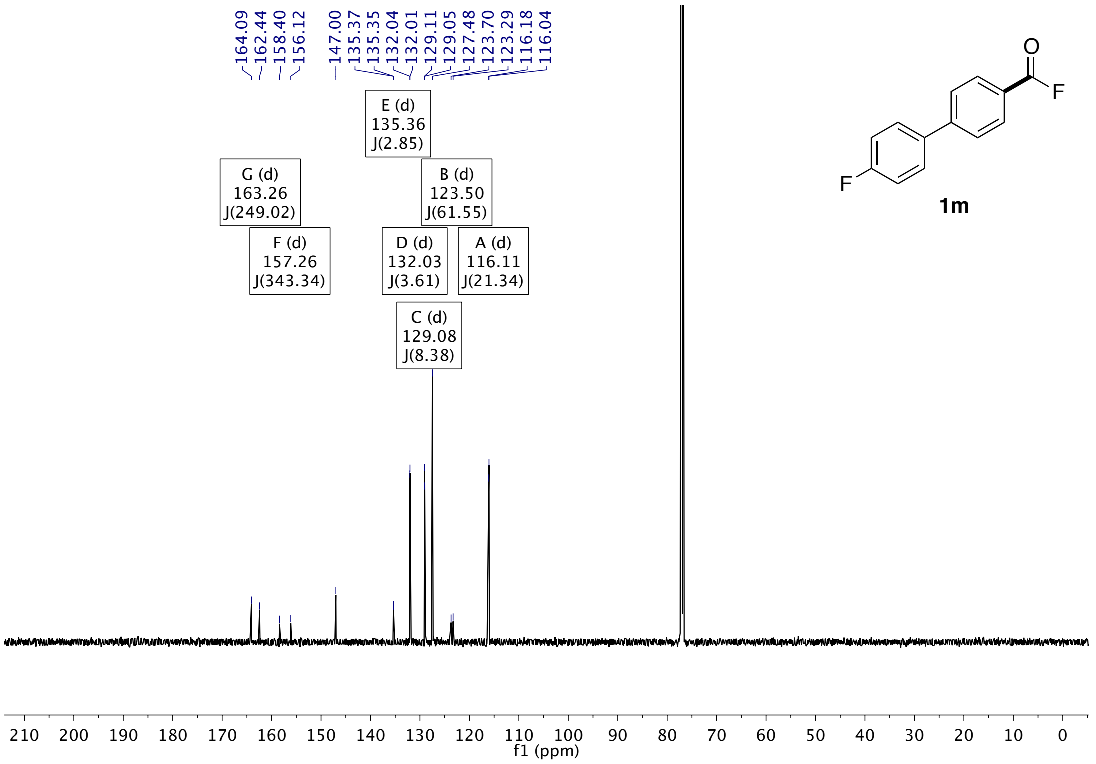

1H (600 MHz, $CDCl_3$)

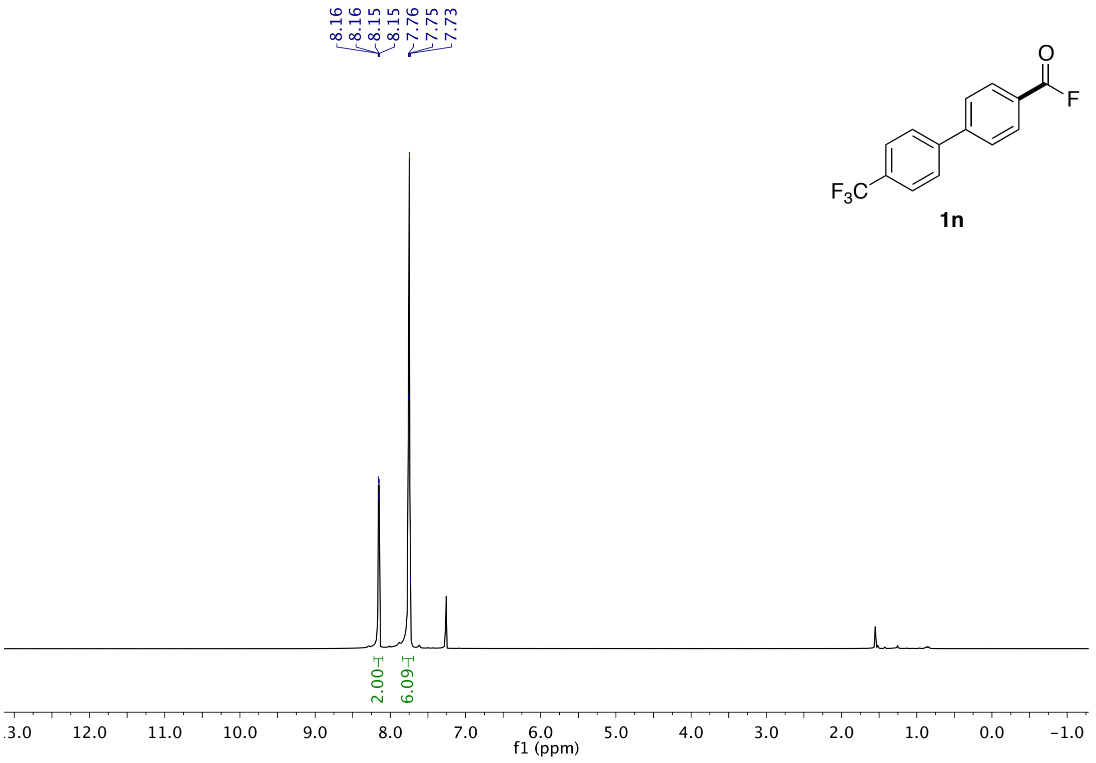

^{19}F (564 MHz, $CDCl_3$)

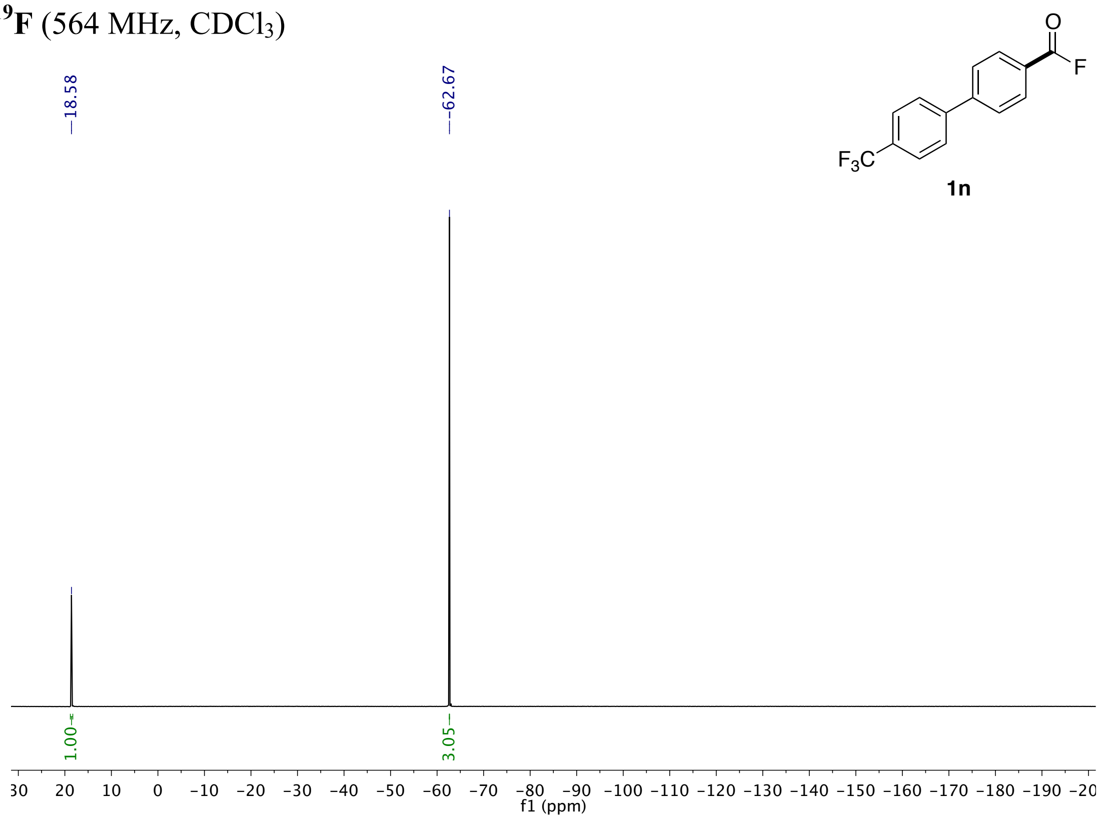

^{13}C (151 MHz, $CDCl_3$)

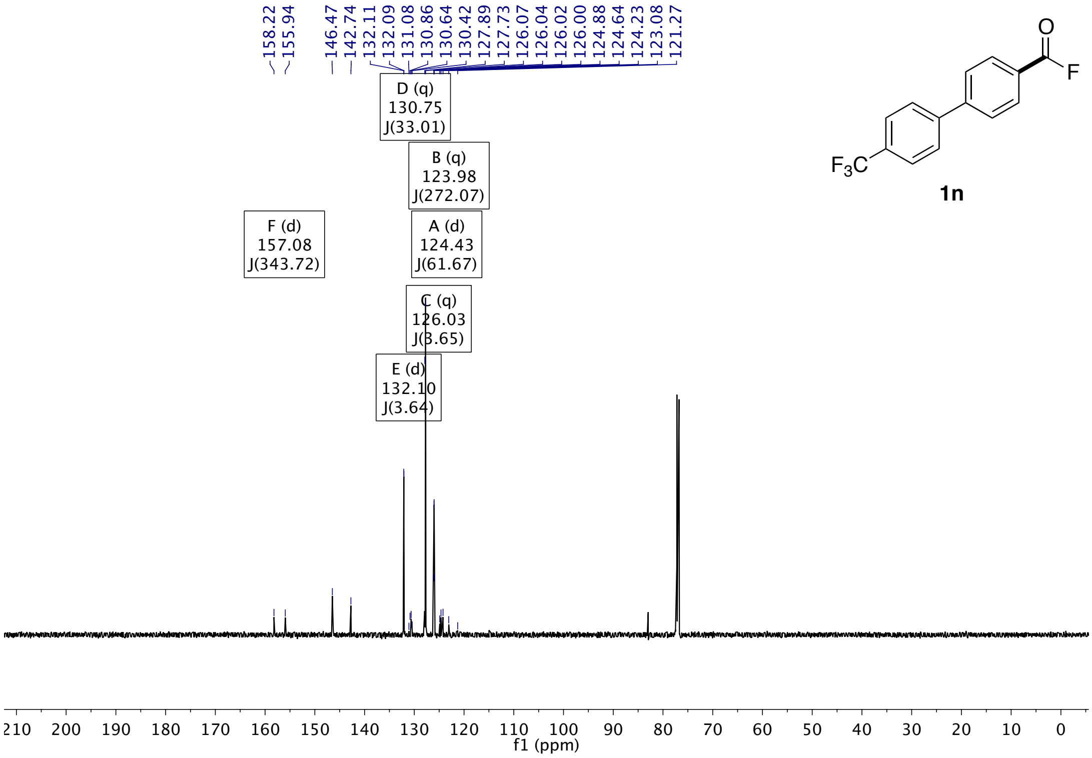

* the peak at 83.3 ppm is an artifact from the spectrometer

^{1}H (600 MHz, $CDCl_3$)

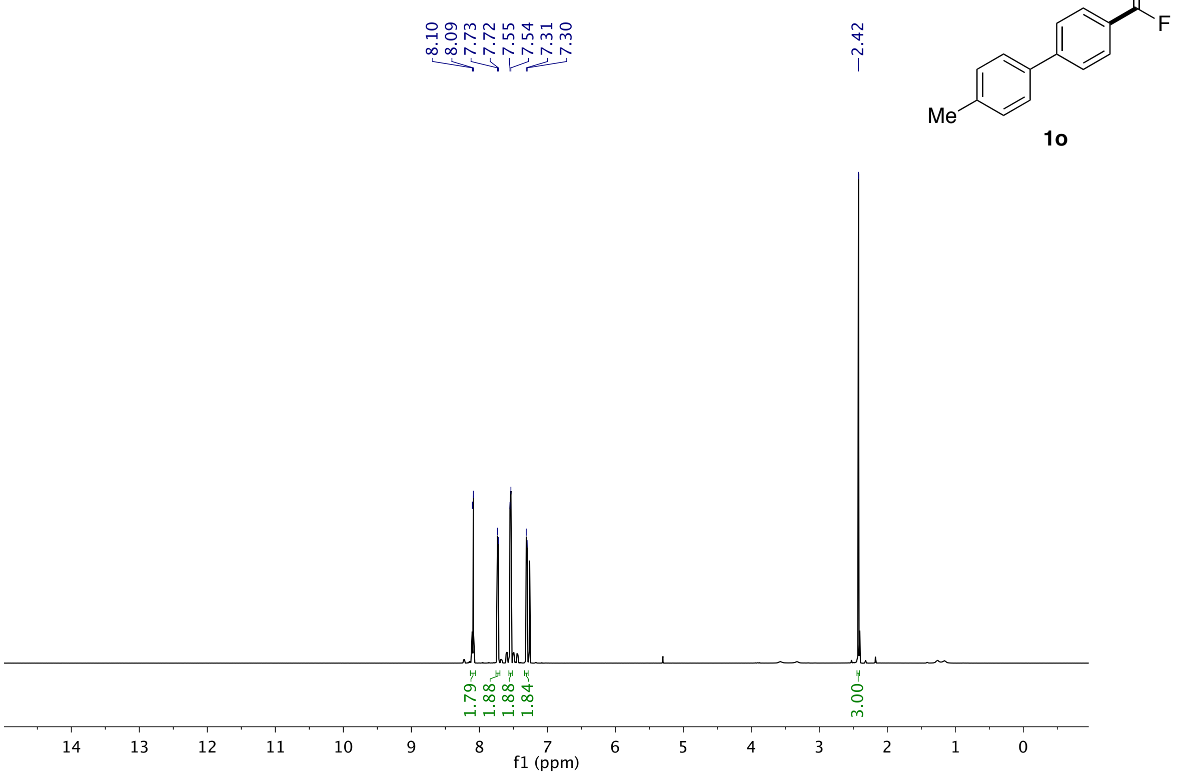

^{19}F (376 MHz, $CDCl_3$)

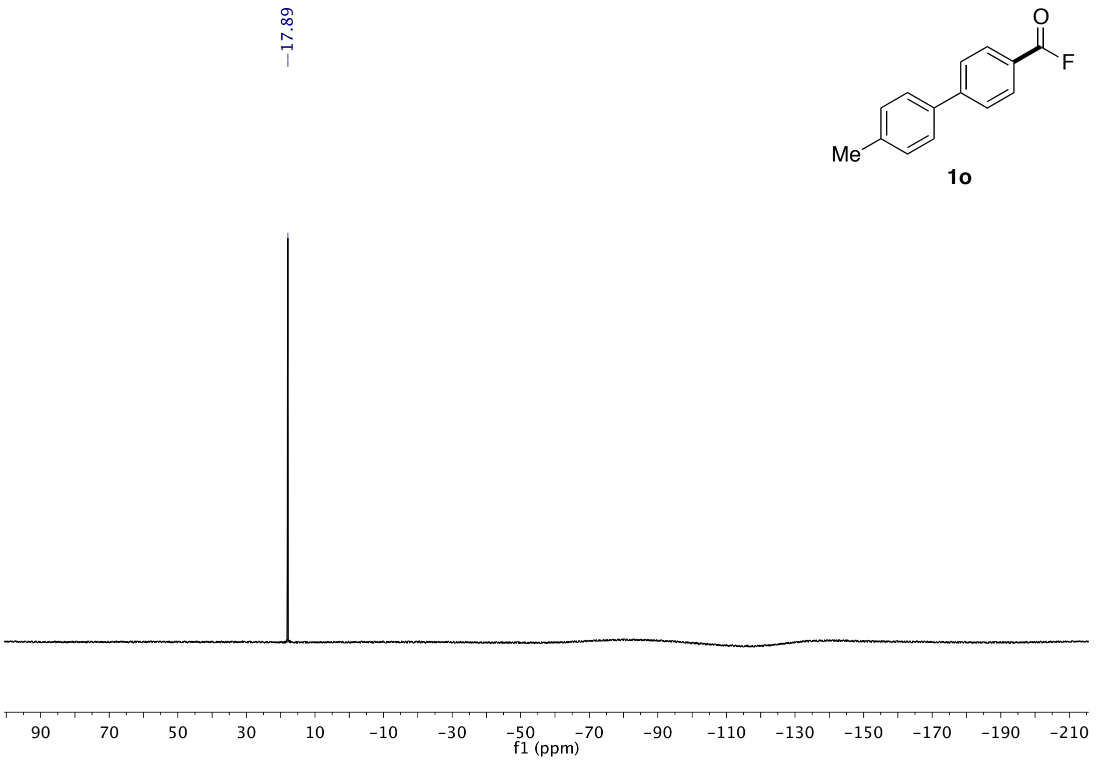

^{13}C (151 MHz, $CDCl_3$)

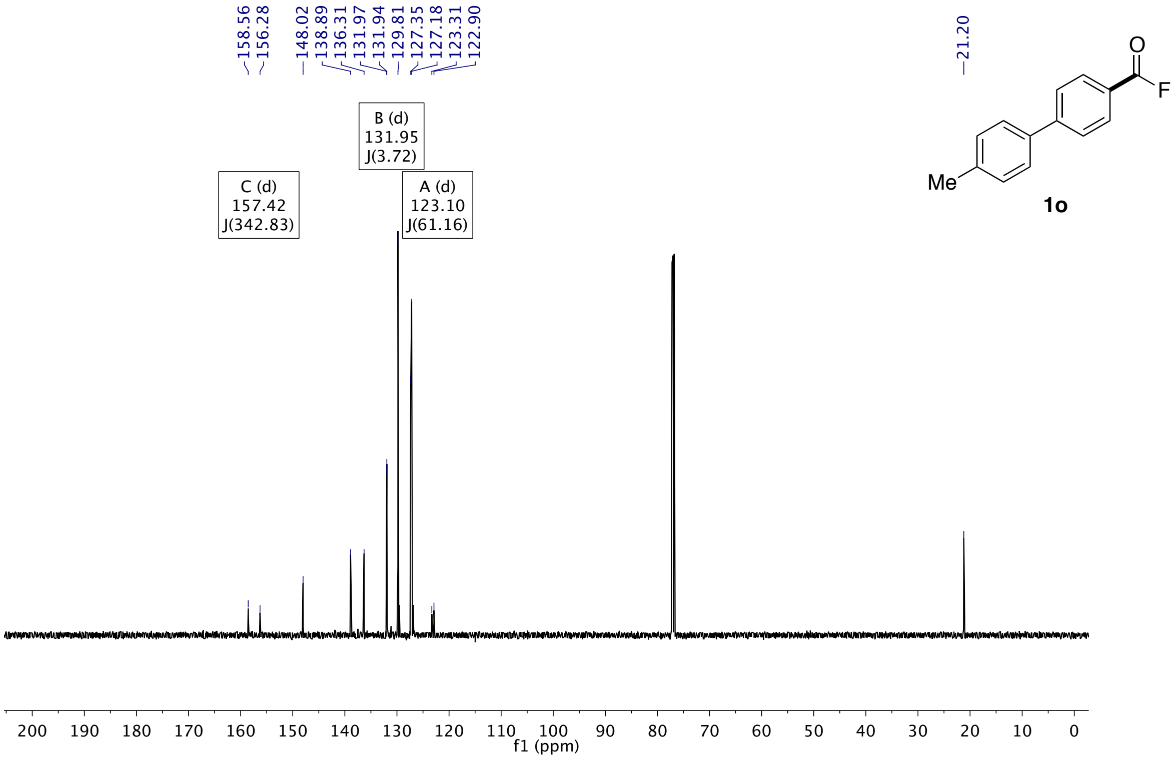

^{1}H (600 MHz, $CDCl_3$)

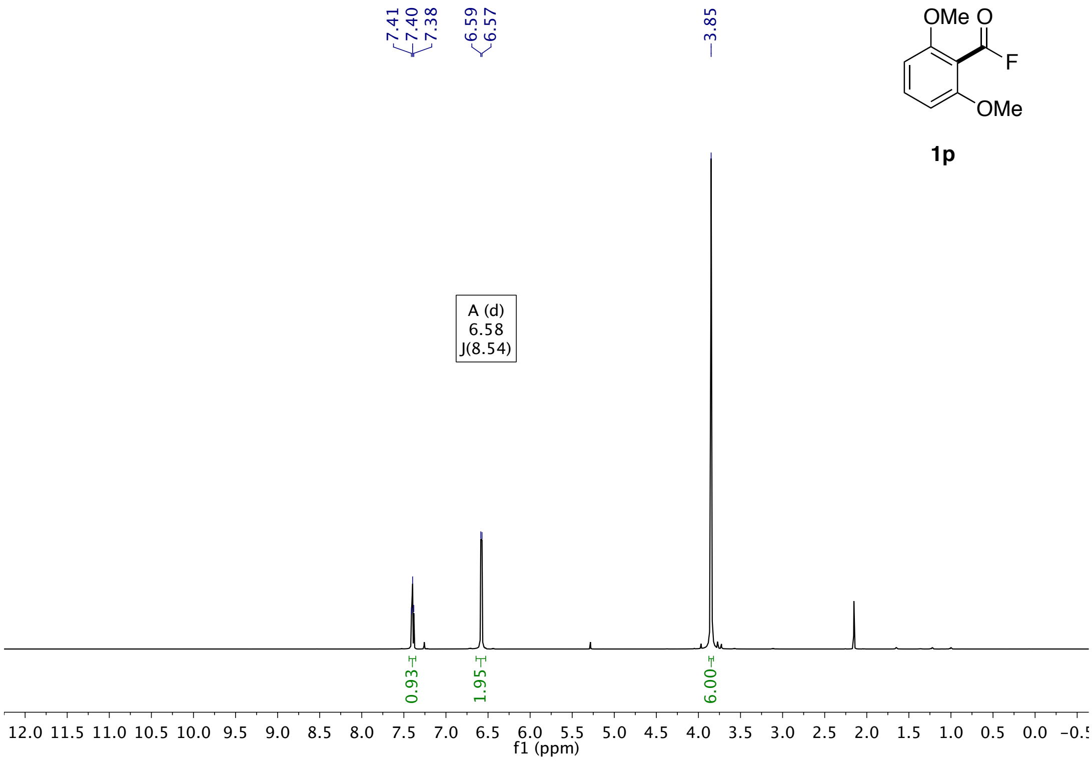

^{19}F (282 MHz, $CDCl_3$)

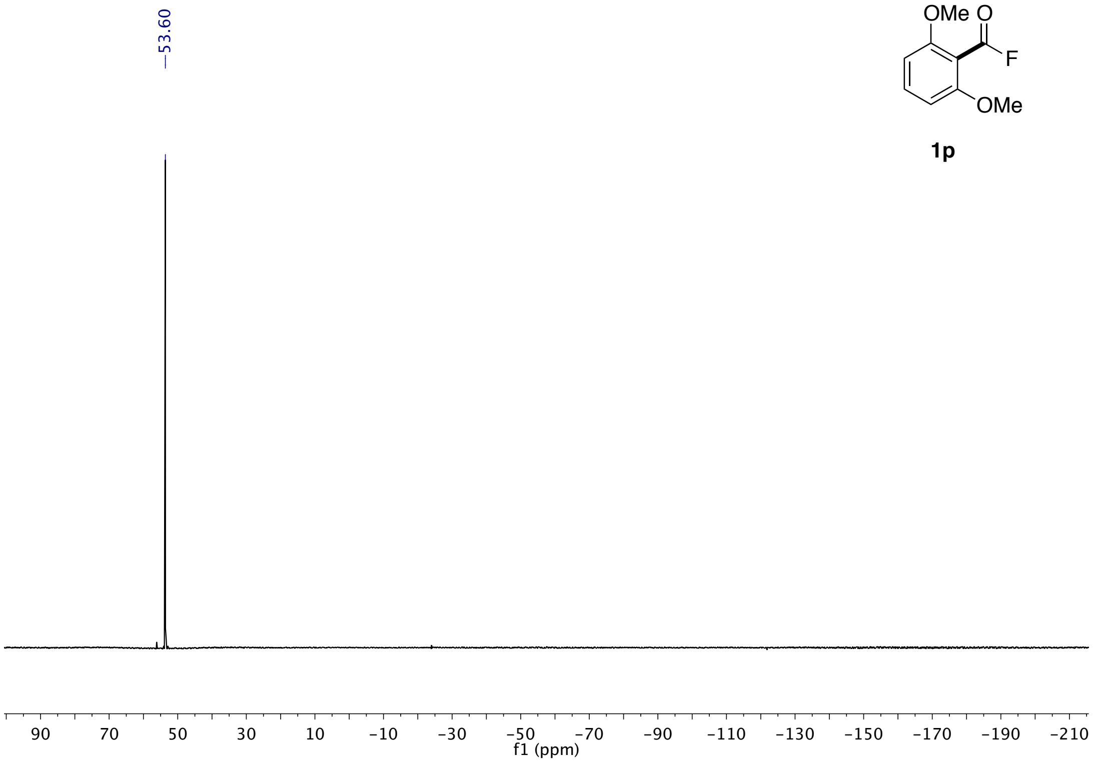

^{13}C (151 MHz, $CDCl_3$)

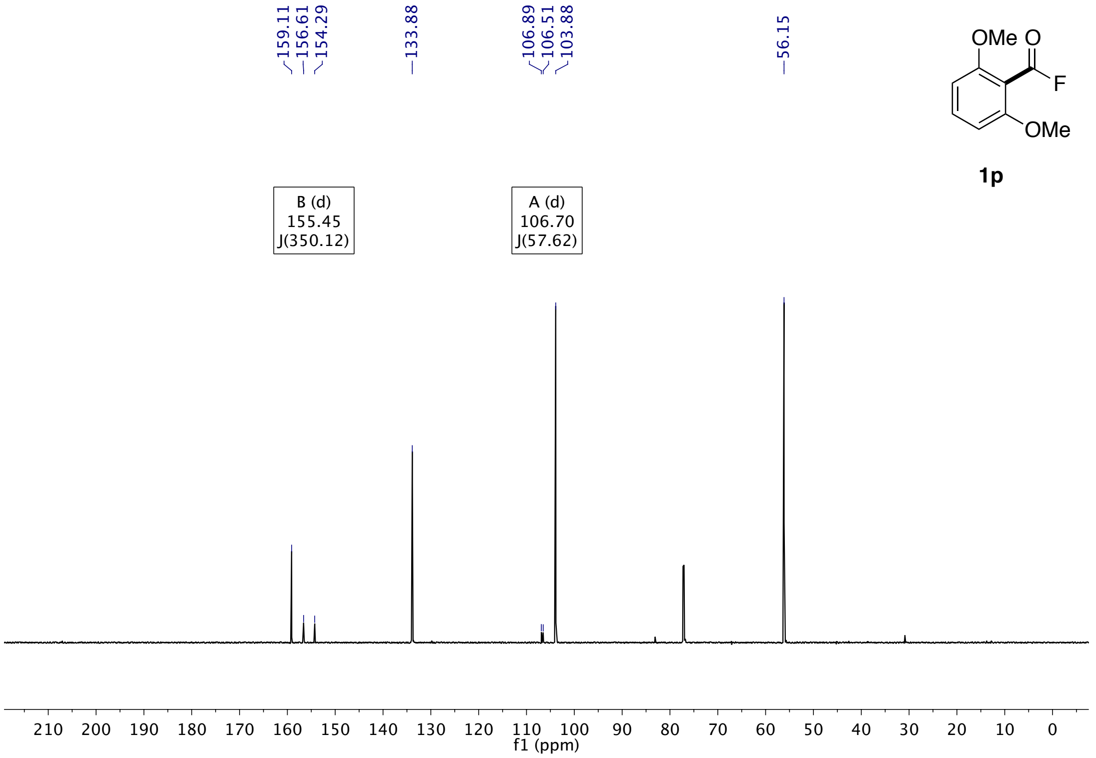

* the peak at 83.3 ppm is an artifact from the spectrometer

Trifluoromethyl arenes

^{1}**H** (400 MHz, $CDCl_3$)

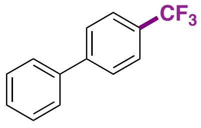

2a

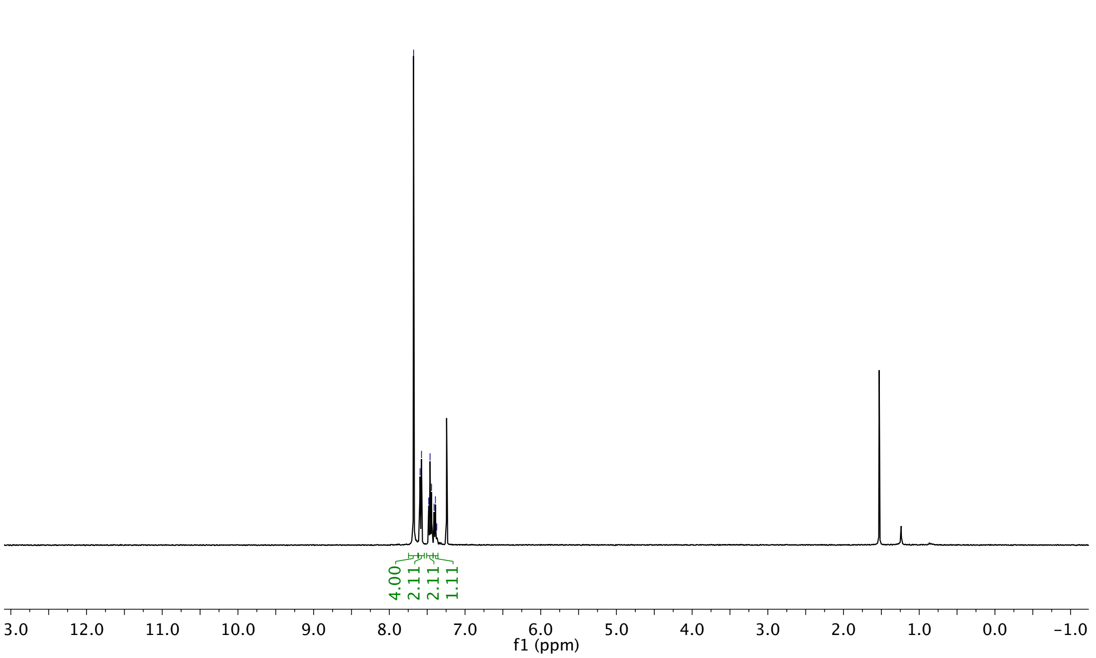

^{19}**F** (376 MHz, $CDCl_3$)

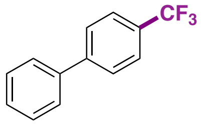

2a

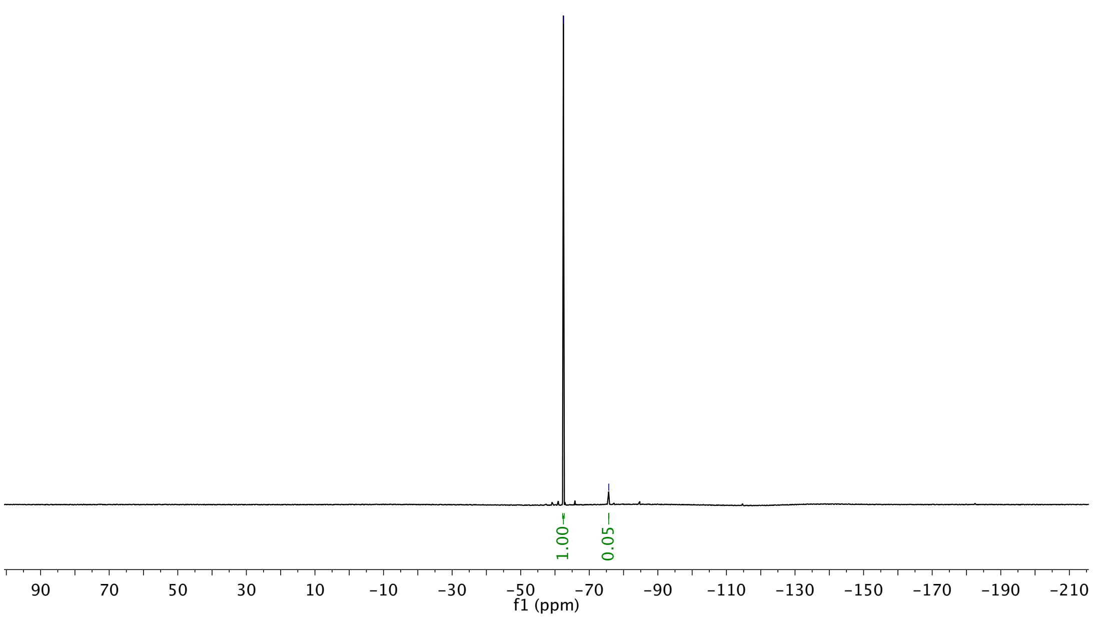

^{13}C (151 MHz, $CDCl_3$)

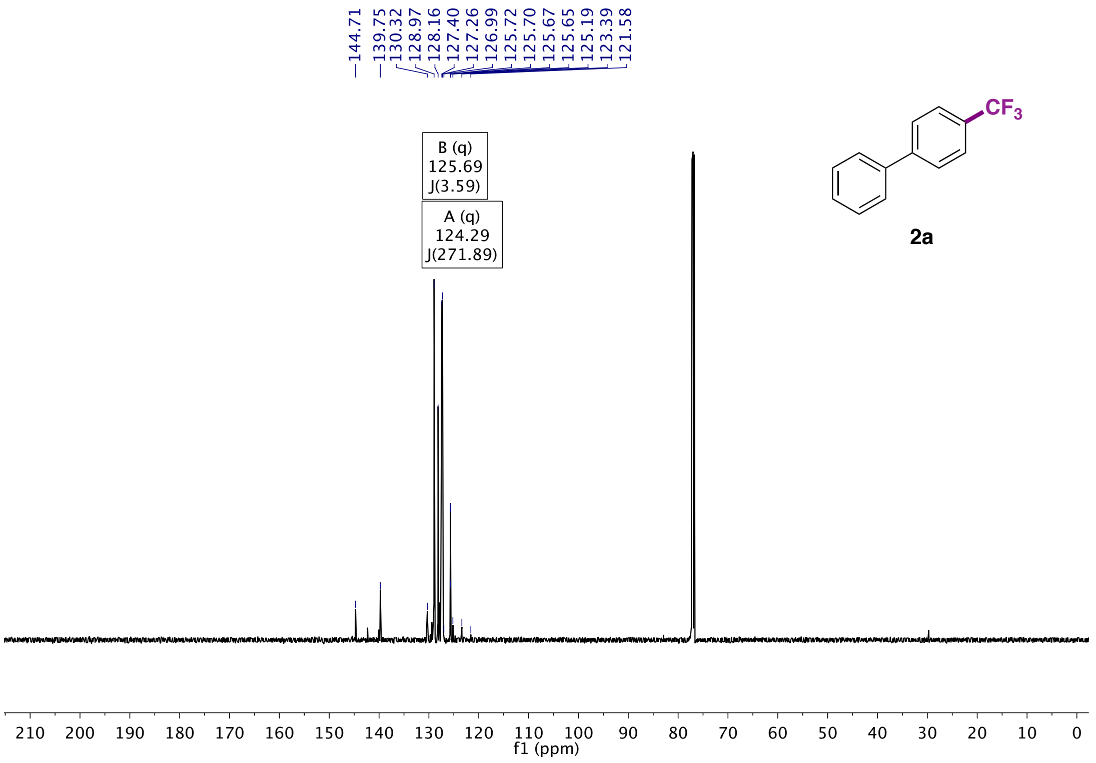

^{1}H (400 MHz, $CDCl_3$)

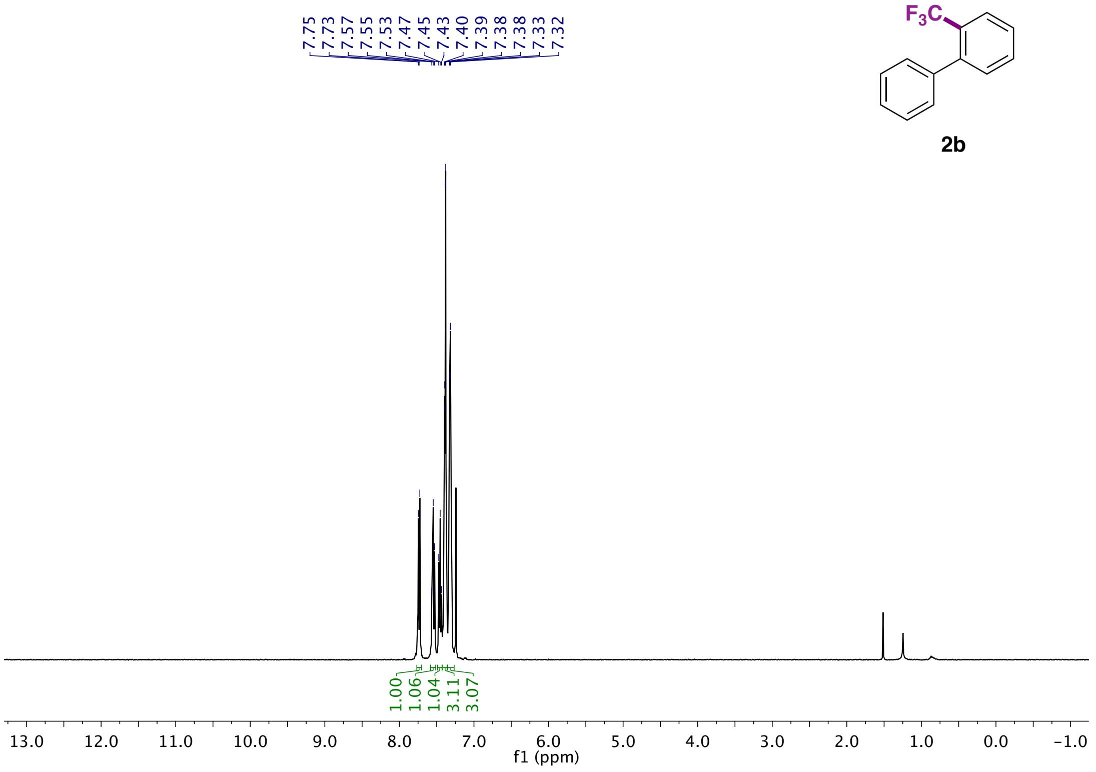

19F (564 MHz, $CDCl_3$)

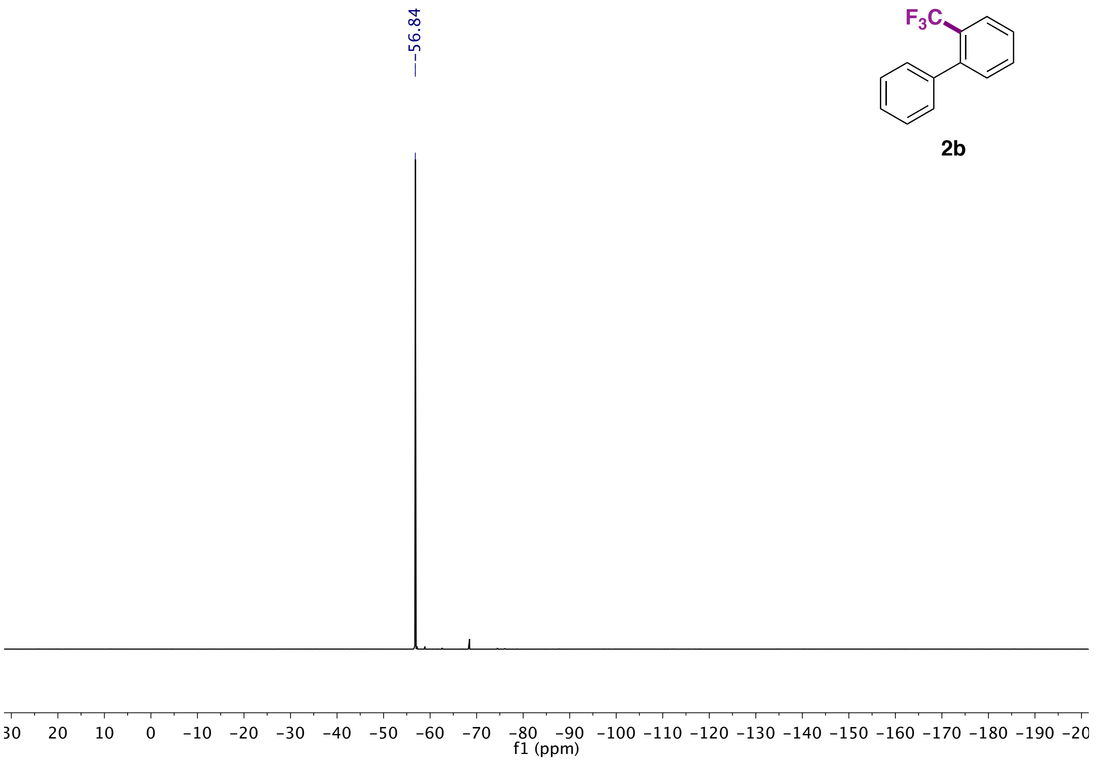

13C (151 MHz, $CDCl_3$)

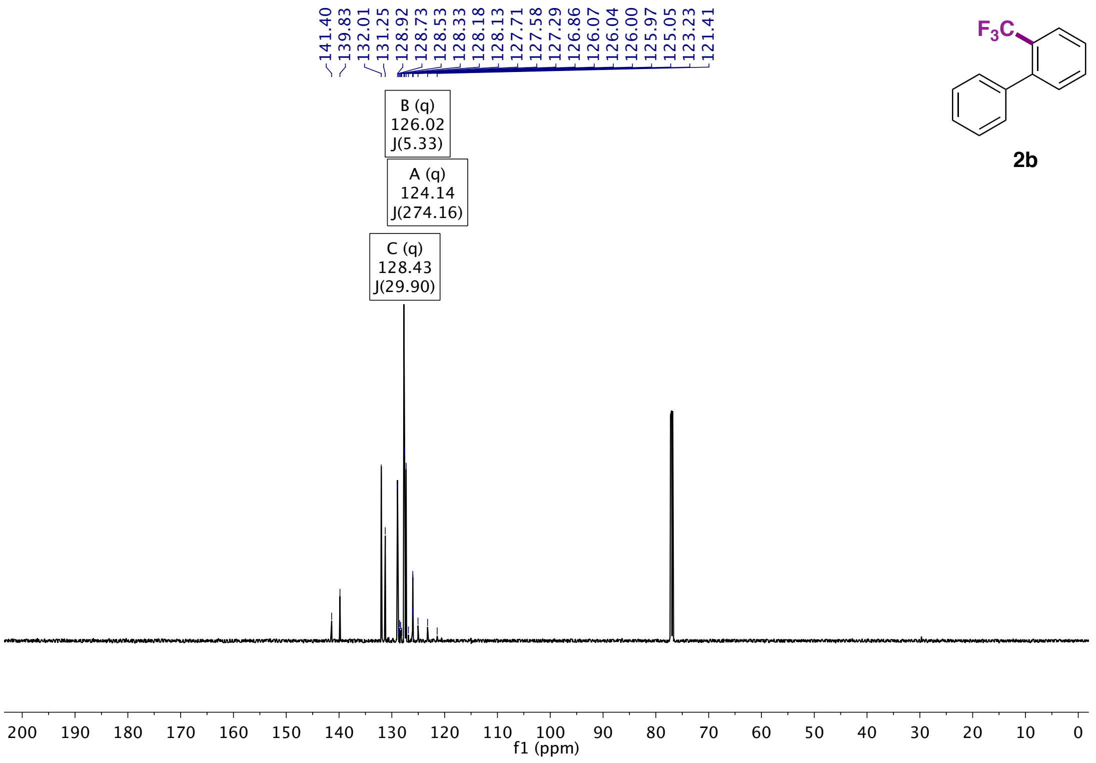

^{1}H (400 MHz, $CDCl_3$)

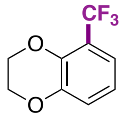

2c

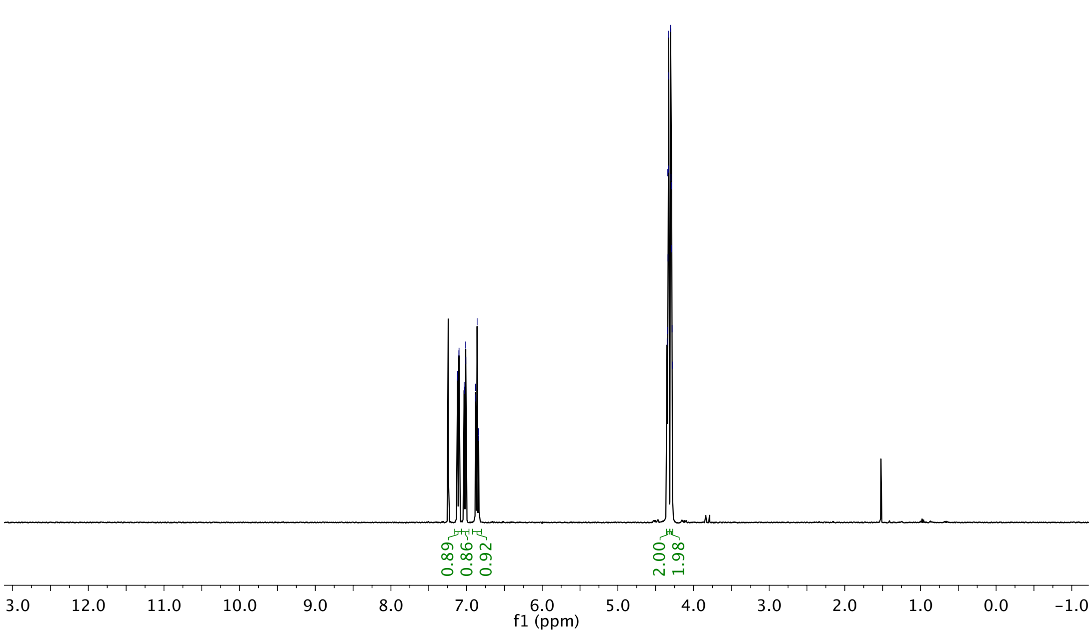

^{19}F (564 MHz, $CDCl_3$)

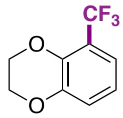

2c

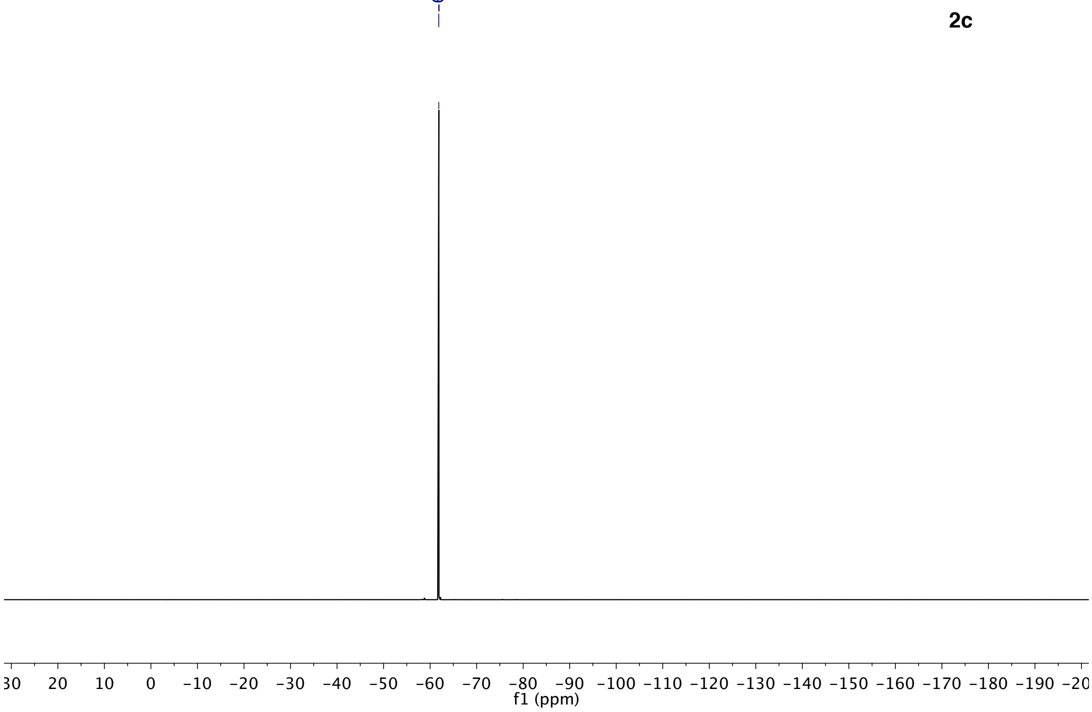

^{13}C (151 MHz, $CDCl_3$)

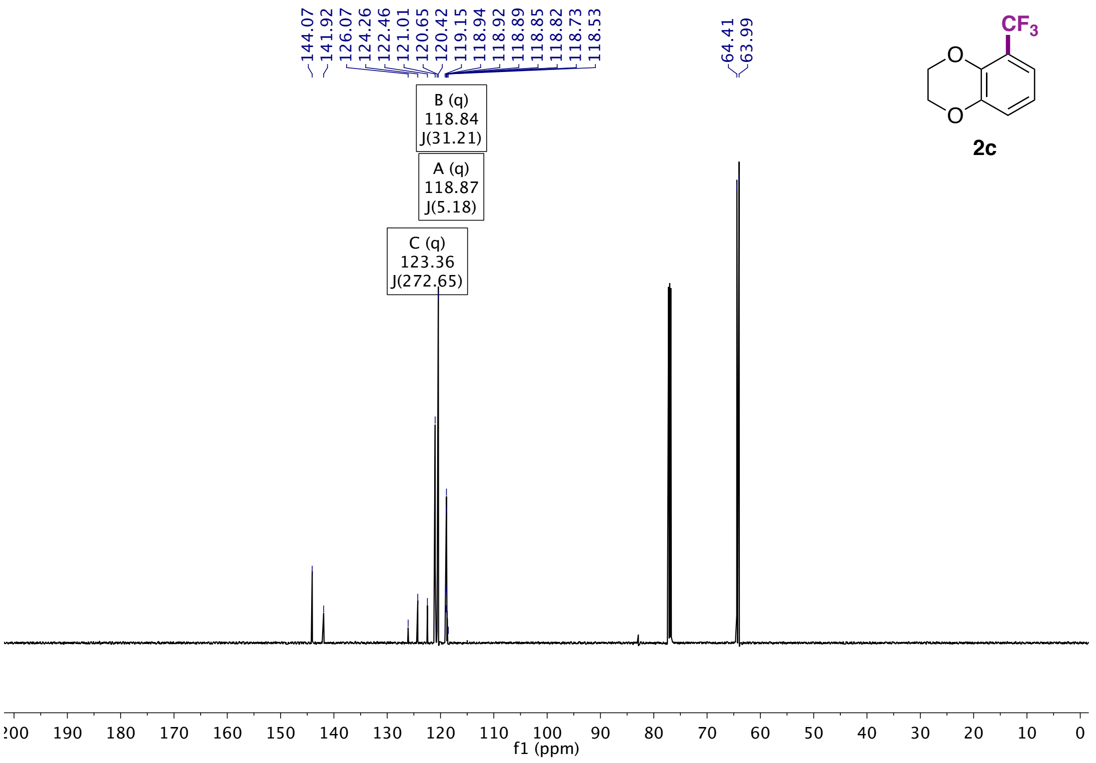

* the peak at 83.3 ppm is an artifact from the spectrometer

^{1}H (400 MHz, $CDCl_3$)

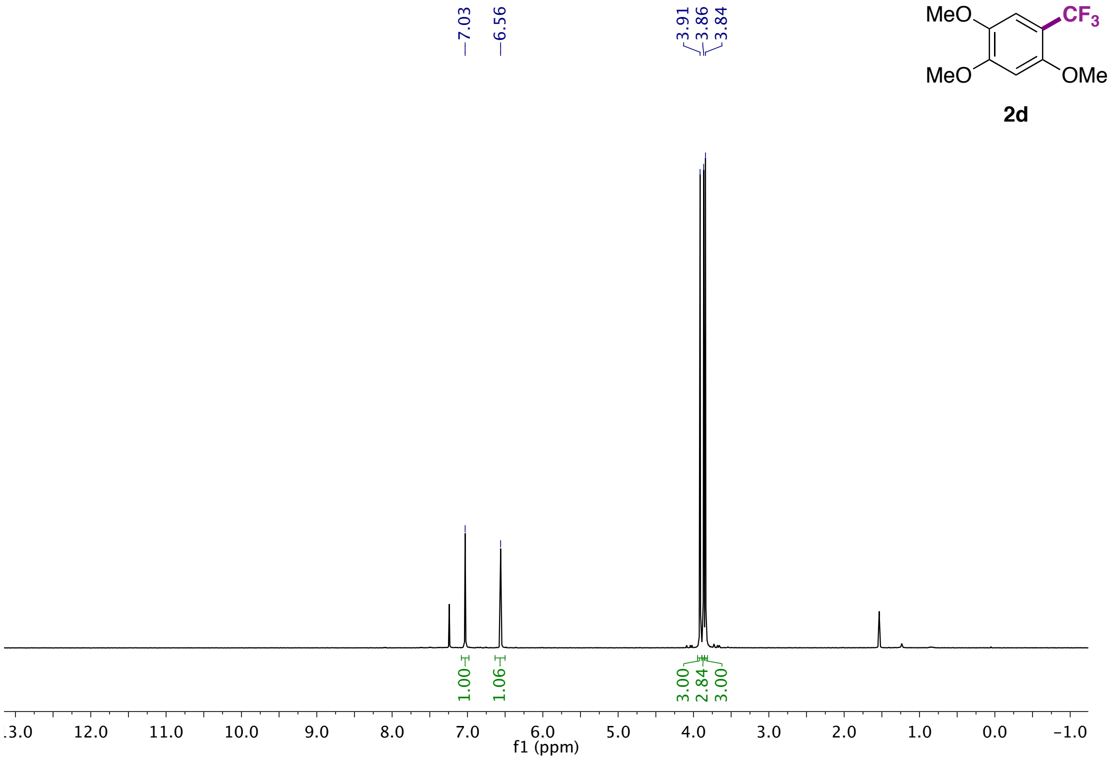

^{19}F (564 MHz, $CDCl_3$)

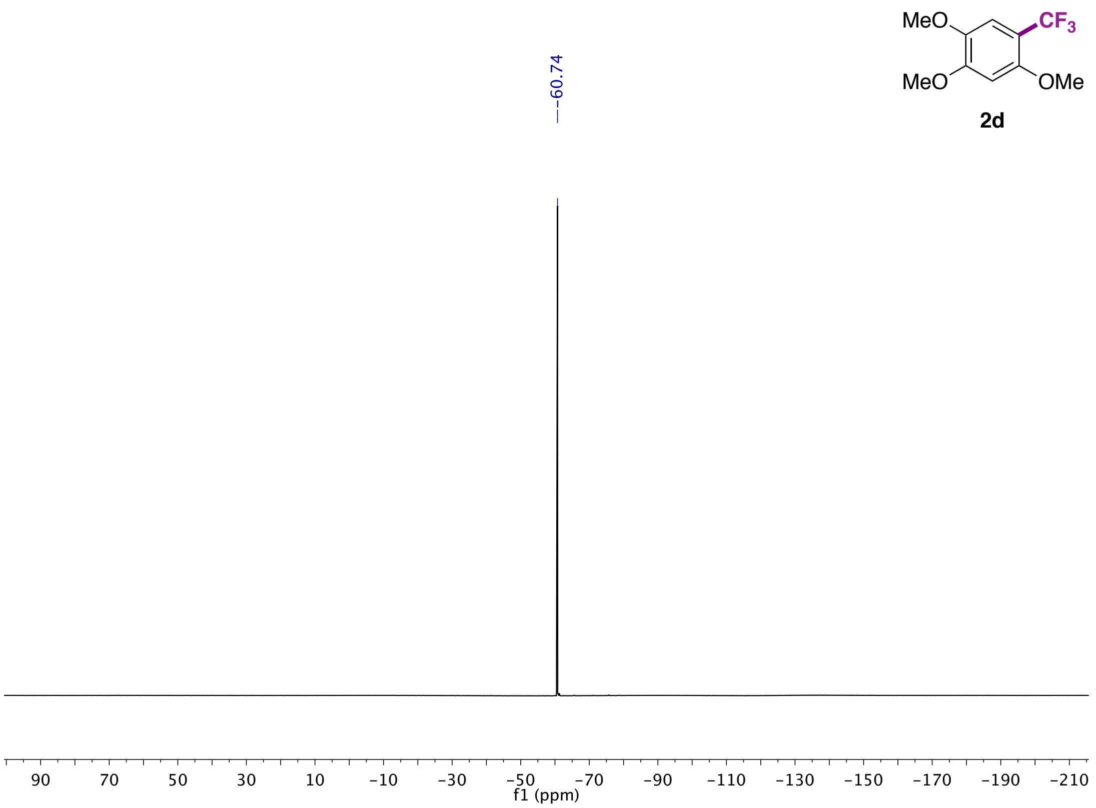

^{13}C (151 MHz, $CDCl_3$)

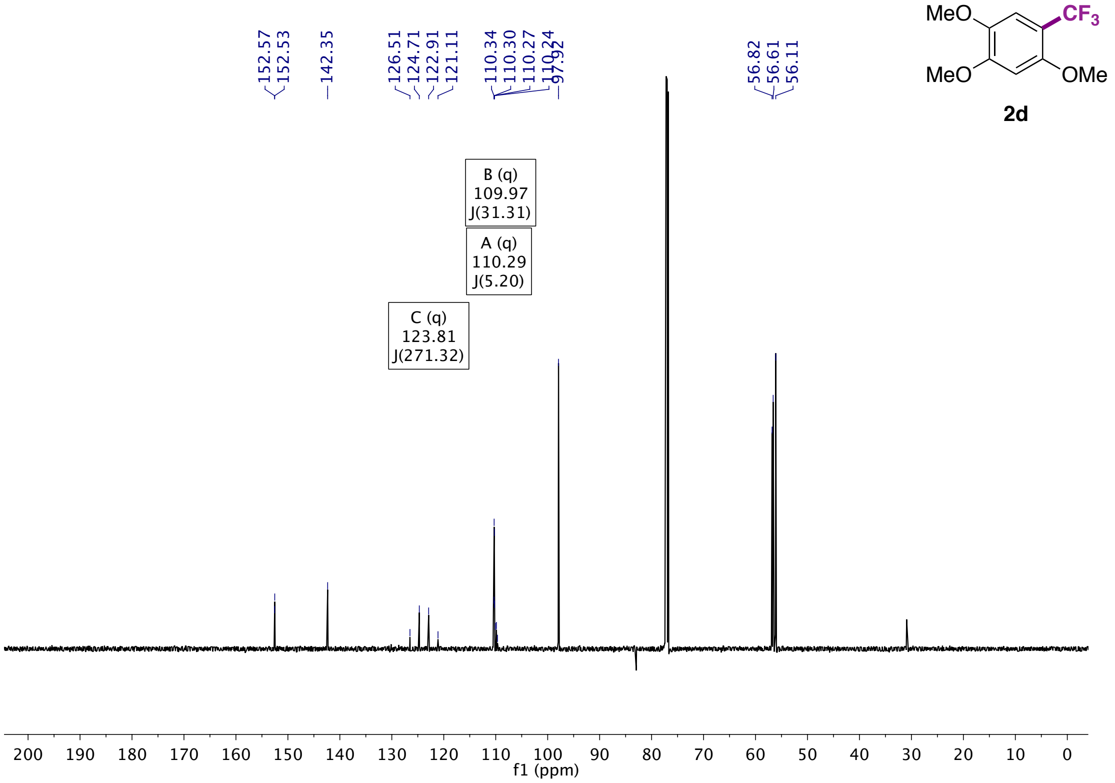

* the peak at 83.3 ppm is an artifact from the spectrometer

^{1}**H** (400 MHz, CDCl$_3$)

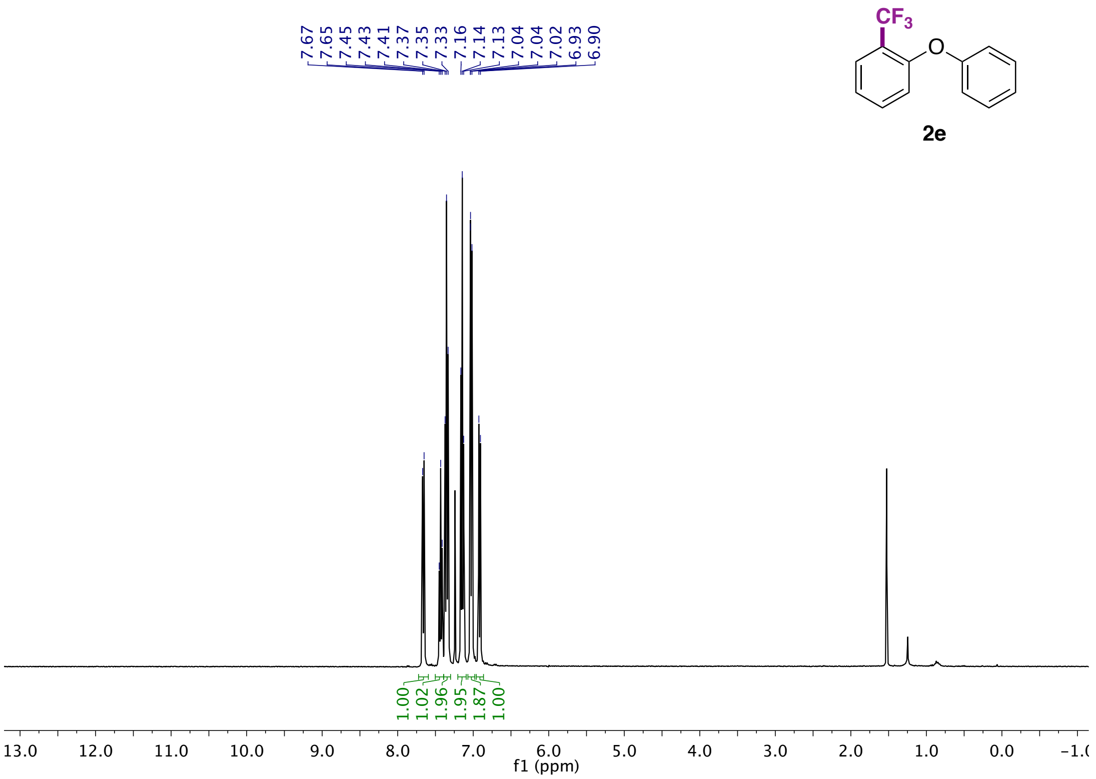

^{19}**F** (376 MHz, CDCl$_3$)

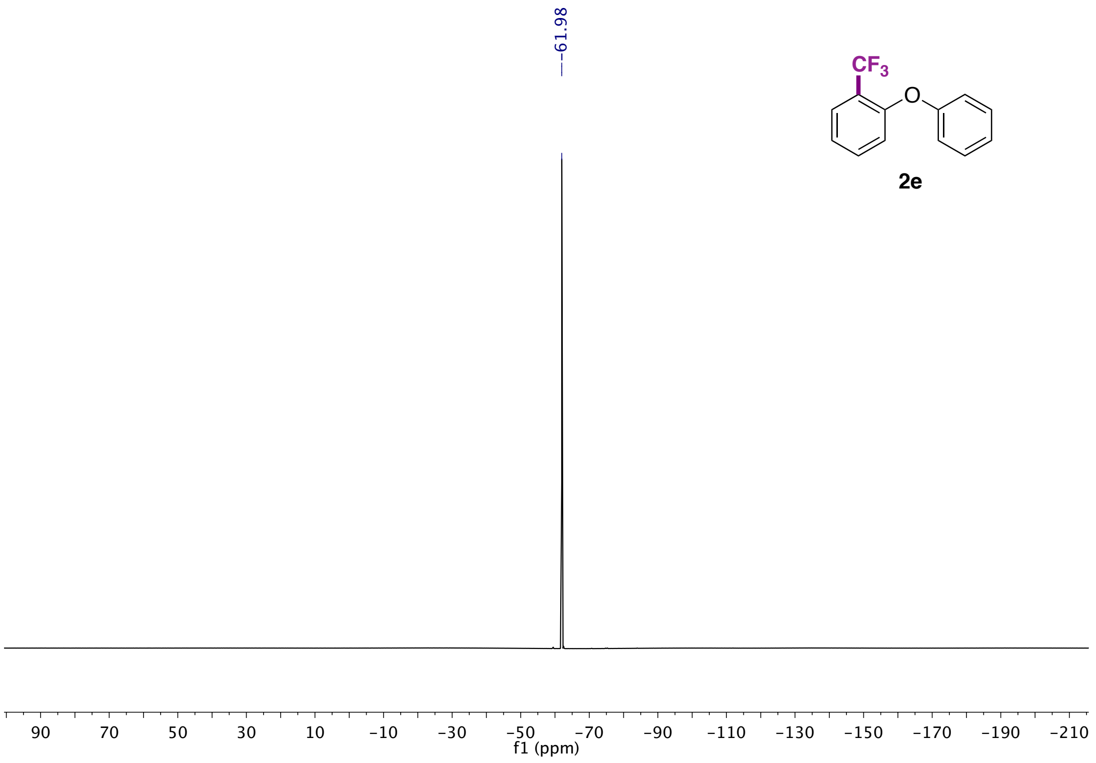

^{13}C (101 MHz, $CDCl_3$)

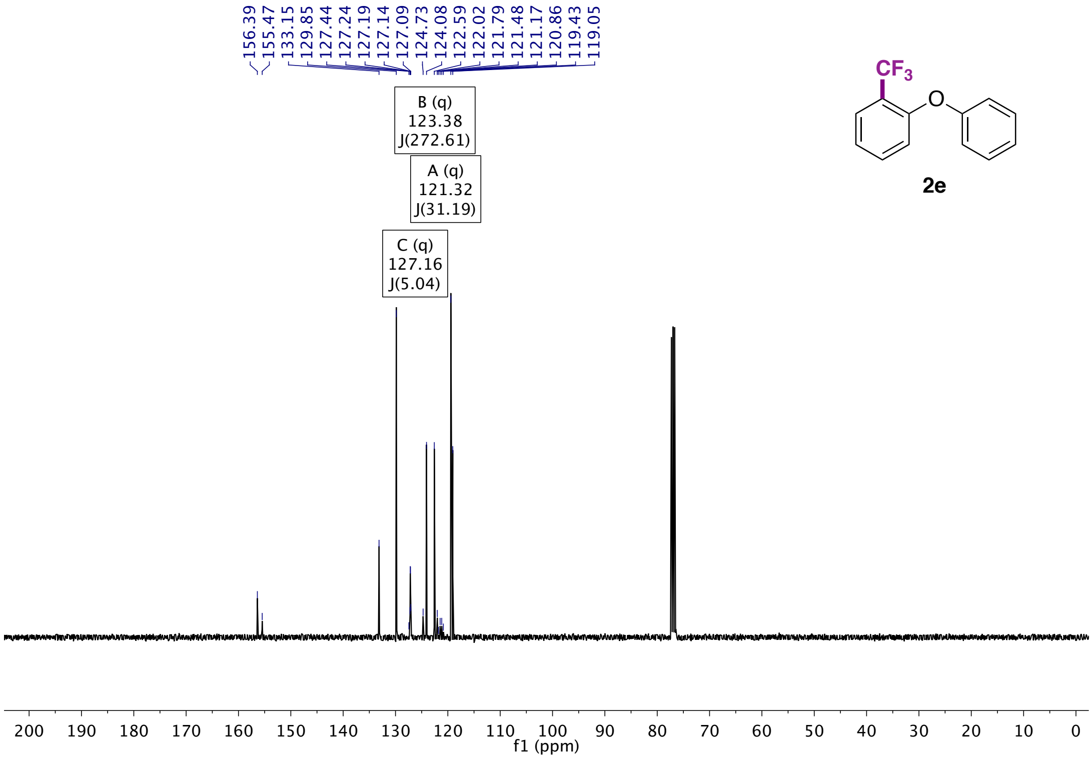

2e

^{1}H (400 MHz, $CDCl_3$)

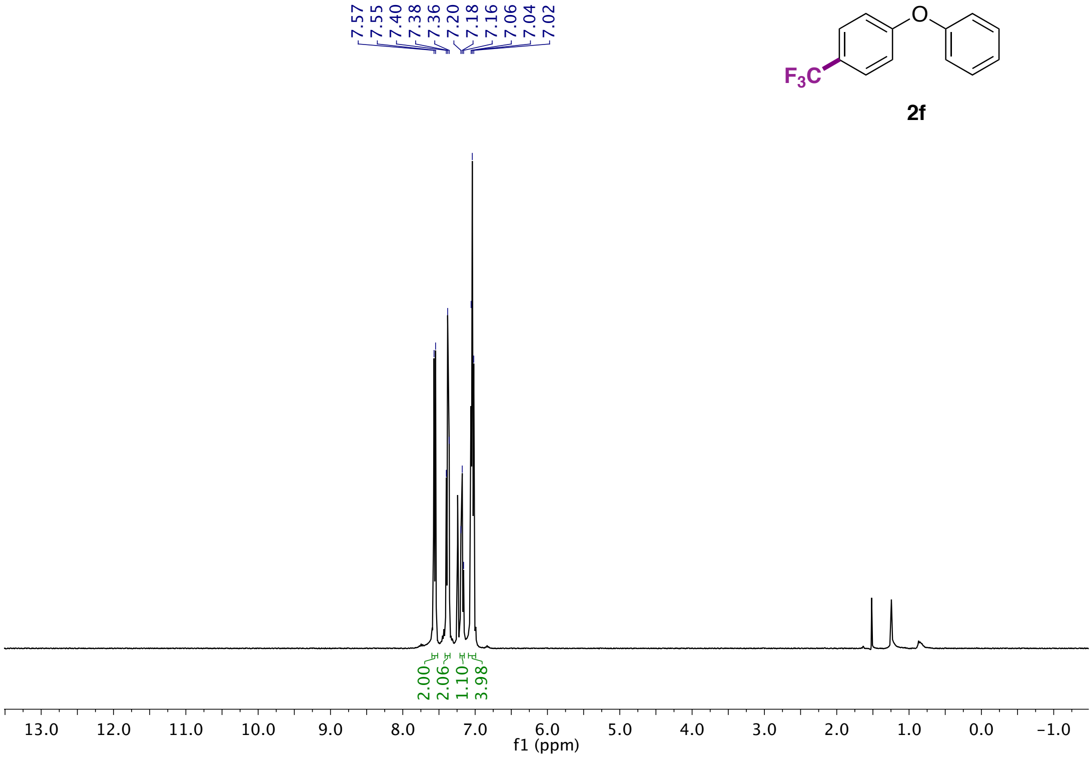

2f

^{19}F (564 MHz, $CDCl_3$)

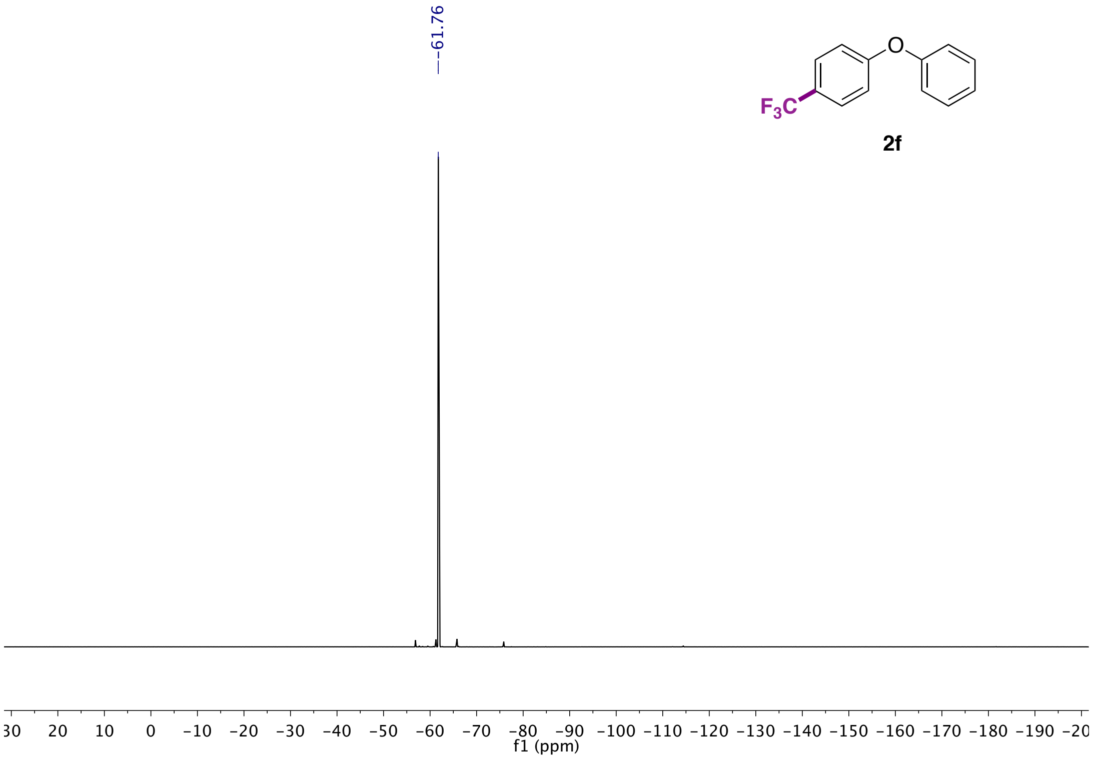

^{13}C (151 MHz, $CDCl_3$)

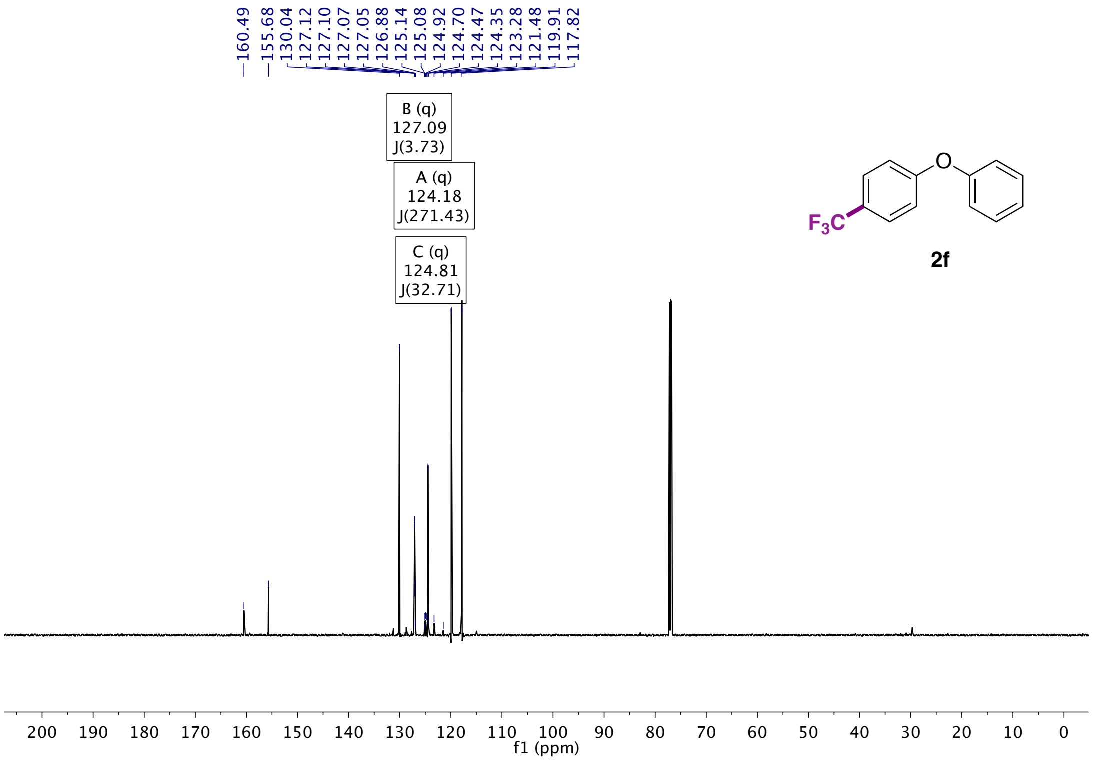

^{1}H (600 MHz, $CDCl_3$)

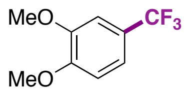

2h

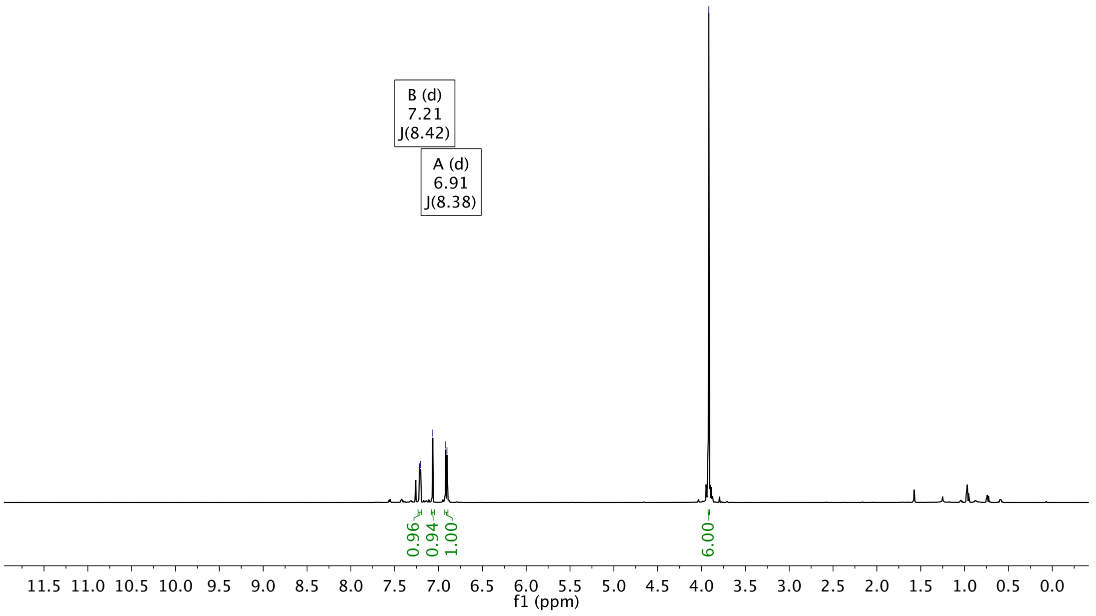

^{19}F (564 MHz, $CDCl_3$)

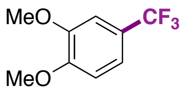

2h

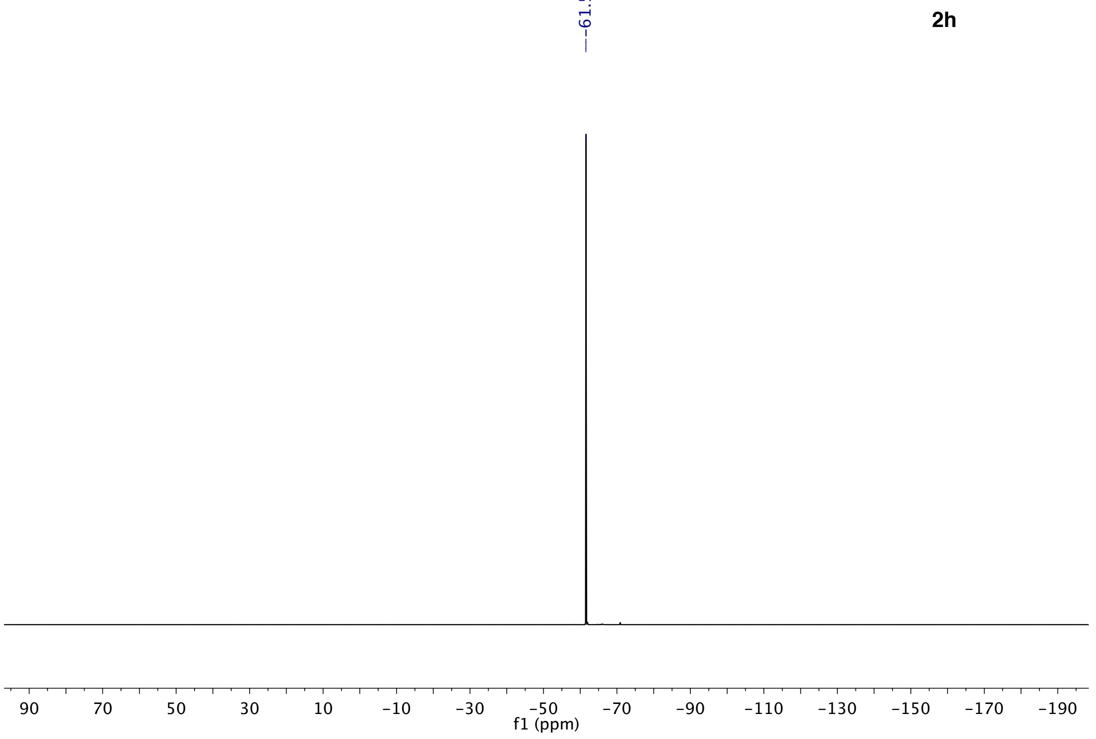

^{13}C (151 MHz, $CDCl_3$)

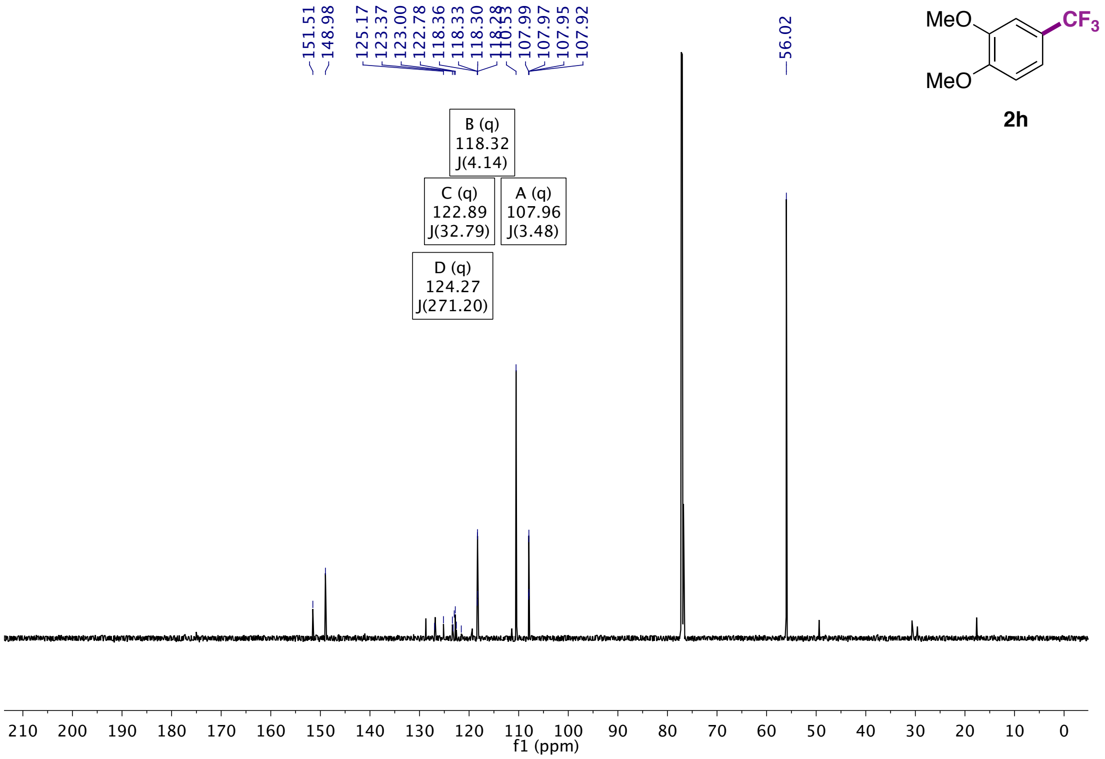

^{1}H (600 MHz, $CDCl_3$)

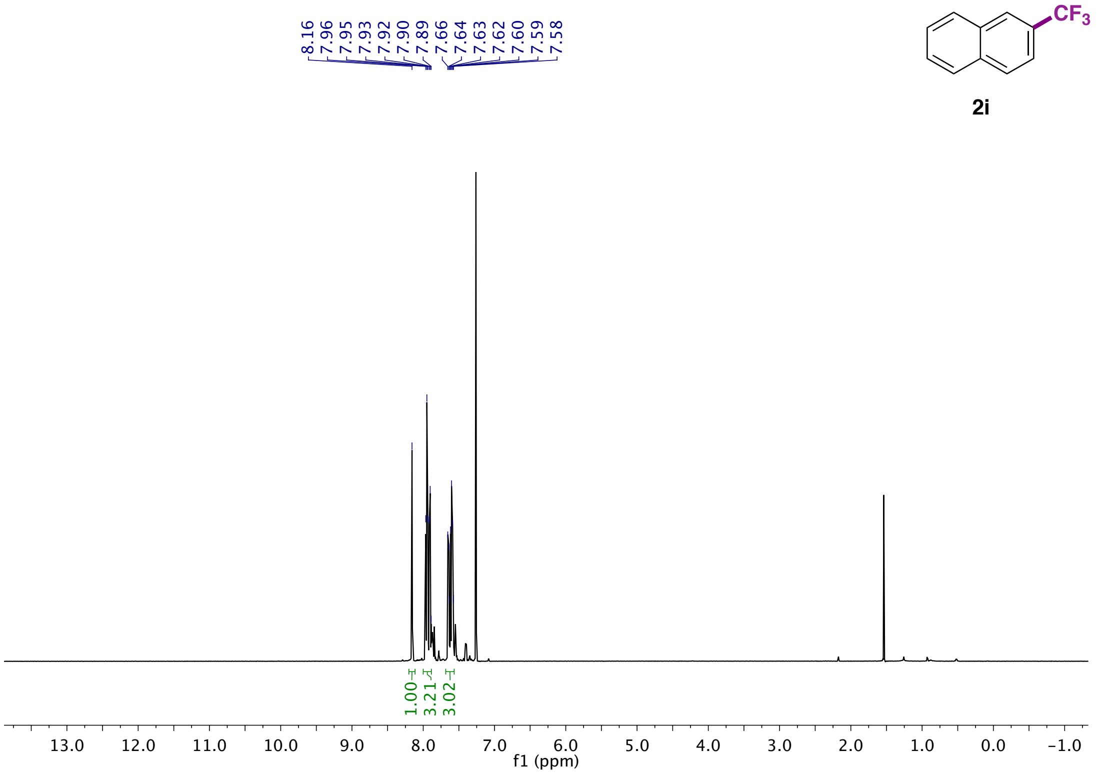

^{19}**F** (564 MHz, $CDCl_3$)

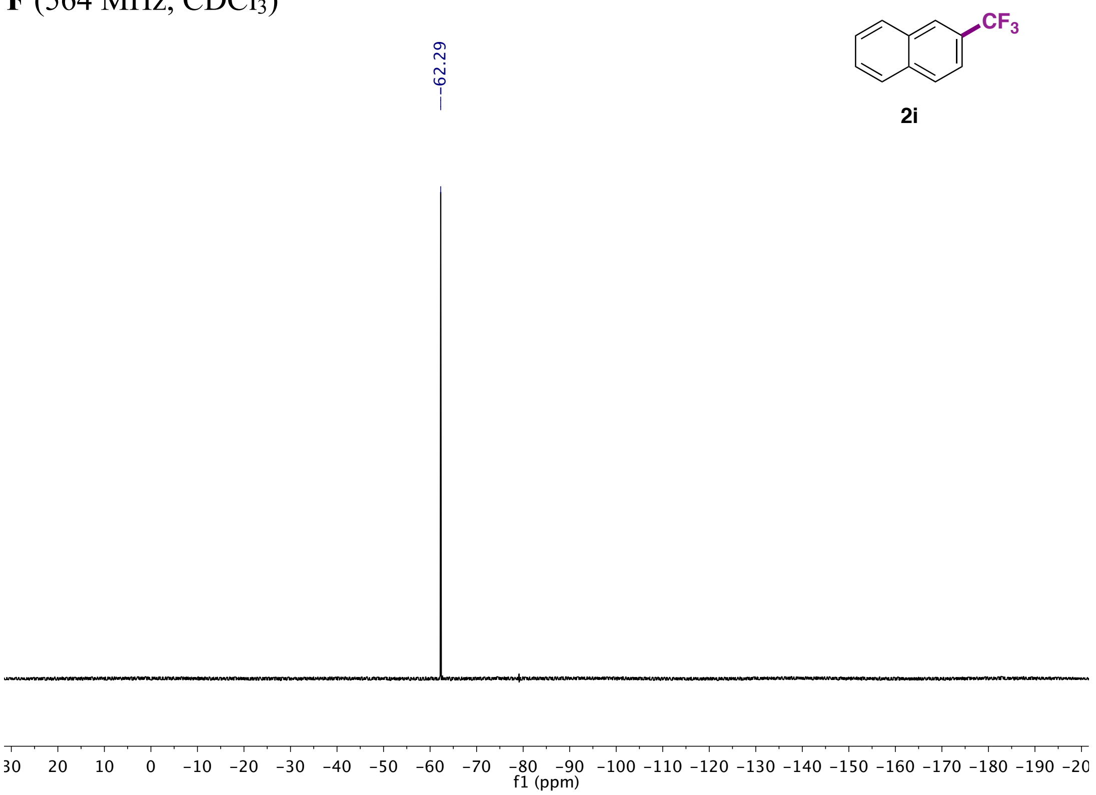

2i

^{13}**C** (151 MHz, $CDCl_3$)

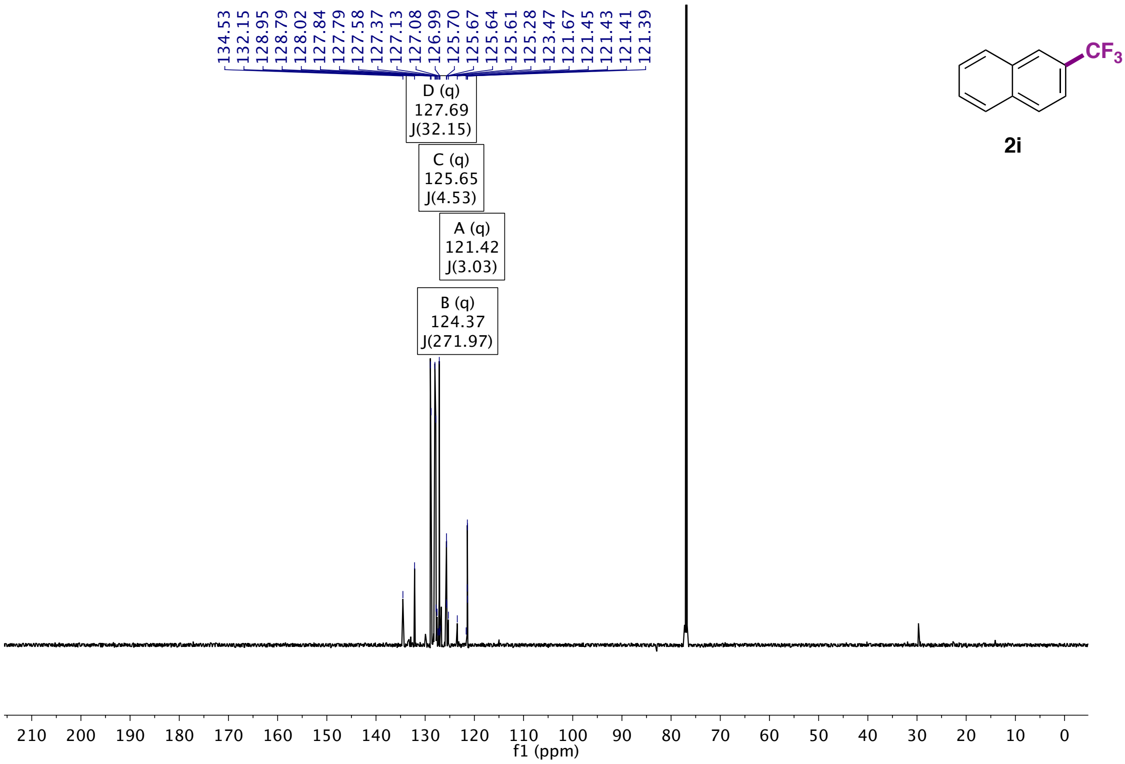

2i

¹H (600 MHz, $CDCl_3$)

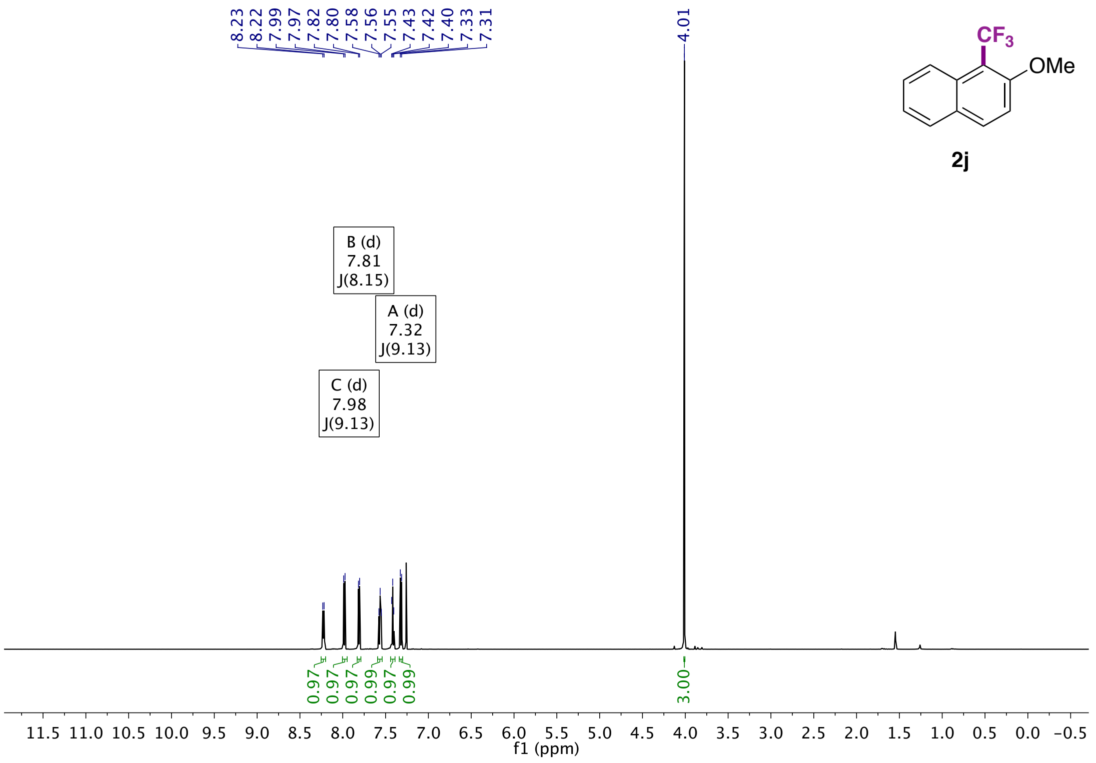

¹⁹F (564 MHz, $CDCl_3$)

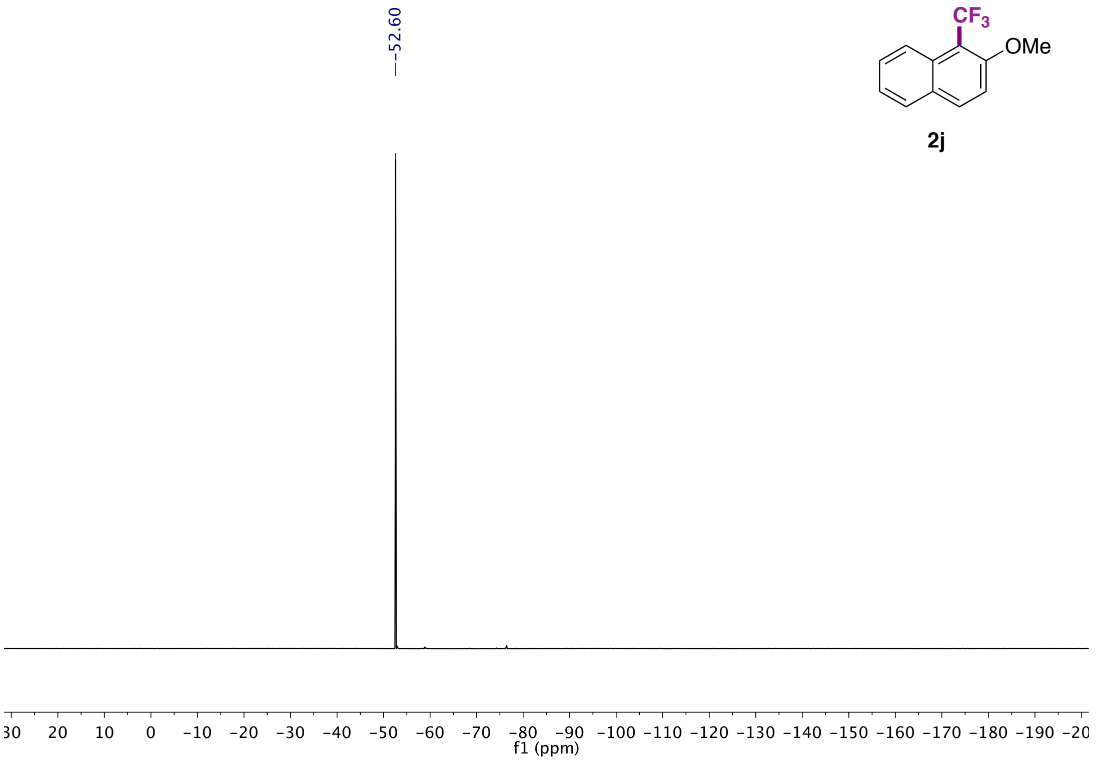

^{13}C (151 MHz, $CDCl_3$)

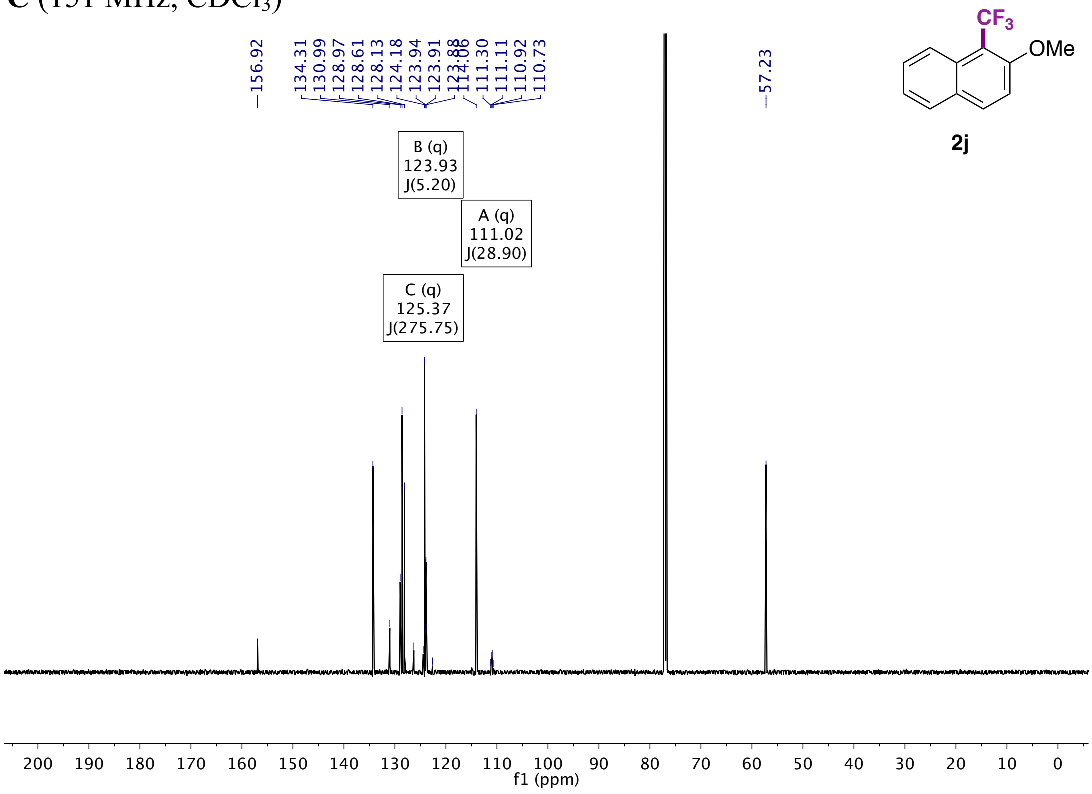

2j

^{1}H (600 MHz, $CDCl_3$)

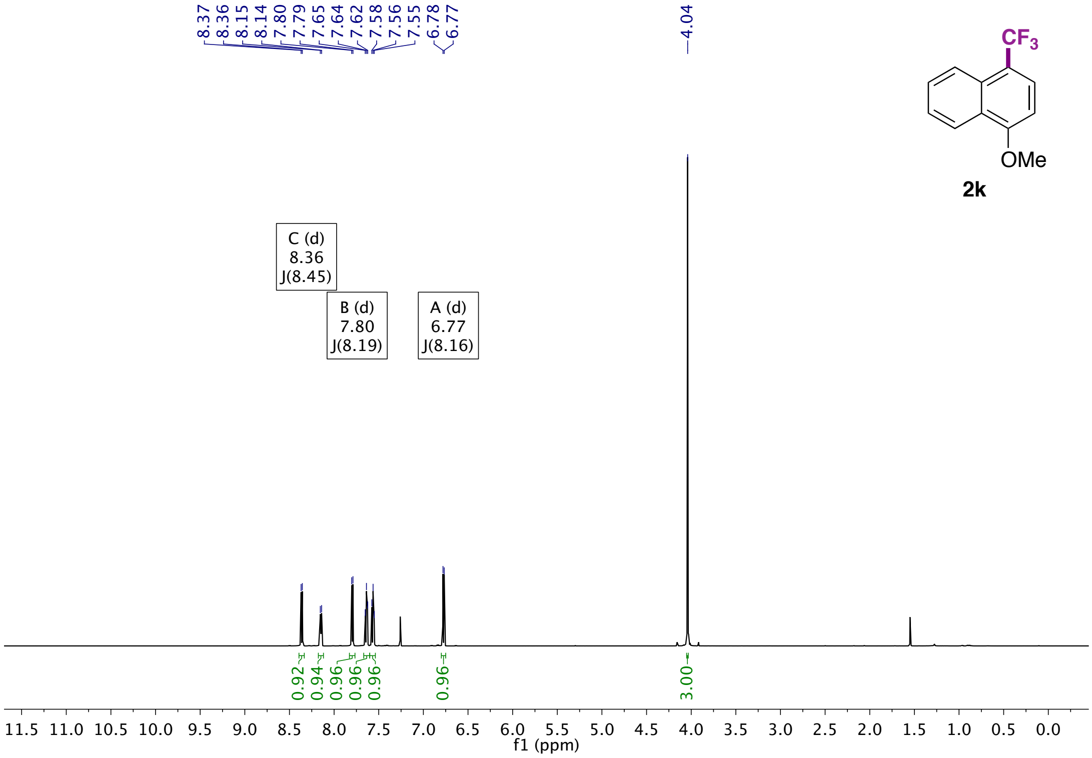

2k

^{19}F (564 MHz, $CDCl_3$)

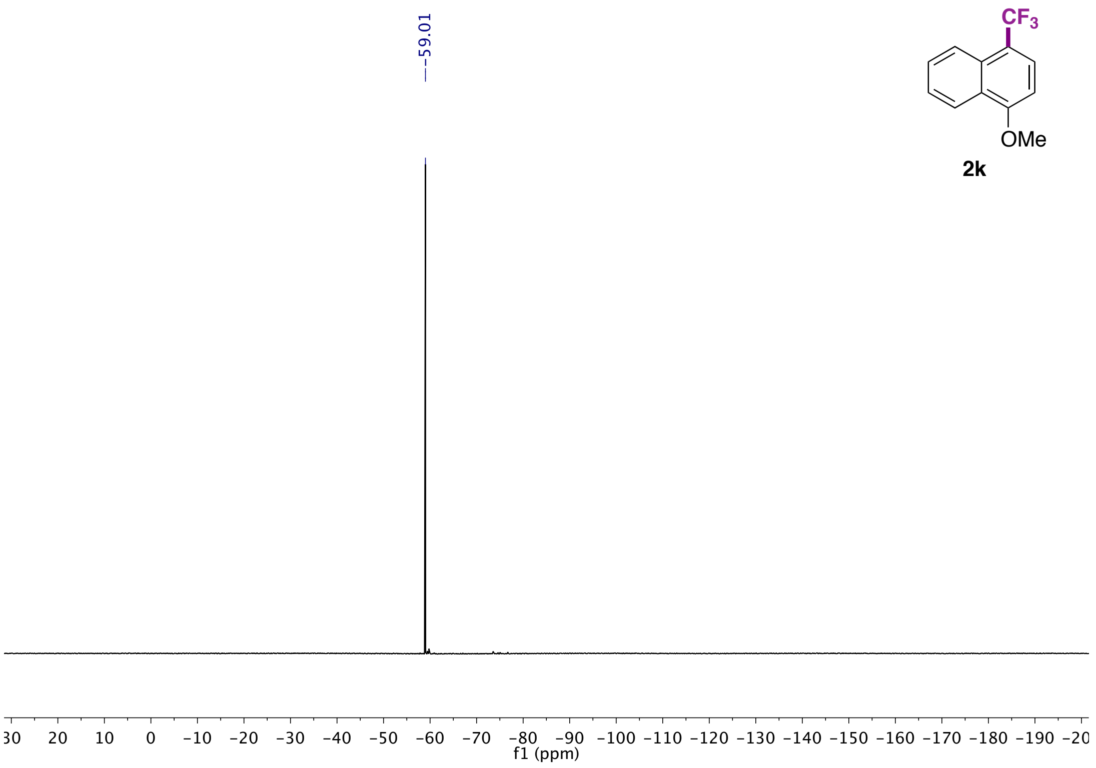

^{13}C (151 MHz, $CDCl_3$)

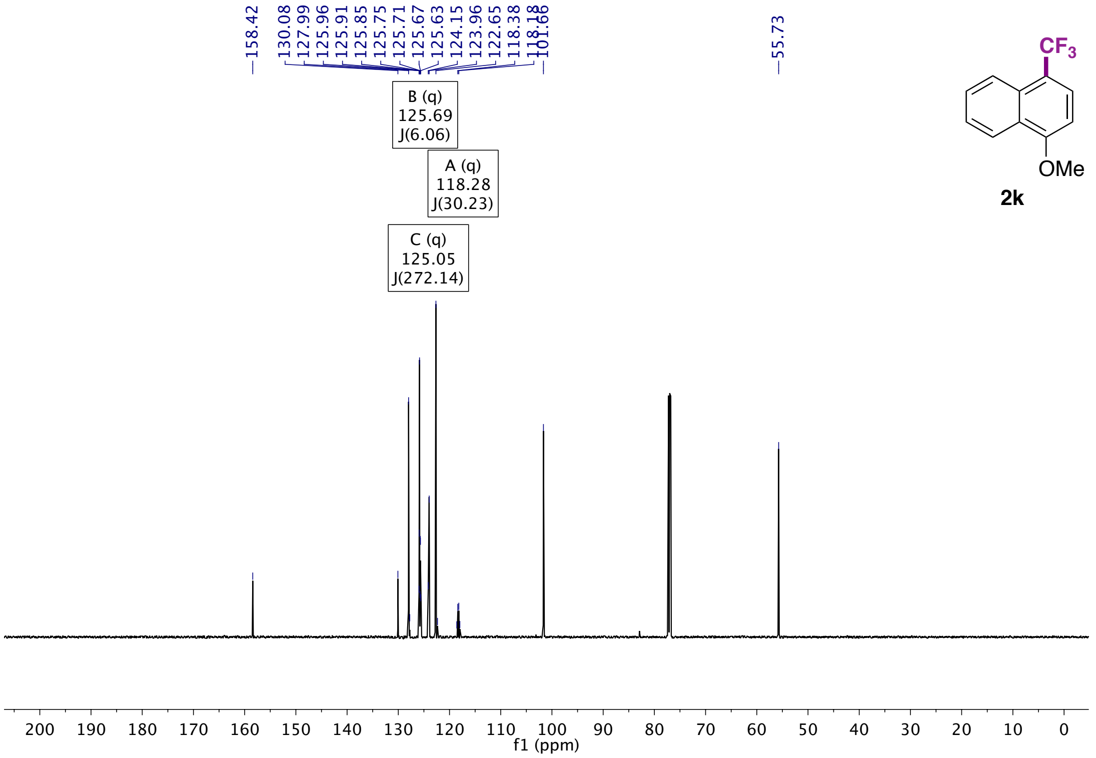

* the peak at 83.3 ppm is an artifact from the spectrometer

^{1}**H** (400 MHz, $CDCl_3$)

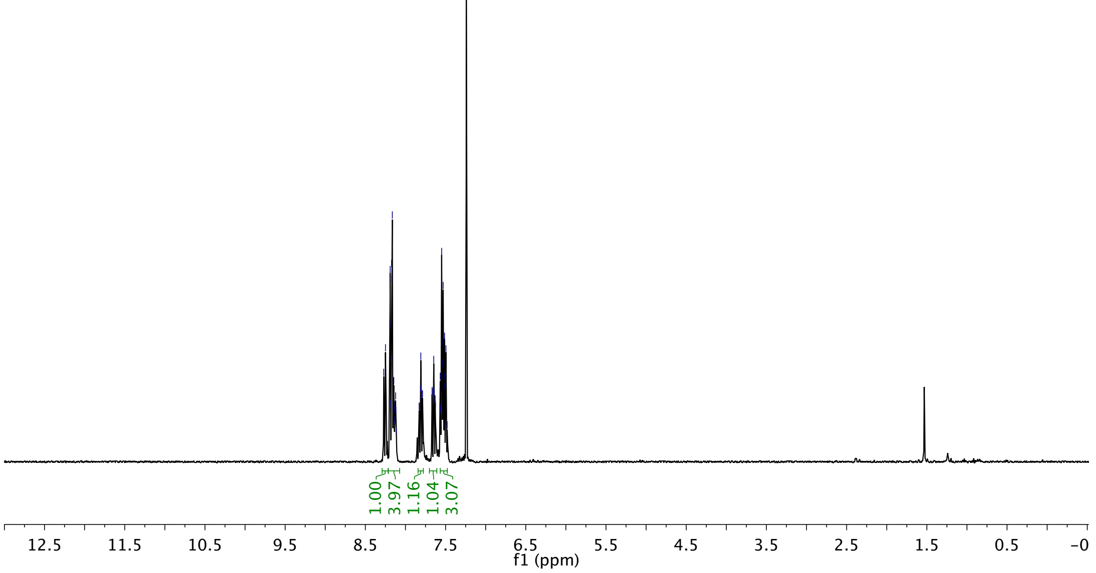

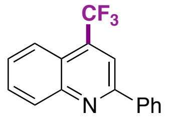

2l

^{19}**F** (376 MHz, $CDCl_3$)

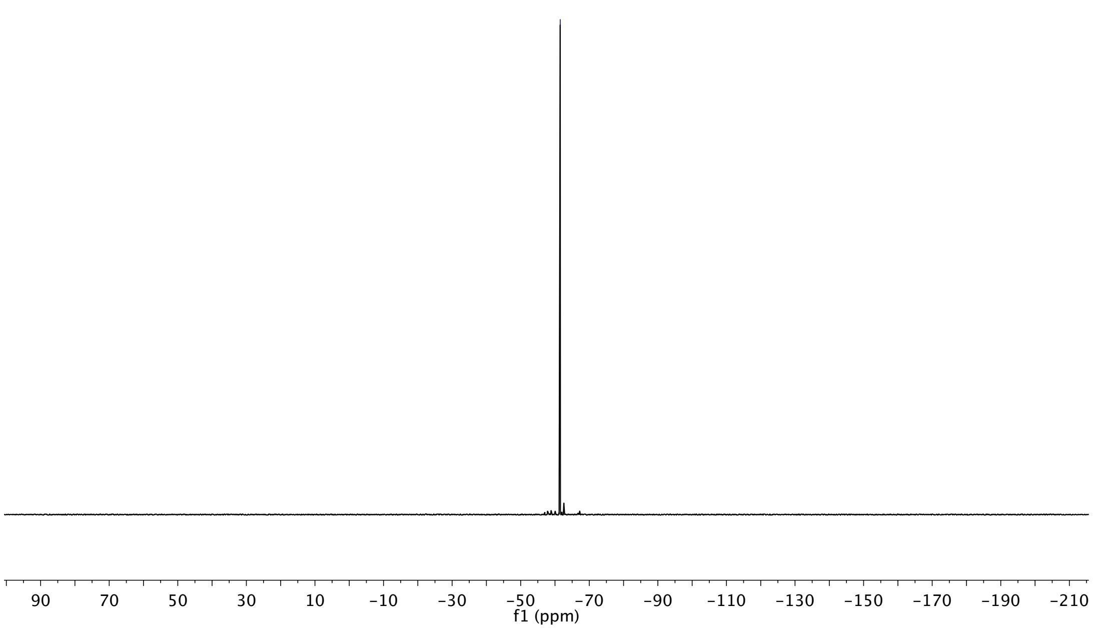

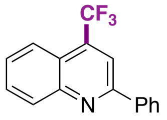

2l

^{13}**C** (151 MHz, $CDCl_3$)

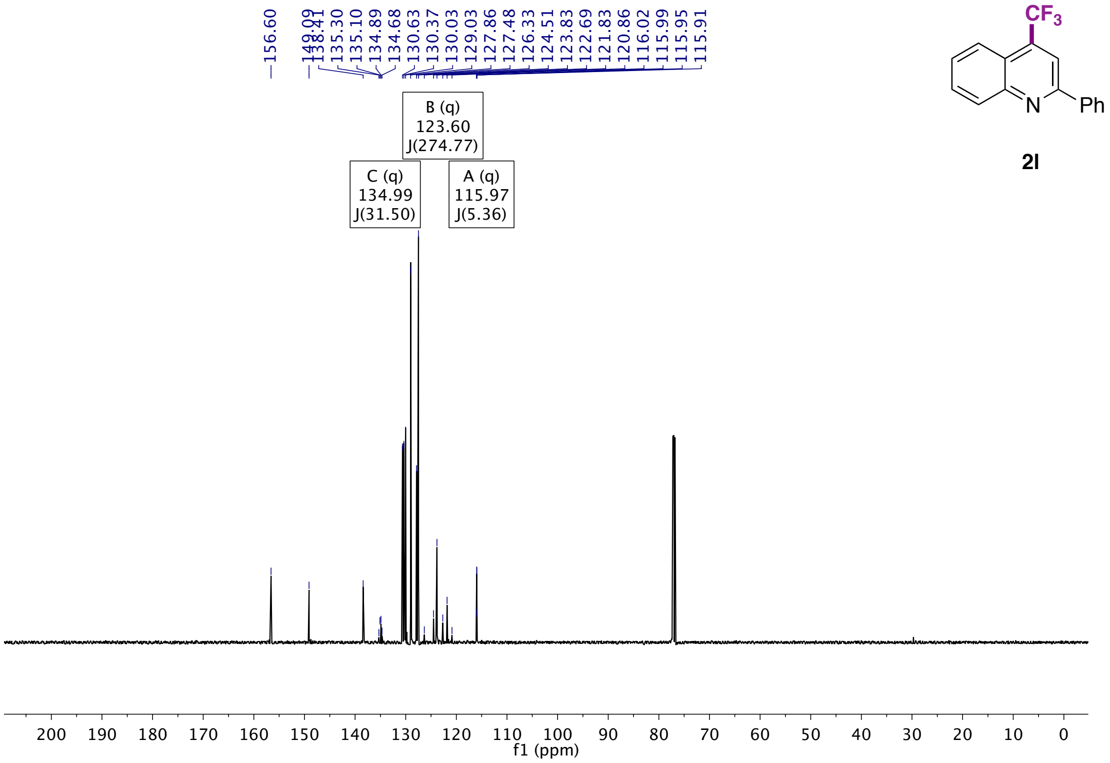

2l

^{1}**H** (400 MHz, $CDCl_3$)

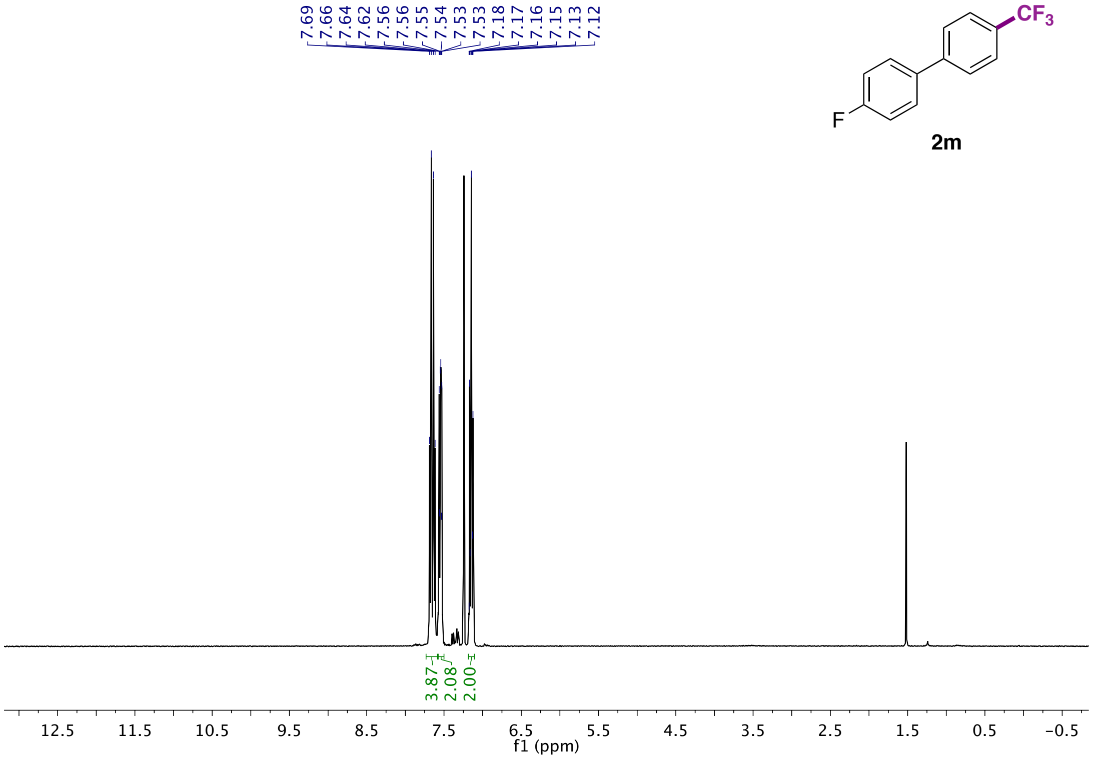

2m

^{19}F (564 MHz, $CDCl_3$)

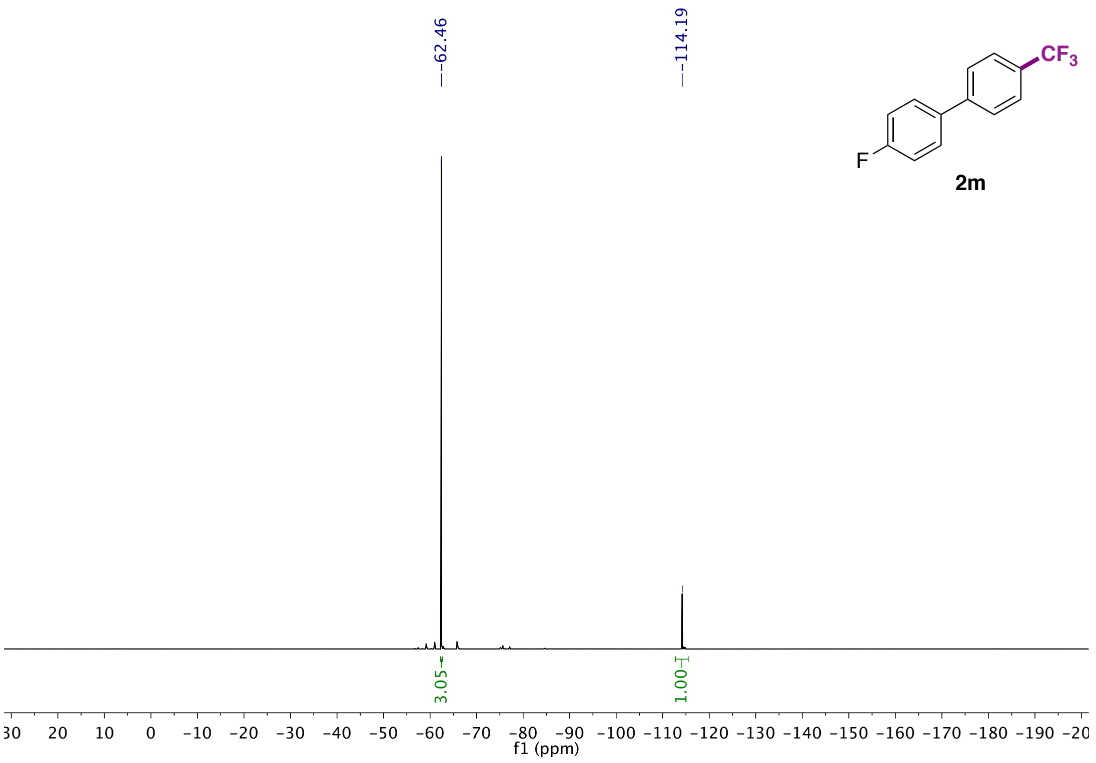

^{13}C (151 MHz, $CDCl_3$)

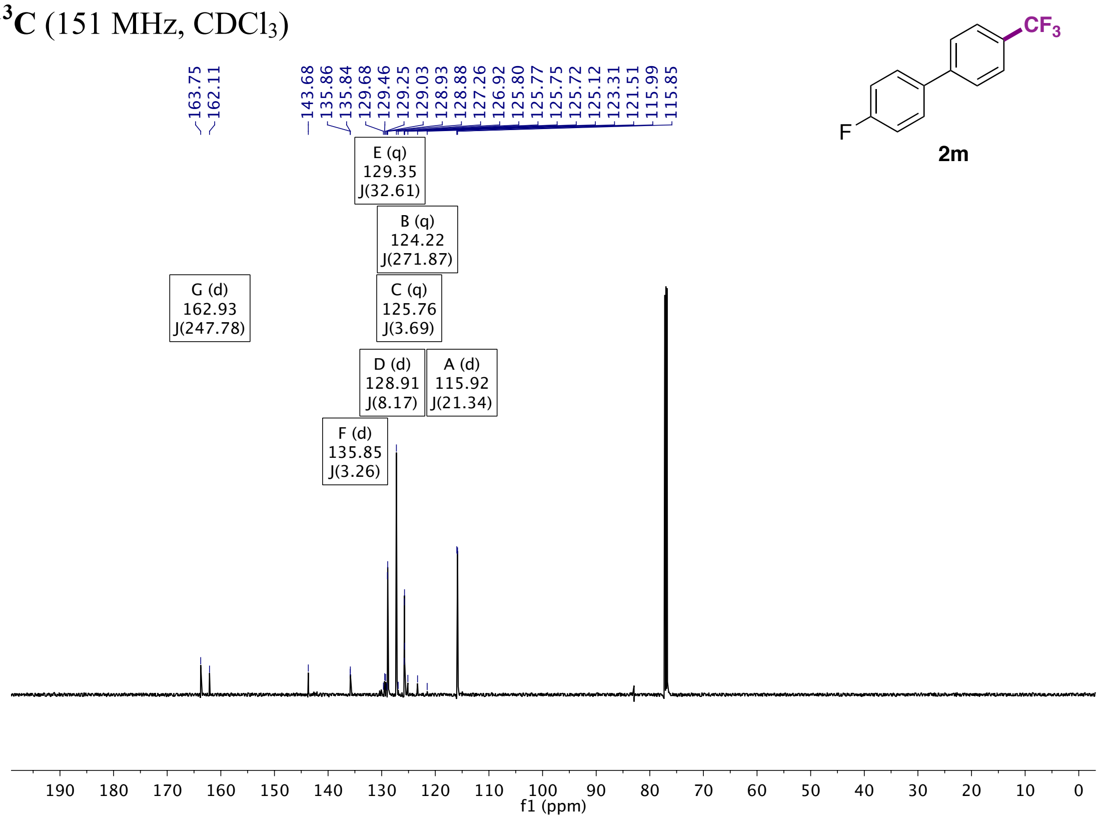

* the peak at 83.3 ppm is an artifact from the spectrometer

^{1}H (400 MHz, $CDCl_3$)

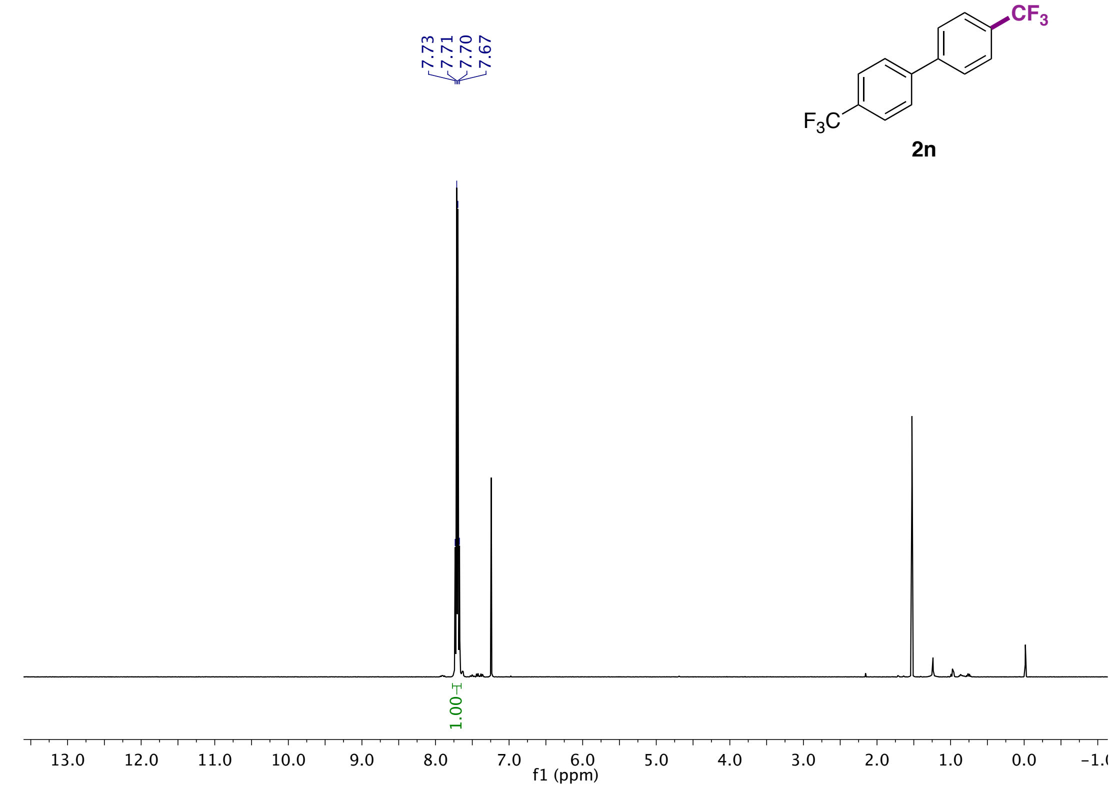

^{19}F (564 MHz, $CDCl_3$)

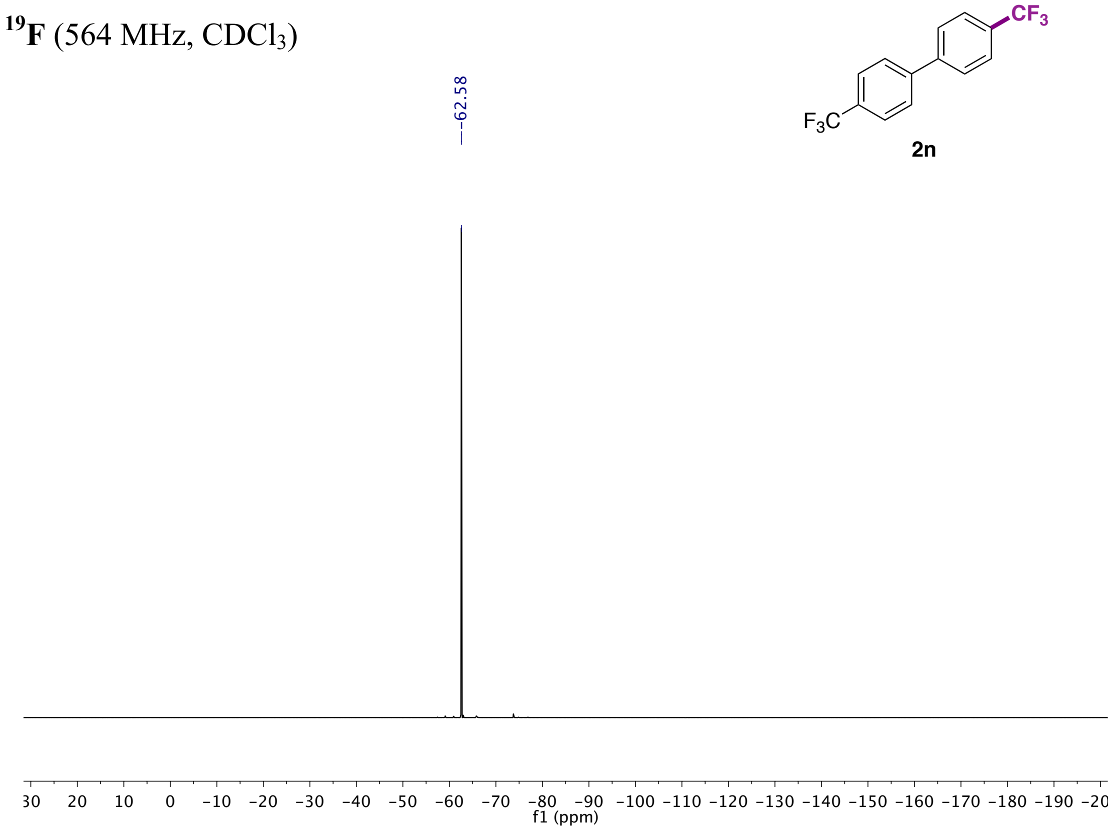

^{13}C (151 MHz, $CDCl_3$)

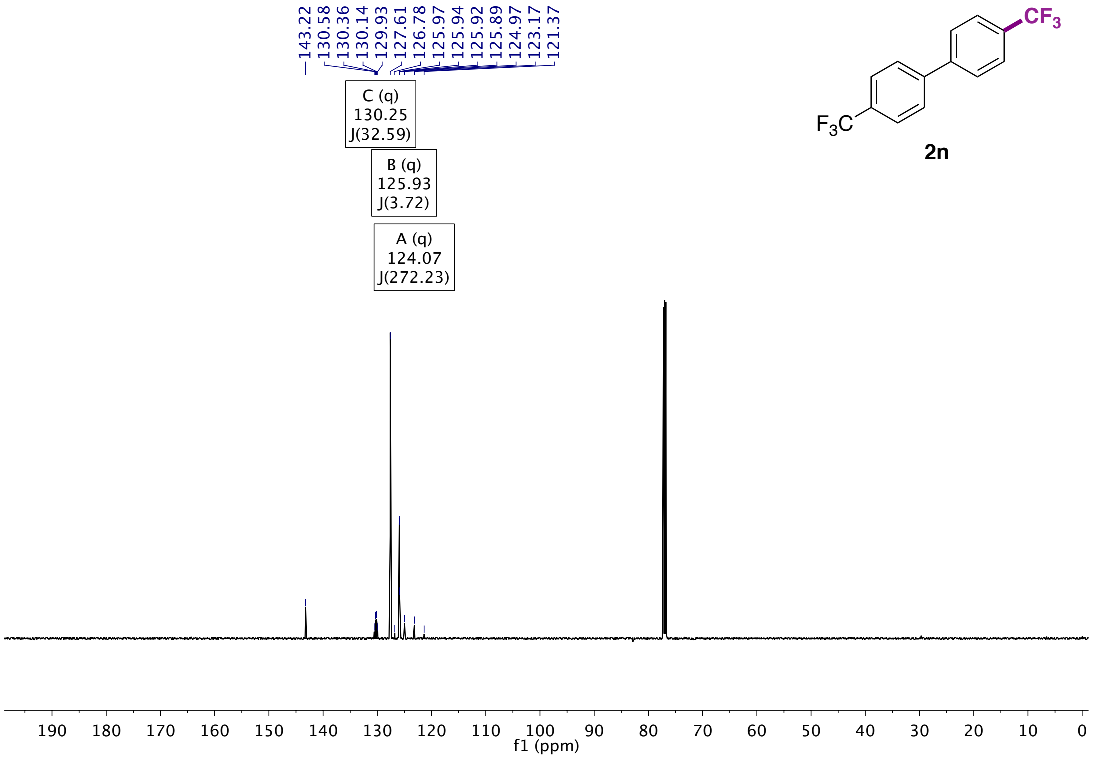

* the peak at 83.3 ppm is an artifact from the spectrometer

^{1}H (600 MHz, $CDCl_3$)

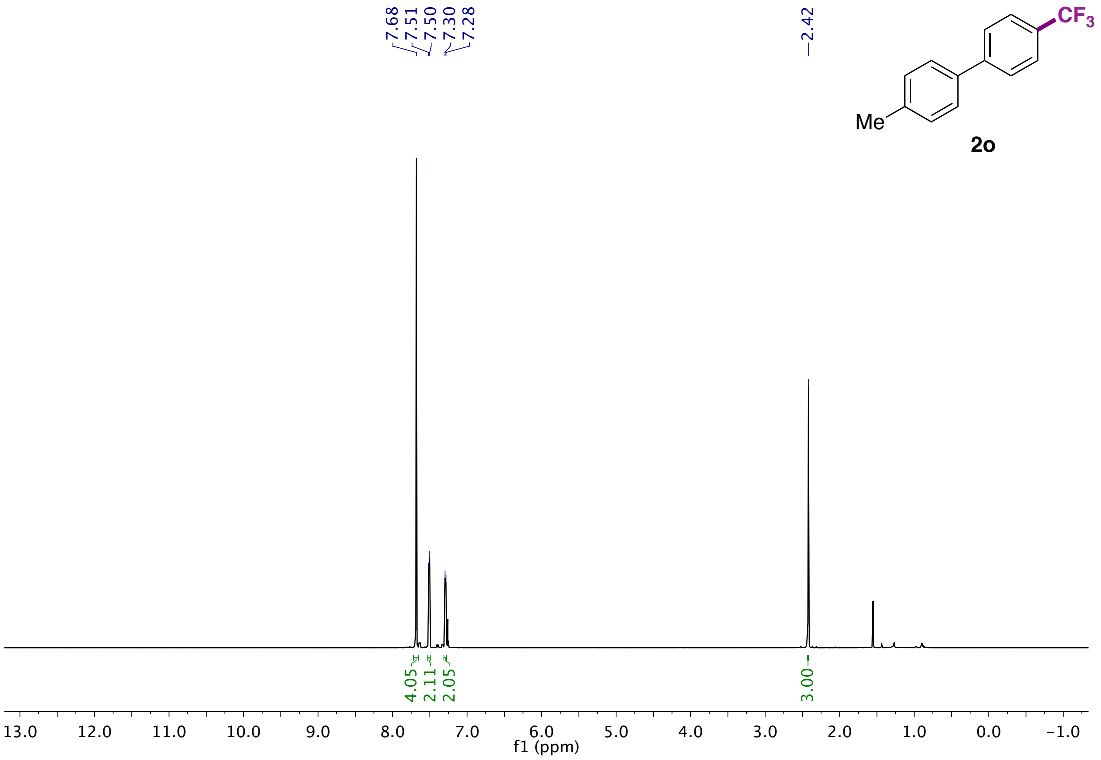

^{19}F (564 MHz, CDCl$_3$)

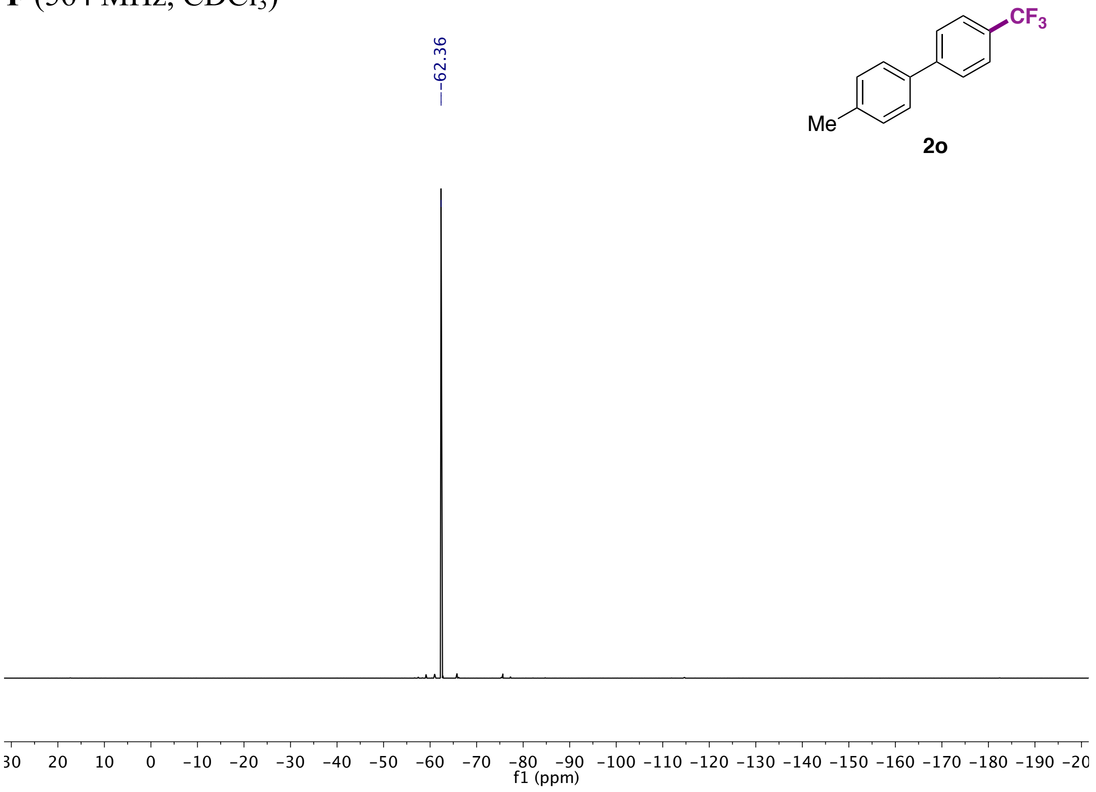

^{13}C (151 MHz, CDCl$_3$)

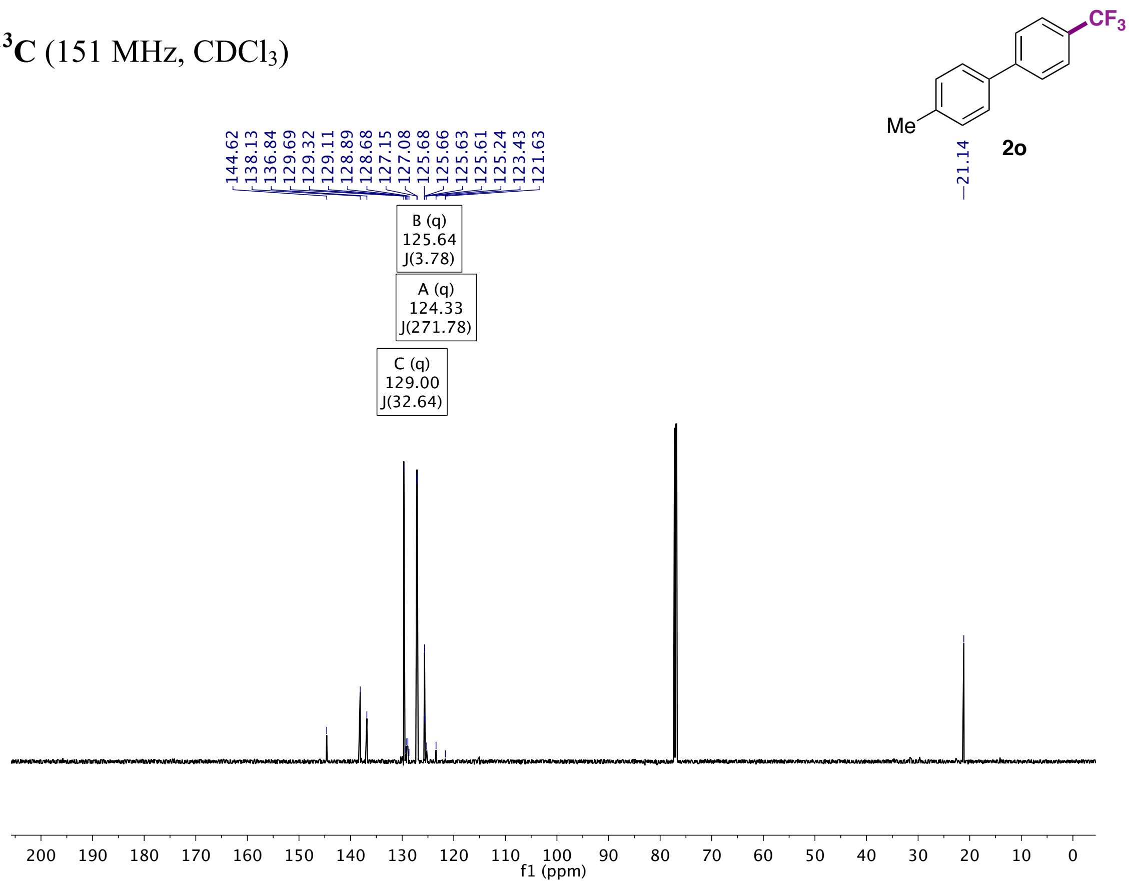

References

(1) Yin, G.; Kalvet, I.; Schoenebeck, F. *Angew. Chem. Int. Ed.* **2015**, *54*, 6809.

(2) Scattolin, T.; Deckers, K.; Schoenebeck, F. *Org. Lett.* **2017,** *19,* 5740.

(3) Beaulieu, F.; Beauregard, L.-P.; Courchesne, G.; Couturier, M.; LaFlamme, F.; L'Heureux, A. *Org. Lett.* **2009**, *11*, 5050.

(4) Mizuta, S.; Stenhagen, I. S. R.; O'Duill, M.; Wolstenhulme, J.; Kirjavainen, A. K.; Forsback, S. J.; Tredwell, M.; Sandford, G.; Moore, P. R.; Huiban, M.; Luthra, S. K.; Passchier, J.; Solin, O.; Gouverneur, V. *Org. Lett.* **2013**, *15*, 2648.

(5) Birrell, J. A.; Desrosiers, J.-N.; Jacobsen, E. N. *J. Am. Chem. Soc.* **2011**, *133*, 13872.

(6) Saito, K.; Umi, T.; Yamada, T.; Suga, T.; Akiyama, T. *Org. Biomol. Chem.* **2017**, *15*, 1767.

(7) Shrestha, B.; Thapa, S.; Gurung, S. K.; Pike, R. A. S.; Giri, R. *J. Org. Chem.* **2016**, *81*, 787.

(8) Wu, X.; Chu, L.; Qing, F.-L. *Tetrahedron Lett.* **2013**, *54*, 249.

(9) Senecal, T. D.; Parsons, A. T.; Buchwald, S. L. *J. Org. Chem.* **2011**, *76*, 1174.

(10) Yang, Y.-D.; Iwamoto, K.; Tokunaga, E.; Shibata, N. *Chem. Commun.* **2013**, *49*, 5510.

(11) Yang, J.-Y.; Xu, X.-H.; Qing, F.-L. *J. Flourine Chem.* **2016**, *186*, 45.

(12) Jiang, B.; Si, Y.-G. *J. Org. Chem.* **2002**, *67*, 9449.

(13) Sloop, J. C.; Bumgardner, C. L.; Loehle, W. D. *J. Flourine Chem.* **2002**, *118*, 135.

(14) Shi, S.; Meng, G.; Szostak, M. *Angew. Chem. Int. Ed.* **2016**, *55*, 6959.

(15) Bajo, S.; Laidlaw, G.; Kennedy, A. R.; Sproules, S.; Nelson, D. J. *Organometallics* **2017**, *36*, 1662.

(16) Luan, Y.-X.; Zhang, T.; Yao, W.-W.; Lu, K.; Kong, L.-Y.; Lin, Y.-T.; Ye, M. *J. Am. Chem. Soc.* **2017**, *139*, 1786.

(17) Frisch, M. J.; Trucks, G. W.; Schlegel, H. B.; Scuseria, G. E. R., M. A.;; Cheeseman, J. R. S., G.; Barone, V.; Mennucci, B.; Petersson,; G. A.; Nakatsuji, H. C., M.; Li, X.; Hratchian, H. P.; Izmaylov,; A. F.; Bloino, J. Z., G.; Sonnenberg, J. L.; Hada, M.; Ehara, M.;; Toyota, K. F., R.; Hasegawa, J.; Ishida, M.; Nakajima, T.; Honda,; Y.; Kitao, O. N., H.; Vreven, T.; Montgomery, J. A., Jr.; Peralta, J.; E.; Ogliaro, F. B., M.; Heyd, J. J.; Brothers, E.; Kudin, K. N.;; Staroverov, V. N. K., R.; Normand, J.; Raghavachari, K.;; Rendell, A. B., J. C.; Iyengar, S. S.; Tomasi, J.; Cossi, M.; Rega,; N.; Millam, J. M. K., M.; Knox, J. E.; Cross, J. B.; Bakken, V.;; Adamo, C. J., J.; Gomperts, R.; Stratmann, R. E.; Yazyev, O.;; Austin, A. J. C., R.; Pomelli, C.; Ochterski, J. W.; Martin, R. L.;; Morokuma, K. Z., V. G.; Voth, G. A.; Salvador, P.;; Dannenberg, J. J. D., S.; Daniels, A. D.; Farkas, Ö.;; Foresman, J. B. O., J. V.; Cioslowski, J.; Fox, D. J. *Gaussian 09, Revision D.01*; Gaussian, Inc.: Wallingford, CT, 2013.

(18) It has been shown that mixtures of Xantphos and Pd_2dba_3 contain both (dba)Pd(Xantphos) and $Pd(Xantphos)_2$, with $Pd(Xantphos)_2$ being more dominant at higher L:Pd ratios (see: Klingensmith, L. M.; Strieter, E. R.; Barder, T. E.; Buchwald, S. L. *Organometallics* **2006**, *25*, 82). As $Pd(Xantphos)_2$ was also shown to be an ineffective pre-catalyst, it is reaonsable to assume that in our studies (where a L:Pd ratio of only 1.5 is used) the (cinnamyl)Pd(Xantphos) species is the active pre-catalyst.

(19) Bickelhaupt, F. M.; Houk, K. N. *Angew. Chem. Int. Ed.* **2017**, *56*, 10070.

(20) Cho, E. J.; Senecal, T. D.; Kinzel, T.; Zhang, Y.; Watson, D. A.; Buchwald, S. L. *Science* **2010**, *328*, 1679.

Energetic data, thermal data and Cartesian coordinates of computed structures

General Structures

(cinnamyl)Pd$^{(II)}$(Xantphos)

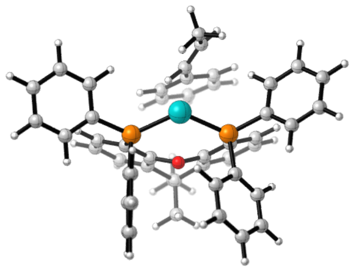

Pd	0.31180000	-0.90869000	-1.46698400
P	-1.63408600	-1.43611500	-0.34650400
P	2.31066000	-0.30716500	-0.57655000
O	0.07227000	0.32962800	1.31617800
C	3.86742500	0.07201800	-1.48108300
C	5.14184500	-0.10831500	-0.93235500
H	5.24486000	-0.51142600	0.07135700
C	6.27692800	0.22477900	-1.66507700
H	7.26157000	0.07809900	-1.23058900
C	6.15094000	0.74735700	-2.95093500
H	7.03845000	1.00705300	-3.52085400
C	4.88717500	0.92839000	-3.50614800
H	4.78357300	1.32926700	-4.51019200
C	3.75216000	0.58481000	-2.77621800
H	2.76327800	0.70677700	-3.21315500
C	2.87859100	-1.59292200	0.61243600
C	2.94532200	-1.40501200	1.99303400
H	2.70481400	-0.43876700	2.42545800
C	3.32054500	-2.45440300	2.83097600
H	3.36010400	-2.29486900	3.90457700
C	3.64761200	-3.69616900	2.29810100
H	3.94312500	-4.51051000	2.95335900
C	3.59160900	-3.89108200	0.91812700
H	3.84279400	-4.85792600	0.49140600
C	3.20081600	-2.85037700	0.08484200
H	3.13267400	-3.01361400	-0.98834800
C	2.12938300	1.19363500	0.48843600
C	3.05156600	2.24553700	0.48407400
H	3.93622600	2.17995900	-0.13985300
C	2.84581000	3.38289300	1.25730200
H	3.57090800	4.19032500	1.23004700
C	1.72550000	3.48170800	2.07625100
H	1.58399900	4.37132600	2.68086700
C	0.78865700	2.45394500	2.12120000
C	1.00083600	1.33960400	1.30669400
C	-0.43496100	2.42254100	3.03192800
C	-1.57008400	1.80184700	2.22307200
C	-2.90512200	2.18854500	2.29031500
H	-3.19440100	3.04249800	2.89315000
C	-3.87953400	1.48406900	1.59188700
H	-4.91963400	1.78797600	1.66029300
C	-3.53022900	0.39023000	0.80894000
H	-4.30342600	-0.15243800	0.27528300
C	-2.19656000	-0.00900400	0.68497700
C	-1.24487500	0.71880300	1.40643800
C	-0.12039700	1.48546200	4.22546500
H	0.13545700	0.47777400	3.88466500
H	-0.99187300	1.41273000	4.88532600
H	0.72379600	1.88170900	4.80084500
C	-0.79632400	3.80396300	3.58054400
H	-1.04736200	4.50291800	2.77773500
H	0.03261000	4.21562700	4.16482200
H	-1.65064500	3.73194300	4.26080700
C	-3.19800000	-1.94120200	-1.17188200
C	-3.73426400	-1.05798400	-2.12002800
H	-3.25564700	-0.09395600	-2.27847600
C	-4.87093900	-1.39614700	-2.84336500
H	-5.27731100	-0.69585500	-3.56782000
C	-5.48091200	-2.63459200	-2.64549200
H	-6.36572200	-2.90467000	-3.21461100
C	-4.94847300	-3.52284500	-1.71747300
H	-5.41645900	-4.49035000	-1.55976400
C	-3.81522100	-3.17863600	-0.98077700
H	-3.41434700	-3.88091800	-0.25637900
C	-1.39995000	-2.69992400	0.97079400
C	-2.31553800	-2.85350400	2.01870400
H	-3.20601900	-2.23100500	2.05346700
C	-2.08954100	-3.79165000	3.01952000
H	-2.80776300	-3.90337400	3.82684300
C	-0.93906300	-4.57944600	2.99072300
H	-0.75874900	-5.30567800	3.77837000
C	-0.02058000	-4.42850900	1.95710200
H	0.88802200	-5.02236000	1.93602500
C	-0.25228900	-3.49413200	0.95008600
H	0.47318300	-3.35991500	0.15271800
C	-0.45580600	3.87291100	-0.73042900
C	-1.41906700	3.10969900	-1.40079400
C	-2.77050400	3.41281700	-1.19628400
C	-3.14599100	4.47324600	-0.38023900
C	-2.17700500	5.24888000	0.25213700
C	-0.83006100	4.94021800	0.07760400
H	0.59748500	3.62981700	-0.85054400
H	-3.53212100	2.79251400	-1.66021200
H	-4.20007200	4.68614800	-0.22628400
H	-2.47058800	6.07943100	0.88822400
H	-0.06394200	5.52548700	0.57853600
C	-0.98390300	2.02224300	-2.29272700
C	-1.61947700	1.60644900	-3.39318900
H	-0.04311700	1.53536600	-2.02160300
H	-2.53899400	2.10653800	-3.70188100
C	-1.14852000	0.47836600	-4.26184300
H	-0.94729800	0.81839900	-5.28548600
H	-0.23137100	0.02736100	-3.85711700
H	-1.90680500	-0.31213500	-4.32560400

E(RM06L) = -2740.84792103
E(RM06) = -2739.53144840

25 °C:
Zero-point correction= 0.771198 (Hartree/Particle)
Thermal correction to Energy= 0.817795
Thermal correction to Enthalpy= 0.818739
Thermal correction to Gibbs Free Energy= 0.688644
Sum of electronic and zero-point Energies= -2738.879285
Sum of electronic and thermal Energies= -2738.832689
Sum of electronic and thermal Enthalpies= -2738.831745
Sum of electronic and thermal Free Energies= -2738.961839

160 °C:
Zero-point correction= 0.771198 (Hartree/Particle)
Thermal correction to Energy= 0.865193
Thermal correction to Enthalpy= 0.866565
Thermal correction to Gibbs Free Energy= 0.620843
Sum of electronic and zero-point Energies= -2738.879285
Sum of electronic and thermal Energies= -2738.785290
Sum of electronic and thermal Enthalpies= -2738.783918
Sum of electronic and thermal Free Energies= -2739.029641

Cinnamyl ligand

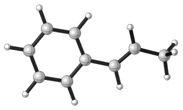

C	-0.57831	1.09305	-0.08115
C	-0.06043	-0.20929	-0.06239
C	-0.96565	-1.27562	-0.00036
C	-2.33723	-1.05431	0.06075

C	-2.83481	0.24484	0.05527
C	-1.94758	1.31691	-0.01832
H	0.09543	1.94136	-0.15918
H	-0.58406	-2.29397	0.00654
H	-3.01851	-1.89878	0.11122
H	-3.90506	0.42229	0.10215
H	-2.32663	2.33482	-0.03512
C	1.38372	-0.5008	-0.10857
C	2.38257	0.36165	0.09643
H	1.63654	-1.54175	-0.31448
H	2.15952	1.40185	0.33216
C	3.83652	0.00388	0.0386
H	4.35634	0.59004	-0.72943
H	4.33324	0.21948	0.99257
H	3.98035	-1.05725	-0.18796

E(RM06L) = -349.037903936
E(RM06) = -348.804390199

25 °C:
Zero-point correction= 0.163685 (Hartree/Particle)
Thermal correction to Energy= 0.171949
Thermal correction to Enthalpy= 0.172893
Thermal correction to Gibbs Free Energy= 0.130188
Sum of electronic and zero-point Energies= -348.680843
Sum of electronic and thermal Energies= -348.672579
Sum of electronic and thermal Enthalpies= -348.671635
Sum of electronic and thermal Free Energies= -348.714340

160 °C:
Zero-point correction= 0.163685 (Hartree/Particle)
Thermal correction to Energy= 0.180127
Thermal correction to Enthalpy= 0.181499
Thermal correction to Gibbs Free Energy= 0.109255
Sum of electronic and zero-point Energies= -348.680843
Sum of electronic and thermal Energies= -348.664401
Sum of electronic and thermal Enthalpies= -348.663029
Sum of electronic and thermal Free Energies= -348.735273

TESCF$_3$

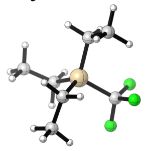

C	1.01171	2.64746	0.92021
H	0.57441	2.51785	1.91658
H	1.96778	2.11489	0.90781
H	1.21991	3.71414	0.7862
C	0.06876	2.12795	-0.17367
H	-0.87552	2.68784	-0.15107
H	0.50601	2.30866	-1.16357
Si	-0.33832	0.27632	-0.03836
C	-0.90272	-0.20323	1.71209
H	-0.07269	-0.01449	2.40413
H	-1.70749	0.48098	2.01197
C	-1.37082	-1.659	1.83882
H	-1.65559	-1.89391	2.86978
H	-2.24075	-1.85567	1.20179
H	-0.58242	-2.35779	1.54173
C	-1.59243	-0.27637	-1.35389
H	-1.66922	-1.37013	-1.32462
H	-1.19786	-0.02647	-2.34639
C	-2.97748	0.35483	-1.15895
H	-3.68267	0.00852	-1.92168
H	-3.40035	0.09988	-0.18013
H	-2.93342	1.44798	-1.22468
C	1.31216	-0.6912	-0.3915
F	1.11735	-2.02833	-0.49138
F	1.88861	-0.29828	-1.55102
F	2.23752	-0.51044	0.58134

E(RM06L) = -864.992855779
E(RM06) = -864.753925342

25 °C:
Zero-point correction= 0.216413 (Hartree/Particle)
Thermal correction to Energy= 0.231434
Thermal correction to Enthalpy= 0.232378
Thermal correction to Gibbs Free Energy= 0.173749
Sum of electronic and zero-point Energies= -864.409967
Sum of electronic and thermal Energies= -864.394946
Sum of electronic and thermal Enthalpies= -864.394002
Sum of electronic and thermal Free Energies= -864.452632

160 °C:
Zero-point correction= 0.216413 (Hartree/Particle)
Thermal correction to Energy= 0.244441
Thermal correction to Enthalpy= 0.245813
Thermal correction to Gibbs Free Energy= 0.144669
Sum of electronic and zero-point Energies= -864.409967
Sum of electronic and thermal Energies= -864.381939
Sum of electronic and thermal Enthalpies= -864.380567
Sum of electronic and thermal Free Energies= -864.481712

TESF

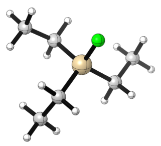

C	2.83472	0.07318	-0.669
H	2.53891	0.78713	-1.44594
H	3.1418	0.64929	0.21044
H	3.71307	-0.46816	-1.03586
C	1.68909	-0.88869	-0.32771
H	1.40415	-1.46275	-1.22037
H	2.02602	-1.62776	0.41031
Si	0.13775	-0.03505	0.33598
C	-0.42886	1.34734	-0.82442
H	0.36294	2.10401	-0.88997
H	-0.52269	0.92235	-1.8335
C	-1.74945	2.00604	-0.40581
H	-2.04054	2.79556	-1.10655
H	-2.56729	1.27682	-0.37239
H	-1.66738	2.45651	0.58903
C	-1.25937	-1.27537	0.63241
H	-2.07546	-0.77092	1.1649
H	-0.90218	-2.06384	1.30653
C	-1.79048	-1.8955	-0.66798
H	-2.59465	-2.61076	-0.46669
H	-2.1915	-1.13088	-1.34371
H	-1.00317	-2.43269	-1.20972
F	0.51952	0.65165	1.79054

E(RM06L) = -627.192175338
E(RM06) = -627.003081194

25 °C:
Zero-point correction= 0.204434 (Hartree/Particle)
Thermal correction to Energy= 0.216376
Thermal correction to Enthalpy= 0.217321
Thermal correction to Gibbs Free Energy= 0.166761
Sum of electronic and zero-point Energies= -626.746107
Sum of electronic and thermal Energies= -626.734164
Sum of electronic and thermal Enthalpies= -626.733220
Sum of electronic and thermal Free Energies= -626.783779

160 °C:
Zero-point correction= 0.204434 (Hartree/Particle)
Thermal correction to Energy= 0.226873
Thermal correction to Enthalpy= 0.228244
Thermal correction to Gibbs Free Energy= 0.141816
Sum of electronic and zero-point Energies= -626.746107
Sum of electronic and thermal Energies= -626.723668
Sum of electronic and thermal Enthalpies= -626.722297
Sum of electronic and thermal Free Energies= -626.808725

CO

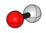

O 0.66614 0.37618 0.
C -0.59226 0.37618 0.

E(RM06L) = -113.344159015
E(RM06) = -113.301490932

25 °C:
Zero-point correction= 0.005118 (Hartree/Particle)
Thermal correction to Energy= 0.007479
Thermal correction to Enthalpy= 0.008423
Thermal correction to Gibbs Free Energy= -0.014016
Sum of electronic and zero-point Energies= -113.264561
Sum of electronic and thermal Energies= -113.262200
Sum of electronic and thermal Enthalpies= -113.261256
Sum of electronic and thermal Free Energies= -113.283695

160 °C:
Zero-point correction= 0.005118 (Hartree/Particle)
Thermal correction to Energy= 0.008553
Thermal correction to Enthalpy= 0.009925
Thermal correction to Gibbs Free Energy= -0.024473
Sum of electronic and zero-point Energies= -113.264561
Sum of electronic and thermal Energies= -113.261126
Sum of electronic and thermal Enthalpies= -113.259754
Sum of electronic and thermal Free Energies= -113.294152

ArCOF

Benzoyl fluoride

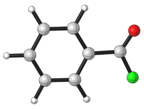

C -2.54488 -0.03618 0.
C -1.81851 -1.22471 -0.00002
C -0.42919 -1.19101 -0.00002
C 0.23158 0.03951 0.
C -0.49754 1.23188 0.00002
C -1.88524 1.1916 0.00002
H -3.63046 -0.06682 0.
H -2.33527 -2.17912 -0.00003
H 0.142 -2.11207 -0.00004
H 0.03683 2.17612 0.00003
H -2.45325 2.11648 0.00003
C 1.70752 0.13153 0.
O 2.37225 1.12006 -0.00004
F 2.29774 -1.08341 0.00005

E(RM06L) = -444.950012541
E(RM06) = -444.736335976

25 °C:
Zero-point correction= 0.104387 (Hartree/Particle)
Thermal correction to Energy= 0.111285
Thermal correction to Enthalpy= 0.112229
Thermal correction to Gibbs Free Energy= 0.072410
Sum of electronic and zero-point Energies= -444.585249
Sum of electronic and thermal Energies= -444.578351
Sum of electronic and thermal Enthalpies= -444.577407
Sum of electronic and thermal Free Energies= -444.617226

160 °C:
Zero-point correction= 0.104387 (Hartree/Particle)
Thermal correction to Energy= 0.118015
Thermal correction to Enthalpy= 0.119387
Thermal correction to Gibbs Free Energy= 0.053046
Sum of electronic and zero-point Energies= -444.585249
Sum of electronic and thermal Energies= -444.571621
Sum of electronic and thermal Enthalpies= -444.570249
Sum of electronic and thermal Free Energies= -444.63659

[1,1'-Biphenyl]-4-carbonyl fluoride 1a

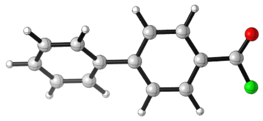

C -0.42531 0.01569 -0.00599
C 0.30097 -1.13236 0.33369
C 1.68819 -1.12546 0.33139
C 2.37676 0.04006 -0.01123
C 1.66444 1.19288 -0.35147
C 0.27888 1.17754 -0.34911
H -0.22921 -2.03381 0.62565
H 2.23756 -2.01951 0.60291
H 2.21108 2.08989 -0.62328
H -0.26838 2.06944 -0.63888
C 3.85194 0.1037 -0.02897
O 4.53434 1.0406 -0.30532
F 4.42097 -1.07158 0.3183
C -1.90914 0.00257 -0.00203
C -2.63069 1.09084 0.50069
C -2.61477 -1.09797 -0.50056
C -4.02108 1.07801 0.50639
H -2.09845 1.94306 0.91395
C -4.00518 -1.10967 -0.49744
H -2.0702 -1.94061 -0.91743
C -4.71272 -0.02194 0.00673
H -4.56469 1.92734 0.90904
H -4.53637 -1.96826 -0.89701
H -5.7985 -0.03152 0.01028
E(RM06L) = -676.055607791
E(RM06) = -675.691285204

25 °C:
Zero-point correction= 0.186343 (Hartree/Particle)
Thermal correction to Energy= 0.197842
Thermal correction to Enthalpy= 0.198787
Thermal correction to Gibbs Free Energy= 0.147413
Sum of electronic and zero-point Energies= -675.483642
Sum of electronic and thermal Energies= -675.472142
Sum of electronic and thermal Enthalpies= -675.471198
Sum of electronic and thermal Free Energies= -675.522572

160 °C:
Zero-point correction= 0.186343 (Hartree/Particle)
Thermal correction to Energy= 0.209572
Thermal correction to Enthalpy= 0.210944
Thermal correction to Gibbs Free Energy= 0.121893
Sum of electronic and zero-point Energies= -675.483642
Sum of electronic and thermal Energies= -675.460413
Sum of electronic and thermal Enthalpies= -675.459041
Sum of electronic and thermal Free Energies= -675.548092

$ArCOCF_3$

2,2,2-Trifluoro-1-phenylethanone

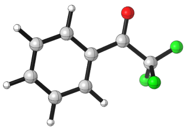

C -3.34151 -0.46085 -0.00004
C -2.34165 -1.4295 -0.00002
C -1.00343 -1.05395 0.00001
C -0.66184 0.30366 0.00001
C -1.67259 1.27239 0.00001
C -3.00635 0.89221 -0.00002
H -4.38535 -0.76043 -0.00007
H -2.60328 -2.48285 -0.00003
H -0.23906 -1.82128 0.00002
H -1.38877 2.3195 0.00003
H -3.78525 1.64792 -0.00003

C	0.73912	0.798	0.00003
O	1.03499	1.96967	0.00001
C	1.89505	-0.23701	0.
F	3.07461	0.36593	0.00002
F	1.82277	-1.02574	-1.08592
F	1.82273	-1.02574	1.08591

E(RM06L) = -682.793072493
E(RM06) = -682.527789904

25 °C:
Zero-point correction= 0.116883 (Hartree/Particle)
Thermal correction to Energy= 0.126620
Thermal correction to Enthalpy= 0.127564
Thermal correction to Gibbs Free Energy= 0.080008
Sum of electronic and zero-point Energies= -682.284011
Sum of electronic and thermal Energies= -682.274274
Sum of electronic and thermal Enthalpies= -682.273330
Sum of electronic and thermal Free Energies= -682.320886

160 °C:
Zero-point correction= 0.116883 (Hartree/Particle)
Thermal correction to Energy= 0.135778
Thermal correction to Enthalpy= 0.137150
Thermal correction to Gibbs Free Energy= 0.056678
Sum of electronic and zero-point Energies= -682.284011
Sum of electronic and thermal Energies= -682.265116
Sum of electronic and thermal Enthalpies= -682.263744
Sum of electronic and thermal Free Energies= -682.344216

1-([1,1'-biphenyl]-4-yl)-2,2,2-trifluoroethanone 4a

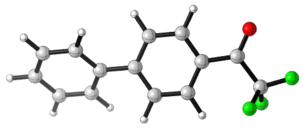

C	-1.4014	0.16688	-0.03582
C	-0.55176	-0.91141	0.23775
C	0.827	-0.76272	0.20926
C	1.39168	0.48197	-0.09151
C	0.54791	1.56552	-0.36311
C	-0.82711	1.40927	-0.33713
H	-0.97869	-1.87483	0.49907
H	1.45136	-1.61873	0.43475
H	0.99486	2.5247	-0.60221
H	-1.46859	2.25184	-0.57745
C	2.85168	0.73476	-0.14487
O	3.33615	1.81781	-0.37714
C	-2.87467	-0.00258	-0.00572
C	-3.69618	0.98464	0.54969
C	-3.4708	-1.15409	-0.53154
C	-5.07671	0.82297	0.58164
H	-3.24775	1.87401	0.98354
C	-4.85141	-1.31462	-0.50188
H	-2.84903	-1.91845	-0.98927
C	-5.65875	-0.32703	0.05564
H	-5.69886	1.59488	1.02456
H	-5.29804	-2.21069	-0.9221
H	-6.73683	-0.45359	0.08089
C	3.82214	-0.45101	0.10018
F	5.08468	-0.06186	0.00258
F	3.60887	-1.42675	-0.79933
F	3.63454	-0.97438	1.32451

E(RM06L) = -913.898971172
E(RM06) = -913.483136815

25 °C:
Zero-point correction= 0.199064 (Hartree/Particle)
Thermal correction to Energy= 0.213376
Thermal correction to Enthalpy= 0.214320
Thermal correction to Gibbs Free Energy= 0.155598
Sum of electronic and zero-point Energies= -913.182470
Sum of electronic and thermal Energies= -913.168158
Sum of electronic and thermal Enthalpies= -913.167214
Sum of electronic and thermal Free Energies= -913.225936

160 °C:
Zero-point correction= 0.199064 (Hartree/Particle)
Thermal correction to Energy= 0.227517
Thermal correction to Enthalpy= 0.228889
Thermal correction to Gibbs Free Energy= 0.126291
Sum of electronic and zero-point Energies= -913.182470
Sum of electronic and thermal Energies= -913.154017
Sum of electronic and thermal Enthalpies= -913.152645
Sum of electronic and thermal Free Energies= -913.255243

<u>ArCF$_3$</u>

(Trifluoromethyl)benzene

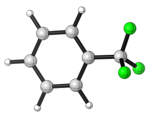

C	-2.82829	-0.01476	0.00006
C	-2.14954	1.19914	0.00014
C	-0.75728	1.22147	0.00001
C	-0.05075	0.02355	-0.00022
C	-0.72772	-1.19586	-0.00029
C	-2.11642	-1.21309	-0.00015
H	-3.91407	-0.02938	0.00015
H	-2.70225	2.13343	0.00029
H	-0.22318	2.16471	0.00005
H	-0.16749	-2.1257	-0.00052
H	-2.64462	-2.16137	-0.00024
C	1.45411	0.00927	0.
F	1.939	-0.62903	1.08051
F	1.97808	1.24573	-0.00303
F	1.93925	-0.63447	-1.07717

E(RM06L) = -569.439792572
E(RM06) = -569.221974444

25 °C:
Zero-point correction= 0.106913 (Hartree/Particle)
Thermal correction to Energy= 0.113838
Thermal correction to Enthalpy= 0.114782
Thermal correction to Gibbs Free Energy= 0.074998
Sum of electronic and zero-point Energies= -569.012475
Sum of electronic and thermal Energies= -569.005550
Sum of electronic and thermal Enthalpies= -569.004606
Sum of electronic and thermal Free Energies= -569.044390

160 °C:
Zero-point correction= 0.106927 (Hartree/Particle)
Thermal correction to Energy= 0.121046
Thermal correction to Enthalpy= 0.122418
Thermal correction to Gibbs Free Energy= 0.055578
Sum of electronic and zero-point Energies= -569.012462
Sum of electronic and thermal Energies= -568.998342
Sum of electronic and thermal Enthalpies= -568.996970
Sum of electronic and thermal Free Energies= -569.063810

4-(Trifluoromethyl)-1,1'-biphenyl 2a

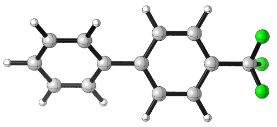

C	0.7964	0.00664	-0.01702
C	0.0791	-1.11533	-0.44694
C	-1.30907	-1.11422	-0.45711
C	-2.00585	0.01646	-0.03872
C	-1.30791	1.14219	0.39017
C	0.08043	1.13403	0.40006

H	0.61525	-1.99054	-0.80151
H	-1.85038	-1.98776	-0.80448
H	-1.84836	2.02332	0.71899
H	0.61713	2.00804	0.75677
C	2.28092	0.00132	-0.00527
C	3.00325	1.12413	-0.42342
C	2.98724	-1.12691	0.42556
C	4.39377	1.11908	-0.41101
H	2.47102	1.99981	-0.78453
C	4.37771	-1.13236	0.43835
H	2.44191	-1.99849	0.77694
C	5.08585	-0.0092	0.02012
H	4.93761	1.9971	-0.74653
H	4.90872	-2.0143	0.78396
H	6.17165	-0.01318	0.03033
C	-3.50829	-0.00193	0.00796
F	-4.03034	1.22603	-0.1523
F	-4.02931	-0.7896	-0.94849
F	-3.96099	-0.46837	1.18676

E(RM06L) = -800.544611851
E(RM06) = -800.176169960

25 °C:
Zero-point correction= 0.188991 (Hartree/Particle)
Thermal correction to Energy= 0.201415
Thermal correction to Enthalpy= 0.202359
Thermal correction to Gibbs Free Energy= 0.147832
Sum of electronic and zero-point Energies= -799.910560
Sum of electronic and thermal Energies= -799.898137
Sum of electronic and thermal Enthalpies= -799.897193
Sum of electronic and thermal Free Energies= -799.951719

160 °C:
Zero-point correction= 0.188991 (Hartree/Particle)
Thermal correction to Energy= 0.214030
Thermal correction to Enthalpy= 0.215401
Thermal correction to Gibbs Free Energy= 0.120717
Sum of electronic and zero-point Energies= -799.910560
Sum of electronic and thermal Energies= -799.885522
Sum of electronic and thermal Enthalpies= -799.884150
Sum of electronic and thermal Free Energies= -799.978835

Oxidative addition transition states

OA of benzoyl fluoride to PdXantPhos

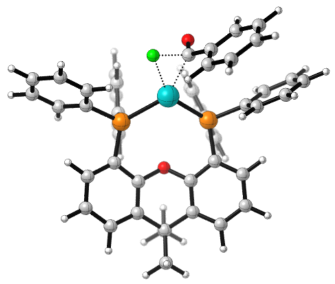

Pd	0.61937	-1.16022	-0.30267
P	1.28067	0.97139	0.27796
P	-1.83333	-1.27896	-0.21108
O	-1.52618	1.57786	-0.27548
C	-2.23886	-1.14044	1.57393
C	-2.98786	-0.10128	2.12508
H	-3.41871	0.66304	1.48594
C	-3.19433	-0.03788	3.5015
H	-3.77163	0.78147	3.9196
C	-2.66844	-1.01838	4.33539
H	-2.83436	-0.96766	5.40759
C	-1.91916	-2.05982	3.79086
H	-1.49693	-2.82521	4.43545
C	-1.69391	-2.11435	2.42132
H	-1.08456	-2.91455	2.00695
C	-2.52601	-2.91742	-0.63816
C	-3.73865	-3.38361	-0.11384
H	-4.29729	-2.76611	0.58485
C	-4.22596	-4.63299	-0.47899
H	-5.16616	-4.98855	-0.06765
C	-3.5021	-5.43245	-1.36382
H	-3.88116	-6.41153	-1.64239
C	-2.29017	-4.98243	-1.87636
H	-1.71268	-5.61076	-2.54744
C	-1.80044	-3.72948	-1.51335
H	-0.83407	-3.39752	-1.88122
C	-2.9778	-0.11009	-1.0371
C	-4.10341	-0.47301	-1.78054
H	-4.39739	-1.51568	-1.8391
C	-4.83192	0.49209	-2.46797
H	-5.70017	0.1973	-3.049
C	-4.45568	1.83242	-2.42147
H	-5.03866	2.56479	-2.97002
C	-3.34585	2.23446	-1.68114
C	-2.63535	1.24115	-1.01072
C	-2.8701	3.67386	-1.49933
C	-1.34262	3.63728	-1.46791
C	-0.5045	4.62313	-1.98399
H	-0.92422	5.47219	-2.51299
C	0.87406	4.54213	-1.81289
H	1.51405	5.32531	-2.20687
C	1.44065	3.46967	-1.13224
H	2.51551	3.42876	-0.99868
C	0.63821	2.44175	-0.62959
C	-0.74005	2.56627	-0.80833
C	-3.40558	4.60128	-2.59293
H	-3.07691	4.28537	-3.58843
H	-3.0719	5.62962	-2.42486
H	-4.49947	4.62314	-2.57682
C	-3.36767	4.18095	-0.12308
H	-2.99942	3.5467	0.68935
H	-4.46284	4.1791	-0.09322
H	-3.01243	5.20234	0.05226
C	0.78713	1.34174	2.00509
C	0.43133	2.62675	2.42497
H	0.43849	3.45247	1.71958
C	0.06299	2.8556	3.74696
H	-0.21765	3.8566	4.06204
C	0.05794	1.80575	4.66145
H	-0.23033	1.98451	5.69338
C	0.42299	0.527	4.25094
H	0.41341	-0.29727	4.95687
C	0.7802	0.28999	2.92734
H	1.05891	-0.712	2.61088
C	3.08544	1.2855	0.24821
C	3.82471	1.57907	1.39421
H	3.32873	1.66005	2.35627
C	5.20327	1.76854	1.30823
H	5.76986	1.99131	2.20768
C	5.84829	1.68053	0.07949
H	6.92196	1.83114	0.01502
C	5.11551	1.38614	-1.06903
H	5.61338	1.2898	-2.0284
C	3.74629	1.17508	-0.98166
H	3.18475	0.91221	-1.87435
C	2.34824	-2.10516	0.21223
F	1.26239	-3.19603	-0.61754
O	2.40722	-2.32742	1.39848
C	3.52324	-2.05	-0.71354
C	4.78841	-1.99828	-0.13027
C	3.39359	-1.9925	-2.10218
C	5.92101	-1.89158	-0.93121
H	4.87022	-2.03429	0.95076
C	4.52671	-1.88633	-2.89934
H	2.40586	-2.03731	-2.54885
C	5.79278	-1.83538	-2.31579
H	6.90347	-1.84444	-0.47087
H	4.4231	-1.84834	-3.98002
H	6.67687	-1.75308	-2.94199

E(RM06L) = -2836.743585
E(RM06) = -2835.43082651

25 °C:
Zero-point correction= 0.709814 (Hartree/Particle)
Thermal correction to Energy= 0.754609
Thermal correction to Enthalpy= 0.755554
Thermal correction to Gibbs Free Energy= 0.629795
Sum of electronic and zero-point Energies= -2834.761084
Sum of electronic and thermal Energies= -2834.716288
Sum of electronic and thermal Enthalpies= -2834.715344
Sum of electronic and thermal Free Energies= -2834.84110

160 °C:
Zero-point correction= 0.709814 (Hartree/Particle)
Thermal correction to Energy= 0.800458
Thermal correction to Enthalpy= 0.801829
Thermal correction to Gibbs Free Energy= 0.564237
Sum of electronic and zero-point Energies= -2834.761084
Sum of electronic and thermal Energies= -2834.670440
Sum of electronic and thermal Enthalpies= -2834.669068
Sum of electronic and thermal Free Energies= -2834.906661

OA of [1,1'-biphenyl]-4-carbonyl fluoride 1a to PdXantPhos

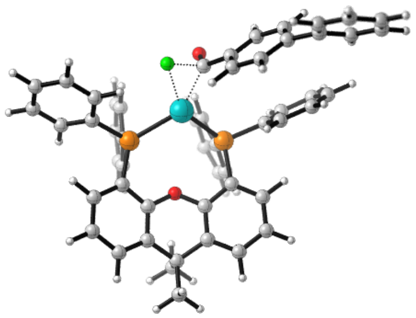

Pd	0.02555	1.08546	0.05591
P	-0.29844	-1.12824	0.63257
P	2.41264	1.42456	-0.4353
O	2.31164	-1.43415	-0.64349
C	3.25315	1.20455	1.1834
C	4.17778	0.19567	1.45074
H	4.47758	-0.4958	0.66967
C	4.73166	0.07048	2.72299
H	5.44654	-0.7227	2.92103
C	4.37809	0.95937	3.73192
H	4.81683	0.86244	4.7208
C	3.45224	1.96816	3.47204
H	3.16219	2.66145	4.25608
C	2.88274	2.0828	2.21025
H	2.14081	2.85546	2.02119
C	2.8735	3.14265	-0.86296
C	4.15848	3.65049	-0.62949
H	4.91395	3.01574	-0.17337
C	4.46679	4.962	-0.96924
H	5.46474	5.34914	-0.78543
C	3.49139	5.78242	-1.53665
H	3.72989	6.8107	-1.79278
C	2.20986	5.28955	-1.75683
H	1.44222	5.93215	-2.17699
C	1.89834	3.97324	-1.42034
H	0.88489	3.60451	-1.54901
C	3.39786	0.41687	-1.60633
C	4.27043	0.92126	-2.57368
H	4.46056	1.98805	-2.62479
C	4.87464	0.06504	-3.48848
H	5.54561	0.46831	-4.24043
C	4.62589	-1.30483	-3.45039
H	5.10843	-1.95103	-4.17615
C	3.76967	-1.84582	-2.49363
C	3.1751	-0.95904	-1.59927
C	3.46823	-3.33026	-2.30427
C	1.99801	-3.43727	-1.90219
C	1.13811	-4.46927	-2.2701
H	1.47598	-5.24298	-2.9514
C	-0.15372	-4.53137	-1.75661
H	-0.80813	-5.35017	-2.03843
C	-0.61267	-3.55715	-0.87638
H	-1.61647	-3.63225	-0.47451
C	0.20398	-2.48187	-0.51445
C	1.49747	-2.46366	-1.03776
C	3.79112	-4.14906	-3.55632
H	3.20507	-3.81752	-4.4197
H	3.59207	-5.21123	-3.38532
H	4.85388	-4.06735	-3.80347
C	4.32743	-3.85317	-1.12648
H	4.11067	-3.3057	-0.20382
H	5.39243	-3.73578	-1.35481
H	4.12025	-4.91451	-0.95072
C	0.64964	-1.53803	2.1478
C	1.21835	-2.79671	2.36185
H	1.11231	-3.57754	1.61454
C	1.92668	-3.0557	3.53099
H	2.37284	-4.03402	3.68491
C	2.06018	-2.06507	4.5001
H	2.6125	-2.26795	5.41322
C	1.48488	-0.81416	4.29695
H	1.59045	-0.03547	5.04542
C	0.78971	-0.54429	3.12251
H	0.3494	0.43729	2.96533
C	-2.02029	-1.61082	1.03896
C	-2.41718	-1.9749	2.32619
H	-1.68948	-2.0131	3.13047
C	-3.7499	-2.29065	2.58594
H	-4.04755	-2.56714	3.59321
C	-4.69021	-2.26072	1.56225
H	-5.7268	-2.51464	1.76617
C	-4.30281	-1.8937	0.27453
H	-5.03709	-1.843	-0.52375
C	-2.98038	-1.55531	0.02008
H	-2.69059	-1.2402	-0.97887
C	-1.5679	1.83541	1.0561
F	-0.84045	3.05625	-0.02075
O	-1.31244	2.04506	2.21761
C	-2.94164	1.68693	0.48878
C	-3.9843	1.49941	1.39411
C	-3.2024	1.64332	-0.88086
C	-5.26983	1.24781	0.9351
H	-3.77317	1.54236	2.45715
C	-4.48864	1.39016	-1.33482
H	-2.38939	1.78942	-1.58443
C	-5.53984	1.17202	-0.43439
H	-6.07475	1.10592	1.65063
H	-4.6754	1.32181	-2.40312
C	-6.89566	0.81475	-0.91979
C	-7.63754	-0.17764	-0.26721
C	-7.45616	1.43597	-2.04169
C	-8.9013	-0.53929	-0.72156
H	-7.20668	-0.68151	0.59395
C	-8.71993	1.07468	-2.49737
H	-6.90453	2.22423	-2.54672
C	-9.44706	0.08522	-1.83986
H	-9.45774	-1.31626	-0.20515
H	-9.14147	1.57266	-3.36571
H	-10.43319	-0.19619	-2.19725

E(RM06L) = -3067.85027725
E(RM06) = -3066.38672391

25 °C:
Zero-point correction= 0.792402 (Hartree/Particle)
Thermal correction to Energy= 0.841704
Thermal correction to Enthalpy= 0.842648
Thermal correction to Gibbs Free Energy= 0.707151
Sum of electronic and zero-point Energies= -3065.658001
Sum of electronic and thermal Energies= -3065.608700
Sum of electronic and thermal Enthalpies= -3065.607756
Sum of electronic and thermal Free Energies= -3065.743252

160 °C:
Zero-point correction= 0.792402 (Hartree/Particle)
Thermal correction to Energy= 0.892479
Thermal correction to Enthalpy= 0.893851
Thermal correction to Gibbs Free Energy= 0.636274

Sum of electronic and zero-point Energies= -3065.658001
Sum of electronic and thermal Energies= -3065.557925
Sum of electronic and thermal Enthalpies= -3065.556553
Sum of electronic and thermal Free Energies= -3065.814129

ArCO-[Pd$^{(II)}$]-F intermediates

PhenylCO-Pd$^{(II)}$-F (trans)

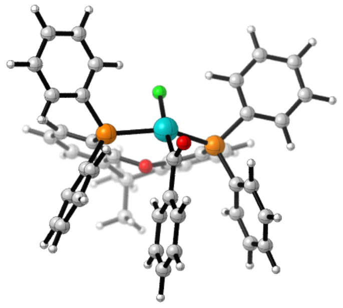

Pd	0.00538	0.19341	-1.2385
P	2.22762	-0.07787	-0.59535
P	-2.22146	-0.07551	-0.60668
O	-0.00401	-1.11892	1.28307
C	-3.37826	-0.61967	-1.90971
C	-4.6883	-0.14783	-2.01764
H	-5.06655	0.58341	-1.30909
C	-5.51177	-0.61399	-3.04005
H	-6.52821	-0.24088	-3.12588
C	-5.03115	-1.55297	-3.9482
H	-5.67529	-1.91517	-4.74441
C	-3.72409	-2.02536	-3.83823
H	-3.34794	-2.75502	-4.54922
C	-2.89028	-1.56016	-2.82715
H	-1.8631	-1.91168	-2.72654
C	-3.05695	1.2959	0.26081
C	-3.45562	1.21486	1.59612
H	-3.35906	0.27901	2.1375
C	-3.96525	2.33663	2.24523
H	-4.2622	2.26447	3.2874
C	-4.08735	3.54355	1.56548
H	-4.47609	4.41989	2.07541
C	-3.70287	3.62816	0.22864
H	-3.78546	4.57004	-0.30469
C	-3.18549	2.51363	-0.41857
H	-2.85045	2.59729	-1.44972
C	-2.27148	-1.46362	0.59641
C	-3.44972	-2.20412	0.73832
H	-4.31193	-1.94906	0.12998
C	-3.52587	-3.26873	1.62439
H	-4.44647	-3.83657	1.71254
C	-2.4209	-3.60606	2.39913
H	-2.49482	-4.43738	3.09187
C	-1.23415	-2.88576	2.30208
C	-1.17489	-1.83135	1.38602
C	-0.01035	-3.11741	3.18463
C	1.22436	-2.87646	2.3201
C	2.41509	-3.58733	2.43546
H	2.48585	-4.41578	3.13194
C	3.52782	-3.24419	1.67448
H	4.45174	-3.80404	1.77753
C	3.45465	-2.18472	0.78226
H	4.32239	-1.92673	0.18309
C	2.27258	-1.45417	0.6214
C	1.16935	-1.82484	1.40039
C	-0.02359	-2.05889	4.31575
H	-0.0235	-1.04368	3.90735
H	0.8618	-2.17385	4.95066
H	-0.91936	-2.18002	4.9347
C	-0.00952	-4.51113	3.81842
H	0.00015	-5.30036	3.05993
H	-0.88996	-4.64641	4.45336
H	0.86124	-4.6388	4.46816
C	3.3883	-0.63568	-1.88931
C	2.89695	-1.5735	-2.80766
H	1.86559	-1.91456	-2.71312
C	3.73298	-2.0503	-3.81158
H	3.35405	-2.77783	-4.52329
C	5.04576	-1.59245	-3.91333
H	5.69163	-1.96355	-4.70403
C	5.53013	-0.65654	-3.00392
H	6.55132	-0.29514	-3.08314
C	4.7044	-0.17874	-1.98883
H	5.08562	0.55025	-1.27952
C	3.05782	1.30255	0.26191
C	3.40034	1.25187	1.61409
H	3.26838	0.3318	2.17477
C	3.89803	2.384	2.25422
H	4.15015	2.33616	3.30949
C	4.06437	3.57081	1.54895
H	4.44382	4.45534	2.05162
C	3.73657	3.62458	0.19549
H	3.85509	4.55072	-0.35833
C	3.23058	2.50001	-0.44336
H	2.94227	2.55965	-1.49005
C	0.01522	2.15595	-1.106
O	0.0279	2.73253	-2.17804
C	0.00796	2.94502	0.175
C	-0.01906	2.32036	1.4203
C	0.02357	4.34077	0.10196
C	-0.03403	3.08233	2.58397
H	-0.02913	1.2357	1.47431
C	0.01054	5.10174	1.26275
H	0.04588	4.80822	-0.87734
C	-0.01962	4.47198	2.50675
H	-0.05779	2.59007	3.5518
H	0.02332	6.18638	1.20156
H	-0.03125	5.06615	3.41646
F	0.00071	-1.81282	-1.75843

E(RM06L) = -2836.76137188
E(RM06) = -2835.44548723

25 °C:
Zero-point correction= 0.710903 (Hartree/Particle)
Thermal correction to Energy= 0.756296
Thermal correction to Enthalpy= 0.757240
Thermal correction to Gibbs Free Energy= 0.629748
Sum of electronic and zero-point Energies= -2834.779220
Sum of electronic and thermal Energies= -2834.733827
Sum of electronic and thermal Enthalpies= -2834.732883
Sum of electronic and thermal Free Energies= -2834.860376

160 °C:
Zero-point correction= 0.710903 (Hartree/Particle)
Thermal correction to Energy= 0.802410
Thermal correction to Enthalpy= 0.803782
Thermal correction to Gibbs Free Energy= 0.563350
Sum of electronic and zero-point Energies= -2834.779220
Sum of electronic and thermal Energies= -2834.687713
Sum of electronic and thermal Enthalpies= -2834.686341
Sum of electronic and thermal Free Energies= -2834.926773

PhenylCO-Pd$^{(II)}$-F (cis)

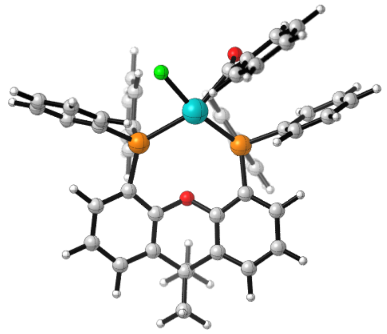

Pd	0.74519	-1.14808	-0.05685
P	1.22656	1.09954	0.16927
P	-1.72495	-1.37254	-0.12394
O	-1.61377	1.52087	-0.25379
C	-2.06845	-1.36332	1.67612
C	-2.94434	-0.46771	2.28868
H	-3.5218	0.23016	1.68962
C	-3.08427	-0.46514	3.67505
H	-3.76547	0.23817	4.14506
C	-2.3563	-1.35969	4.45278
H	-2.46975	-1.35718	5.5333
C	-1.48083	-2.25849	3.84366
H	-0.90481	-2.95473	4.44595
C	-1.32903	-2.25969	2.46204
H	-0.63101	-2.94269	1.98088
C	-2.29572	-3.01846	-0.66879
C	-3.31521	-3.70939	-0.00871
H	-3.75982	-3.28936	0.88936
C	-3.76296	-4.93309	-0.49815
H	-4.55574	-5.46466	0.02013
C	-3.19249	-5.47336	-1.64802
H	-3.54013	-6.42937	-2.02902
C	-2.16867	-4.79322	-2.30301
H	-1.70907	-5.22213	-3.18826
C	-1.71686	-3.57287	-1.81241
H	-0.89559	-3.05764	-2.30069
C	-3.01141	-0.27814	-0.8447
C	-4.18496	-0.72805	-1.45685
H	-4.41976	-1.78702	-1.45621
C	-5.03495	0.1695	-2.09342
H	-5.93933	-0.19387	-2.57113
C	-4.72671	1.52713	-2.13979
H	-5.39345	2.20466	-2.66281
C	-3.57444	2.01521	-1.52817
C	-2.75768	1.09015	-0.88074
C	-3.13936	3.47879	-1.48749
C	-1.6158	3.4845	-1.61138
C	-0.86227	4.43834	-2.29144
H	-1.35729	5.22822	-2.84658
C	0.52897	4.40152	-2.25489
H	1.10232	5.15859	-2.78039
C	1.19261	3.41016	-1.53928
H	2.2761	3.40922	-1.50353
C	0.47196	2.41708	-0.87049
C	-0.91833	2.48795	-0.9297
C	-3.81316	4.30958	-2.58217
H	-3.58136	3.9271	-3.58152
H	-3.49456	5.35478	-2.52425
H	-4.8995	4.30794	-2.45136
C	-3.51357	4.06776	-0.10524
H	-3.03797	3.50999	0.70727
H	-4.59884	4.02855	0.03884
H	-3.18822	5.11185	-0.03953
C	0.7794	1.66476	1.85421
C	0.56917	3.01814	2.14111
H	0.68868	3.76651	1.36273
C	0.19742	3.41237	3.42178
H	0.03175	4.46447	3.63436
C	0.03204	2.45918	4.42484
H	-0.26665	2.76776	5.42268
C	0.25027	1.11383	4.14727
H	0.11739	0.36296	4.91929
C	0.62379	0.71449	2.86714
H	0.80144	-0.33586	2.66203
C	3.01111	1.46562	-0.0095
C	3.80362	1.86575	1.06588
H	3.35581	2.02944	2.04081
C	5.17616	2.03645	0.89549
H	5.78707	2.33917	1.74074
C	5.76237	1.81736	-0.34676
H	6.83301	1.94663	-0.47395
C	4.97586	1.41821	-1.42566
H	5.42909	1.21915	-2.39145
C	3.61043	1.23373	-1.25414
H	3.00698	0.8861	-2.08887
C	2.61638	-1.5032	0.55275
F	0.66429	-3.1613	-0.01964
O	2.85804	-1.49293	1.73686
C	3.66555	-1.8368	-0.46441
C	5.00497	-1.80913	-0.07024
C	3.33677	-2.08674	-1.79688
C	6.00953	-2.00871	-1.0088
H	5.23768	-1.60659	0.97008
C	4.34241	-2.29713	-2.73375
H	2.28968	-2.11852	-2.08645
C	5.67939	-2.24911	-2.34193
H	7.05171	-1.97094	-0.70481
H	4.08626	-2.49863	-3.76982
H	6.46495	-2.40409	-3.0765

E(RM06L) = -2836.75676937
E(RM06) = -2835.44311905

25 °C:
Zero-point correction= 0.710668 (Hartree/Particle)
Thermal correction to Energy= 0.756048
Thermal correction to Enthalpy= 0.756992
Thermal correction to Gibbs Free Energy= 0.630670
Sum of electronic and zero-point Energies= -2834.773252
Sum of electronic and thermal Energies= -2834.727872
Sum of electronic and thermal Enthalpies= -2834.726928
Sum of electronic and thermal Free Energies= -2834.853250

160 °C:
Zero-point correction= 0.710674 (Hartree/Particle)
Thermal correction to Energy= 0.802226
Thermal correction to Enthalpy= 0.803598
Thermal correction to Gibbs Free Energy= 0.564810
Sum of electronic and zero-point Energies= -2834.773246
Sum of electronic and thermal Energies= -2834.681694
Sum of electronic and thermal Enthalpies= -2834.680322
Sum of electronic and thermal Free Energies= -2834.919110

[1,1'-Biphenyl]-4-CO-Pd$^{(II)}$-F

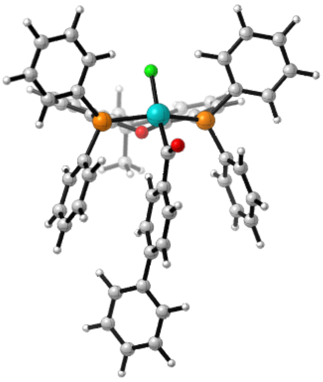

Pd	-1.21353	0.11186	-1.23232
P	-1.61462	-2.02478	-0.40098
P	-0.53892	2.28179	-0.66739
O	-0.92961	0.23075	1.60401
C	-1.34495	3.64527	-1.57393
C	-0.68043	4.80923	-1.96541
H	0.37439	4.94022	-1.74138
C	-1.37354	5.80644	-2.64849
H	-0.85471	6.70997	-2.95518
C	-2.72621	5.64478	-2.93409
H	-3.26431	6.42452	-3.4657
C	-3.38896	4.48291	-2.54147
H	-4.44366	4.35421	-2.76579
C	-2.70528	3.47763	-1.86674
H	-3.20372	2.56078	-1.55096
C	1.24312	2.687	-0.62172
C	1.91666	3.00624	0.55911
H	1.36553	3.12938	1.48603
C	3.30244	3.14951	0.56051
H	3.81582	3.38997	1.48732
C	4.02622	2.97651	-0.61457
H	5.10846	3.06661	-0.60717

C	3.35863	2.67717	-1.80059
H	3.91607	2.53009	-2.72013
C	1.97737	2.53152	-1.80387
H	1.46772	2.26035	-2.72548
C	-1.07958	2.5587	1.06627
C	-1.36661	3.85613	1.50211
H	-1.2955	4.67887	0.79773
C	-1.75773	4.10353	2.81012
H	-1.98714	5.11643	3.12508
C	-1.85067	3.05319	3.71705
H	-2.14724	3.26121	4.73932
C	-1.55998	1.74857	3.32895
C	-1.20023	1.52086	1.99783
C	-1.53348	0.55052	4.27423
C	-2.0778	-0.64547	3.49854
C	-2.86196	-1.65225	4.05275
H	-3.17864	-1.58417	5.08786
C	-3.24279	-2.75708	3.29782
H	-3.85193	-3.5368	3.74358
C	-2.84702	-2.85825	1.97244
H	-3.15838	-3.71538	1.38351
C	-2.07568	-1.85885	1.36924
C	-1.70568	-0.76091	2.1555
C	-0.05786	0.25739	4.64442
H	0.54328	0.0569	3.75242
H	-0.00079	-0.61816	5.30056
H	0.37766	1.11683	5.16588
C	-2.31728	0.81303	5.56282
H	-3.37216	1.02443	5.35993
H	-1.88884	1.65957	6.1073
H	-2.25667	-0.04831	6.23475
C	-3.03266	-2.91961	-1.11883
C	-4.15341	-2.1443	-1.44529
H	-4.12379	-1.06928	-1.26392
C	-5.27737	-2.75966	-1.98564
H	-6.14642	-2.15987	-2.23936
C	-5.28783	-4.13525	-2.21096
H	-6.16586	-4.60876	-2.64079
C	-4.17192	-4.90322	-1.88959
H	-4.17729	-5.97496	-2.06582
C	-3.04361	-4.29861	-1.3407
H	-2.17372	-4.89957	-1.09069
C	-0.21468	-3.19382	-0.39373
C	0.47322	-3.52352	0.77521
H	0.10194	-3.18734	1.73857
C	1.64948	-4.2662	0.7111
H	2.18445	-4.50438	1.62558
C	2.1413	-4.69308	-0.51771
H	3.06527	-5.2615	-0.56649
C	1.4502	-4.38155	-1.68711
H	1.83262	-4.70575	-2.65014
C	0.28329	-3.63052	-1.62794
H	-0.22712	-3.35449	-2.54717
C	0.4233	-0.43262	-2.17534
O	0.28718	-0.5786	-3.37599
C	1.76588	-0.63386	-1.53505
C	1.95454	-0.46915	-0.16489
C	2.86504	-0.92619	-2.34601
C	3.22643	-0.55436	0.38248
H	1.1009	-0.25272	0.47073
C	4.13535	-1.01296	-1.79873
H	2.70483	-1.05306	-3.41201
C	4.33764	-0.80967	-0.42731
H	3.36194	-0.42194	1.45203
H	4.98997	-1.19953	-2.44364
F	-3.11026	0.67264	-0.61583
C	5.70266	-0.84037	0.15144
C	6.63532	-1.80283	-0.25103
C	6.08835	0.10584	1.10849
C	7.91664	-1.82144	0.28983
H	6.34413	-2.55527	-0.97874
C	7.36931	0.08745	1.65042
H	5.38048	0.87376	1.40897
C	8.28826	-0.87648	1.24288
H	8.62549	-2.57987	-0.02945
H	7.65376	0.83275	2.38757
H	9.28791	-0.8916	1.66683

E(RM06L) = -3067.86704140
E(RM06) = -3066.40090686

25 °C:
Zero-point correction= 0.793326 (Hartree/Particle)
Thermal correction to Energy= 0.843153
Thermal correction to Enthalpy= 0.844097
Thermal correction to Gibbs Free Energy= 0.707053
Sum of electronic and zero-point Energies= -3065.676449
Sum of electronic and thermal Energies= -3065.626622
Sum of electronic and thermal Enthalpies= -3065.625678
Sum of electronic and thermal Free Energies= -3065.762723

160 °C:
Zero-point correction= 0.793331 (Hartree/Particle)
Thermal correction to Energy= 0.894217
Thermal correction to Enthalpy= 0.895589
Thermal correction to Gibbs Free Energy= 0.635427
Sum of electronic and zero-point Energies= -3065.676445
Sum of electronic and thermal Energies= -3065.575558
Sum of electronic and thermal Enthalpies= -3065.574186
Sum of electronic and thermal Free Energies= -3065.834348

Decarbonylation from ArCO-[Pd$^{(II)}$]-F transition states

Decarbonylation from phenylCO-Pd$^{(II)}$-F: 'bis-ligated' TS

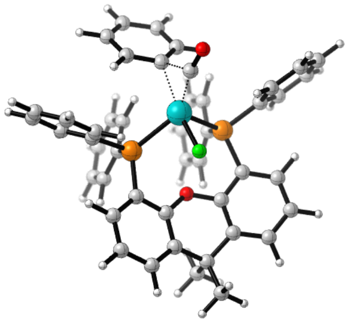

P	1.81365	-0.77697	-0.00576
P	-1.75345	0.72392	-0.05262
O	0.80941	1.9219	0.30689
C	-1.78749	0.50374	1.75892
C	-1.96853	1.56548	2.64896
H	-2.07316	2.57774	2.26829
C	-2.01456	1.32957	4.01986
H	-2.15025	2.1602	4.70655
C	-1.89044	0.03192	4.50963
H	-1.92608	-0.15046	5.57985
C	-1.70933	-1.0295	3.6272
H	-1.5891	-2.0404	4.00338
C	-1.64718	-0.79484	2.25864
H	-1.47699	-1.62195	1.57561
C	-3.51099	0.58962	-0.55165
C	-4.55529	0.38259	0.34975
H	-4.35619	0.35954	1.41689
C	-5.85567	0.19235	-0.11645
H	-6.66103	0.02596	0.59319
C	-6.12152	0.2179	-1.48162
H	-7.13496	0.06834	-1.84247
C	-5.08288	0.43291	-2.38771
H	-5.28315	0.45198	-3.45485
C	-3.78313	0.60872	-1.92724
H	-2.96726	0.75055	-2.63362
C	-1.38307	2.49156	-0.30158
C	-2.30276	3.45323	-0.71834
H	-3.34611	3.18438	-0.84771
C	-1.87952	4.74928	-0.99475
H	-2.59726	5.49196	-1.3282

C -0.53944 5.09772 -0.85204
H -0.23352 6.11345 -1.07949
C 0.40337 4.16829 -0.41488
C -0.05333 2.88075 -0.14905
C 1.87277 4.47677 -0.1342
C 2.68132 3.21528 -0.43716
C 3.99658 3.20569 -0.89736
H 4.49325 4.13864 -1.14161
C 4.69487 2.01053 -1.04241
H 5.71953 2.02528 -1.40047
C 4.08898 0.79857 -0.73241
H 4.63904 -0.12792 -0.85838
C 2.76692 0.76412 -0.28537
C 2.09849 1.98105 -0.15703
C 2.36585 5.68217 -0.94088
H 2.27327 5.50737 -2.01729
H 3.41243 5.90189 -0.71032
H 1.798 6.58073 -0.68143
C 2.01915 4.78555 1.37581
H 1.6661 3.94676 1.98461
H 1.4316 5.67178 1.64124
H 3.07013 4.97194 1.6232
C 1.80331 -0.99035 1.82019
C 1.89011 0.09343 2.69884
H 2.05759 1.09339 2.31505
C 1.76322 -0.09359 4.072
H 1.82553 0.76345 4.736
C 1.55965 -1.36663 4.59392
H 1.46466 -1.51045 5.66614
C 1.47129 -2.45459 3.72956
H 1.31388 -3.45452 4.12441
C 1.57935 -2.26591 2.35662
H 1.51019 -3.12634 1.69819
C 3.00357 -2.05297 -0.57828
C 3.98638 -2.63331 0.23096
H 4.06564 -2.34702 1.27543
C 4.86496 -3.57693 -0.29368
H 5.6207 -4.02261 0.34671
C 4.7804 -3.94298 -1.63522
H 5.46697 -4.6798 -2.04193
C 3.81658 -3.35898 -2.45277
H 3.74701 -3.63282 -3.50113
C 2.93117 -2.42208 -1.92721
H 2.18751 -1.95884 -2.57084
O -0.51609 -3.02394 -3.48455
Pd -0.47193 -0.84621 -1.43819
C -0.59254 -2.32586 -2.5525
C -1.33763 -2.77408 -0.97931
C -0.58009 -3.64976 -0.19091
C -2.73303 -2.76855 -0.86233
C -1.20379 -4.45596 0.75413
H 0.49777 -3.68836 -0.32433
C -3.35329 -3.56724 0.09309
H -3.32713 -2.11697 -1.49567
C -2.58945 -4.40292 0.90568
H -0.61073 -5.12086 1.37532
H -4.43244 -3.53029 0.20628
H -3.0762 -5.02519 1.651
F 0.18441 0.69076 -2.58455

E(RM06L) = -2836.71076093
E(RM06) = -2835.39598289

25 °C:
Zero-point correction= 0.707830 (Hartree/Particle)
Thermal correction to Energy= 0.753523
Thermal correction to Enthalpy= 0.754467
Thermal correction to Gibbs Free Energy= 0.626197
Sum of electronic and zero-point Energies= -2834.727090
Sum of electronic and thermal Energies= -2834.681397
Sum of electronic and thermal Enthalpies= -2834.680453
Sum of electronic and thermal Free Energies= -2834.808724

160 °C:
Zero-point correction= 0.707830 (Hartree/Particle)
Thermal correction to Energy= 0.799654
Thermal correction to Enthalpy= 0.801026
Thermal correction to Gibbs Free Energy= 0.559441
Sum of electronic and zero-point Energies= -2834.727090
Sum of electronic and thermal Energies= -2834.635266
Sum of electronic and thermal Enthalpies= -2834.633894
Sum of electronic and thermal Free Energies= -2834.875479

Decarbonylation from phenylCO-Pd$^{(II)}$-F: 'mono-ligated' TS

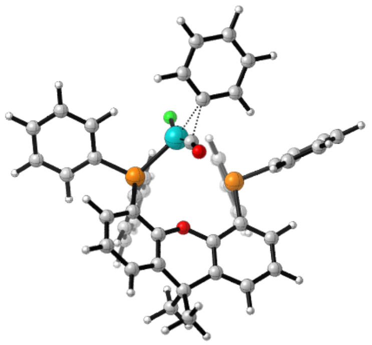

Pd -0.11743 1.79523 -0.26712
P -1.84647 -0.7454 0.29867
P 1.96696 0.77788 0.16806
O 0.7874 -1.77257 -0.44309
C 2.16991 -0.15798 1.72592
C 2.97927 -1.2944 1.82728
H 3.50284 -1.6767 0.95504
C 3.10929 -1.94945 3.04679
H 3.73342 -2.83563 3.11902
C 2.43687 -1.47088 4.16961
H 2.53332 -1.98702 5.12053
C 1.63849 -0.33632 4.06996
H 1.10169 0.03003 4.93913
C 1.49823 0.32369 2.8519
H 0.85518 1.19676 2.74874
C 3.2891 2.04064 0.26002
C 4.58297 1.70852 0.67947
H 4.81349 0.68664 0.96788
C 5.57314 2.68171 0.73681
H 6.574 2.41503 1.06294
C 5.27856 3.99879 0.38588
H 6.05192 4.75961 0.43701
C 3.9917 4.33937 -0.01672
H 3.75334 5.36684 -0.2742
C 2.99906 3.36392 -0.07818
H 1.98613 3.63229 -0.36581
C 2.56531 -0.3903 -1.10466
C 3.65904 -0.1579 -1.93805
H 4.20107 0.77932 -1.86644
C 4.05689 -1.12382 -2.85704
H 4.89841 -0.92961 -3.51419
C 3.40486 -2.35183 -2.91147
H 3.76236 -3.10722 -3.60302
C 2.31795 -2.62712 -2.08178
C 1.88766 -1.60533 -1.23777
C 1.66217 -3.99733 -1.92719
C 0.18489 -3.77866 -1.61111
C -0.83228 -4.6631 -1.96523
H -0.602 -5.54609 -2.55163
C -2.14656 -4.4392 -1.56767
H -2.92196 -5.14853 -1.83943
C -2.47128 -3.30371 -0.83541
H -3.50334 -3.12046 -0.5547
C -1.48845 -2.37355 -0.48396
C -0.17225 -2.65973 -0.85749
C 1.86337 -4.87532 -3.16618
H 1.41244 -4.42524 -4.05639
H 1.42533 -5.86613 -3.01569
H 2.92855 -5.03635 -3.35651
C 2.31588 -4.68955 -0.70513
H 2.18849 -4.0894 0.20202
H 3.38887 -4.82983 -0.87821
H 1.85499 -5.66886 -0.53475

C	-1.80123	-1.07237	2.09687
C	-1.75168	0.05157	2.93072
H	-1.6101	1.04279	2.50118
C	-1.80938	-0.10718	4.31278
H	-1.77491	0.76993	4.95276
C	-1.87787	-1.38087	4.87097
H	-1.90978	-1.5022	5.95023
C	-1.89242	-2.50221	4.04414
H	-1.93389	-3.49796	4.47632
C	-1.86642	-2.34923	2.66112
H	-1.89691	-3.2258	2.01994
C	-3.65213	-0.61769	-0.038
C	-4.0461	-0.44985	-1.37424
H	-3.29558	-0.45535	-2.16211
C	-5.38309	-0.26733	-1.70428
H	-5.66908	-0.1399	-2.74416
C	-6.35053	-0.23383	-0.70039
H	-7.39543	-0.08234	-0.95484
C	-5.96962	-0.39109	0.62788
H	-6.71719	-0.36511	1.41546
C	-4.62899	-0.58558	0.95922
H	-4.34559	-0.70847	1.99989
C	-0.50137	1.62501	-2.0548
O	-0.60084	1.28133	-3.16021
C	-1.55197	3.04424	-1.29217
C	-2.90821	2.8493	-1.02245
C	-1.09688	4.30756	-1.68344
C	-3.79581	3.91689	-1.11626
C	-1.98697	5.37315	-1.77584
C	-3.33651	5.17737	-1.49109
H	-3.27356	1.87349	-0.72656
H	-0.04675	4.45891	-1.919
H	-4.84724	3.76022	-0.8935
H	-1.62819	6.35429	-2.07306
H	-4.03189	6.00851	-1.56539
F	-0.26003	2.4864	1.6376

E(RM06L) = -2836.71481640
E(RM06) = -2835.40159608

25 °C:
Zero-point correction= 0.707652 (Hartree/Particle)
Thermal correction to Energy= 0.753575
Thermal correction to Enthalpy= 0.754519
Thermal correction to Gibbs Free Energy= 0.623198
Sum of electronic and zero-point Energies= -2834.733015
Sum of electronic and thermal Energies= -2834.687092
Sum of electronic and thermal Enthalpies= -2834.686148
Sum of electronic and thermal Free Energies= -2834.817469

160 °C:
Zero-point correction= 0.709185 (Hartree/Particle)
Thermal correction to Energy= 0.801575
Thermal correction to Enthalpy= 0.802947
Thermal correction to Gibbs Free Energy= 0.560268
Sum of electronic and zero-point Energies= -2834.748459
Sum of electronic and thermal Energies= -2834.656068
Sum of electronic and thermal Enthalpies= -2834.654697
Sum of electronic and thermal Free Energies= -2834.897376

Decarbonylation from [1,1'-biphenyl]-4-CO-Pd$^{(II)}$-F

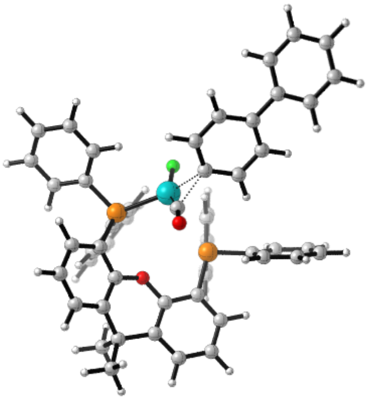

Pd	-0.6681	0.99483	-0.02849
P	0.25519	-1.96347	0.38622
P	1.48283	1.95347	0.15959
O	2.60433	-0.56263	-0.65045
C	2.52047	1.47997	1.58852
C	3.91141	1.35719	1.50902
H	4.42538	1.52262	0.56595
C	4.64457	1.00906	2.63801
H	5.72364	0.905	2.56861
C	3.99405	0.78923	3.85044
H	4.56629	0.51051	4.73075
C	2.61166	0.921	3.93114
H	2.10079	0.73533	4.87039
C	1.86817	1.26211	2.80429
H	0.78219	1.33833	2.84146
C	1.36385	3.77296	0.31084
C	2.48832	4.55077	0.61236
H	3.45197	4.07274	0.76556
C	2.37545	5.931	0.7266
H	3.25246	6.5265	0.96234
C	1.13688	6.54697	0.55031
H	1.04799	7.6249	0.64856
C	0.01311	5.77946	0.26323
H	-0.956	6.25345	0.14109
C	0.12533	4.39591	0.14548
H	-0.75597	3.79085	-0.04991
C	2.5875	1.69878	-1.27343
C	2.98936	2.70626	-2.14963
H	2.62548	3.71872	-2.00837
C	3.85404	2.41579	-3.20024
H	4.14981	3.20008	-3.88951
C	4.37212	1.13291	-3.34897
H	5.0837	0.94158	-4.14506
C	4.00674	0.10345	-2.48141
C	3.06766	0.40554	-1.49742
C	4.66387	-1.27423	-2.43464
C	3.6125	-2.28407	-1.9813
C	3.60781	-3.62812	-2.35038
H	4.35209	-4.00042	-3.04611
C	2.66559	-4.5103	-1.8317
H	2.68967	-5.55718	-2.1176
C	1.68618	-4.05217	-0.95869
H	0.9364	-4.74071	-0.58297
C	1.6335	-2.70608	-0.58484
C	2.62953	-1.86215	-1.08538
C	5.2911	-1.6595	-3.77817
H	4.53906	-1.71496	-4.57161
H	5.79682	-2.62661	-3.70718
H	6.0556	-0.93376	-4.07031
C	5.77343	-1.21938	-1.35491
H	5.35802	-0.94976	-0.37817
H	6.52952	-0.47424	-1.62646
H	6.25876	-2.19722	-1.26115
C	0.76612	-2.13715	2.13288
C	0.02441	-1.40985	3.07203
H	-0.7115	-0.67924	2.73871
C	0.27902	-1.56957	4.43134
H	-0.30484	-1.00542	5.15305

C	1.29385	-2.42084	4.86039
H	1.49644	-2.53655	5.92154
C	2.05794	-3.117	3.92626
H	2.85789	-3.77359	4.25638
C	1.78801	-2.98552	2.5672
H	2.37208	-3.54708	1.84326
C	-1.00642	-3.29443	0.21289
C	-1.59081	-3.46266	-1.05152
H	-1.26205	-2.84278	-1.88334
C	-2.58996	-4.40677	-1.25347
H	-3.03029	-4.52403	-2.23928
C	-3.03343	-5.19093	-0.18906
H	-3.81909	-5.92495	-0.34239
C	-2.46678	-5.02603	1.07031
H	-2.80785	-5.63238	1.90447
C	-1.45708	-4.08562	1.27161
H	-1.02131	-3.96955	2.25897
C	-0.99095	0.60097	-1.79325
O	-0.92316	0.31346	-2.91697
C	-2.64822	0.68647	-0.81977
C	-3.32719	-0.48138	-0.46681
C	-3.38152	1.83862	-1.1194
C	-4.7138	-0.48538	-0.38425
C	-4.76821	1.83003	-1.03669
C	-5.45496	0.66841	-0.66468
H	-2.77965	-1.38584	-0.23078
H	-2.86928	2.74607	-1.42793
H	-5.22548	-1.39109	-0.07063
H	-5.32739	2.72737	-1.28718
F	-1.06146	1.31471	1.93508
C	-6.93608	0.65989	-0.57049
C	-7.62112	1.73916	-0.0007
C	-7.67786	-0.42852	-1.04355
C	-9.00879	1.73029	0.09402
H	-7.05828	2.57989	0.39528
C	-9.06565	-0.43785	-0.94964
H	-7.16268	-1.26492	-1.50797
C	-9.73611	0.64151	-0.37997
H	-9.52192	2.57291	0.54818
H	-9.62471	-1.28849	-1.32846
H	-10.81943	0.63355	-0.30409

E(RM06L) = -3067.82042411
E(RM06) = -3066.35667342

25 °C:
Zero-point correction= 0.790093 (Hartree/Particle)
Thermal correction to Energy= 0.840573
Thermal correction to Enthalpy= 0.841517
Thermal correction to Gibbs Free Energy= 0.699939
Sum of electronic and zero-point Energies= -3065.626952
Sum of electronic and thermal Energies= -3065.576472
Sum of electronic and thermal Enthalpies= -3065.575528
Sum of electronic and thermal Free Energies= -3065.717106

160 °C:
Zero-point correction= 0.790093 (Hartree/Particle)
Thermal correction to Energy= 0.891610
Thermal correction to Enthalpy= 0.892982
Thermal correction to Gibbs Free Energy= 0.626252
Sum of electronic and zero-point Energies= -3065.626952
Sum of electronic and thermal Energies= -3065.525435
Sum of electronic and thermal Enthalpies= -3065.524063
Sum of electronic and thermal Free Energies= -3065.790793

Ar-[Pd$^{(II)}$]-F intermediates

Phenyl-Pd$^{(II)}$-F (cis)

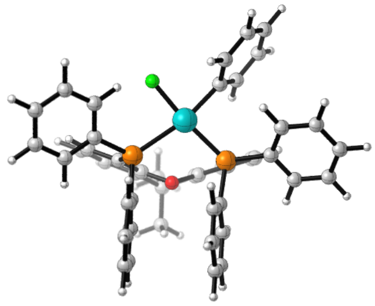

Pd	-0.34926	-1.37094	-0.66034
P	1.97069	-0.72244	-0.04368
P	-1.60721	0.01151	0.67134
O	0.44607	1.74803	-0.30005
C	-0.89236	0.63911	2.23825
C	-0.86567	1.99376	2.57484
H	-1.20033	2.74108	1.8617
C	-0.41971	2.396	3.82995
H	-0.39952	3.45275	4.08021
C	-0.00194	1.44842	4.75863
H	0.35032	1.76205	5.73684
C	-0.02246	0.09679	4.42673
H	0.321	-0.645	5.14045
C	-0.45966	-0.30567	3.17062
H	-0.45837	-1.36144	2.90941
C	-3.27684	-0.44241	1.28763
C	-4.02049	0.50711	2.0032
H	-3.61845	1.50377	2.16312
C	-5.26722	0.18365	2.52154
H	-5.83385	0.93069	3.06936
C	-5.78292	-1.10008	2.34671
H	-6.75693	-1.3543	2.75441
C	-5.04324	-2.05264	1.65632
H	-5.43559	-3.0552	1.51629
C	-3.79485	-1.72743	1.12885
H	-3.23592	-2.4725	0.57731
C	-1.89475	1.52997	-0.31565
C	-3.13486	1.97501	-0.7753
H	-4.03988	1.46151	-0.46834
C	-3.20668	3.04768	-1.65946
H	-4.17375	3.37941	-2.0237
C	-2.04952	3.69022	-2.09403
H	-2.134	4.51545	-2.79327
C	-0.79462	3.28191	-1.64487
C	-0.76018	2.20505	-0.76408
C	0.54179	3.93661	-1.99476
C	1.57187	2.81159	-2.11693
C	2.6229	2.76604	-3.03078
H	2.73923	3.55495	-3.7664
C	3.53044	1.70999	-3.01673
H	4.33953	1.68851	-3.73969
C	3.40177	0.6737	-2.09761
H	4.09988	-0.15586	-2.11746
C	2.35695	0.67809	-1.17182
C	1.47473	1.75655	-1.21352
C	0.45595	4.77856	-3.26986
H	0.1718	4.17208	-4.13594
H	1.41705	5.25778	-3.47898
H	-0.27565	5.58391	-3.15218
C	0.95277	4.85024	-0.81418
H	1.01928	4.28444	0.1206
H	0.21372	5.6479	-0.67957
H	1.92951	5.30567	-1.01016
C	2.49096	-0.13731	1.62311
C	2.67931	1.20421	1.95371
H	2.49206	1.98134	1.22081
C	3.1291	1.55999	3.22364
H	3.27225	2.60915	3.46467
C	3.40367	0.58156	4.17208

H	3.76697	0.86199	5.15652
C	3.20679	-0.76048	3.85414
H	3.41583	-1.5328	4.58867
C	2.74475	-1.11547	2.59361
H	2.60827	-2.16644	2.35178
C	3.33672	-1.90964	-0.37205
C	4.64386	-1.61131	0.04345
H	4.85249	-0.676	0.55533
C	5.67785	-2.50888	-0.18951
H	6.68606	-2.26687	0.13422
C	5.41774	-3.71969	-0.8313
H	6.22472	-4.42554	-1.00734
C	4.12553	-4.01761	-1.24756
H	3.91964	-4.95494	-1.75599
C	3.08329	-3.11728	-1.02678
H	2.07938	-3.30987	-1.39616
F	0.54387	-2.45922	-2.07654
C	-2.03869	-1.99494	-1.55283
C	-2.29636	-3.37056	-1.51327
C	-2.89104	-1.17709	-2.2944
C	-3.40951	-3.90492	-2.15667
H	-1.62021	-4.03041	-0.97591
C	-4.00163	-1.71454	-2.94907
H	-2.69842	-0.11137	-2.37832
C	-4.27277	-3.07588	-2.87178
H	-3.59774	-4.97465	-2.10701
H	-4.6533	-1.06104	-3.52406
H	-5.14132	-3.49237	-3.37432

E(RM06L) = -2723.36953056
E(RM06) = -2722.10294593

25 °C:
Zero-point correction= 0.701277 (Hartree/Particle)
Thermal correction to Energy= 0.744466
Thermal correction to Enthalpy= 0.745411
Thermal correction to Gibbs Free Energy= 0.624779
Sum of electronic and zero-point Energies= -2721.470056
Sum of electronic and thermal Energies= -2721.426866
Sum of electronic and thermal Enthalpies= -2721.425922
Sum of electronic and thermal Free Energies= -2721.546554
160 °C:
Zero-point correction= 0.701277 (Hartree/Particle)
Thermal correction to Energy= 0.789065
Thermal correction to Enthalpy= 0.790436
Thermal correction to Gibbs Free Energy= 0.561780
Sum of electronic and zero-point Energies= -2721.470057
Sum of electronic and thermal Energies= -2721.382269
Sum of electronic and thermal Enthalpies= -2721.380897
Sum of electronic and thermal Free Energies= -2721.609554

Phenyl-Pd$^{(II)}$-F (trans)

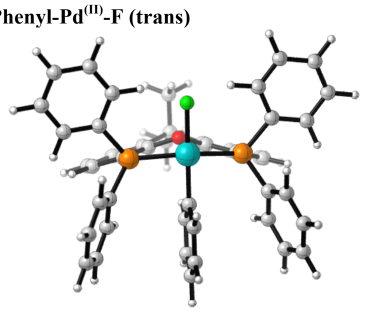

Pd	-0.00001	0.52325	1.06409
P	-2.25393	0.18745	0.56797
P	2.25392	0.18747	0.56801
O	0.	-1.70287	-0.42742
C	3.40989	-0.5018	1.80882
C	2.87134	-1.182	2.90579
H	1.78835	-1.21914	3.02617
C	3.72791	-1.76717	3.83721
H	3.3079	-2.28938	4.69186
C	5.10752	-1.67379	3.68598
H	5.76895	-2.12796	4.41855
C	5.64389	-0.98581	2.59843
H	6.72022	-0.89965	2.48131
C	4.79926	-0.40131	1.6625
H	5.22517	0.15086	0.82954
C	3.14959	1.62988	-0.11128
C	3.21537	1.91535	-1.47578
H	2.82073	1.21193	-2.20008
C	3.7564	3.11942	-1.91654
H	3.78814	3.33225	-2.98072
C	4.24126	4.04736	-1.0011
H	4.66137	4.98693	-1.34776
C	4.17945	3.76974	0.36253
H	4.55136	4.49032	1.08485
C	3.63105	2.57197	0.8058
H	3.57366	2.36993	1.87234
C	2.28851	-1.08729	-0.76755
C	3.47786	-1.31549	-1.46784
H	4.35833	-0.73026	-1.22234
C	3.54868	-2.25477	-2.48533
H	4.47937	-2.40411	-3.02308
C	2.4279	-3.01456	-2.80476
H	2.49933	-3.75465	-3.59418
C	1.23276	-2.85152	-2.11104
C	1.17867	-1.87676	-1.10882
C	0.00002	-3.73214	-2.30256
C	-1.23272	-2.85152	-2.11107
C	-2.42784	-3.01456	-2.80483
H	-2.49925	-3.75464	-3.59426
C	-3.54863	-2.25476	-2.48542
H	-4.47931	-2.4041	-3.0232
C	-3.47784	-1.31549	-1.46792
H	-4.35831	-0.73025	-1.22245
C	-2.2885	-1.0873	-0.7676
C	-1.17866	-1.87677	-1.10884
C	0.00004	-4.43778	-3.66169
H	0.00005	-3.72245	-4.49078
H	-0.8749	-5.08687	-3.75912
H	0.87499	-5.08687	-3.7591
C	0.	-4.80497	-1.18486
H	-0.00002	-4.34431	-0.19279
H	0.89085	-5.43684	-1.26987
H	-0.89086	-5.43683	-1.2699
C	-3.40992	-0.50183	1.80875
C	-4.79929	-0.40141	1.66235
H	-5.22518	0.15069	0.82934
C	-5.64393	-0.98591	2.59827
H	-6.72027	-0.89981	2.48108
C	-5.10759	-1.6738	3.68588
H	-5.76904	-2.12796	4.41844
C	-3.72798	-1.7671	3.8372
H	-3.308	-2.28922	4.69191
C	-2.87139	-1.18194	2.90579
H	-1.78841	-1.219	3.02624
C	-3.1496	1.62987	-0.1113
C	-3.21521	1.91547	-1.47579
H	-2.82051	1.21212	-2.20011
C	-3.7562	3.11958	-1.9165
H	-3.7878	3.3325	-2.98067
C	-4.24118	4.04743	-1.00104
H	-4.66125	4.98703	-1.34767
C	-4.17955	3.76969	0.36256
H	-4.55156	4.49019	1.0849
C	-3.63118	2.57188	0.8058
H	-3.57393	2.36974	1.87233
F	-0.00002	-0.63373	2.75203
C	-0.00004	1.75975	-0.52714
C	0.00004	1.35252	-1.86533
C	-0.00007	3.13651	-0.26632
C	0.00005	2.28169	-2.90685
H	0.00014	0.29535	-2.11575
C	-0.00006	4.06695	-1.30097
H	-0.00013	3.48951	0.76259
C	0.00001	3.64363	-2.62939
H	0.00013	1.93323	-3.9373
H	-0.00011	5.12903	-1.06975
H	0.00003	4.37104	-3.43649

E(RM06L) = -2723.36938190
E(RM06) = -2722.10276989

25 °C:
Zero-point correction= 0.701244 (Hartree/Particle)
Thermal correction to Energy= 0.744365
Thermal correction to Enthalpy= 0.745309
Thermal correction to Gibbs Free Energy= 0.625226
Sum of electronic and zero-point Energies= -2721.473024
Sum of electronic and thermal Energies= -2721.429903
Sum of electronic and thermal Enthalpies= -2721.428959
Sum of electronic and thermal Free Energies= -2721.549042

160 °C:
Zero-point correction= 0.701249 (Hartree/Particle)
Thermal correction to Energy= 0.788938
Thermal correction to Enthalpy= 0.790310
Thermal correction to Gibbs Free Energy= 0.562507
Sum of electronic and zero-point Energies= -2721.473019
Sum of electronic and thermal Energies= -2721.385330
Sum of electronic and thermal Enthalpies= -2721.383959
Sum of electronic and thermal Free Energies= -2721.611762

[1,1'-Biphenyl]-4-Pd$^{(II)}$-F

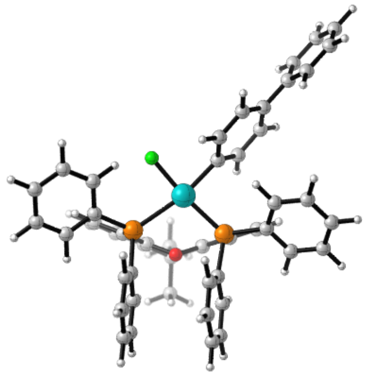

Pd	-0.23462	1.01442	-0.15709
P	2.21932	1.40571	-0.14279
P	-0.36625	-1.19701	-0.77108
O	1.78865	-1.18803	1.11173
C	0.9801	-1.92941	-1.77472
C	1.6372	-3.11225	-1.43173
H	1.42955	-3.59897	-0.48336
C	2.55805	-3.68033	-2.30722
H	3.06557	-4.60026	-2.03177
C	2.82554	-3.07358	-3.53008
H	3.54639	-3.51518	-4.21173
C	2.17822	-1.89015	-3.87335
H	2.39822	-1.40142	-4.81695
C	1.26379	-1.31836	-2.99769
H	0.77029	-0.38603	-3.2621
C	-1.80251	-1.88575	-1.68721
C	-1.87936	-3.26932	-1.90208
H	-1.10839	-3.92569	-1.50762
C	-2.93344	-3.81504	-2.62319
H	-2.98494	-4.88911	-2.77445
C	-3.91737	-2.98327	-3.15635
H	-4.74364	-3.40937	-3.71774
C	-3.8338	-1.60874	-2.97057
H	-4.59297	-0.95167	-3.38357
C	-2.78088	-1.06036	-2.24127
H	-2.7374	0.01032	-2.0887
C	-0.32978	-2.15786	0.79116
C	-1.37368	-2.94168	1.28538
H	-2.26381	-3.09758	0.68527
C	-1.29068	-3.48975	2.56216
H	-2.11181	-4.08861	2.9428
C	-0.17212	-3.26544	3.36118
H	-0.13869	-3.69698	4.35596
C	0.89442	-2.49711	2.89733
C	0.7779	-1.96506	1.61642
C	2.19453	-2.22003	3.65232
C	2.60588	-0.78466	3.31889
C	3.20665	0.11308	4.19919
H	3.3831	-0.17068	5.23136
C	3.58167	1.38406	3.77135
H	4.04241	2.07271	4.4723
C	3.35625	1.78752	2.45929
H	3.62515	2.7911	2.14862
C	2.75	0.9196	1.54906
C	2.39522	-0.34522	2.01467
C	2.04208	-2.4387	5.15947
H	1.27989	-1.77977	5.58757
H	2.99113	-2.25426	5.67163
H	1.76962	-3.47666	5.37364
C	3.2819	-3.17937	3.11046
H	3.41866	-3.05351	2.03172
H	2.9984	-4.22025	3.30153
H	4.23989	-2.98081	3.60313
C	3.40755	0.57076	-1.27445
C	4.17619	-0.53732	-0.91965
H	4.07706	-0.98155	0.06465
C	5.09048	-1.07763	-1.82169
H	5.68064	-1.94162	-1.5306
C	5.25333	-0.51374	-3.08172
H	5.9764	-0.92974	-3.77729
C	4.48037	0.58549	-3.4489
H	4.59804	1.03392	-4.43113
C	3.55849	1.11611	-2.55621
H	2.97248	1.98407	-2.84756
C	2.84379	3.12939	-0.29348
C	4.22393	3.3687	-0.38427
H	4.92491	2.53909	-0.35866
C	4.70641	4.66417	-0.51684
H	5.7764	4.83682	-0.58714
C	3.81579	5.73645	-0.56903
H	4.19291	6.74925	-0.68079
C	2.44878	5.5049	-0.47406
H	1.75102	6.33661	-0.50391
C	1.95623	4.20741	-0.33149
H	0.89457	4.0145	-0.20263
F	-0.33106	2.84582	0.62867
C	-2.22617	1.02523	0.09659
C	-2.9366	1.98796	-0.63084
C	-2.93648	0.21471	0.98114
C	-4.31867	2.08859	-0.52378
H	-2.40298	2.67011	-1.28697
C	-4.32178	0.32309	1.09619
H	-2.42069	-0.52282	1.58939
C	-5.03926	1.24691	0.33327
H	-4.84581	2.85167	-1.09185
H	-4.85468	-0.34386	1.77008
C	-6.51697	1.34135	0.43122
C	-7.30003	1.53632	-0.71316
C	-7.16587	1.23686	1.66731
C	-8.68538	1.62712	-0.62553
H	-6.81531	1.59996	-1.68371
C	-8.55147	1.32233	1.75658
H	-6.57411	1.11195	2.56992
C	-9.31766	1.51895	0.61037
H	-9.27257	1.7753	-1.52754
H	-9.03378	1.24499	2.72691
H	-10.39911	1.59058	0.68088

E(RM06L) = -2954.47528680
E(RM06) = -2953.05785099

25 °C:
Zero-point correction= 0.782982 (Hartree/Particle)
Thermal correction to Energy= 0.830951
Thermal correction to Enthalpy= 0.831895
Thermal correction to Gibbs Free Energy= 0.699166
Sum of electronic and zero-point Energies= -2952.366296
Sum of electronic and thermal Energies= -2952.318326
Sum of electronic and thermal Enthalpies= -2952.317382
Sum of electronic and thermal Free Energies= -2952.450112

160 °C:
Zero-point correction= 0.782980 (Hartree/Particle)
Thermal correction to Energy= 0.880565
Thermal correction to Enthalpy= 0.881937
Thermal correction to Gibbs Free Energy= 0.629753
Sum of electronic and zero-point Energies= -2952.366298
Sum of electronic and thermal Energies= -2952.268713
Sum of electronic and thermal Enthalpies= -2952.267342
Sum of electronic and thermal Free Energies= -2952.519525

ArCO-[Pd$^{(II)}$]-CF$_3$ intermediates

PhenylCO-Pd$^{(II)}$-CF$_3$ (cis)

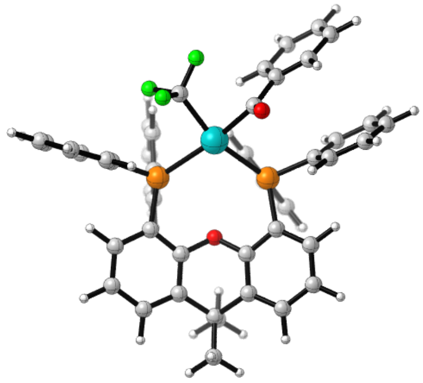

P	-1.83244	-1.14322	-0.01661
P	1.24043	1.2086	-0.16209
C	-3.1196	0.10581	-0.45644
C	-1.91774	-1.20451	1.816
C	-2.72853	-2.65026	-0.55135
C	0.43317	2.71888	-0.83987
C	1.07009	1.50883	1.64031
C	3.0081	1.5826	-0.48423
C	-2.79863	1.45973	-0.42742
O	-1.53913	1.7798	0.00641
C	-0.94132	2.82426	-0.64896
C	-4.41947	-0.23012	-0.85167
C	-5.3208	0.75691	-1.231
C	-4.94969	2.09873	-1.21734
C	-3.67593	2.47759	-0.8032
C	1.07481	3.78802	-1.4691
C	0.34115	4.88372	-1.91351
C	-1.03498	4.94642	-1.7133
C	-1.70385	3.91586	-1.05645
C	1.03669	2.80372	2.17
C	0.92883	2.99821	3.54224
C	0.86245	1.90232	4.40093
C	0.90247	0.61291	3.88208
C	0.99999	0.41811	2.50741
C	3.94101	1.71223	0.54601
C	5.28301	1.93923	0.25347
C	5.70324	2.05131	-1.06757
C	4.77929	1.91708	-2.10118
C	3.44259	1.6709	-1.81262
C	-2.6218	-0.27229	2.57874
C	-2.62075	-0.35715	3.9694
C	-1.9305	-1.38112	4.60886
C	-1.22286	-2.31473	3.85303
C	-1.20522	-2.22191	2.46624
C	-3.48334	-3.44637	0.31103
C	-4.19418	-4.53611	-0.18572
C	-4.16506	-4.83039	-1.54577
C	-3.42077	-4.0325	-2.4113
C	-2.70404	-2.94998	-1.91621
H	-4.72331	-1.26976	-0.88253
H	-6.32136	0.47765	-1.54558
H	2.14806	3.76699	-1.61719
H	0.84994	5.70199	-2.4132
H	-1.58225	5.81543	-2.06303
H	1.09295	3.66287	1.50763
H	0.89807	4.00723	3.94294
H	0.77521	2.05604	5.47252
H	0.83942	-0.24671	4.54077
H	1.00411	-0.58878	2.10424
H	3.62778	1.61995	1.58065
H	6.00148	2.02117	1.0632
H	6.75091	2.2281	-1.29233
H	5.1007	1.9847	-3.1361
H	2.73704	1.52751	-2.62597
H	-3.17896	0.52319	2.09433
H	-3.16946	0.37715	4.5516
H	-1.94109	-1.45234	5.69272
H	-0.68125	-3.11842	4.34356
H	-0.65005	-2.94644	1.87852
H	-3.5197	-3.22068	1.37217
H	-4.77405	-5.15441	0.49322
H	-4.71719	-5.6828	-1.93041
H	-3.38534	-4.25995	-3.47225
H	-2.10965	-2.34247	-2.59206
H	-5.66742	2.85024	-1.52851
C	-3.182	3.91723	-0.67441
C	0.62144	-3.15232	-0.98009
F	-0.16285	-3.8352	-0.07118
F	1.82587	-3.78547	-0.86913
F	0.1515	-3.53589	-2.20002
Pd	0.59127	-1.08193	-0.72139
C	-3.29312	4.33903	0.81154
H	-2.9173	5.3597	0.943
H	-4.33893	4.30434	1.13612
H	-2.70882	3.67493	1.45613
C	-3.99886	4.88713	-1.53185
H	-5.04765	4.89083	-1.22028
H	-3.63422	5.91105	-1.40619
H	-3.95015	4.62729	-2.59429
C	2.45173	-1.3024	-1.47728
O	2.59147	-1.17688	-2.67289
C	3.63457	-1.5381	-0.57613
C	4.91835	-1.41284	-1.1079
C	3.47539	-1.81518	0.78018
C	6.03023	-1.53714	-0.28344
H	5.02401	-1.19773	-2.16598
C	4.58501	-1.94275	1.60678
H	2.47315	-1.94423	1.17872
C	5.86502	-1.79529	1.07575
H	7.02725	-1.4237	-0.69947
H	4.45285	-2.1586	2.66304
H	6.73419	-1.88717	1.72127

E(RM06L) = -3074.59401873
E(RM06) = -3073.23579684

25 °C:
Zero-point correction= 0.723159 (Hartree/Particle)
Thermal correction to Energy= 0.771058
Thermal correction to Enthalpy= 0.772002
Thermal correction to Gibbs Free Energy= 0.641033
Sum of electronic and zero-point Energies= -3072.474452
Sum of electronic and thermal Energies= -3072.426553
Sum of electronic and thermal Enthalpies= -3072.425609
Sum of electronic and thermal Free Energies= -3072.556578

160 °C:
Zero-point correction= 0.723174 (Hartree/Particle)
Thermal correction to Energy= 0.819707
Thermal correction to Enthalpy= 0.821079
Thermal correction to Gibbs Free Energy= 0.572622
Sum of electronic and zero-point Energies= -3072.474437
Sum of electronic and thermal Energies= -3072.377904
Sum of electronic and thermal Enthalpies= -3072.376532
Sum of electronic and thermal Free Energies= -3072.624989

PhenylCO-Pd(II)-CF$_3$ (trans)

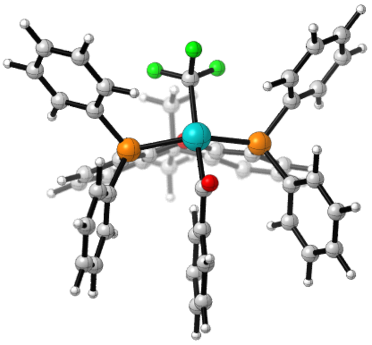

Pd	0.01226	0.08838	-1.13082
P	2.2403	0.01926	-0.43967
P	-2.22231	-0.05868	-0.46671
O	0.02299	-1.48452	1.06368
C	-3.32853	-0.96357	-1.61197
C	-3.07273	-0.79027	-2.97603
H	-2.20986	-0.20901	-3.29225
C	-3.8946	-1.38307	-3.92864
H	-3.68324	-1.2409	-4.98395
C	-4.96709	-2.17364	-3.52718
H	-5.60346	-2.64685	-4.26913
C	-5.21151	-2.37095	-2.17079
H	-6.03545	-3.00252	-1.85183
C	-4.39931	-1.76664	-1.21634
H	-4.59378	-1.94757	-0.16513
C	-3.13011	1.51096	-0.16483
C	-3.2072	2.1077	1.09661
H	-2.81938	1.5954	1.96961
C	-3.75146	3.37916	1.24645
H	-3.78733	3.83188	2.23252
C	-4.22741	4.07154	0.13789
H	-4.64815	5.06591	0.2543
C	-4.15724	3.48452	-1.12272
H	-4.52305	4.01697	-1.99536
C	-3.60979	2.21577	-1.27429
H	-3.54864	1.77802	-2.26595
C	-2.2864	-0.91932	1.15984
C	-3.47695	-0.95398	1.89761
H	-4.35686	-0.45159	1.50757
C	-3.54311	-1.57772	3.13536
H	-4.47537	-1.58193	3.69077
C	-2.41593	-2.20449	3.65871
H	-2.48753	-2.70272	4.6194
C	-1.21199	-2.20541	2.96171
C	-1.16291	-1.53649	1.73485
C	0.03554	-2.97901	3.38263
C	1.25613	-2.15327	2.98186
C	2.44601	-2.09817	3.70044
H	2.52155	-2.59111	4.66352
C	3.55358	-1.42245	3.19638
H	4.47457	-1.38379	3.769
C	3.48294	-0.80545	1.95552
H	4.34666	-0.2655	1.57967
C	2.30617	-0.82607	1.19559
C	1.19973	-1.48866	1.75302
C	0.0317	-3.31914	4.87512
H	0.00381	-2.41884	5.49837
H	0.9206	-3.89935	5.13964
H	-0.8293	-3.94534	5.12641
C	0.06929	-4.29589	2.56534
H	0.07367	-4.09835	1.489
H	-0.81104	-4.90438	2.7999
H	0.97033	-4.86771	2.81328
C	3.38697	-0.86461	-1.56033
C	4.47954	-1.62534	-1.14135
H	4.66668	-1.7864	-0.08542
C	5.32471	-2.2118	-2.07822
H	6.16628	-2.81001	-1.74146
C	5.09165	-2.03808	-3.43993
H	5.75432	-2.49634	-4.16812
C	3.99833	-1.28911	-3.86433
H	3.79675	-1.16505	-4.92382
C	3.1438	-0.71447	-2.92936
H	2.26675	-0.16515	-3.26286
C	3.10843	1.60968	-0.13325
C	3.07676	2.24428	1.1118
H	2.62899	1.75007	1.96635
C	3.59855	3.52404	1.26823
H	3.54881	4.00522	2.24018
C	4.16375	4.18611	0.18319
H	4.56908	5.18631	0.30435
C	4.20604	3.55987	-1.05953
H	4.64563	4.06698	-1.91303
C	3.67895	2.28304	-1.21874
H	3.71156	1.81238	-2.19649
C	0.05161	-1.95326	-1.72031
F	1.13166	-2.70118	-1.2921
F	-1.01652	-2.735	-1.32469
F	0.07454	-2.09253	-3.08889
C	-0.02725	2.10979	-0.92327
O	-0.01121	2.72003	-1.97852
C	-0.09228	2.86752	0.37725
C	-0.06797	2.19016	1.5947
C	-0.19224	4.25914	0.36858
C	-0.13706	2.88652	2.79599
H	0.0065	1.10568	1.5917
C	-0.26721	4.96075	1.56571
H	-0.21395	4.76961	-0.58895
C	-0.23853	4.27599	2.78088
H	-0.1149	2.34842	3.73966
H	-0.35013	6.04394	1.5562
H	-0.29678	4.82613	3.71615

E(RM06L) = -3074.58168799
E(RM06) = -3073.21950490

25 °C:
Zero-point correction= 0.722502 (Hartree/Particle)
Thermal correction to Energy= 0.770650
Thermal correction to Enthalpy= 0.771595
Thermal correction to Gibbs Free Energy= 0.638586
Sum of electronic and zero-point Energies= -3072.460836
Sum of electronic and thermal Energies= -3072.412688
Sum of electronic and thermal Enthalpies= -3072.411744
Sum of electronic and thermal Free Energies= -3072.544753

160 °C:
Zero-point correction= 0.722469 (Hartree/Particle)
Thermal correction to Energy= 0.819294
Thermal correction to Enthalpy= 0.820666
Thermal correction to Gibbs Free Energy= 0.569136
Sum of electronic and zero-point Energies= -3072.460869
Sum of electronic and thermal Energies= -3072.364044
Sum of electronic and thermal Enthalpies= -3072.362673
Sum of electronic and thermal Free Energies= -3072.614202

[1,1'-Biphenyl]-4-CO-Pd(II)-CF$_3$

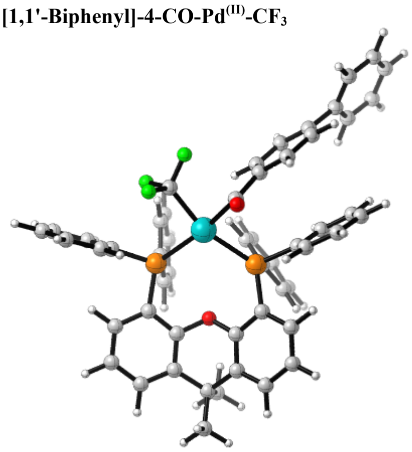

P	-2.37065	-1.35776	0.15342
P	0.28297	1.39699	-0.44514

C	-3.87219	-0.29459	-0.00538
C	-2.10871	-1.44437	1.96819
C	-3.13966	-2.96896	-0.26286
C	-0.828	2.79324	-0.90358
C	0.4056	1.63934	1.37055
C	1.89971	2.02074	-1.05382
C	-3.73468	1.0899	0.0189
O	-2.47189	1.57426	0.23726
C	-2.14689	2.70281	-0.4686
C	-5.16475	-0.80183	-0.17965
C	-6.24287	0.05686	-0.35713
C	-6.05787	1.43654	-0.35572
C	-4.79295	1.98213	-0.15577
C	-0.45935	3.95205	-1.59088
C	-1.40124	4.94242	-1.85249
C	-2.71422	4.8091	-1.41009
C	-3.10953	3.68462	-0.68899
C	0.29334	2.90817	1.95006
C	0.423	3.06641	3.32514
C	0.67443	1.96092	4.1359
C	0.7921	0.69785	3.56656
C	0.65229	0.53809	2.19092
C	2.97488	2.2561	-0.19451
C	4.20186	2.67456	-0.70194
C	4.36158	2.87891	-2.06827
C	3.29611	2.64004	-2.93295
C	2.07743	2.19821	-2.43141
C	-2.77055	-0.61486	2.87393
C	-2.49991	-0.70454	4.23756
C	-1.57802	-1.63229	4.70986
C	-0.91171	-2.46337	3.81052
C	-1.1644	-2.36314	2.44726
C	-3.6039	-3.8727	0.69366
C	-4.24389	-5.04419	0.29662
C	-4.43484	-5.31435	-1.05523
C	-3.98073	-4.41085	-2.01302
C	-3.33279	-3.2464	-1.61905
H	-5.32822	-1.87286	-0.19584
H	-7.23653	-0.3546	-0.50297
H	0.56258	4.08357	-1.92644
H	-1.10514	5.83185	-2.39959
H	-3.42803	5.59821	-1.62184
H	0.10424	3.77632	1.32522
H	0.32921	4.05503	3.76488
H	0.7765	2.0866	5.20982
H	0.97225	-0.17219	4.18909
H	0.72118	-0.45226	1.7548
H	2.86418	2.09766	0.87291
H	5.03594	2.83101	-0.02448
H	5.31921	3.20674	-2.46176
H	3.41832	2.77656	-4.00307
H	1.26721	1.97147	-3.11862
H	-3.50519	0.10304	2.52395
H	-3.01953	-0.04974	4.93066
H	-1.37762	-1.70877	5.77466
H	-0.18957	-3.19105	4.16942
H	-0.64022	-3.00656	1.7475
H	-3.46712	-3.66844	1.75084
H	-4.59462	-5.74587	1.04769
H	-4.93281	-6.22924	-1.3625
H	-4.1192	-4.61753	-3.06973
H	-2.96453	-2.55412	-2.3704
H	-6.91292	2.08714	-0.50587
C	-4.48189	3.47378	-0.0548
C	0.11291	-2.99749	-1.30224
F	-0.38806	-3.78689	-0.28565
F	1.393	-3.45538	-1.43107
F	-0.51635	-3.43898	-2.42648
Pd	-0.15053	-0.95439	-0.97605
C	-4.38265	3.84914	1.44438
H	-4.13263	4.91031	1.5522
H	-5.33988	3.66217	1.94333
H	-3.60904	3.26255	1.94981
C	-5.56216	4.33498	-0.71387
H	-6.52904	4.1819	-0.22521
H	-5.3263	5.39797	-0.6066
H	-5.66938	4.1063	-1.77912
C	1.5457	-0.90822	-2.07017
O	1.4438	-0.75388	-3.26639
C	2.89278	-0.96666	-1.40462
C	4.02633	-0.64054	-2.14841
C	3.02749	-1.24048	-0.0455
C	5.26395	-0.54102	-1.53012
H	3.90974	-0.4258	-3.20541
C	4.26375	-1.14174	0.57497
H	2.15078	-1.53772	0.52233
C	5.3986	-0.77	-0.15594
H	6.1317	-0.23489	-2.10825
H	4.35812	-1.36563	1.63397
C	6.71099	-0.59992	0.51438
C	6.79886	0.05016	1.751
C	7.88821	-1.07312	-0.07618
C	8.02795	0.2214	2.37962
H	5.89579	0.4423	2.21141
C	9.11757	-0.90276	0.55172
H	7.83334	-1.59846	-1.02563
C	9.19217	-0.25467	1.78215
H	8.0774	0.73358	3.33624
H	10.01916	-1.28557	0.08238
H	10.152	-0.12167	2.27238

E(RM06L) = -3305.70008287
E(RM06) = -3304.19064434

25 °C:
Zero-point correction= 0.806580 (Hartree/Particle)
Thermal correction to Energy= 0.858902
Thermal correction to Enthalpy= 0.859847
Thermal correction to Gibbs Free Energy= 0.719032
Sum of electronic and zero-point Energies= -3303.370093
Sum of electronic and thermal Energies= -3303.317770
Sum of electronic and thermal Enthalpies= -3303.316826
Sum of electronic and thermal Free Energies= -3303.457641

160 °C:
Zero-point correction= 0.806607 (Hartree/Particle)
Thermal correction to Energy= 0.912414
Thermal correction to Enthalpy= 0.913785
Thermal correction to Gibbs Free Energy= 0.645304
Sum of electronic and zero-point Energies= -3303.370066
Sum of electronic and thermal Energies= -3303.264259
Sum of electronic and thermal Enthalpies= -3303.262887
Sum of electronic and thermal Free Energies= -3303.531368

Decarbonylation from ArCO-[Pd$^{(II)}$]-CF_3 transition states

Decarbonylation from phenylCO-Pd$^{(II)}$-CF_3

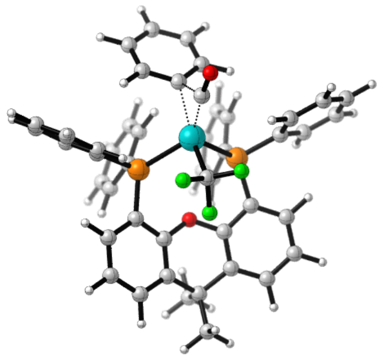

P	1.79477	-0.73495	0.05131
P	-1.76286	0.63138	0.07692
O	0.72807	1.90702	0.59506
C	-1.73884	0.35645	1.8877
C	-1.97697	1.39098	2.79794

H	-2.14159	2.40241	2.43737
C	-2.00803	1.13164	4.16477
H	-2.19024	1.94306	4.86357
C	-1.81386	-0.16507	4.63392
H	-1.84222	-0.36719	5.70084
C	-1.57254	-1.19793	3.73338
H	-1.39878	-2.2081	4.08929
C	-1.52207	-0.9379	2.36859
H	-1.30072	-1.74399	1.6788
C	-3.53622	0.475	-0.36062
C	-4.54665	0.29772	0.58626
H	-4.30656	0.29066	1.64494
C	-5.86623	0.11538	0.17662
H	-6.64416	-0.0292	0.92069
C	-6.18614	0.11781	-1.1776
H	-7.21481	-0.02807	-1.49404
C	-5.18258	0.30238	-2.12742
H	-5.42376	0.30175	-3.18618
C	-3.86273	0.47183	-1.72364
H	-3.07784	0.59489	-2.46532
C	-1.45994	2.42739	-0.07165
C	-2.39555	3.37431	-0.48592
H	-3.41842	3.07256	-0.68686
C	-2.01149	4.69991	-0.6666
H	-2.74077	5.43111	-1.00025
C	-0.69743	5.09449	-0.42895
H	-0.42307	6.13221	-0.58673
C	0.25841	4.17828	0.00835
C	-0.15981	2.86279	0.17557
C	1.70647	4.50834	0.36725
C	2.56148	3.30552	-0.03485
C	3.86672	3.37448	-0.51767
H	4.33408	4.339	-0.68507
C	4.58697	2.21525	-0.79108
H	5.60121	2.28894	-1.1706
C	4.01634	0.96345	-0.58783
H	4.58514	0.06959	-0.82006
C	2.70763	0.85161	-0.11461
C	2.01762	2.03586	0.14177
C	2.18274	5.80023	-0.30242
H	2.13365	5.72746	-1.39357
H	3.21261	6.02955	-0.01295
H	1.57316	6.64994	0.01964
C	1.79729	4.67329	1.9039
H	1.44979	3.77156	2.41829
H	1.17545	5.51388	2.23157
H	2.83383	4.8633	2.20346
C	1.80455	-1.04489	1.8698
C	1.84514	-0.01421	2.81311
H	1.929	1.01808	2.49571
C	1.78038	-0.2946	4.17471
H	1.80728	0.52238	4.88944
C	1.68064	-1.60825	4.62017
H	1.6314	-1.82327	5.68361
C	1.63932	-2.64367	3.69083
H	1.56355	-3.67533	4.02279
C	1.69516	-2.36337	2.33054
H	1.67693	-3.18444	1.62273
C	3.03118	-1.95652	-0.54472
C	4.13677	-2.35161	0.22014
H	4.27837	-1.94063	1.2155
C	5.05216	-3.26811	-0.28471
H	5.90359	-3.56828	0.31922
C	4.87746	-3.80011	-1.56162
H	5.591	-4.51986	-1.95213
C	3.7888	-3.40681	-2.33302
H	3.64704	-3.81445	-3.32926
C	2.87016	-2.48919	-1.82735
H	2.0265	-2.17902	-2.43682
O	-0.90551	-3.13289	-3.32296
Pd	-0.45434	-0.89415	-1.34958
C	-0.78154	-2.36402	-2.45934
C	-1.27823	-2.83761	-0.6562
C	-0.42742	-3.75786	-0.03774
C	-2.65094	-2.89409	-0.39242
C	-0.92477	-4.66018	0.89876
H	0.62979	-3.76444	-0.29131
C	-3.15046	-3.7966	0.54214
H	-3.3292	-2.21644	-0.90297
C	-2.28579	-4.67024	1.19913
H	-0.252	-5.35741	1.39083
H	-4.21464	-3.81477	0.75888
H	-2.67458	-5.37234	1.93112
C	0.06296	0.49531	-2.78713
F	-0.99782	0.87641	-3.57122
F	0.59179	1.67478	-2.34855
F	0.99157	0.02156	-3.67193

E(RM06L) = -3074.55977449
E(RM06) = -3073.19938991

25 °C:
Zero-point correction= 0.719645 (Hartree/Particle)
Thermal correction to Energy= 0.768126
Thermal correction to Enthalpy= 0.769070
Thermal correction to Gibbs Free Energy= 0.635613
Sum of electronic and zero-point Energies= -3072.445054
Sum of electronic and thermal Energies= -3072.396573
Sum of electronic and thermal Enthalpies= -3072.395629
Sum of electronic and thermal Free Energies= -3072.529086

160 °C:
Zero-point correction= 0.719645 (Hartree/Particle)
Thermal correction to Energy= 0.816763
Thermal correction to Enthalpy= 0.818135
Thermal correction to Gibbs Free Energy= 0.566033
Sum of electronic and zero-point Energies= -3072.445054
Sum of electronic and thermal Energies= -3072.347936
Sum of electronic and thermal Enthalpies= -3072.346565
Sum of electronic and thermal Free Energies= -3072.598666

Decarbonylation from phenylCO-Pd$^{(II)}$-CF_3 – alternative transition state (with similar energy)

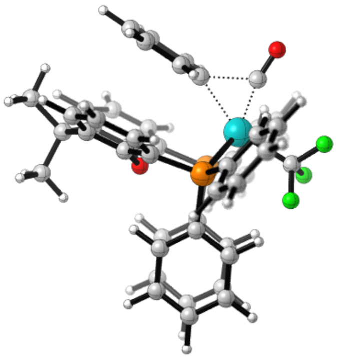

P	1.9417	-0.31361	0.16762
P	-1.83832	-0.72674	0.12714
O	-0.195	1.34826	1.20213
C	-1.66519	-1.43871	1.80513
C	-2.15542	-0.80425	2.94992
H	-2.64704	0.16085	2.86855
C	-2.02236	-1.40578	4.19826
H	-2.40723	-0.90406	5.08159
C	-1.40392	-2.64696	4.31197
H	-1.29644	-3.11509	5.28617
C	-0.9101	-3.28157	3.1756
H	-0.40867	-4.24028	3.25979
C	-1.03325	-2.68029	1.92847
H	-0.62667	-3.17239	1.05217
C	-3.37333	-1.4997	-0.50632
C	-4.41434	-1.90084	0.33722
H	-4.30873	-1.79669	1.41342
C	-5.58253	-2.43612	-0.19576
H	-6.38342	-2.749	0.46774
C	-5.72163	-2.57507	-1.57527
H	-6.63226	-2.99752	-1.98987
C	-4.68705	-2.18425	-2.42057
H	-4.78377	-2.30627	-3.49507
C	-3.51563	-1.65414	-1.88773

H	-2.69435	-1.37842	-2.54442
C	-2.38892	0.98977	0.46192
C	-3.65653	1.49794	0.17813
H	-4.4359	0.83273	-0.17934
C	-3.91552	2.85717	0.32886
H	-4.90267	3.24667	0.10016
C	-2.9189	3.72253	0.77071
H	-3.14489	4.77826	0.87557
C	-1.64564	3.24819	1.08446
C	-1.42212	1.8859	0.91743
C	-0.51054	4.09213	1.66036
C	0.80757	3.51847	1.14134
C	1.953	4.26791	0.8806
H	1.93339	5.34674	0.99162
C	3.13352	3.65047	0.47917
H	4.01697	4.25049	0.28418
C	3.19023	2.26982	0.32118
H	4.11108	1.80634	-0.01537
C	2.06226	1.48222	0.55989
C	0.90058	2.1397	0.96916
C	-0.6653	5.57309	1.30268
H	-0.65167	5.72139	0.21802
H	0.13778	6.16676	1.74903
H	-1.60219	5.97365	1.70075
C	-0.53003	3.93435	3.20008
H	-0.43338	2.88231	3.48763
H	-1.47218	4.31578	3.60922
H	0.30029	4.49028	3.64935
C	2.06275	-1.13064	1.81156
C	1.63834	-0.51102	2.98989
H	1.2315	0.49306	2.968
C	1.72929	-1.17518	4.20904
H	1.38791	-0.68028	5.11321
C	2.2483	-2.46397	4.27038
H	2.32322	-2.97783	5.22434
C	2.66511	-3.09242	3.10032
H	3.07033	-4.09958	3.13469
C	2.56719	-2.43521	1.87905
H	2.89495	-2.93746	0.97556
C	3.59081	-0.66715	-0.55402
C	4.76049	-0.6797	0.21808
H	4.70053	-0.5091	1.2894
C	5.99465	-0.91305	-0.37675
H	6.89442	-0.92058	0.23158
C	6.07664	-1.14068	-1.75036
H	7.04129	-1.32605	-2.21357
C	4.9208	-1.13908	-2.52346
H	4.97545	-1.32943	-3.59093
C	3.68312	-0.90623	-1.92707
H	2.77898	-0.92855	-2.52919
O	0.11543	-0.32058	-4.50442
Pd	0.05389	-0.75036	-1.52412
C	0.07601	-0.31859	-3.34029
C	-0.15296	1.30188	-2.37832
C	0.28107	-2.79709	-1.60839
C	0.96684	2.14019	-2.37981
C	-1.42724	1.87326	-2.45958
C	0.81514	3.52293	-2.39042
H	1.9642	1.70967	-2.36571
C	-1.58266	3.25543	-2.47208
H	-2.30461	1.23413	-2.50551
C	-0.46074	4.08081	-2.42931
H	1.69266	4.16177	-2.36375
H	-2.57818	3.68671	-2.51014
F	0.83116	-3.26168	-2.76494
F	1.0892	-3.32158	-0.62299
F	-0.88965	-3.49541	-1.4755
H	-0.58127	5.16073	-2.44357

E(RM06L) = -3074.56256349
E(RM06) = -3073.20125389

25 °C:
Zero-point correction= 0.720979 (Hartree/Particle)
Thermal correction to Energy= 0.768949
Thermal correction to Enthalpy= 0.769893
Thermal correction to Gibbs Free Energy= 0.639559
Sum of electronic and zero-point Energies= -3072.449155
Sum of electronic and thermal Energies= -3072.401185
Sum of electronic and thermal Enthalpies= -3072.400241
Sum of electronic and thermal Free Energies= -3072.530575

160 °C:
Zero-point correction= 0.720979 (Hartree/Particle)
Thermal correction to Energy= 0.817471
Thermal correction to Enthalpy= 0.818842
Thermal correction to Gibbs Free Energy= 0.571417
Sum of electronic and zero-point Energies= -3072.449155
Sum of electronic and thermal Energies= -3072.352663
Sum of electronic and thermal Enthalpies= -3072.351292
Sum of electronic and thermal Free Energies= -3072.598717

Decarbonylation from [1,1'-biphenyl]-4-CO-Pd$^{(II)}$-CF_3

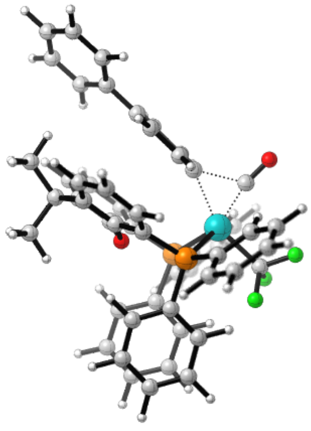

P	-1.39409	-1.79797	0.09572
P	-0.89471	1.97819	0.02388
O	0.41114	-0.06748	1.54144
C	-1.9921	2.02724	1.48904
C	-1.54057	2.38443	2.76255
H	-0.49333	2.62456	2.9218
C	-2.42917	2.43815	3.83283
H	-2.06739	2.71514	4.81904
C	-3.77441	2.1412	3.63777
H	-4.46777	2.17958	4.47303
C	-4.22819	1.77945	2.37248
H	-5.27198	1.52637	2.2174
C	-3.34242	1.71579	1.30314
H	-3.70077	1.41164	0.32634
C	-1.11918	3.63418	-0.72677
C	-1.41714	4.7669	0.03793
H	-1.56344	4.67209	1.11026
C	-1.53478	6.01243	-0.57008
H	-1.77207	6.88576	0.03044
C	-1.35515	6.13825	-1.94631
H	-1.45269	7.11062	-2.42009
C	-1.06527	5.01434	-2.71451
H	-0.94043	5.10418	-3.78928
C	-0.95368	3.76676	-2.10767
H	-0.7629	2.88196	-2.70958
C	0.7797	2.11331	0.76125
C	1.62716	3.21437	0.63978
H	1.27756	4.11105	0.13831
C	2.9234	3.15955	1.14481
H	3.57696	4.02088	1.04549
C	3.3864	2.01228	1.78324
H	4.39781	1.99502	2.1755
C	2.56553	0.89529	1.93343
C	1.27845	0.985	1.41284
C	2.93265	-0.37153	2.704
C	2.22981	-1.54281	2.02017
C	2.7458	-2.83234	1.91506
H	3.74135	-3.05002	2.28663
C	1.99819	-3.85223	1.33265
H	2.41628	-4.85143	1.25969
C	0.72094	-3.60193	0.84129
H	0.15976	-4.40444	0.37492

C	0.17243	-2.31911	0.91246
C	0.95593	-1.32579	1.50099
C	4.44684	-0.58314	2.78706
H	4.89536	-0.70437	1.79651
H	4.6787	-1.46728	3.38883
H	4.9298	0.26297	3.28491
C	2.35947	-0.23643	4.13617
H	1.27733	-0.07068	4.11466
H	2.82533	0.61176	4.65001
H	2.55694	-1.14846	4.71003
C	-2.58999	-1.66649	1.48864
C	-2.19759	-1.3598	2.79426
H	-1.1516	-1.20631	3.03242
C	-3.14425	-1.24745	3.80785
H	-2.82105	-1.00361	4.81546
C	-4.49354	-1.44371	3.53452
H	-5.23039	-1.35968	4.3281
C	-4.89434	-1.74161	2.23499
H	-5.94552	-1.89452	2.00854
C	-3.95209	-1.84645	1.21842
H	-4.2789	-2.07747	0.21058
C	-1.93883	-3.3483	-0.71973
C	-2.40217	-4.45525	0.00471
H	-2.48228	-4.39544	1.08661
C	-2.76556	-5.62475	-0.65206
H	-3.12371	-6.47592	-0.08041
C	-2.67381	-5.70375	-2.04169
H	-2.9611	-6.61754	-2.55357
C	-2.22447	-4.60788	-2.77004
H	-2.16331	-4.65691	-3.85291
C	-1.86143	-3.43455	-2.11153
H	-1.53397	-2.57252	-2.68534
O	0.04961	-0.37473	-4.4161
Pd	-0.99424	0.12395	-1.63227
C	-0.19903	-0.19035	-3.29389
C	1.18328	-0.12642	-1.98217
C	-2.9682	0.36785	-2.17881
C	1.79019	-1.35101	-1.69362
C	1.98246	1.02107	-2.01382
C	3.13517	-1.414	-1.35364
H	1.20434	-2.26528	-1.70855
C	3.33232	0.95612	-1.70015
H	1.54499	1.97875	-2.28236
C	3.92822	-0.26014	-1.3444
H	3.56093	-2.36871	-1.06034
H	3.92999	1.8618	-1.73906
F	-3.2846	-0.14395	-3.40151
F	-3.87489	-0.22607	-1.32786
F	-3.37843	1.67346	-2.23199
C	5.36221	-0.32563	-0.96687
C	5.98161	0.74649	-0.3115
C	6.12933	-1.46489	-1.24039
C	7.31765	0.6777	0.06851
H	5.40025	1.63135	-0.07163
C	7.46499	-1.53581	-0.85942
H	5.68248	-2.29621	-1.77754
C	8.065	-0.46574	-0.20104
H	7.77483	1.51933	0.58097
H	8.04111	-2.42793	-1.08722
H	9.10827	-0.52134	0.09502

E(RM06L) = -3305.67168642
E(RM06) = -3304.16028605

25 °C:
Zero-point correction= 0.801912 (Hartree/Particle)
Thermal correction to Energy= 0.854874
Thermal correction to Enthalpy= 0.855818
Thermal correction to Gibbs Free Energy= 0.712560
Sum of electronic and zero-point Energies= -3303.350561
Sum of electronic and thermal Energies= -3303.297600
Sum of electronic and thermal Enthalpies= -3303.296656
Sum of electronic and thermal Free Energies= -3303.439914

160 °C:
Zero-point correction= 0.801912 (Hartree/Particle)
Thermal correction to Energy= 0.908502
Thermal correction to Enthalpy= 0.909873
Thermal correction to Gibbs Free Energy= 0.637619
Sum of electronic and zero-point Energies= -3303.350561
Sum of electronic and thermal Energies= -3303.243972
Sum of electronic and thermal Enthalpies= -3303.242600
Sum of electronic and thermal Free Energies= -3303.514855

Reductive elimination from ArCO-[$Pd^{(II)}$]-CF_3 transition states

Reductive elimination from phenylCO-$Pd^{(II)}$-CF_3

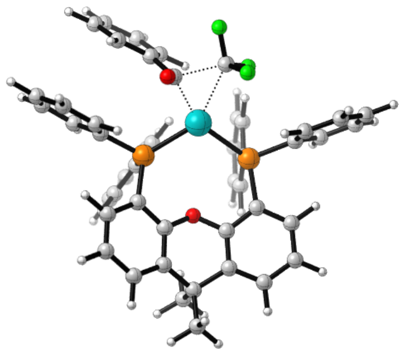

P	1.19868	-1.28127	-0.18882
P	-1.7964	1.21035	0.01794
C	0.27838	-2.71244	-0.89198
C	1.0734	-1.64842	1.60649
C	2.93099	-1.7206	-0.59339
C	-3.17013	0.03687	-0.33052
C	-1.73614	1.28855	1.85025
C	-2.59493	2.79697	-0.43747
C	-1.09744	-2.75762	-0.66881
O	-1.63936	-1.71466	0.03903
C	-2.89818	-1.33048	-0.34354
C	0.84851	-3.77636	-1.59401
C	0.05255	-4.81551	-2.06647
C	-1.31728	-4.82739	-1.82028
C	-1.91828	-3.79872	-1.09713
C	-4.4659	0.43563	-0.67356
C	-5.4192	-0.50528	-1.0443
C	-5.09897	-1.85997	-1.0801
C	-3.82727	-2.29987	-0.72146
C	-2.4408	0.41989	2.68291
C	-2.30715	0.51035	4.06729
C	-1.48019	1.47611	4.63045
C	-0.77238	2.34847	3.80416
C	-0.89202	2.24981	2.42328
C	-3.29629	3.59981	0.4658
C	-3.91104	4.77134	0.03124
C	-3.83871	5.14488	-1.30819
C	-3.14698	4.34521	-2.21467
C	-2.52528	3.18014	-1.78022
C	0.95247	-2.95715	2.08739
C	0.87653	-3.19999	3.45448
C	0.92943	-2.1396	4.35706
C	1.05773	-0.83697	3.88675
C	1.12359	-0.59326	2.51812
C	3.87572	-2.06147	0.37599
C	5.18971	-2.33989	0.00783
C	5.56874	-2.28973	-1.32989
C	4.63266	-1.94334	-2.30213
C	3.32479	-1.64713	-1.93586
H	1.91732	-3.79184	-1.77632
H	0.50724	-5.63027	-2.62108
H	-4.72235	1.48941	-0.67318
H	-6.41837	-0.18019	-1.31675
H	-5.8542	-2.57512	-1.38838
H	-3.10008	-0.3301	2.25676
H	-2.8577	-0.17479	4.70496
H	-1.38281	1.54958	5.70977
H	-0.12323	3.10534	4.23513

H	-0.33657	2.92734	1.78053
H	-3.36467	3.31217	1.51069
H	-4.44988	5.3918	0.74141
H	-4.31641	6.06047	-1.64427
H	-3.07779	4.63448	-3.25884
H	-1.96667	2.57023	-2.48402
H	0.9168	-3.79013	1.39104
H	0.77647	-4.21935	3.8161
H	0.86618	-2.33002	5.42456
H	1.08628	-0.00314	4.58053
H	1.20198	0.42503	2.15459
H	3.59299	-2.10273	1.42285
H	5.91772	-2.59602	0.77175
H	6.59385	-2.50935	-1.6141
H	4.92326	-1.88264	-3.34666
H	2.61048	-1.33846	-2.69355
H	-1.91388	-5.65564	-2.18789
C	-3.38267	-3.76002	-0.66279
C	2.32196	1.52591	-1.4535
C	1.20474	3.13738	-1.33228
F	0.48874	3.69781	-0.30167
F	0.48511	3.31859	-2.45597
F	2.27603	3.95567	-1.44728
Pd	0.51778	0.9108	-0.76263
C	-4.266	-4.66621	-1.52394
H	-3.94076	-5.70795	-1.44552
H	-5.30294	-4.63828	-1.17577
H	-4.24288	-4.37096	-2.57801
C	-3.45792	-4.23327	0.80987
H	-4.48994	-4.17455	1.17311
H	-3.11476	-5.27054	0.89121
H	-2.82892	-3.6136	1.45657
C	3.41437	1.60257	-0.42311
C	4.70223	1.22057	-0.79685
C	3.15886	1.96309	0.90076
C	5.71663	1.1656	0.15143
H	4.88426	0.94832	-1.83032
C	4.17304	1.90994	1.84944
H	2.16309	2.29183	1.18667
C	5.45268	1.50239	1.47707
H	6.71287	0.84842	-0.14252
H	3.96629	2.18603	2.87937
H	6.24372	1.45348	2.21989
O	2.54331	1.29533	-2.62796

E(RM06L) = -3074.56681876
E(RM06) = -3073.21164853

25 °C:
Zero-point correction= 0.722102 (Hartree/Particle)
Thermal correction to Energy= 0.769618
Thermal correction to Enthalpy= 0.770562
Thermal correction to Gibbs Free Energy= 0.639965
Sum of electronic and zero-point Energies= -3072.444849
Sum of electronic and thermal Energies= -3072.397333
Sum of electronic and thermal Enthalpies= -3072.396389
Sum of electronic and thermal Free Energies= -3072.526986

160 °C:
Zero-point correction= 0.722094 (Hartree/Particle)
Thermal correction to Energy= 0.817904
Thermal correction to Enthalpy= 0.819276
Thermal correction to Gibbs Free Energy= 0.571719
Sum of electronic and zero-point Energies= -3072.444857
Sum of electronic and thermal Energies= -3072.349046
Sum of electronic and thermal Enthalpies= -3072.347675
Sum of electronic and thermal Free Energies= -3072.595232

Reductive elimination from [1,1'-biphenyl]-4-CO-Pd$^{(II)}$-CF$_3$

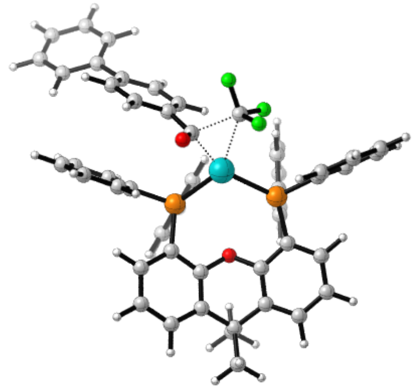

P	0.33945	-1.3845	-0.43635
P	-2.36421	1.33761	0.21725
C	-0.78892	-2.77014	-0.88488
C	0.52075	-1.65875	1.3703
C	1.93651	-1.98608	-1.10972
C	-3.86546	0.2758	0.13504
C	-1.98152	1.42136	2.00991
C	-3.10836	2.97799	-0.12999
C	-2.10462	-2.69394	-0.42886
O	-2.43521	-1.58623	0.30952
C	-3.70738	-1.10915	0.12648
C	-0.43056	-3.90526	-1.61555
C	-1.37198	-4.89199	-1.89208
C	-2.67659	-4.7787	-1.42122
C	-3.06432	-3.6768	-0.66152
C	-5.16459	0.77333	-0.00554
C	-6.23654	-0.09381	-0.18119
C	-6.03435	-1.47091	-0.21539
C	-4.75954	-2.0073	-0.0519
C	-2.56235	0.57933	2.95783
C	-2.19077	0.66541	4.29787
C	-1.24778	1.60249	4.70525
C	-0.66109	2.44697	3.76342
C	-1.0149	2.34868	2.42304
C	-3.55366	3.85744	0.86007
C	-4.15238	5.06461	0.50821
C	-4.32272	5.39734	-0.83286
C	-3.8889	4.52116	-1.8247
C	-3.28018	3.32139	-1.47465
C	0.37361	-2.9243	1.94835
C	0.552	-3.09762	3.3167
C	0.89092	-2.01135	4.12076
C	1.04509	-0.75142	3.55213
C	0.85242	-0.57564	2.18542
C	3.01009	-2.35479	-0.29721
C	4.20517	-2.78862	-0.86611
C	4.33738	-2.86437	-2.24878
C	3.27532	-2.4828	-3.0664
C	2.08764	-2.03215	-2.50193
H	0.58554	-4.01795	-1.97641
H	-1.08219	-5.76228	-2.47251
H	-5.33566	1.84415	0.00023
H	-7.23787	0.30769	-0.30085
H	-6.88486	-2.12728	-0.36631
H	-3.31067	-0.14701	2.65732
H	-2.64522	-0.00192	5.02407
H	-0.96784	1.6752	5.7522
H	0.07706	3.18144	4.07245
H	-0.55062	3.00353	1.69049
H	-3.43415	3.60395	1.9089
H	-4.48859	5.74504	1.28511
H	-4.78945	6.33967	-1.10421
H	-4.01254	4.7758	-2.87283
H	-2.92407	2.64968	-2.25015
H	0.11942	-3.77869	1.32749
H	0.42972	-4.08362	3.75554
H	1.03285	-2.14871	5.18873
H	1.29798	0.10296	4.17124

H	0.94919	0.41175	1.74839
H	2.92022	-2.30408	0.78281
H	5.03095	-3.0721	-0.2198
H	5.26728	-3.21125	-2.69039
H	3.37652	-2.51939	-4.147
H	1.27775	-1.69996	-3.14579
H	-3.39039	-5.56497	-1.64297
C	-4.42857	-3.49717	0.0016
C	1.34025	1.26221	-2.12024
C	0.45631	2.98158	-1.74747
F	0.0616	3.59411	-0.58233
F	-0.48111	3.25461	-2.67427
F	1.55043	3.68803	-2.11561
Pd	-0.28274	0.83388	-0.98701
C	-5.51096	-4.35255	-0.66185
H	-5.26374	-5.41557	-0.58451
H	-6.4717	-4.22078	-0.15522
H	-5.63664	-4.09887	-1.71942
C	-4.29951	-3.91294	1.4878
H	-5.25031	-3.75147	2.00764
H	-4.03514	-4.9736	1.56133
H	-3.52427	-3.33122	1.99627
C	2.67045	1.23166	-1.42562
C	3.77639	0.76473	-2.13334
C	2.81159	1.5566	-0.07722
C	4.98992	0.57654	-1.49005
H	3.65898	0.53224	-3.18587
C	4.0248	1.36375	0.56803
H	1.96153	1.9531	0.47128
C	5.12907	0.85342	-0.1251
H	5.84409	0.2101	-2.05197
H	4.11008	1.57904	1.62953
O	1.21208	1.00059	-3.30261
C	6.41347	0.59717	0.57146
C	6.90367	1.48549	1.53545
C	7.1614	-0.54992	0.28009
C	8.10486	1.23384	2.19033
H	6.35043	2.3946	1.75475
C	8.36254	-0.80249	0.93419
H	6.78425	-1.25785	-0.45284
C	8.83878	0.08839	1.89269
H	8.47276	1.94018	2.92891
H	8.92488	-1.7017	0.69905
H	9.77753	-0.10708	2.40242

E(RM06L) = -3305.67329744
E(RM06) = -3304.16723501

25 °C:
Zero-point correction= 0.804331 (Hartree/Particle)
Thermal correction to Energy= 0.856543
Thermal correction to Enthalpy= 0.857487
Thermal correction to Gibbs Free Energy= 0.715768
Sum of electronic and zero-point Energies= -3303.342183
Sum of electronic and thermal Energies= -3303.289971
Sum of electronic and thermal Enthalpies= -3303.289027
Sum of electronic and thermal Free Energies= -3303.430746

160 °C:
Zero-point correction= 0.804331 (Hartree/Particle)
Thermal correction to Energy= 0.909805
Thermal correction to Enthalpy= 0.911177
Thermal correction to Gibbs Free Energy= 0.641600
Sum of electronic and zero-point Energies= -3303.342183
Sum of electronic and thermal Energies= -3303.236709
Sum of electronic and thermal Enthalpies= -3303.235337
Sum of electronic and thermal Free Energies= -3303.504914

Ar-[Pd$^{(II)}$]-CF_3 intermediates

Phenyl-Pd$^{(II)}$-CF_3 (cis)

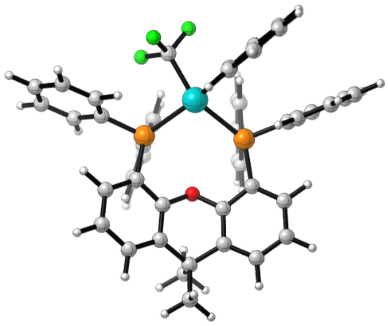

P	-1.75674	-0.94574	-0.14107
P	1.53451	0.95634	0.21922
C	-2.61651	0.32614	-1.15869
C	-2.30984	-0.53686	1.55949
C	-2.75845	-2.40967	-0.59839
C	1.02934	2.63872	-0.33282
C	1.20048	0.95126	2.02359
C	3.35505	1.12697	0.10429
C	-2.21997	1.65147	-1.01557
O	-1.18	1.8799	-0.15285
C	-0.32486	2.87932	-0.53615
C	-3.67055	0.06915	-2.0397
C	-4.27027	1.11109	-2.7407
C	-3.8572	2.42755	-2.55122
C	-2.82444	2.72562	-1.66513
C	1.91588	3.67212	-0.65166
C	1.44088	4.8603	-1.19629
C	0.0807	5.03845	-1.43916
C	-0.83534	4.04412	-1.10525
C	0.52847	1.98046	2.68294
C	0.28546	1.89658	4.05187
C	0.72642	0.79483	4.77668
C	1.3965	-0.23847	4.12572
C	1.6177	-0.16886	2.7559
C	4.18203	1.30907	1.21257
C	5.56223	1.41163	1.04697
C	6.12269	1.34186	-0.22354
C	5.29941	1.17676	-1.33615
C	3.92569	1.06977	-1.17242
C	-3.31321	0.39454	1.84087
C	-3.67458	0.66109	3.15837
C	-3.04655	-0.00852	4.20446
C	-2.05537	-0.94612	3.92933
C	-1.6817	-1.20461	2.6154
C	-3.89925	-2.78331	0.11275
C	-4.66361	-3.8672	-0.3108
C	-4.29841	-4.57751	-1.45072
C	-3.16195	-4.20589	-2.16574
C	-2.3924	-3.12929	-1.73914
H	-4.02125	-0.94641	-2.1843
H	-5.07746	0.89499	-3.43335
H	2.98037	3.53885	-0.49679
H	2.14053	5.65068	-1.44892
H	-0.26166	5.96404	-1.89002
H	0.19453	2.85538	2.13456
H	-0.24722	2.70021	4.55137
H	0.54082	0.73537	5.84513
H	1.73487	-1.10883	4.68006
H	2.12127	-0.98917	2.25141
H	3.7568	1.36693	2.20921
H	6.19786	1.54578	1.91738
H	7.19907	1.41343	-0.34903
H	5.72891	1.1041	-2.33013
H	3.29414	0.90673	-2.04021
H	-3.82013	0.91394	1.03324
H	-4.45143	1.39149	3.3654
H	-3.32814	0.19998	5.23256
H	-1.55795	-1.46947	4.73985

H -0.90751 -1.93509 2.40514
H -4.19037 -2.23262 1.00213
H -5.54597 -4.15643 0.25256
H -4.89404 -5.4245 -1.77845
H -2.86406 -4.76344 -3.04845
H -1.49177 -2.85852 -2.28338
H -4.35428 3.2238 -3.09548
C -2.34999 4.12759 -1.28235
C 2.53591 -1.77403 -1.00502
C 3.65903 -2.04327 -0.21888
C 2.68984 -1.79187 -2.39611
C 4.90209 -2.28539 -0.79793
H 3.57512 -2.06186 0.86376
C 3.93287 -2.03468 -2.98184
H 1.83145 -1.61135 -3.04105
C 5.04676 -2.27675 -2.18298
H 5.76234 -2.47776 -0.16188
H 4.02662 -2.04165 -4.0653
H 6.01661 -2.46443 -2.63551
C 0.548 -3.28648 0.01751
F -0.57653 -3.62292 0.74238
F 1.55681 -3.74326 0.81205
F 0.52679 -4.11668 -1.05623
Pd 0.67408 -1.27332 -0.35404
C -2.98586 4.49229 0.08178
H -2.62834 5.47274 0.4153
H -4.077 4.52879 -0.0097
H -2.72949 3.75403 0.84813
C -2.75131 5.1792 -2.31896
H -3.83979 5.22642 -2.42019
H -2.42383 6.17483 -2.0043
H -2.31796 4.96455 -3.3012

E(RM06L) = -2961.21036188
E(RM06) = -2959.89940693

25 °C:
Zero-point correction= 0.712421 (Hartree/Particle)
Thermal correction to Energy= 0.758689
Thermal correction to Enthalpy= 0.759633
Thermal correction to Gibbs Free Energy= 0.631705
Sum of electronic and zero-point Energies= -2959.174967
Sum of electronic and thermal Energies= -2959.128700
Sum of electronic and thermal Enthalpies= -2959.127755
Sum of electronic and thermal Free Energies= -2959.255683

160 °C:
Zero-point correction= 0.712421 (Hartree/Particle)
Thermal correction to Energy= 0.805827
Thermal correction to Enthalpy= 0.807199
Thermal correction to Gibbs Free Energy= 0.564920
Sum of electronic and zero-point Energies= -2959.174967
Sum of electronic and thermal Energies= -2959.081561
Sum of electronic and thermal Enthalpies= -2959.080190
Sum of electronic and thermal Free Energies= -2959.322468

Phenyl-Pd$^{(II)}$-CF_3 (trans)

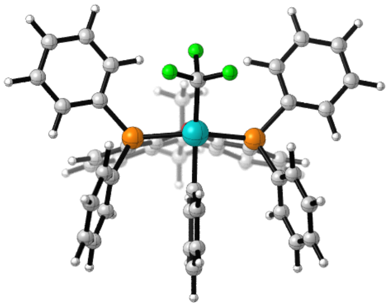

Pd -0.00004 0.58714 -0.91079
P 2.23348 0.20473 -0.40696
P -2.2335 0.20475 -0.4069
O -0.00001 -1.70771 0.55408
C -3.47139 -0.4964 -1.56791
C -3.04969 -1.48901 -2.45661
H -2.0011 -1.75796 -2.51101
C -3.96563 -2.11232 -3.29667
H -3.62334 -2.87373 -3.99094
C -5.31001 -1.75146 -3.25948
H -6.02264 -2.23472 -3.92153
C -5.7368 -0.76497 -2.37536
H -6.78376 -0.47802 -2.34043
C -4.82331 -0.14142 -1.52992
H -5.16771 0.62766 -0.84539
C -3.06299 1.68119 0.27883
C -3.21679 1.92277 1.6437
H -2.91862 1.17437 2.36915
C -3.70568 3.14871 2.08602
H -3.80517 3.33034 3.15169
C -4.04921 4.13875 1.17164
H -4.42872 5.09461 1.52074
C -3.89422 3.90489 -0.19302
H -4.15259 4.67517 -0.91341
C -3.39523 2.68649 -0.63771
H -3.25483 2.51724 -1.70279
C -2.27894 -1.08308 0.91027
C -3.47534 -1.31869 1.59496
H -4.35567 -0.73626 1.33926
C -3.5509 -2.26848 2.60256
H -4.4846 -2.42482 3.13302
C -2.43144 -3.03153 2.9211
H -2.50747 -3.77773 3.70435
C -1.2332 -2.86643 2.23262
C -1.17642 -1.88308 1.23979
C 0. -3.74728 2.424
C 1.23319 -2.86642 2.2326
C 2.43143 -3.03152 2.92108
H 2.50747 -3.77773 3.70432
C 3.55089 -2.26848 2.60253
H 4.48459 -2.42481 3.13299
C 3.47532 -1.3187 1.59492
H 4.35565 -0.73626 1.33921
C 2.27893 -1.08309 0.91022
C 1.1764 -1.88307 1.23977
C 0.00003 -4.45036 3.78458
H 0. -3.73266 4.61151
H 0.87511 -5.09898 3.88378
H -0.875 -5.09905 3.88378
C 0. -4.82153 1.30812
H -0.00002 -4.36144 0.31568
H -0.89084 -5.45337 1.3933
H 0.89085 -5.45335 1.39327
C 3.47143 -0.49632 -1.56797
C 4.82338 -0.14144 -1.52977
H 5.16774 0.62756 -0.84513
C 5.73692 -0.76497 -2.37516
H 6.78391 -0.4781 -2.34008
C 5.31017 -1.75135 -3.25943
H 6.02285 -2.23461 -3.92142
C 3.96577 -2.1121 -3.29683
H 3.62351 -2.87342 -3.99121
C 3.04976 -1.4888 -2.45683
H 2.00116 -1.75764 -2.5114
C 3.06293 1.68119 0.2788
C 3.21682 1.92271 1.64367
H 2.91874 1.17426 2.36911
C 3.70569 3.14865 2.08601
H 3.80525 3.33024 3.15168
C 4.04911 4.13874 1.17166
H 4.4286 5.09461 1.52077
C 3.89404 3.90494 -0.19301
H 4.15232 4.67527 -0.91338
C 3.39508 2.68654 -0.63772
H 3.25462 2.51733 -1.70279
C -0.00001 1.56701 0.93796
C 0.00003 1.0891 2.25181
C 0.00001 2.9624 0.77402
C 0.00007 1.95313 3.35009
H 0.00003 0.0227 2.45514
C 0.00005 3.83278 1.85841
H -0.00001 3.38584 -0.22982
C 0.00008 3.32912 3.15886

H	0.00009	1.54096	4.35683
H	0.00006	4.90657	1.68954
H	0.00011	4.00507	4.00984
C	-0.00004	0.08353	-2.91447
F	1.07793	0.59928	-3.58972
F	0.00002	-1.25697	-3.27077
F	-1.07796	0.59921	-3.58984

E(RM06L) = -2961.20458157
E(RM06) = -2959.89155497

25 °C:
Zero-point correction= 0.713404 (Hartree/Particle)
Thermal correction to Energy= 0.759422
Thermal correction to Enthalpy= 0.760366
Thermal correction to Gibbs Free Energy= 0.634125
Sum of electronic and zero-point Energies= -2959.169806
Sum of electronic and thermal Energies= -2959.123788
Sum of electronic and thermal Enthalpies= -2959.122844
Sum of electronic and thermal Free Energies= -2959.249085

160 °C:
Zero-point correction= 0.713404 (Hartree/Particle)
Thermal correction to Energy= 0.806507
Thermal correction to Enthalpy= 0.807878
Thermal correction to Gibbs Free Energy= 0.568113
Sum of electronic and zero-point Energies= -2959.169806
Sum of electronic and thermal Energies= -2959.076703
Sum of electronic and thermal Enthalpies= -2959.075332
Sum of electronic and thermal Free Energies= -2959.315097

[1,1'-Biphenyl]-4-Pd$^{(II)}$-CF$_3$

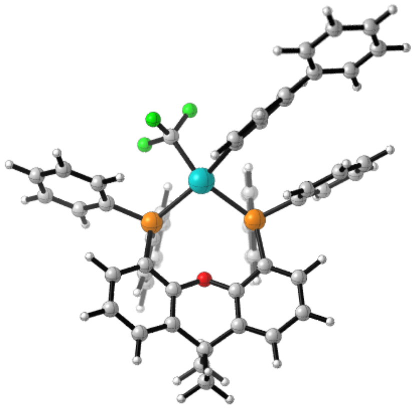

P	2.18551	1.30009	-0.27217
P	-0.44585	-1.36326	0.51897
C	3.07447	0.34254	-1.57168
C	3.16194	0.93172	1.23653
C	2.71434	2.98796	-0.74896
C	0.27831	-2.82789	-0.32912
C	0.27838	-1.40662	2.20403
C	-2.16407	-1.9598	0.75097
C	3.01015	-1.04583	-1.51773
O	2.24899	-1.57401	-0.50842
C	1.57005	-2.71864	-0.8328
C	3.84668	0.90455	-2.59211
C	4.4975	0.08779	-3.51167
C	4.41929	-1.29892	-3.41257
C	3.67947	-1.89665	-2.39469
C	-0.40666	-4.0206	-0.58189
C	0.18084	-5.01689	-1.35368
C	1.45452	-4.84278	-1.8904
C	2.18117	-3.68339	-1.63129
C	1.29968	-2.27866	2.58138
C	1.82556	-2.22843	3.87011
C	1.32365	-1.32259	4.79826
C	0.30346	-0.44863	4.43019
C	-0.20425	-0.47893	3.13743
C	-2.67132	-2.39363	1.97557
C	-3.99641	-2.81476	2.0753
C	-4.81839	-2.81262	0.95367
C	-4.31367	-2.39198	-0.27579
C	-2.99484	-1.97212	-0.37572
C	4.37938	0.24593	1.21926
C	5.06401	0.00095	2.40624
C	4.54529	0.44874	3.6176
C	3.33811	1.14117	3.63926
C	2.64433	1.37534	2.45765
C	3.83869	3.60354	-0.19834
C	4.23443	4.8627	-0.64203
C	3.51643	5.50914	-1.64377
C	2.39514	4.89651	-2.19909
C	1.99249	3.6438	-1.75028
H	3.93626	1.98159	-2.67478
H	5.08199	0.538	-4.30769
H	-1.409	-4.16162	-0.19415
H	-0.364	-5.93485	-1.54989
H	1.87988	-5.62533	-2.50993
H	1.68887	-3.00412	1.87418
H	2.62781	-2.90528	4.14797
H	1.73418	-1.28962	5.80317
H	-0.08725	0.27029	5.14428
H	-0.9837	0.22098	2.84964
H	-2.04019	-2.39999	2.85833
H	-4.38342	-3.14589	3.03459
H	-5.85324	-3.13299	1.03465
H	-4.95689	-2.36387	-1.14973
H	-2.61607	-1.61933	-1.32988
H	4.80182	-0.09774	0.28
H	6.00781	-0.53611	2.38119
H	5.07992	0.25782	4.54361
H	2.92337	1.48931	4.58003
H	1.70255	1.91382	2.47952
H	4.40574	3.10444	0.58139
H	5.10646	5.33774	-0.20234
H	3.82555	6.49178	-1.98796
H	1.82327	5.40042	-2.97227
H	1.10349	3.18036	-2.16911
H	4.95071	-1.91315	-4.13198
C	3.60255	-3.39644	-2.11086
C	-2.26942	1.1135	-0.11247
C	-3.19157	1.04401	0.93475
C	-2.77583	1.15209	-1.4158
C	-4.55615	0.95115	0.69179
H	-2.85062	1.05642	1.96576
C	-4.14339	1.05645	-1.66467
H	-2.09562	1.23263	-2.26167
C	-5.05752	0.93524	-0.61429
H	-5.24563	0.89703	1.53057
H	-4.49995	1.04355	-2.69229
C	-0.48126	3.02282	0.54814
F	0.66216	3.56377	1.09915
F	-1.40802	3.23908	1.52108
F	-0.81446	3.86781	-0.46184
Pd	-0.24544	1.04885	0.04712
C	4.5811	-3.71823	-0.95477
H	4.52153	-4.78127	-0.69664
H	5.6093	-3.4866	-1.25374
H	4.34335	-3.13318	-0.06042
C	3.97859	-4.23525	-3.33469
H	5.00776	-4.02743	-3.64256
H	3.9332	-5.30315	-3.10095
H	3.31297	-4.03498	-4.18057
C	-6.50783	0.7629	-0.87349
C	-7.27231	-0.10921	-0.08762
C	-7.14874	1.44975	-1.91182
C	-8.63002	-0.28887	-0.33068
H	-6.78705	-0.66451	0.71041
C	-8.50519	1.26681	-2.15995
H	-6.57985	2.1506	-2.51648
C	-9.253	0.39674	-1.37042
H	-9.20311	-0.97164	0.29067
H	-8.98236	1.81386	-2.96831
H	-10.31267	0.25565	-1.56208

E(RM06L) = -3192.31756962
E(RM06) = -3190.85561601

25 °C:
Zero-point correction= 0.794193 (Hartree/Particle)
Thermal correction to Energy= 0.845197
Thermal correction to Enthalpy= 0.846141
Thermal correction to Gibbs Free Energy= 0.706639
Sum of electronic and zero-point Energies= -3190.073717
Sum of electronic and thermal Energies= -3190.022712
Sum of electronic and thermal Enthalpies= -3190.021768
Sum of electronic and thermal Free Energies= -3190.161270

160 °C:
Zero-point correction= 0.794168 (Hartree/Particle)
Thermal correction to Energy= 0.897371
Thermal correction to Enthalpy= 0.898743
Thermal correction to Gibbs Free Energy= 0.633356
Sum of electronic and zero-point Energies= -3190.073742
Sum of electronic and thermal Energies= -3189.970538
Sum of electronic and thermal Enthalpies= -3189.969167
Sum of electronic and thermal Free Energies= -3190.234554

Reductive elimination from Ar-[Pd$^{(II)}$]-CF_3 transition states

Reductive elimination from phenyl-Pd$^{(II)}$-CF_3

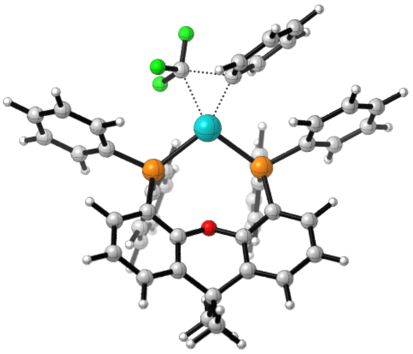

P	-1.7532	-1.04021	-0.08573
P	1.47452	1.05873	0.17458
C	-2.80539	0.15525	-1.00904
C	-2.1464	-0.61686	1.65804
C	-2.69677	-2.59175	-0.35921
C	0.84845	2.64247	-0.53475
C	1.23225	1.27724	1.98308
C	3.2769	1.31035	-0.04792
C	-2.42946	1.49668	-0.97509
O	-1.32334	1.79832	-0.22345
C	-0.52506	2.79932	-0.71205
C	-3.94372	-0.17066	-1.74943
C	-4.64066	0.81671	-2.43978
C	-4.23138	2.14629	-2.38306
C	-3.117	2.5149	-1.6315
C	1.67052	3.68014	-0.98541
C	1.12311	4.79373	-1.61223
C	-0.25327	4.89997	-1.79469
C	-1.10912	3.90188	-1.33524
C	0.39115	2.24418	2.53485
C	0.20936	2.31806	3.91381
C	0.87844	1.44001	4.759
C	1.7198	0.46985	4.2182
C	1.88136	0.37961	2.84118
C	4.13797	1.80105	0.93666
C	5.48942	1.98856	0.65773
C	5.98993	1.70137	-0.60932
C	5.13477	1.22207	-1.59809
C	3.78918	1.02108	-1.31608
C	-3.26143	0.14042	2.02761
C	-3.51168	0.4169	3.36873
C	-2.65758	-0.07139	4.35352
C	-1.54982	-0.83315	3.9933
C	-1.28959	-1.09678	2.6531
C	-3.56984	-3.13772	0.58391
C	-4.26107	-4.31434	0.30348
C	-4.09614	-4.94867	-0.92388
C	-3.23169	-4.40638	-1.87264
C	-2.53128	-3.24007	-1.58798
H	-4.28083	-1.20062	-1.79557
H	-5.51737	0.548	-3.02089
H	2.74533	3.6079	-0.86238
H	1.77629	5.58571	-1.96504
H	-0.65598	5.77588	-2.29238
H	-0.12679	2.9492	1.89354
H	-0.45798	3.06937	4.32535
H	0.74071	1.50651	5.83424
H	2.24568	-0.22325	4.86859
H	2.53353	-0.38327	2.42526
H	3.75508	2.03998	1.92388
H	6.15136	2.36535	1.43223
H	7.04457	1.84832	-0.82357
H	5.51912	0.98127	-2.58441
H	3.13187	0.61523	-2.07963
H	-3.94083	0.51434	1.26703
H	-4.37891	1.01073	3.64348
H	-2.85268	0.14366	5.40022
H	-0.87211	-1.20674	4.75416
H	-0.41381	-1.67589	2.37479
H	-3.71118	-2.64844	1.54247
H	-4.93253	-4.73265	1.04773
H	-4.63423	-5.86714	-1.13954
H	-3.09158	-4.89933	-2.82999
H	-1.84253	-2.83176	-2.32214
H	-4.79684	2.89819	-2.92355
C	-2.63484	3.94601	-1.40145
C	2.45422	-2.10531	-0.77609
C	3.49787	-2.09621	0.16702
C	2.78725	-2.18063	-2.13745
C	4.82273	-2.09783	-0.24103
H	3.26082	-2.11168	1.22719
C	4.12172	-2.18255	-2.54082
H	1.99855	-2.25833	-2.88042
C	5.14358	-2.14211	-1.59865
H	5.61265	-2.06776	0.50403
H	4.35795	-2.23479	-3.60052
H	6.18253	-2.15564	-1.91419
C	1.08363	-3.39743	-0.38653
F	1.99296	-4.38454	-0.187
F	0.39903	-3.79249	-1.48599
F	0.25766	-3.56099	0.70159
Pd	0.66878	-1.13832	-0.39699
C	-3.13859	4.90656	-2.48151
H	-4.23238	4.93333	-2.49407
H	-2.80545	5.92839	-2.27631
H	-2.78461	4.61715	-3.47634
C	-3.16489	4.41563	-0.02397
H	-2.79604	5.42304	0.19803
H	-4.26038	4.43408	-0.02749
H	-2.83649	3.7434	0.77508

E(RM06L) = -2961.17209015
E(RM06) = -2959.86567813

25 °C:
Zero-point correction= 0.711735 (Hartree/Particle)
Thermal correction to Energy= 0.757475
Thermal correction to Enthalpy= 0.758419
Thermal correction to Gibbs Free Energy= 0.631871
Sum of electronic and zero-point Energies= -2959.134164
Sum of electronic and thermal Energies= -2959.088424
Sum of electronic and thermal Enthalpies= -2959.087480
Sum of electronic and thermal Free Energies= -2959.214028

160 °C:
Zero-point correction= 0.711739 (Hartree/Particle)
Thermal correction to Energy= 0.804266
Thermal correction to Enthalpy= 0.805638
Thermal correction to Gibbs Free Energy= 0.565784
Sum of electronic and zero-point Energies= -2959.134160
Sum of electronic and thermal Energies= -2959.041633
Sum of electronic and thermal Enthalpies= -2959.040261
Sum of electronic and thermal Free Energies= -2959.280115

Reductive elimination from [1,1'-biphenyl]-4-Pd$^{(II)}$-CF_3

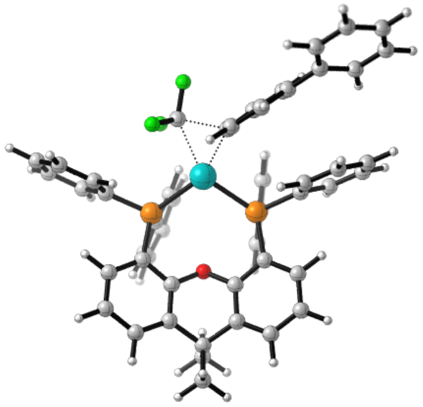

P	2.21063	1.35428	-0.19791
P	-0.43107	-1.39355	0.40346
C	3.32188	0.44888	-1.35129
C	2.9603	0.96986	1.43349
C	2.74274	3.08437	-0.50418
C	0.38861	-2.78236	-0.49249
C	0.13415	-1.61021	2.13825
C	-2.15581	-2.01553	0.42687
C	3.23968	-0.94215	-1.35561
O	2.35853	-1.49889	-0.46573
C	1.71618	-2.63237	-0.89069
C	4.2333	1.03785	-2.23029
C	4.99635	0.24882	-3.08605
C	4.88248	-1.13865	-3.06255
C	4.00503	-1.76462	-2.17895
C	-0.26128	-3.95988	-0.87533
C	0.39425	-4.9089	-1.65143
C	1.71154	-4.70702	-2.0547
C	2.40436	-3.56163	-1.67044
C	1.2329	-2.39153	2.49756
C	1.64646	-2.458	3.82597
C	0.95924	-1.75966	4.81252
C	-0.13951	-0.97691	4.464
C	-0.53804	-0.89168	3.13585
C	-2.73971	-2.70633	1.49095
C	-4.04619	-3.1798	1.38733
C	-4.77493	-2.97574	0.21888
C	-4.19837	-2.28823	-0.84634
C	-2.89883	-1.80905	-0.73982
C	4.24195	0.43248	1.57865
C	4.75714	0.17222	2.84532
C	4.00227	0.45981	3.97901
C	2.72776	1.0026	3.84222
C	2.20457	1.24673	2.57724
C	3.58273	3.7958	0.35464
C	3.93252	5.11269	0.06336
C	3.45851	5.72527	-1.09243
C	2.6281	5.01814	-1.95954
C	2.26673	3.70985	-1.6619
H	4.34068	2.11702	-2.25339
H	5.6927	0.7198	-3.77278
H	-1.29173	-4.12727	-0.58268
H	-0.1273	-5.81334	-1.94843
H	2.19908	-5.45734	-2.66815
H	1.77246	-2.9568	1.74508
H	2.51007	-3.06212	4.08778
H	1.28036	-1.8202	5.8483
H	-0.68227	-0.42477	5.22595
H	-1.39096	-0.27439	2.86782
H	-2.17626	-2.88136	2.40216
H	-4.4915	-3.71402	2.22177
H	-5.79437	-3.34202	0.13857
H	-4.77077	-2.10193	-1.74966
H	-2.45921	-1.25458	-1.56351
H	4.84319	0.2162	0.70028
H	5.75183	-0.25274	2.94552
H	4.4058	0.25881	4.96721
H	2.12888	1.21854	4.72127
H	1.20266	1.65372	2.4731
H	3.96288	3.32631	1.2566
H	4.57865	5.65999	0.74349
H	3.72916	6.75332	-1.31472
H	2.24751	5.49087	-2.86
H	1.60182	3.16902	-2.3294
H	5.49474	-1.73284	-3.73277
C	3.87273	-3.27325	-1.97975
C	-2.17732	1.50879	-0.14693
C	-3.01374	1.27316	0.95906
C	-2.76554	1.52682	-1.42078
C	-4.35917	0.99666	0.78961
H	-2.60353	1.34042	1.96278
C	-4.1177	1.24575	-1.5826
H	-2.16009	1.76518	-2.29085
C	-4.94108	0.97478	-0.48585
H	-4.9793	0.82226	1.66477
H	-4.53728	1.22447	-2.58531
C	-1.05879	3.06244	0.06768
F	-2.10912	3.8334	0.44561
F	-0.65395	3.60466	-1.10541
F	-0.12318	3.38045	1.02403
Pd	-0.19699	0.94304	-0.1277
C	4.37115	-4.06207	-3.19355
H	5.43082	-3.86001	-3.37637
H	4.2861	-5.13836	-3.0164
H	3.80664	-3.81144	-4.09759
C	4.71387	-3.6684	-0.74085
H	4.60359	-4.73913	-0.53676
H	5.77289	-3.45086	-0.91834
H	4.39393	-3.11331	0.14687
C	-6.38289	0.68307	-0.66741
C	-7.02972	-0.27514	0.12381
C	-7.13504	1.35557	-1.63878
C	-8.3803	-0.55564	-0.0552
H	-6.45832	-0.82236	0.86788
C	-8.48468	1.07358	-1.82097
H	-6.66131	2.12623	-2.24055
C	-9.11387	0.11542	-1.03051
H	-8.86027	-1.30661	0.56632
H	-9.04934	1.61271	-2.57635
H	-10.16831	-0.10318	-1.1712

E(RM06L) = -3192.27989796
E(RM06) = -3190.82307730

25 °C:
Zero-point correction= 0.793731 (Hartree/Particle)
Thermal correction to Energy= 0.844081
Thermal correction to Enthalpy= 0.845025
Thermal correction to Gibbs Free Energy= 0.707042
Sum of electronic and zero-point Energies= -3190.033276
Sum of electronic and thermal Energies= -3189.982926
Sum of electronic and thermal Enthalpies= -3189.981982
Sum of electronic and thermal Free Energies= -3190.119965

160 °C:
Zero-point correction= 0.793731 (Hartree/Particle)
Thermal correction to Energy= 0.895870
Thermal correction to Enthalpy= 0.897242
Thermal correction to Gibbs Free Energy= 0.634847
Sum of electronic and zero-point Energies= -3190.033276
Sum of electronic and thermal Energies= -3189.931136
Sum of electronic and thermal Enthalpies= -3189.929764
Sum of electronic and thermal Free Energies= -3190.192159
